# Supplementary material for: Effector‐dependent activation and oligomerization of plant NRC class helper NLRs by sensor NLR immune receptors Rpi‐amr3 and Rpi‐amr1
Source: EMBO J. 2023 Jan 2;42(5):e111484. doi: 10.15252/embj.2022111484 (PMC9975942; doi:10.15252/embj.2022111484)
Supplement: Supplementary file 1 — Expanded View Figures PDF [file EMBJ-42-e111484-s012.pdf]

Expanded View Figures

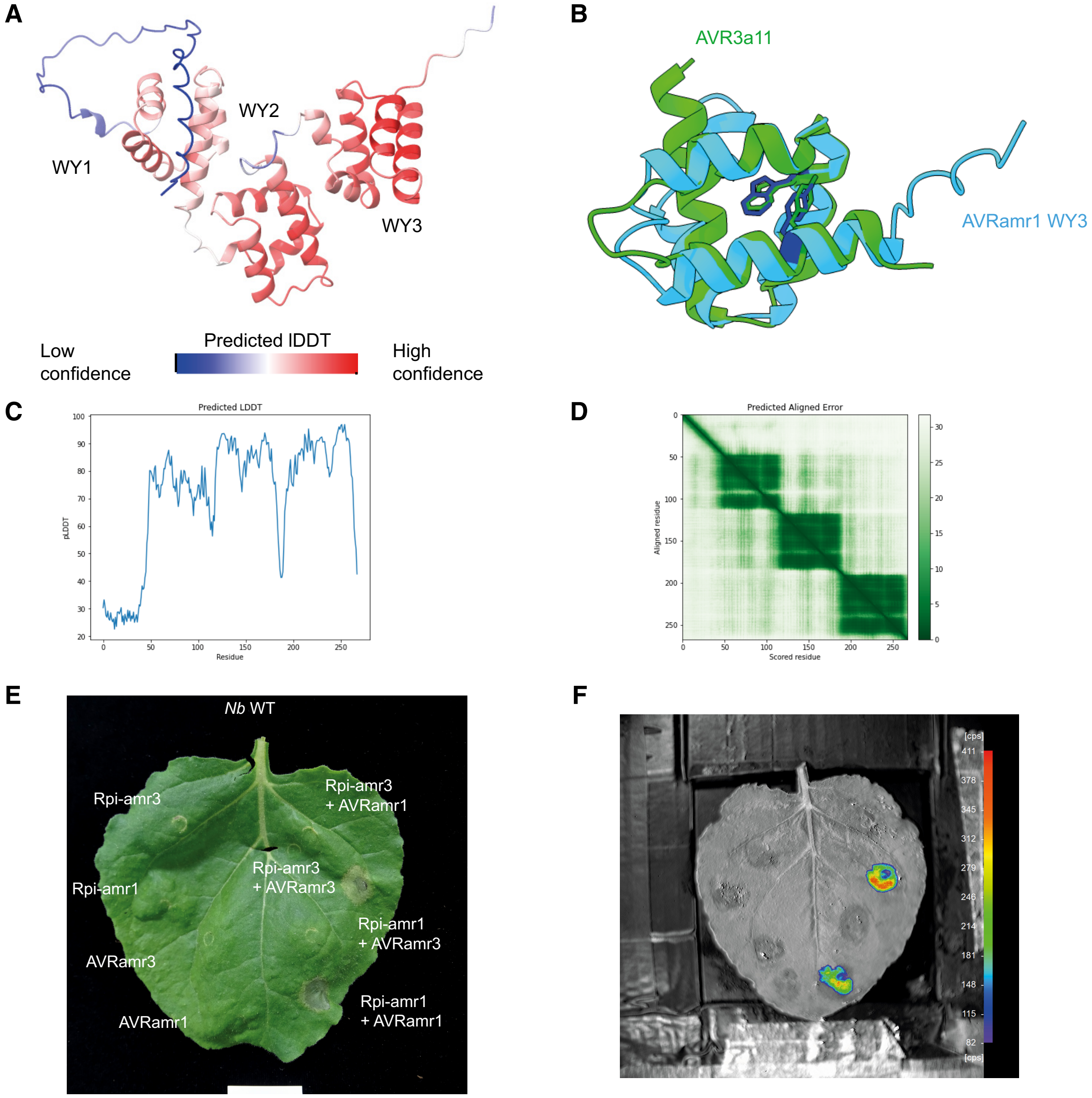

Figure EV1. AVRamr1 is an RXLR effector with WY domains interacting with Rpi-amr1.

A Predicted protein structure of AVRamr1 indicate 3 WY domains. Protein structure model was predicted using AlphaFold and visualized using ChimeraX software. Confidence level of b factors are indicated in colors.

B Third WY domain (WY3, blue) of AVRamr1 shows conserved structure of WY domains with four α-helices as well as the Trp (220W) and Tyr (252Y) residues. The WY3 domain and the WY residues overlap with previously identified WY domain structure of AVR3a11 (PDB ID: 3ZR8) (green).

C Predicted lDDT plot for AVRamr1 structure prediction.

D Predicted aligned error plot for AVRamr1 structure prediction.

E Rpi-amr1 recognizes AVRamr1 and induces HR. Wild-type *N. benthamiana* plants were transiently infiltrated, and leaf samples were imaged at 5 dpi for HR.

F Rpi-amr1 interacts with AVRamr1 *in planta*. Constructs with truncations of luciferase (Nluc or Cluc) were transiently expressed in *nrc2/3/4 KO* N. benthamiana plants and imaged at 3 dpi.

Data information: Experiments were performed with at least three biological replicates with similar results.
Source data are available online for this figure.

 © 2023 The Authors

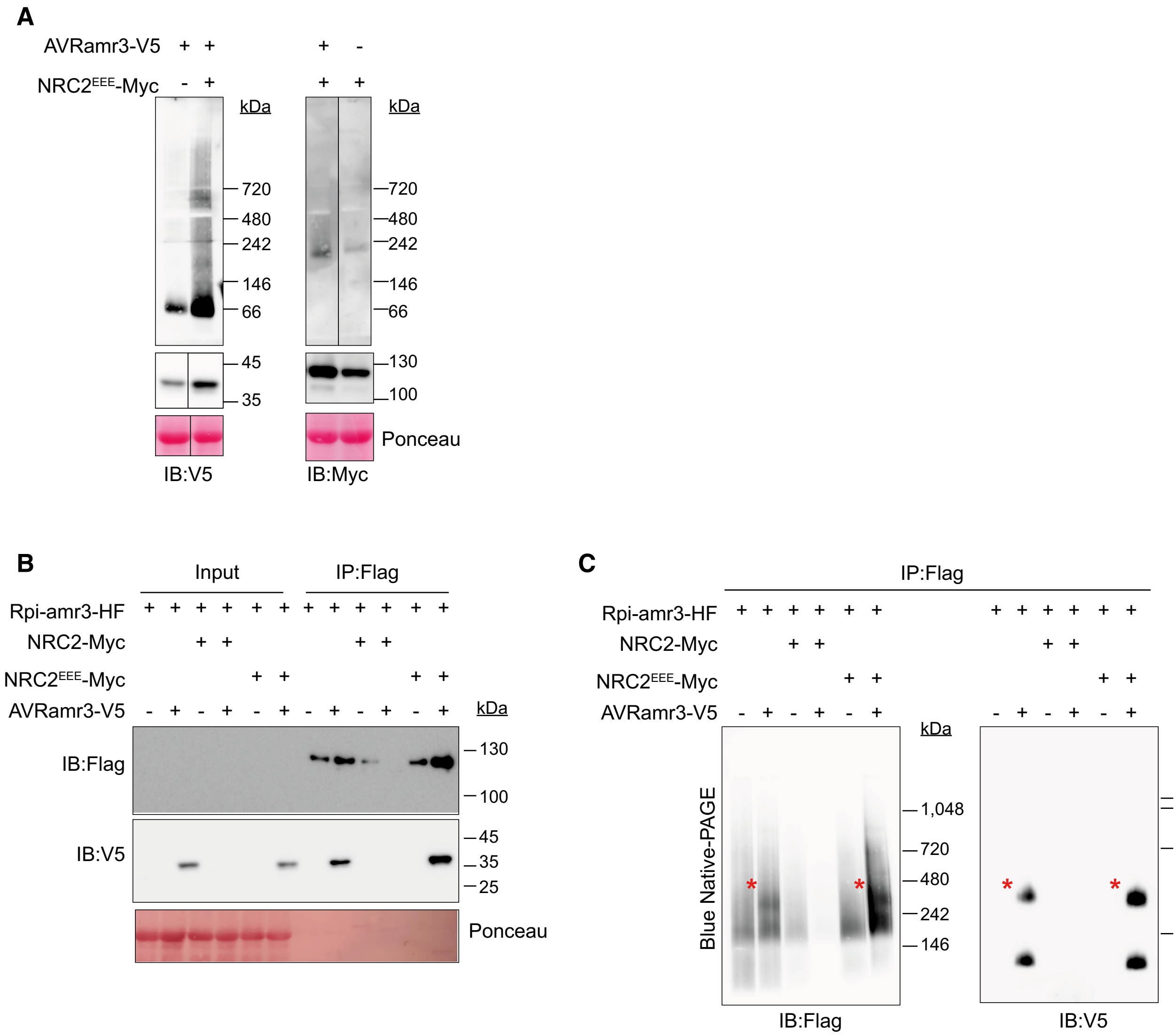

Figure EV2. NRC2 activation leads to degradation of Rpi-amr3 and AVRamr3.

A Protein extracts from *nrc2/3/4* knockout *N. benthamiana* plants expressing AVRamr3-V5 and/or NRC2^{EEE}-Myc were loaded on blue native-PAGE. Aliquot of the protein extracts that were SDS-boiled serve as control.

B NRC2-Myc co-expression leads to degradation of Rpi-amr3 and AVRamr3. Immunoprecipitation with anti-Flag antibody of protein extracts in *nrc2/3/4* knockout *N. benthamiana* plants. Aliquot of samples were SDS-boiled and loaded on SDS–PAGE.

C Immunoprecipitated and eluted samples from (B) were loaded on blue native-PAGE. Rpi-amr3 and AVRamr3 complex is indicated (*, red).

Data information: Ponceau S staining serves as loading control for panels (A and B). Molecular markers are shown on the right. Similar results were observed with at least three biological replicates.
Source data are available online for this figure.

© 2023 The Authors

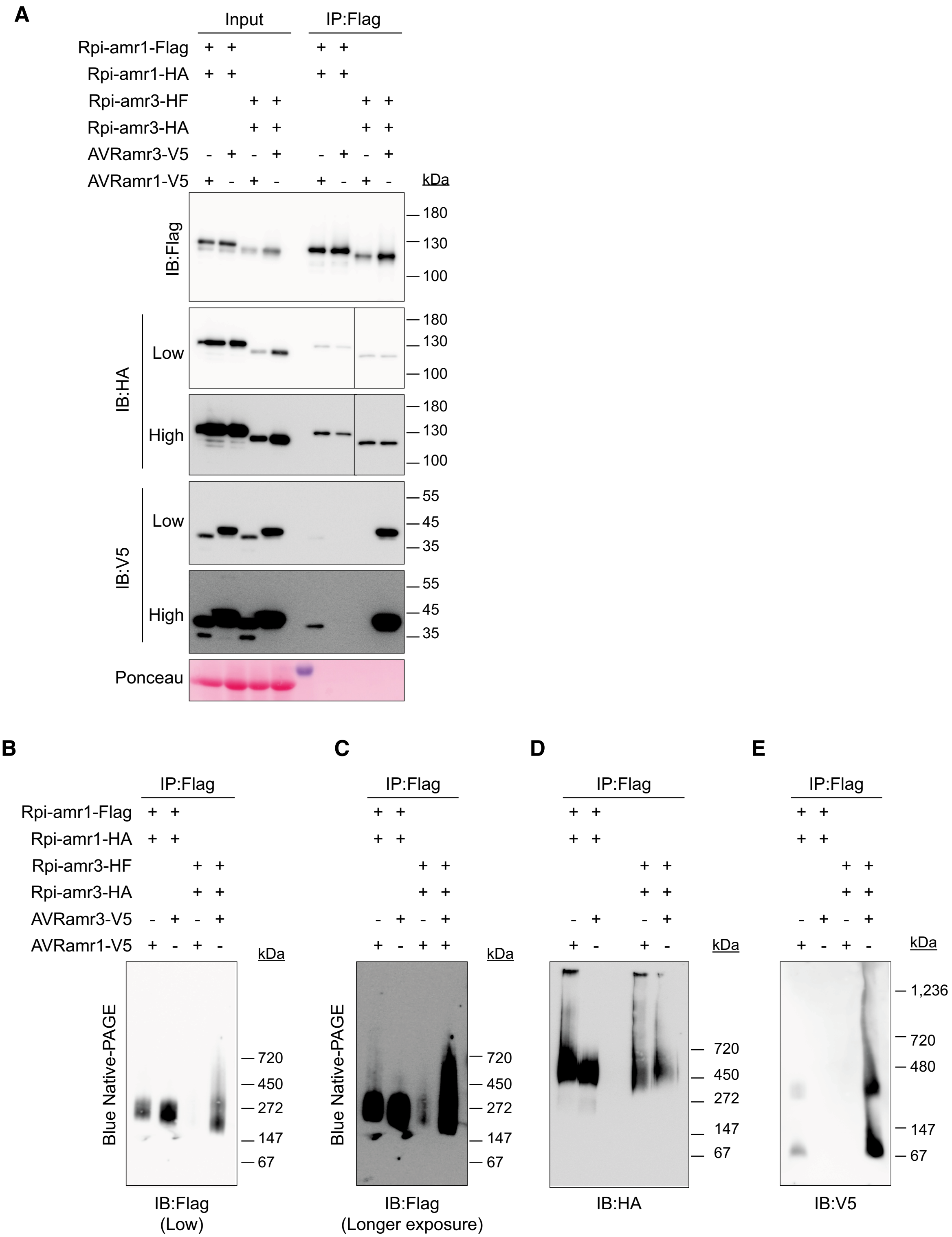

Figure EV3.

 © 2023 The Authors

Figure EV3. Small proportion of Rpi-amr1 or Rpi-amr3 can self-associate.

A Rpi-amr1 and Rpi-amr3 can self-associate weakly *in vivo* regardless of effector co-expression. Protein samples were immunoprecipitated with anti-FLAG antibodies and were blotted for HA-tagged Rpi-amr (anti-HA), and AVRamr (anti-V5). Both low and high exposures of the blots are shown. Solid line in membrane blotted with anti-HA indicate gaps between the samples.

B, C Rpi-amr1 and Rpi-amr3 are mostly monomers *in vivo*. Immunoprecipitated samples of (A) were loaded on blue native-PAGE. High exposure versions of (B) are shown in (C).

D Rpi-amr1 and Rpi-amr3 can self-associate weakly *in vivo* regardless of effector co-expression and form slower-migrating protein complexes. Protein samples were immunoprecipitated with anti-FLAG antibodies, loaded onto blue native-PAGE, and were blotted for HA-tagged Rpi-amr (anti-HA).

E AVRamr1 and AVRamr3 form a protein complex with Rpi-amr1 and Rpi-amr3, respectively. Protein extracts from *N. benthamiana nrc2/3/4 knockout* plants were immunoprecipitated with anti-Flag antibodies and loaded on blue native-PAGE.

Data information: Ponceau S staining serve as loading control in panel (A). Molecular weight markers are shown on the right. Experiments were done with at least three biological replicates with similar results.
Source data are available online for this figure.

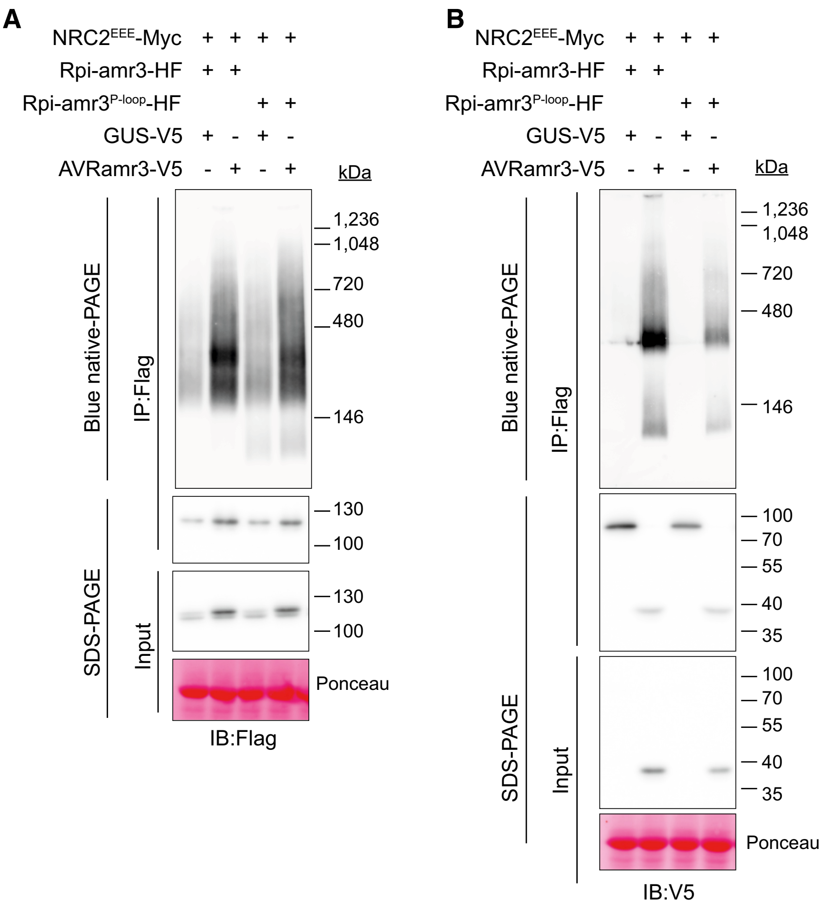

Figure EV4. Rpi-amr3 P-loop mutant interacts with and forms stable complex with AVRamr3.

A P-loop of Rpi-amr3 is dispensable for association with AVRamr3. Protein extracts from *N. benthamiana nrc2/3/4 knockout* plants were immunoprecipitated with anti-Flag antibody and blue native-PAGE was performed. Membranes were immunoblotted with anti-Flag.

B AVRamr3 associates with both wild-type Rpi-amr3 and Rpi-amr3$^{P\text{-}loop}$. Protein extracts from *N. benthamiana nrc2/3/4 knockout* plants were immunoprecipitated with anti-Flag antibody and blue native-PAGE was performed. Membranes were immunoblotted with anti-V5.

Data information: Ponceau S staining serves as loading control for panels (A and B). Molecular markers are shown on the right. Similar results were observed in at least three biological replicates.
Source data are available online for this figure.

© 2023 The Authors

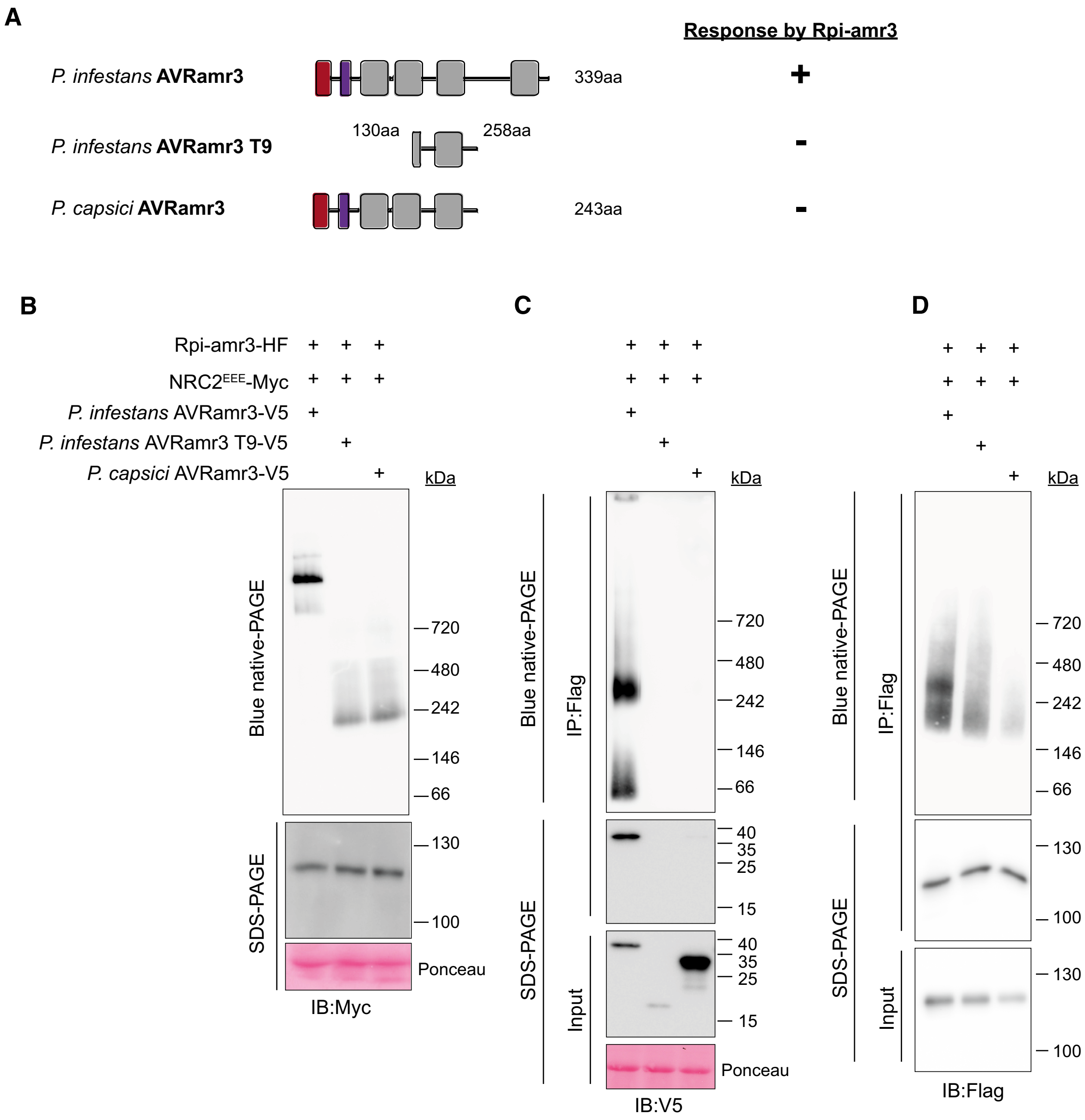

Figure EV5. Non-recognized alleles of AVRamr3 do not trigger NRC2 oligomerization and do not interact with Rpi-amr3.

A Schematic depiction of AVRamr3 from *Phytophthora infestans*, an unrecognized, truncated version of AVRamr3 (AVRamr3 T9), and AVRamr3 from *P. capsici*. Response of the corresponding alleles by Rpi-amr3, and thus occurrence of HR is indicated as + (recognition) or − (no recognition).

B $NRC2^{EEE}$-Myc oligomerizes in recognition-dependent manner. Protein extracts from *N. benthamiana nrc2/3/4 knockout* were loaded on blue native-PAGE, and blotted for Myc.

C Recognition is correlated with interaction and protein complex formation of AVRamr3 with Rpi-amr3. Protein extracts from (B) were immunoprecipitated with anti-Flag antibody and were loaded on blue native-PAGE and blotted for V5.

D Recognition is correlated with interaction and protein complex formation of Rpi-amr3 with AVRamr3. Protein extracts from (B) were immunoprecipitated with anti-Flag antibody and were loaded on blue native-PAGE and blotted for Flag.

Data information: SDS-boiled input and IP eluates were loaded onto SDS–PAGE as control. Ponceau S staining serves as loading control in panels (B and C). Molecular markers are indicated on the right. Experiments were done with at least three biological replicates with similar results. Ponceau S loading for (D) was same as with (C). Source data are available online for this figure.

© 2023 The Authors
