# Supplementary figures and images for: Effector‐dependent activation and oligomerization of plant NRC class helper NLRs by sensor NLR immune receptors Rpi‐amr3 and Rpi‐amr1 (part 1 of 2)
Source: EMBO J. 2023 Jan 2;42(5):e111484. doi: 10.15252/embj.2022111484 (PMC9975942; doi:10.15252/embj.2022111484)

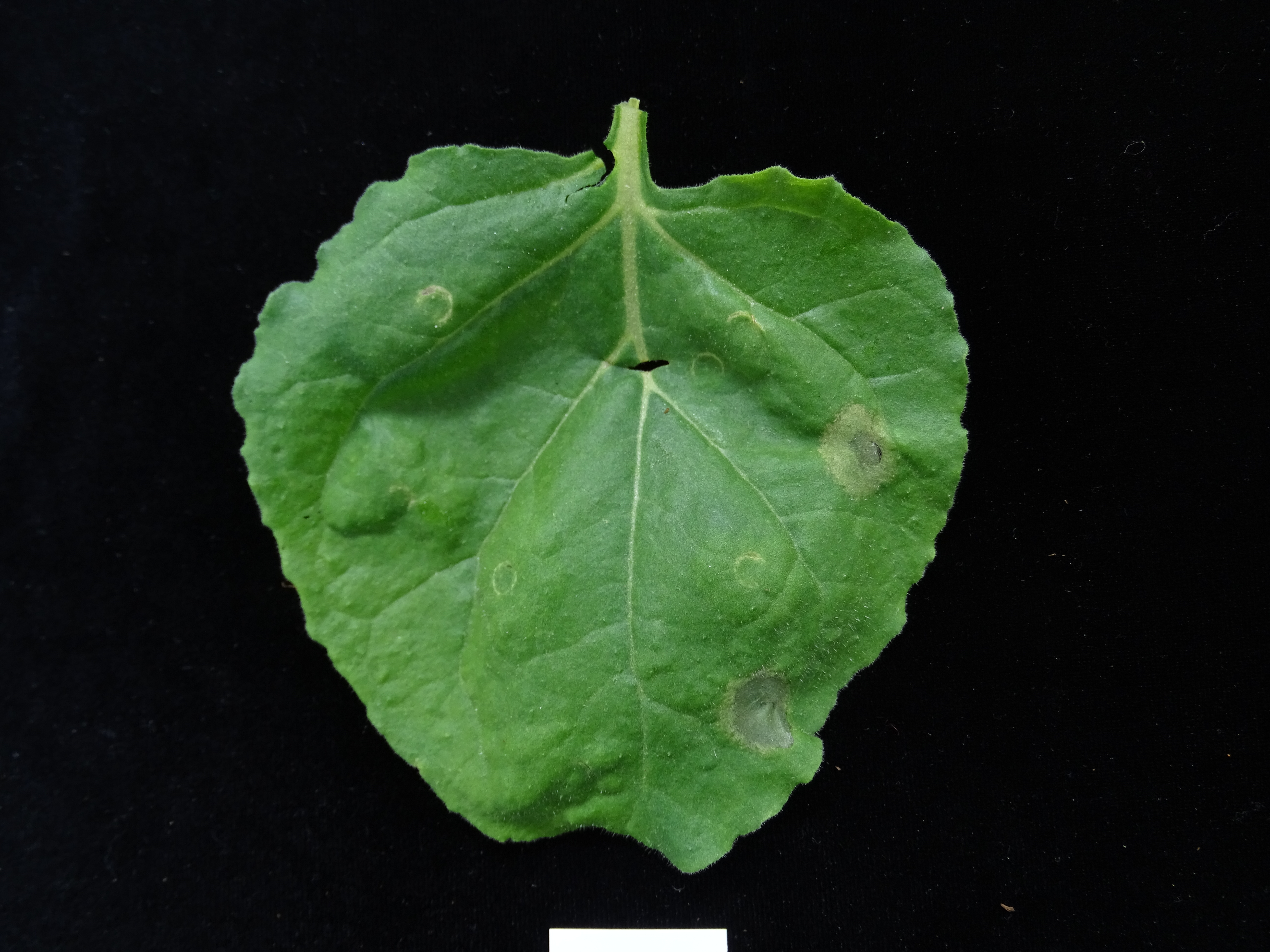

Supplement: Supplementary file 4 — Source Data for Expanded View [file EMBJ-42-e111484-s002.zip › EMBOJ-2022-11484_SourceData/Figure EV1/EV1E/DSC05573.JPG]

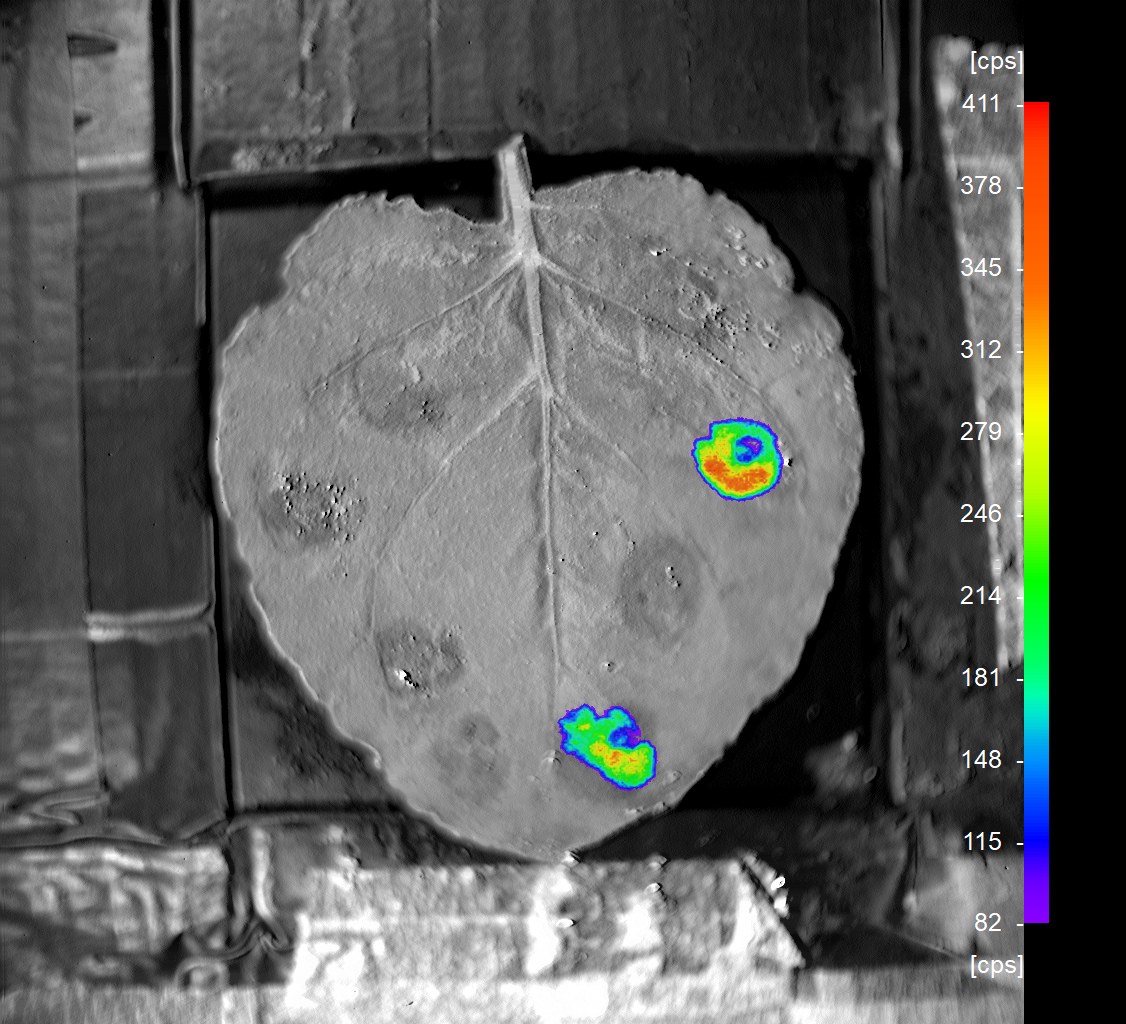

Supplement: Supplementary file 4 — Source Data for Expanded View [file EMBJ-42-e111484-s002.zip › EMBOJ-2022-11484_SourceData/Figure EV1/EV1F/Id13_2min_Rep1_leave2_Rpi1and3-Avr1and3_3dpi.png]

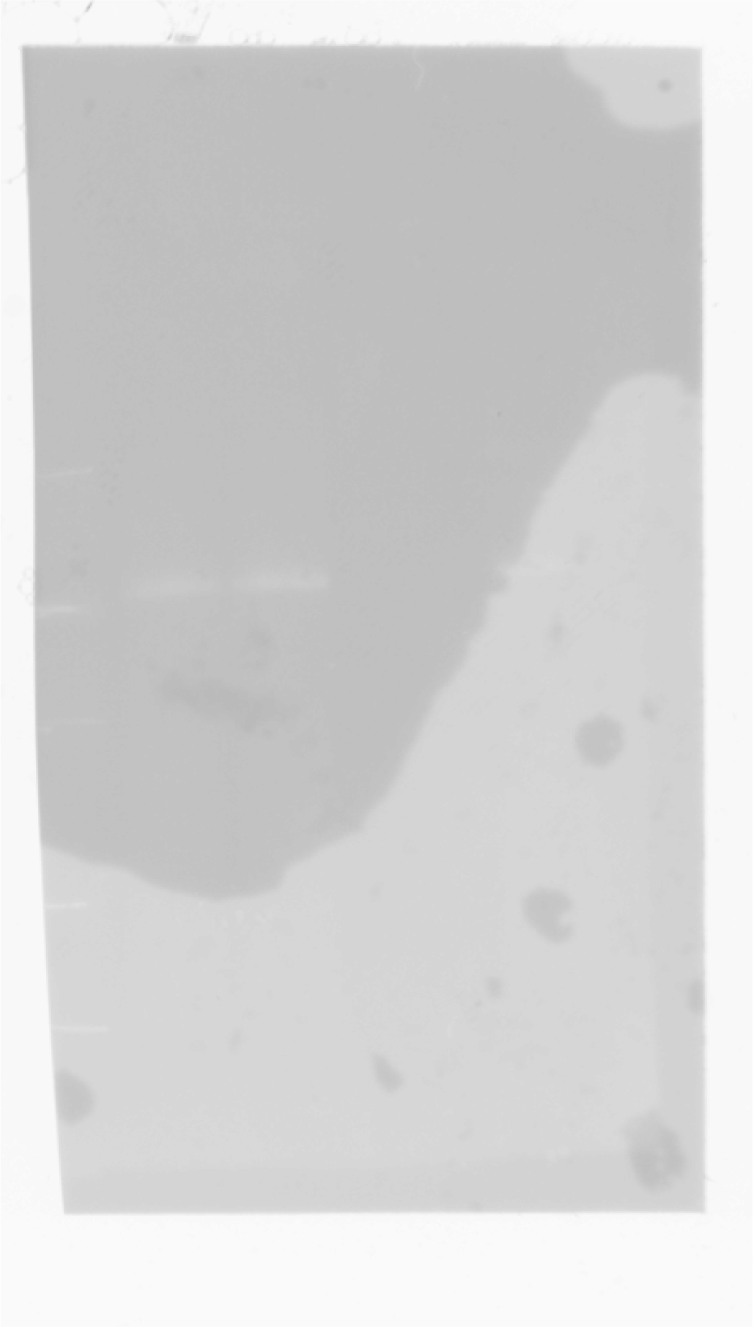

Supplement: Supplementary file 4 — Source Data for Expanded View [file EMBJ-42-e111484-s002.zip › EMBOJ-2022-11484_SourceData/Figure EV2/EV2A/BNP Western Myc Marker.tif]

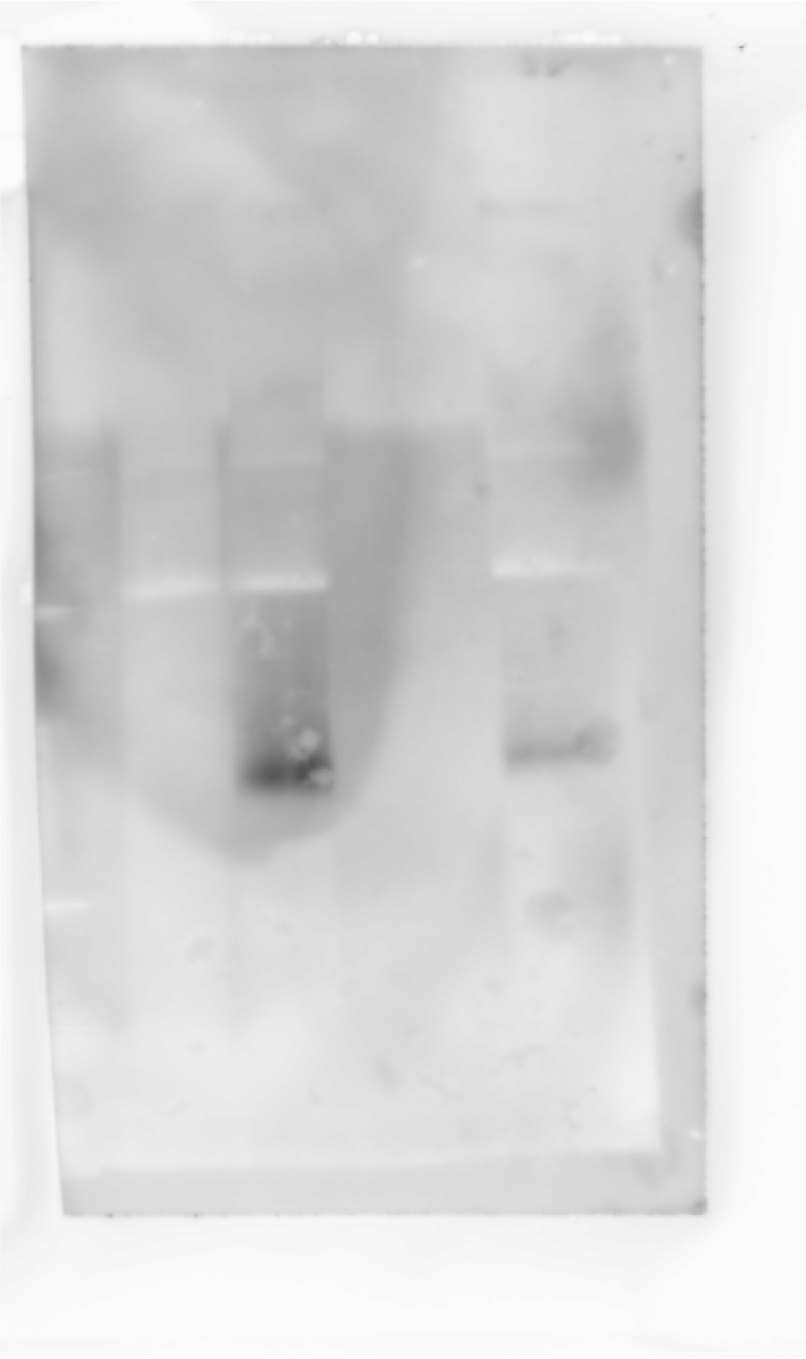

Supplement: Supplementary file 4 — Source Data for Expanded View [file EMBJ-42-e111484-s002.zip › EMBOJ-2022-11484_SourceData/Figure EV2/EV2A/BNP Western Myc.tif]

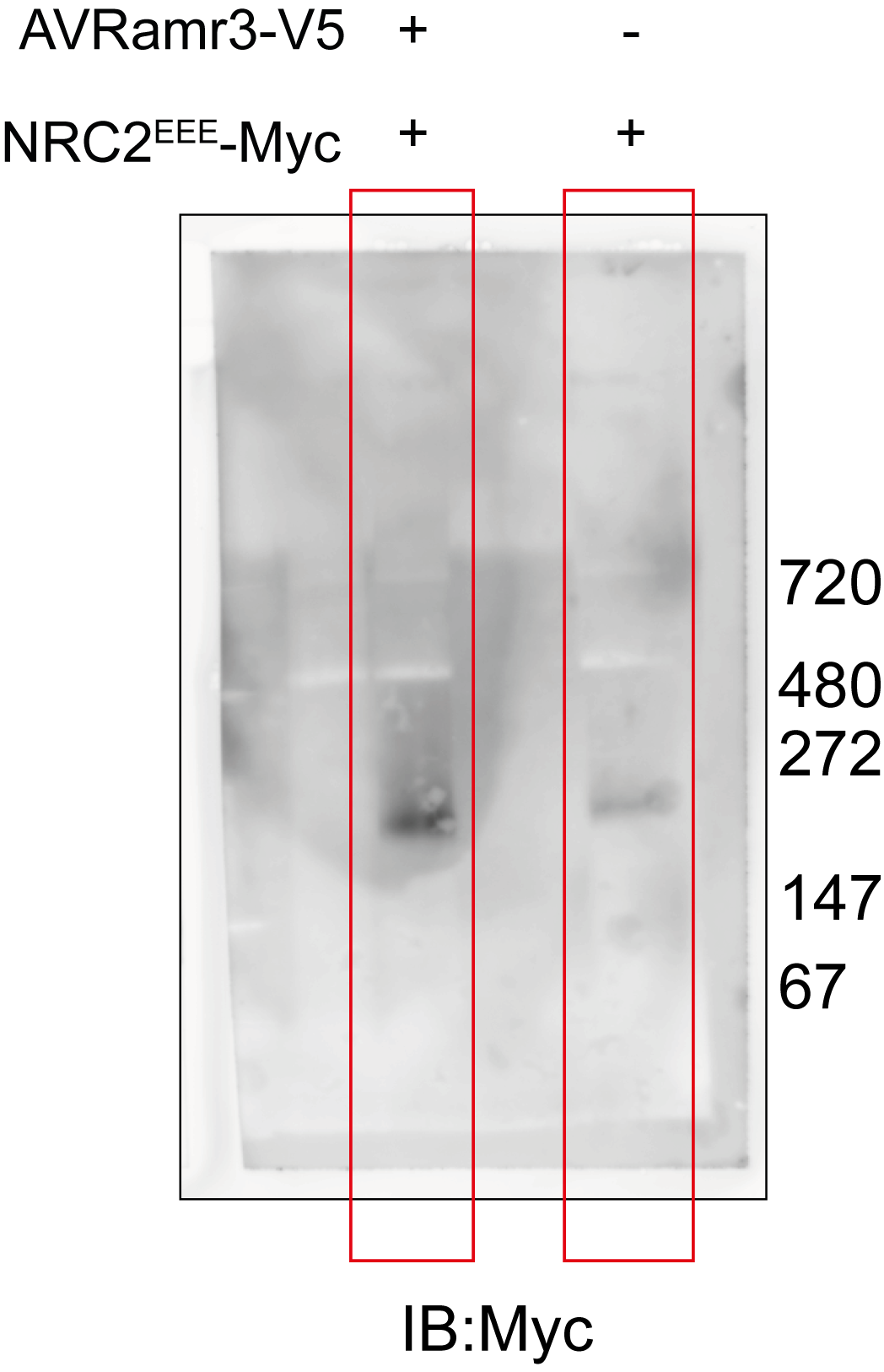

Supplement: Supplementary file 4 — Source Data for Expanded View [file EMBJ-42-e111484-s002.zip › EMBOJ-2022-11484_SourceData/Figure EV2/EV2A/BNP Western Myc_annotations.tif]

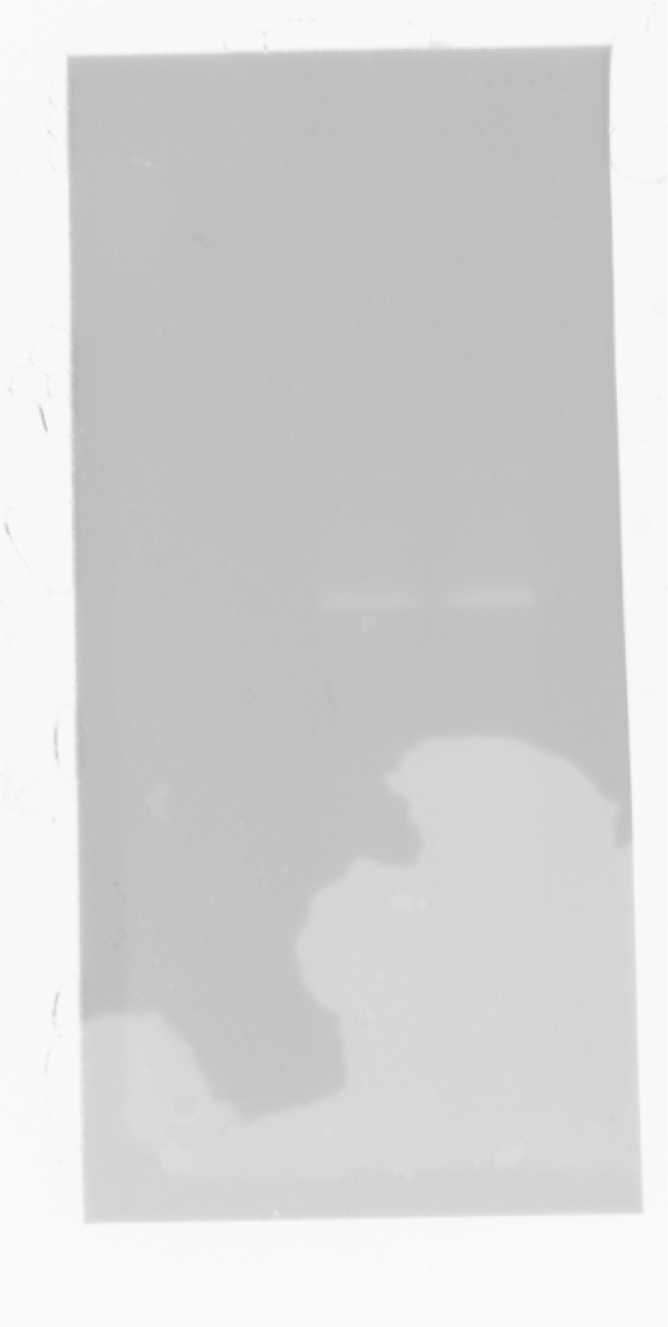

Supplement: Supplementary file 4 — Source Data for Expanded View [file EMBJ-42-e111484-s002.zip › EMBOJ-2022-11484_SourceData/Figure EV2/EV2A/BNP Western V5 Marker.tif]

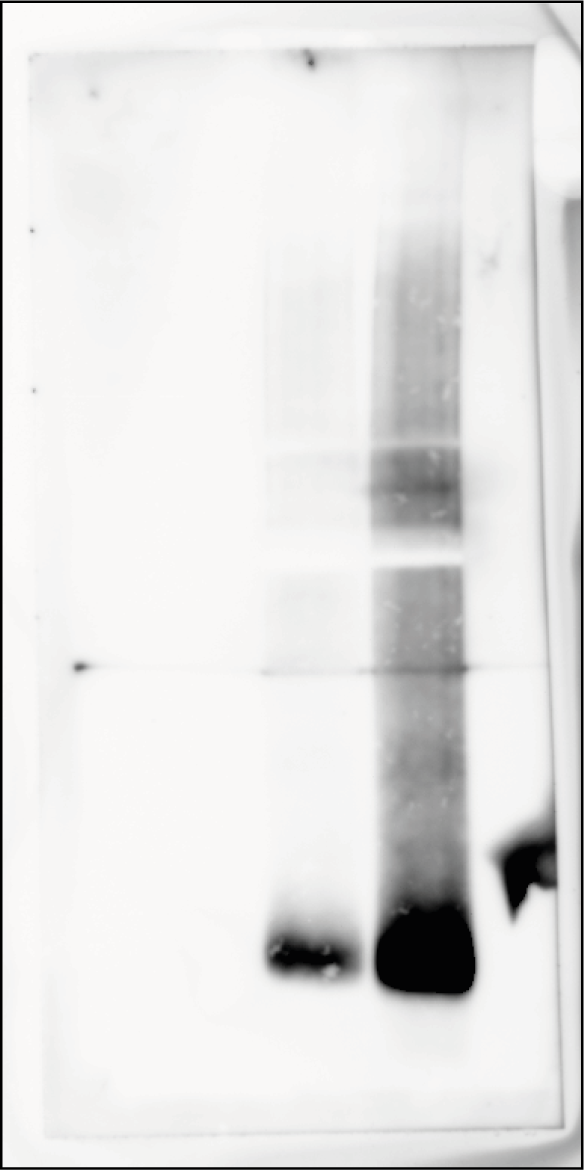

Supplement: Supplementary file 4 — Source Data for Expanded View [file EMBJ-42-e111484-s002.zip › EMBOJ-2022-11484_SourceData/Figure EV2/EV2A/BNP Western V5.tif]

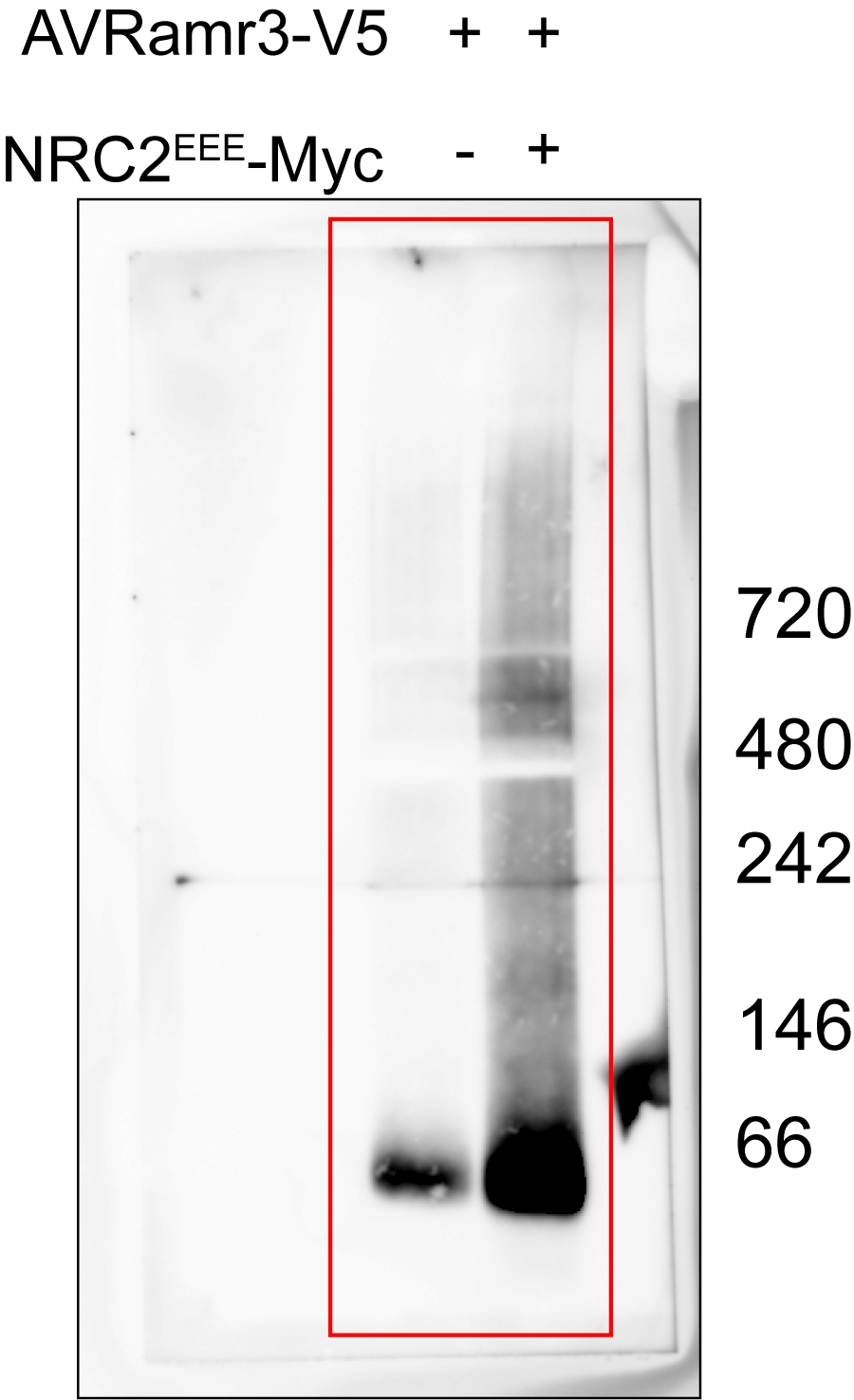

Supplement: Supplementary file 4 — Source Data for Expanded View [file EMBJ-42-e111484-s002.zip › EMBOJ-2022-11484_SourceData/Figure EV2/EV2A/BNP Western V5_annotations.tif]

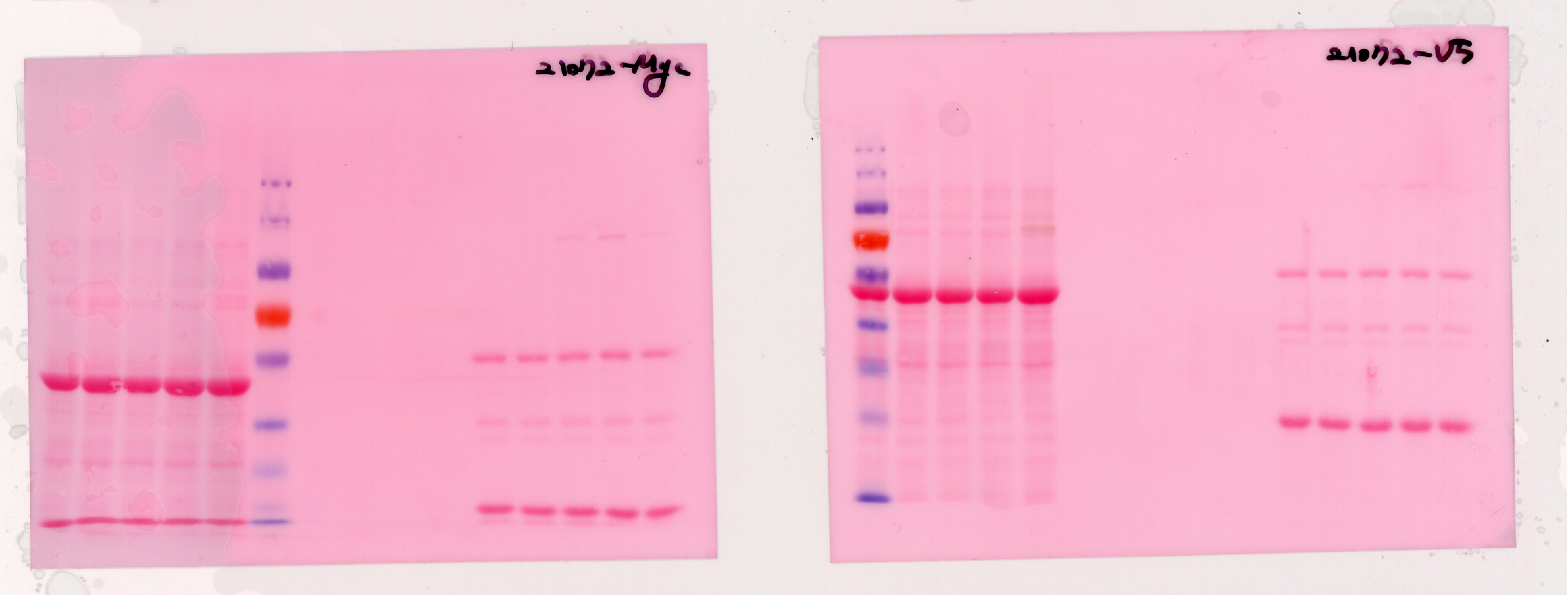

Supplement: Supplementary file 4 — Source Data for Expanded View [file EMBJ-42-e111484-s002.zip › EMBOJ-2022-11484_SourceData/Figure EV2/EV2A/SDS Ponceau.tif]

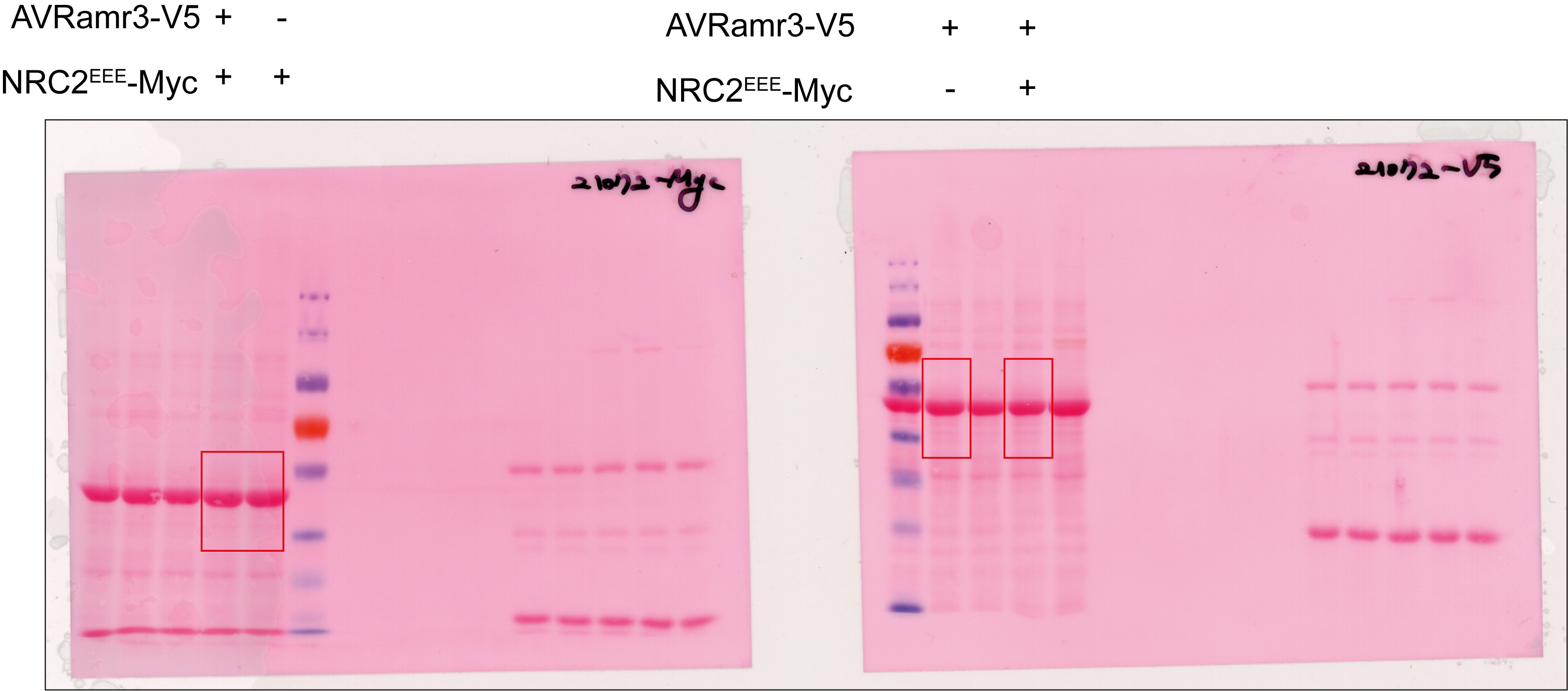

Supplement: Supplementary file 4 — Source Data for Expanded View [file EMBJ-42-e111484-s002.zip › EMBOJ-2022-11484_SourceData/Figure EV2/EV2A/SDS Ponceau_annotations.tif]

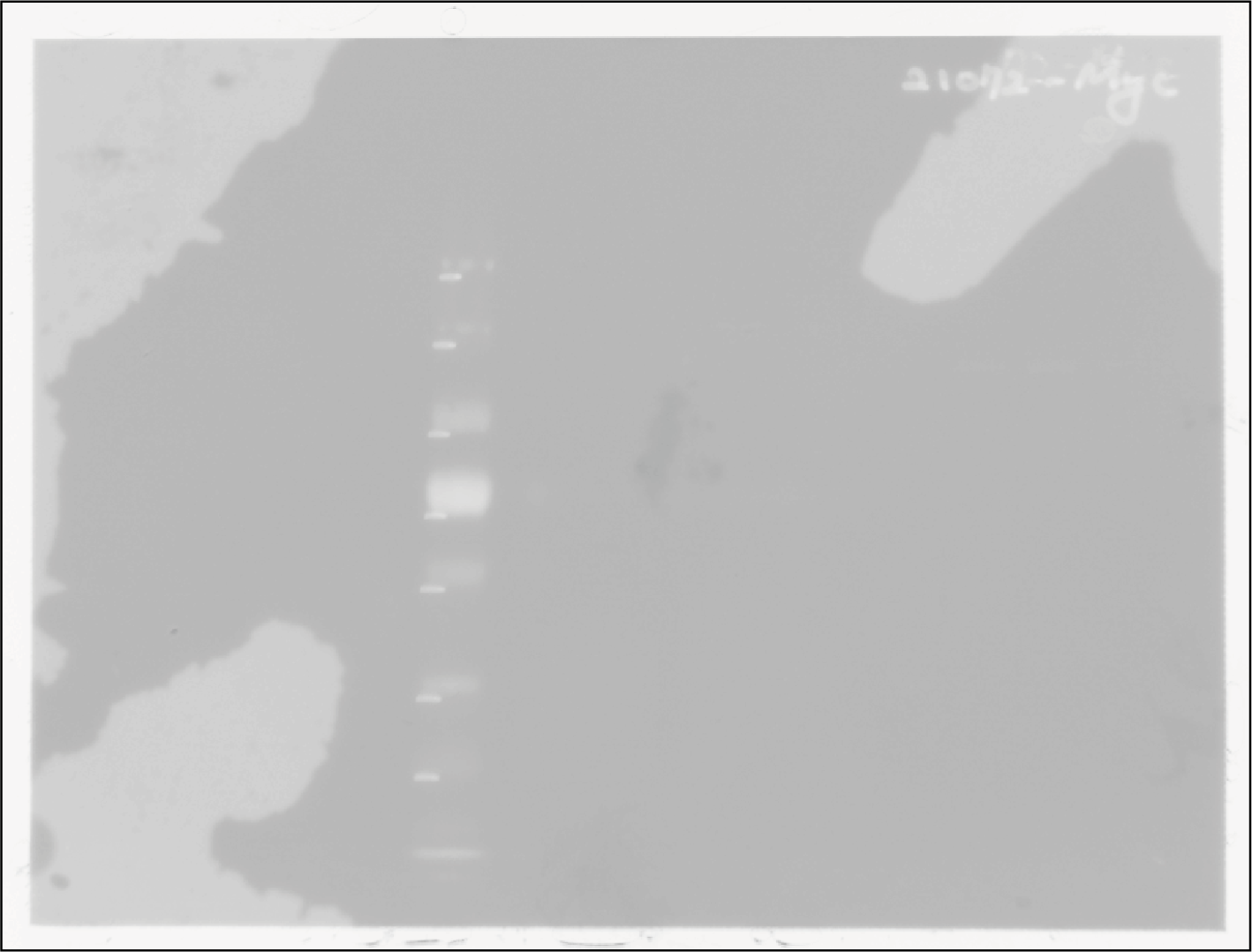

Supplement: Supplementary file 4 — Source Data for Expanded View [file EMBJ-42-e111484-s002.zip › EMBOJ-2022-11484_SourceData/Figure EV2/EV2A/SDS Western Myc Marker.tif]

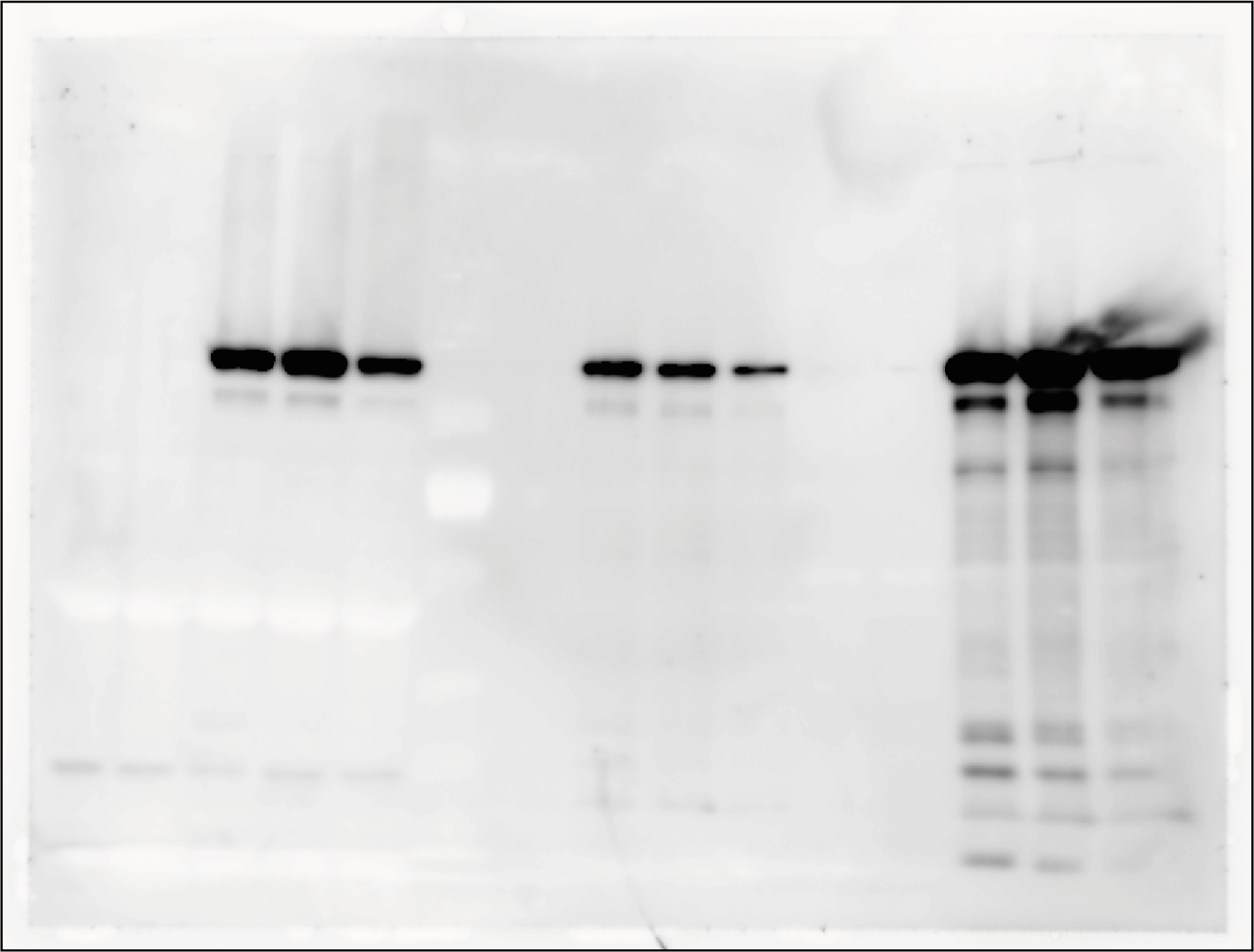

Supplement: Supplementary file 4 — Source Data for Expanded View [file EMBJ-42-e111484-s002.zip › EMBOJ-2022-11484_SourceData/Figure EV2/EV2A/SDS Western Myc.tif]

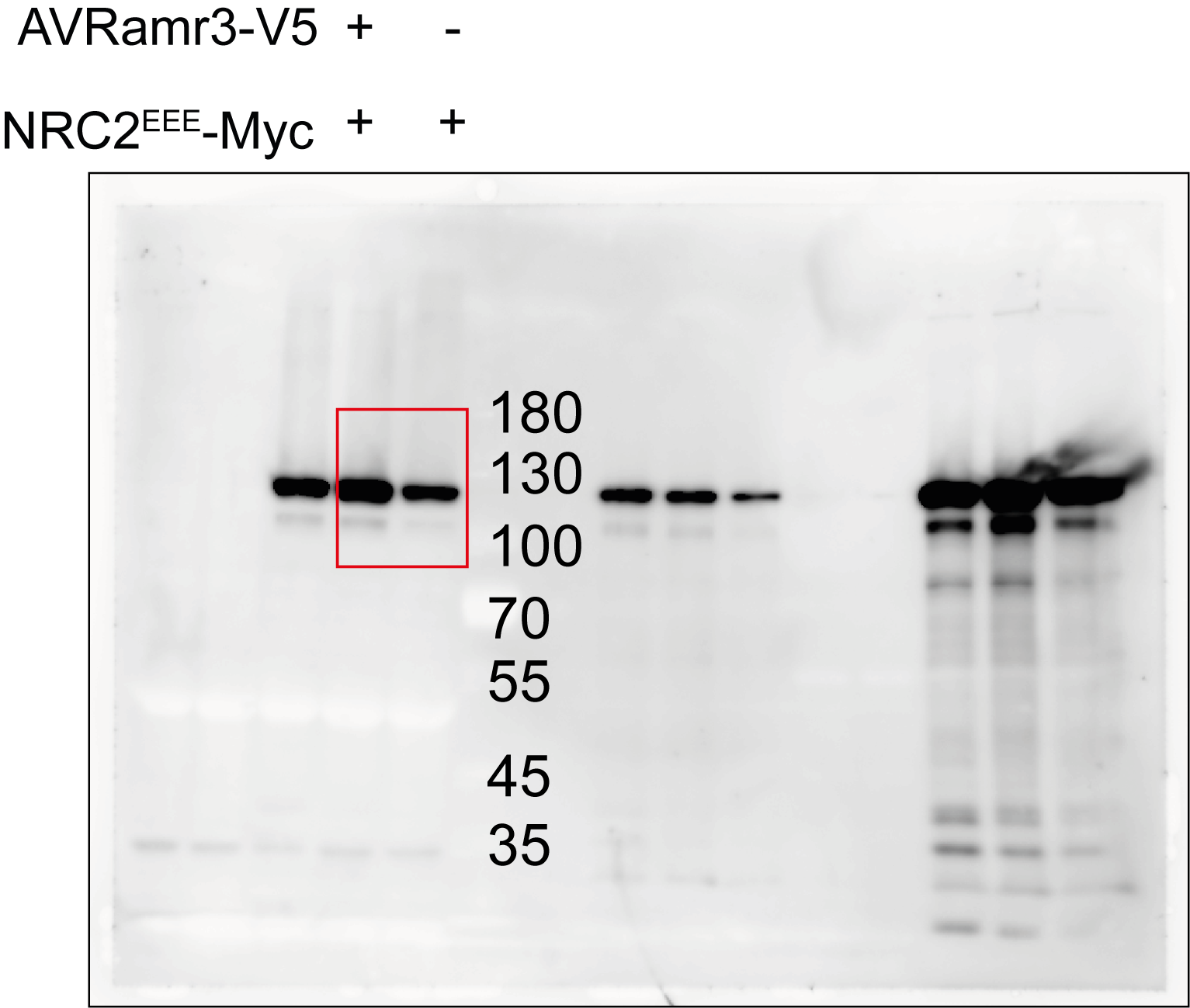

Supplement: Supplementary file 4 — Source Data for Expanded View [file EMBJ-42-e111484-s002.zip › EMBOJ-2022-11484_SourceData/Figure EV2/EV2A/SDS Western Myc_annotations.tif]

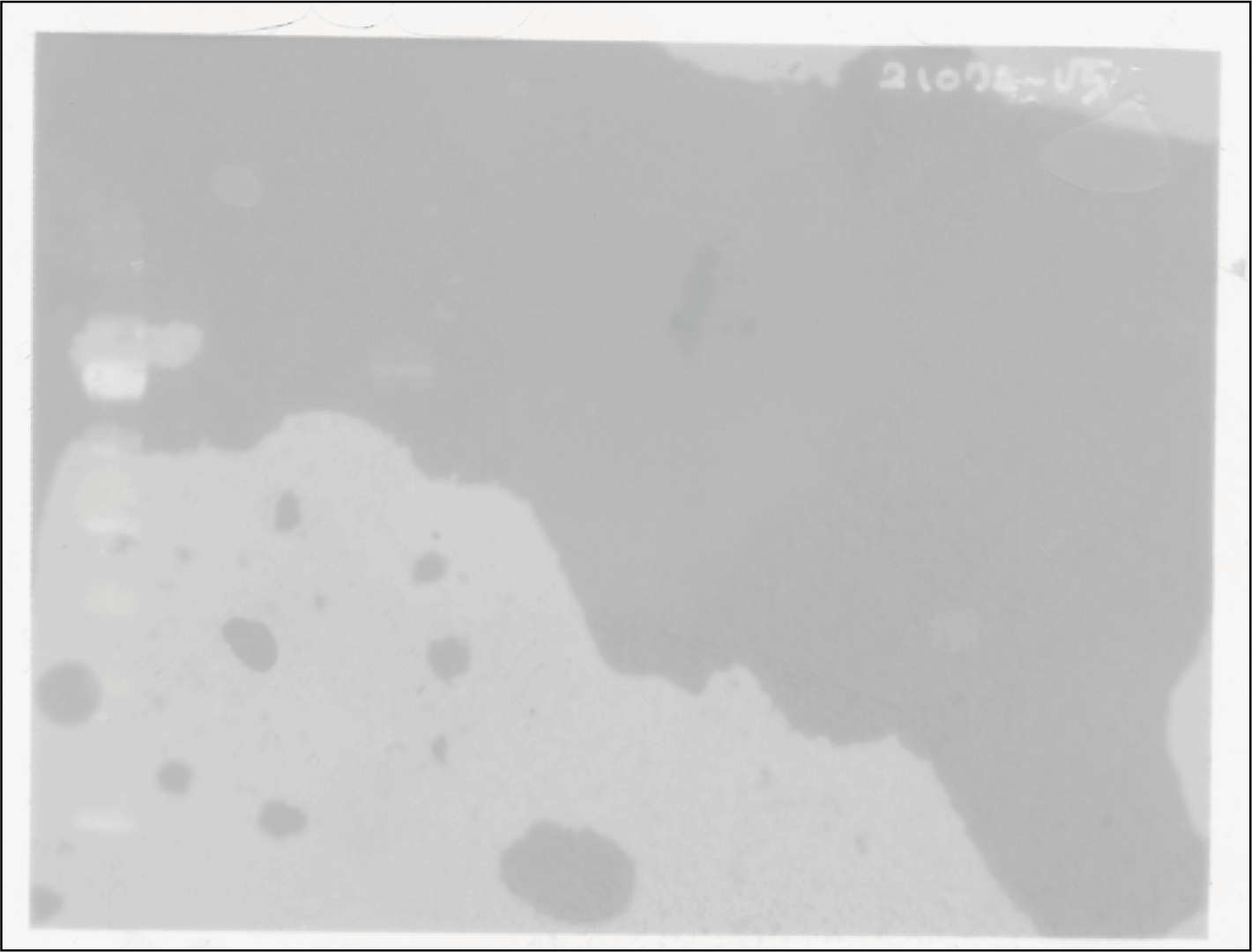

Supplement: Supplementary file 4 — Source Data for Expanded View [file EMBJ-42-e111484-s002.zip › EMBOJ-2022-11484_SourceData/Figure EV2/EV2A/SDS Western V5 Marker.tif]

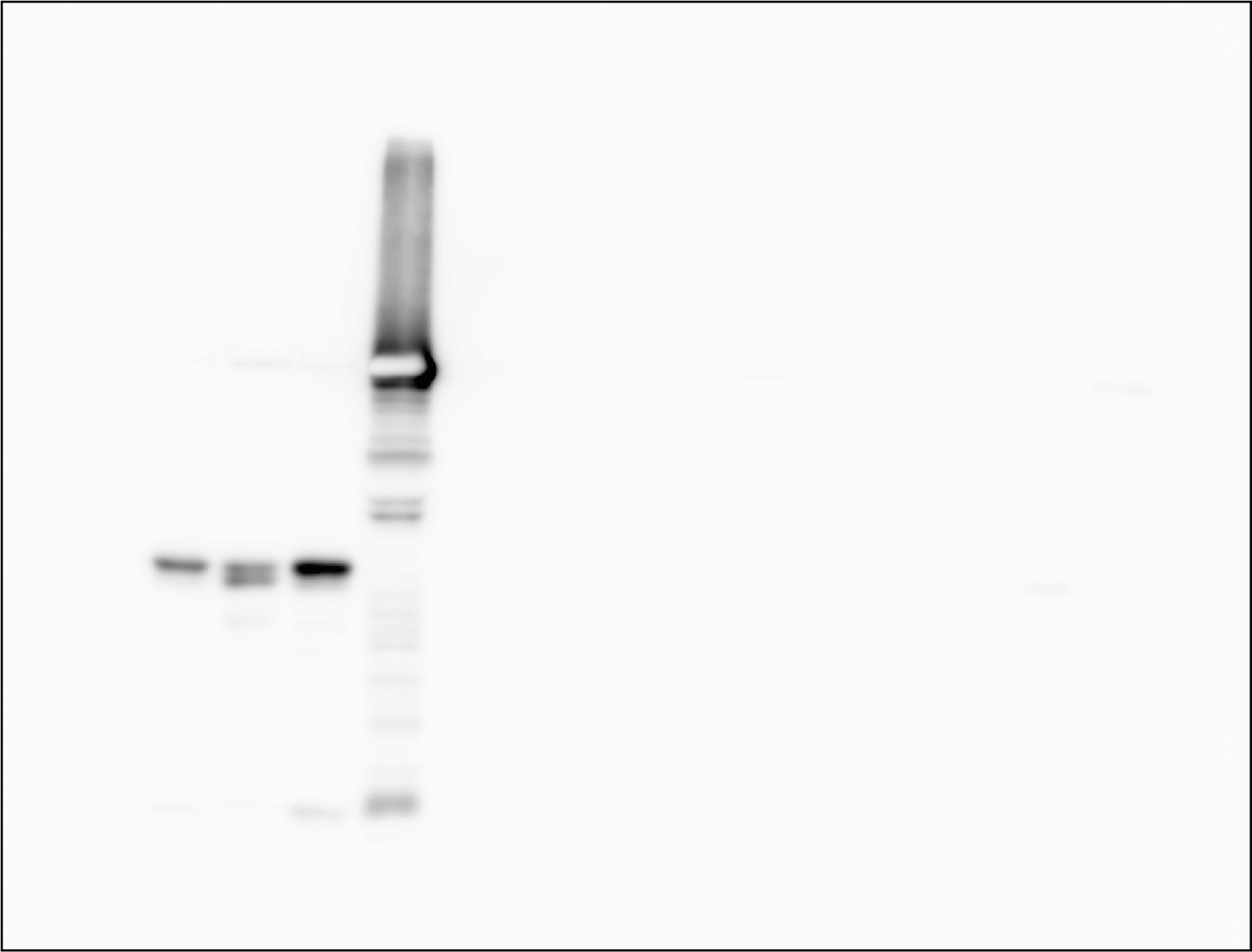

Supplement: Supplementary file 4 — Source Data for Expanded View [file EMBJ-42-e111484-s002.zip › EMBOJ-2022-11484_SourceData/Figure EV2/EV2A/SDS Western V5.tif]

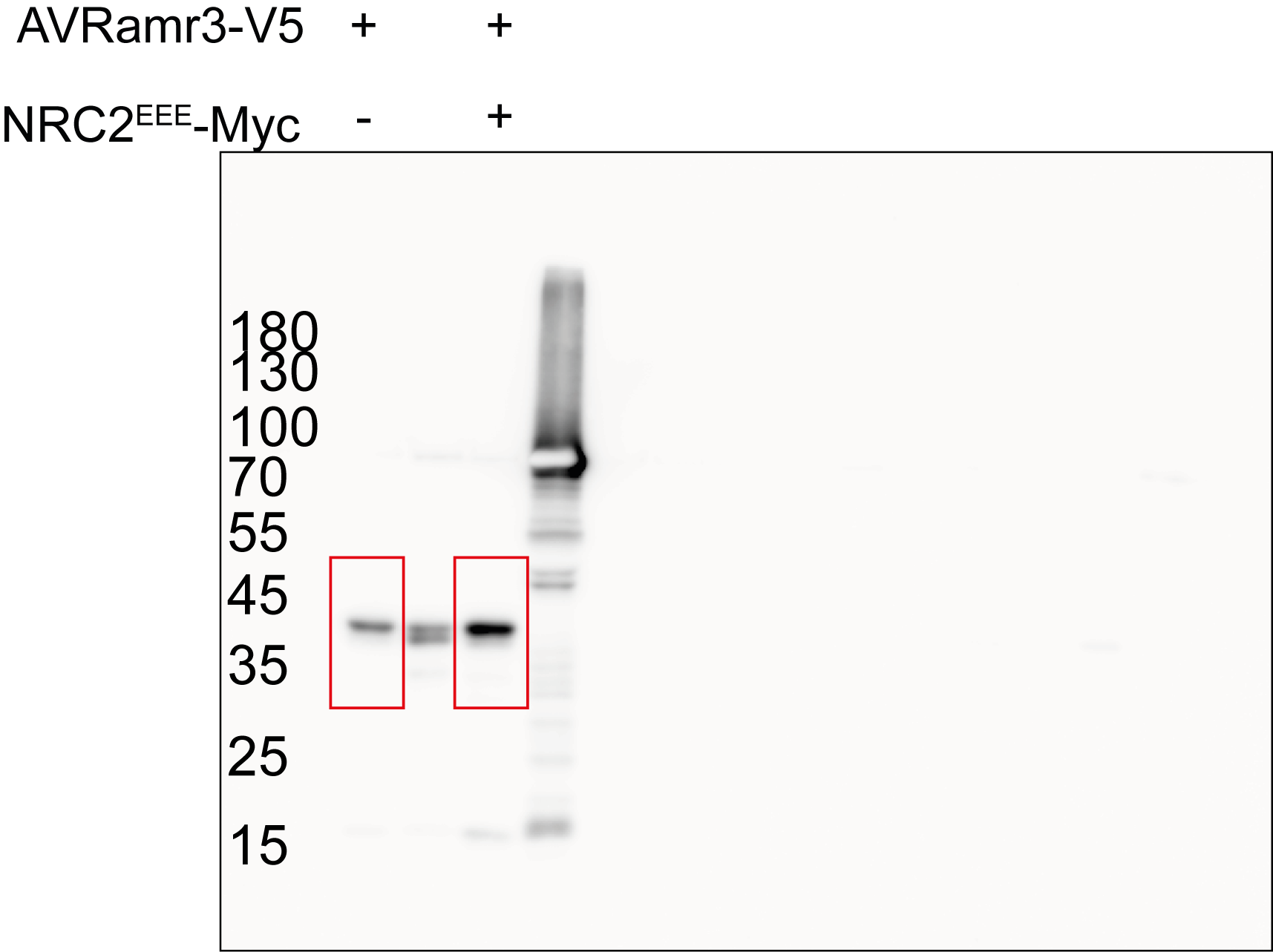

Supplement: Supplementary file 4 — Source Data for Expanded View [file EMBJ-42-e111484-s002.zip › EMBOJ-2022-11484_SourceData/Figure EV2/EV2A/SDS Western V5_annotations.tif]

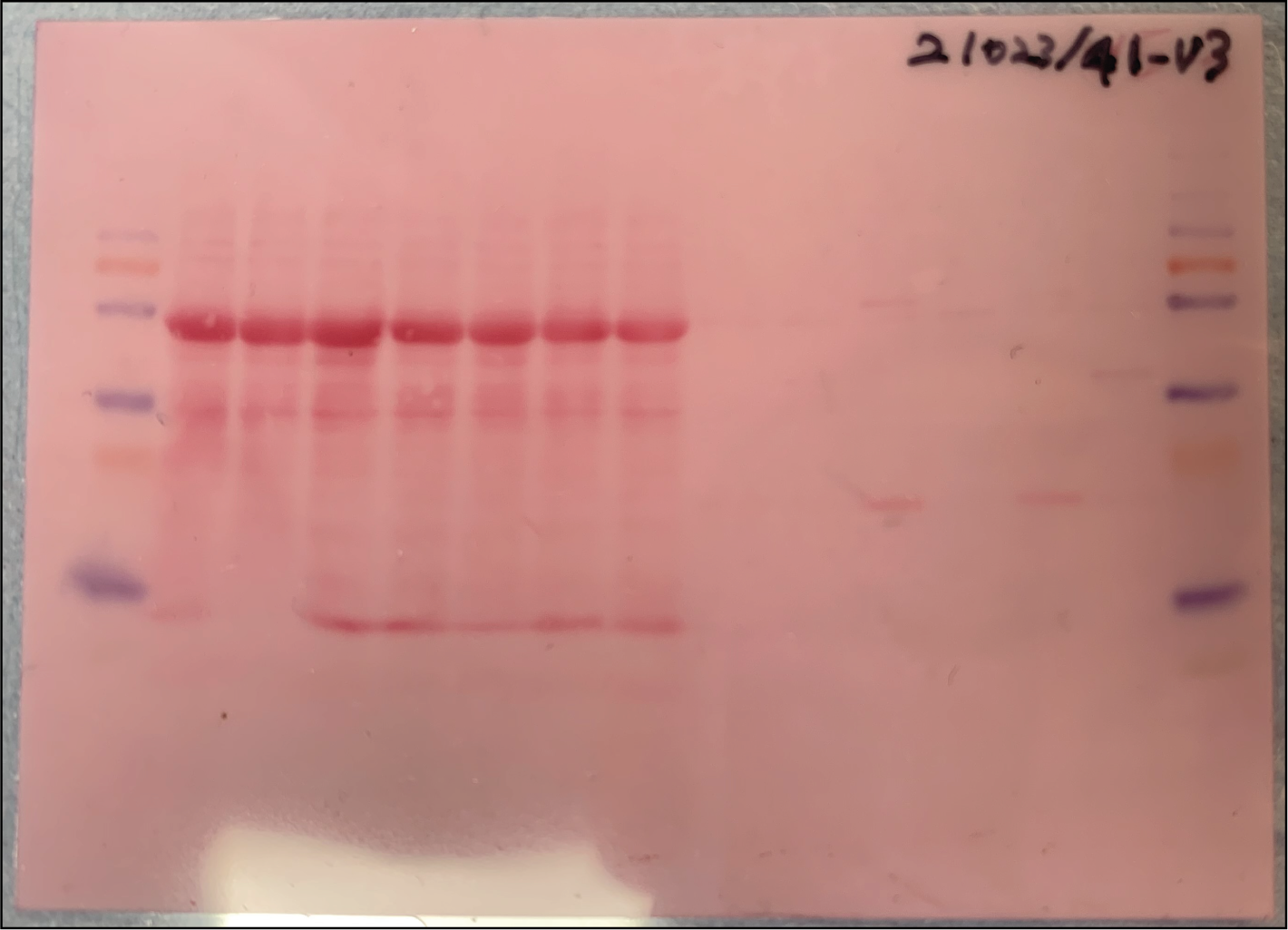

Supplement: Supplementary file 4 — Source Data for Expanded View [file EMBJ-42-e111484-s002.zip › EMBOJ-2022-11484_SourceData/Figure EV2/EV2B/SDS Ponceau.tif]

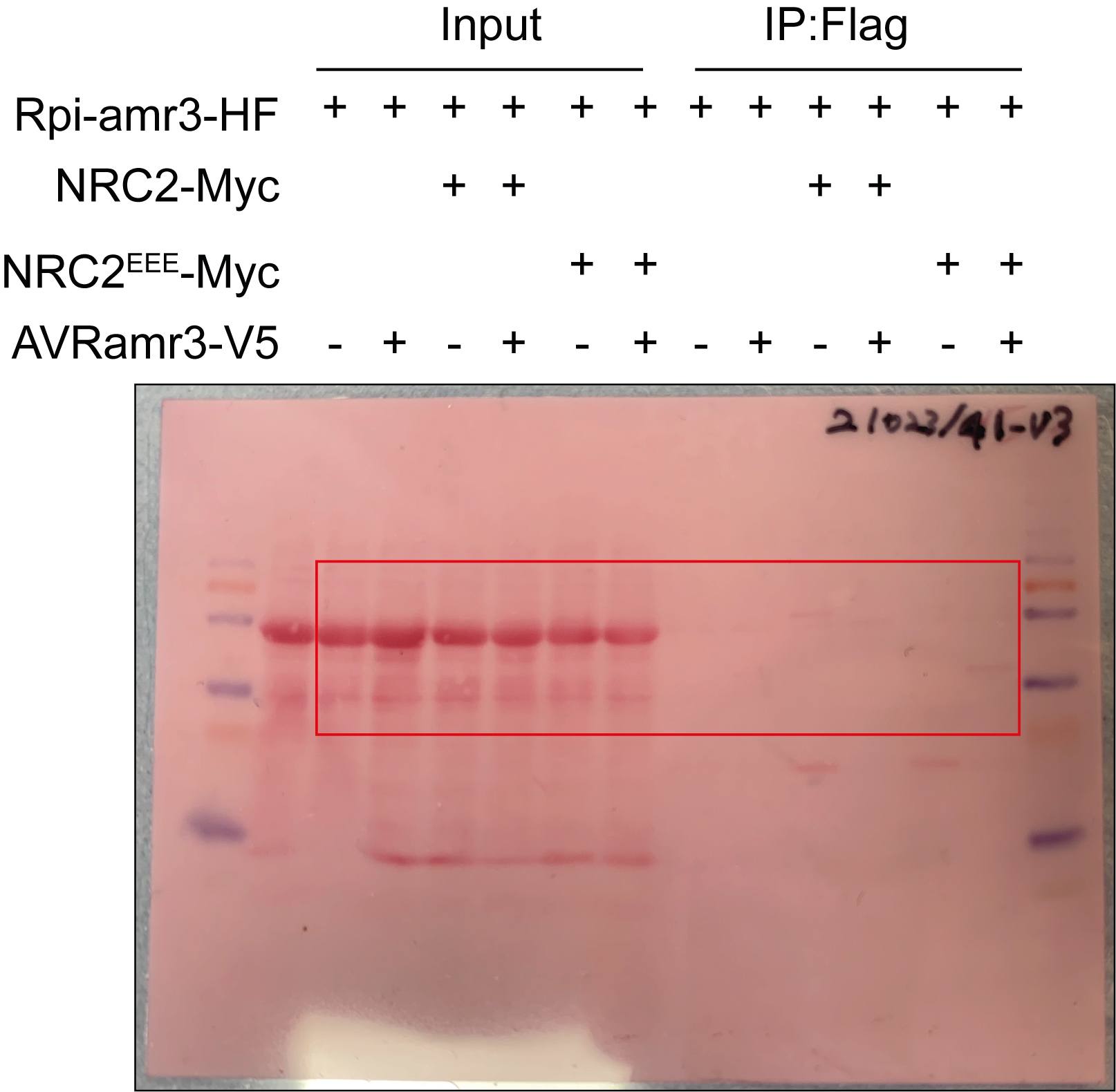

Supplement: Supplementary file 4 — Source Data for Expanded View [file EMBJ-42-e111484-s002.zip › EMBOJ-2022-11484_SourceData/Figure EV2/EV2B/SDS Ponceau_annotations.tif]

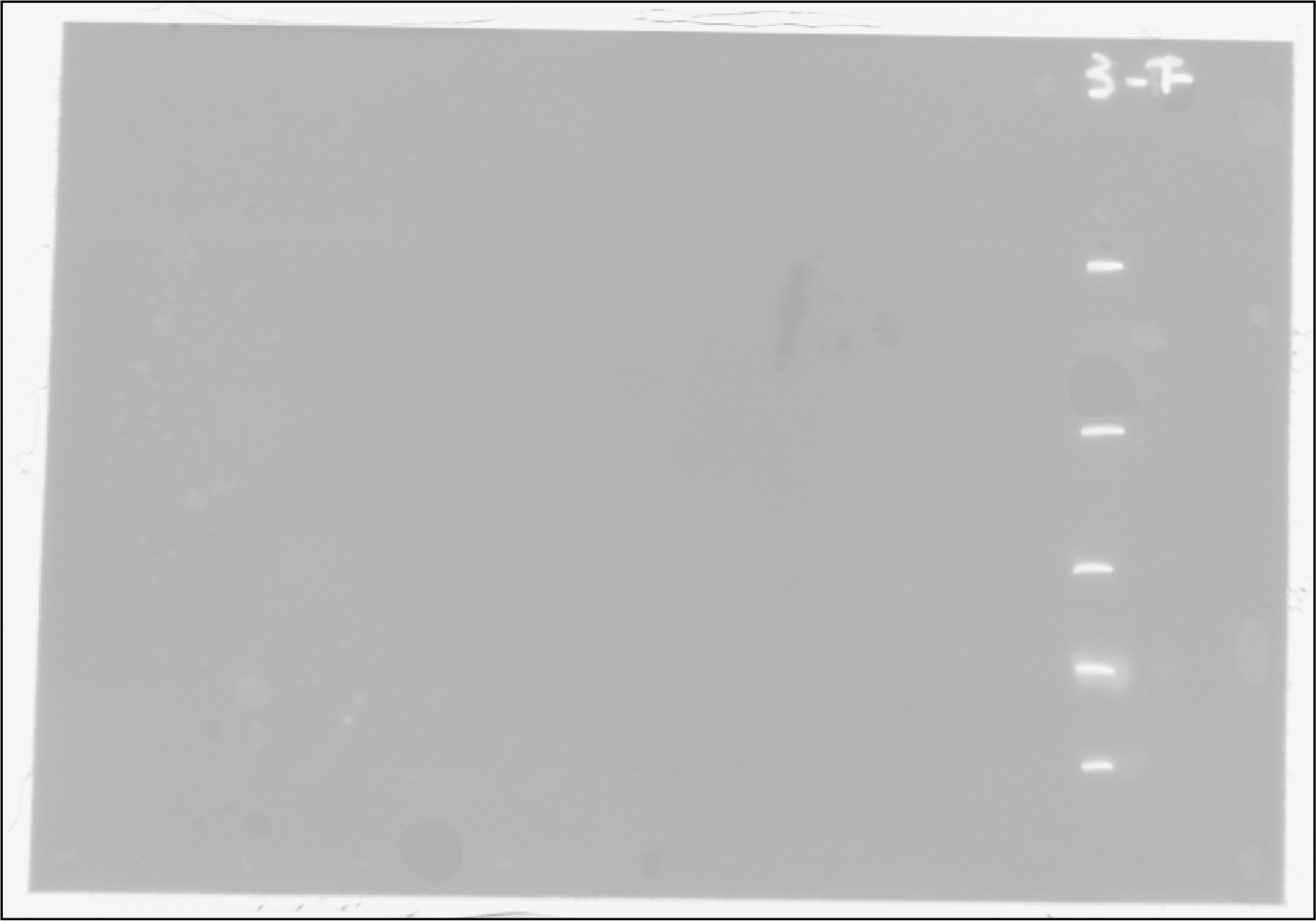

Supplement: Supplementary file 4 — Source Data for Expanded View [file EMBJ-42-e111484-s002.zip › EMBOJ-2022-11484_SourceData/Figure EV2/EV2B/Western Flag Marker.tif]

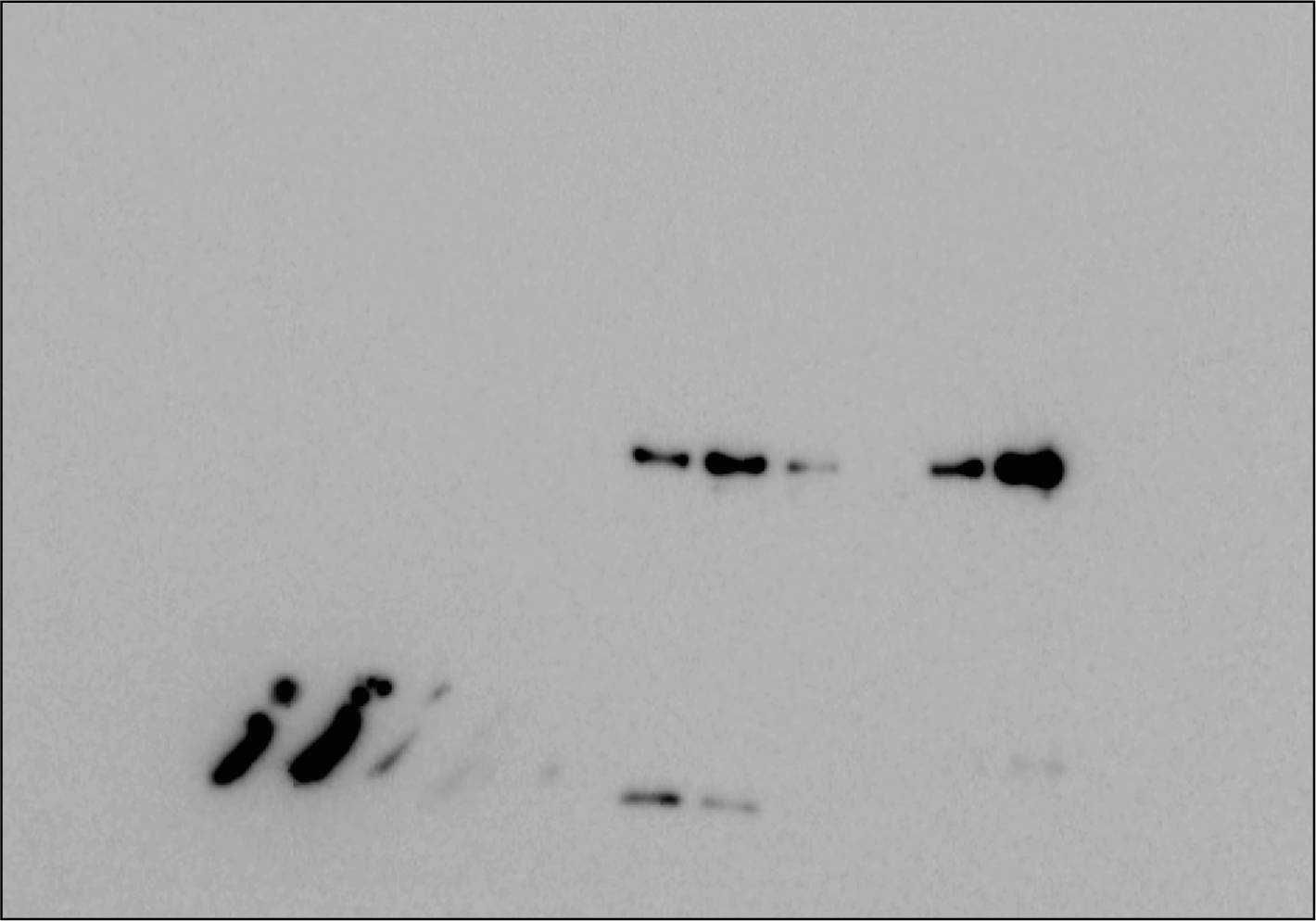

Supplement: Supplementary file 4 — Source Data for Expanded View [file EMBJ-42-e111484-s002.zip › EMBOJ-2022-11484_SourceData/Figure EV2/EV2B/Western Flag.tif]

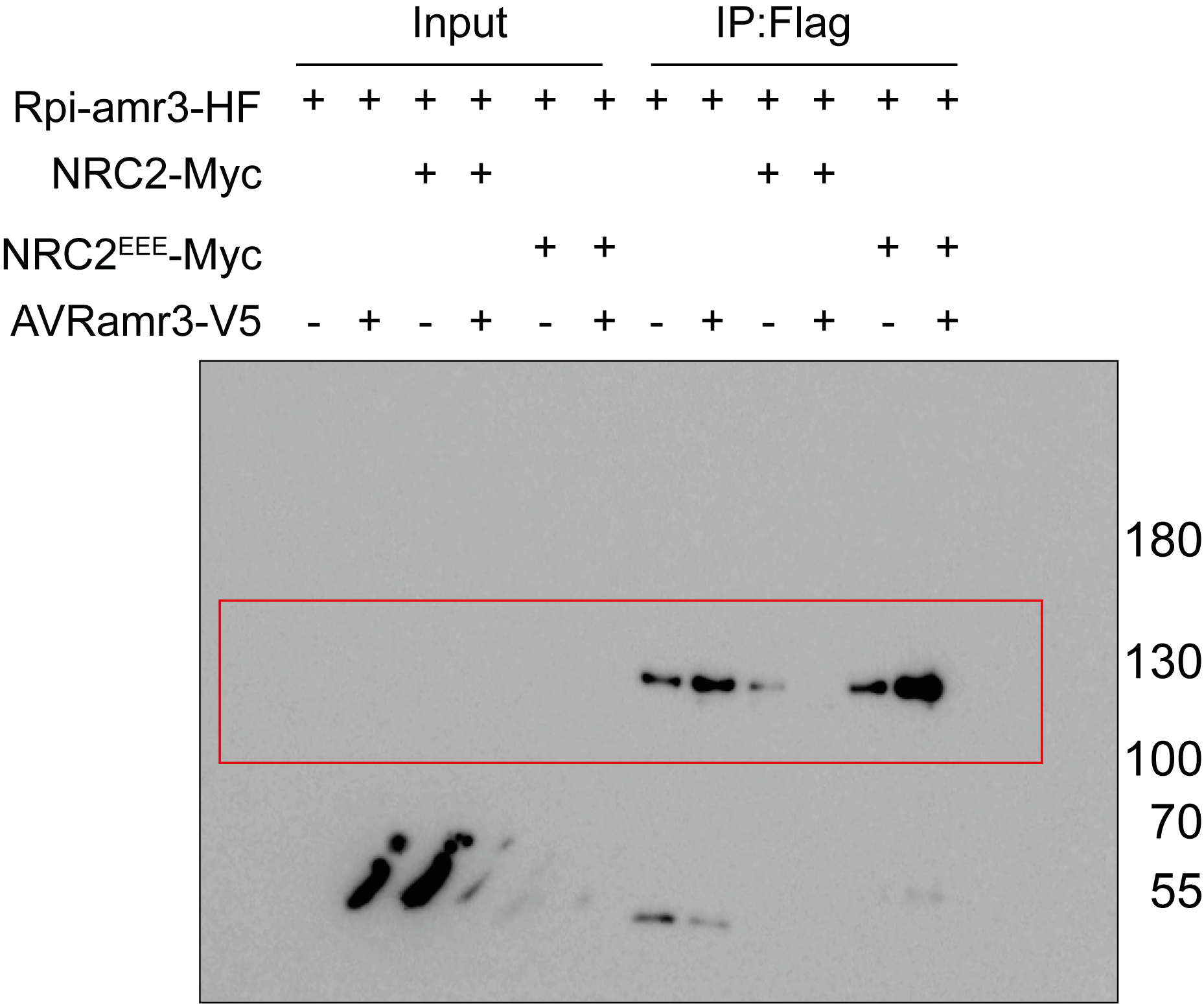

Supplement: Supplementary file 4 — Source Data for Expanded View [file EMBJ-42-e111484-s002.zip › EMBOJ-2022-11484_SourceData/Figure EV2/EV2B/Western Flag_annotation.tif]

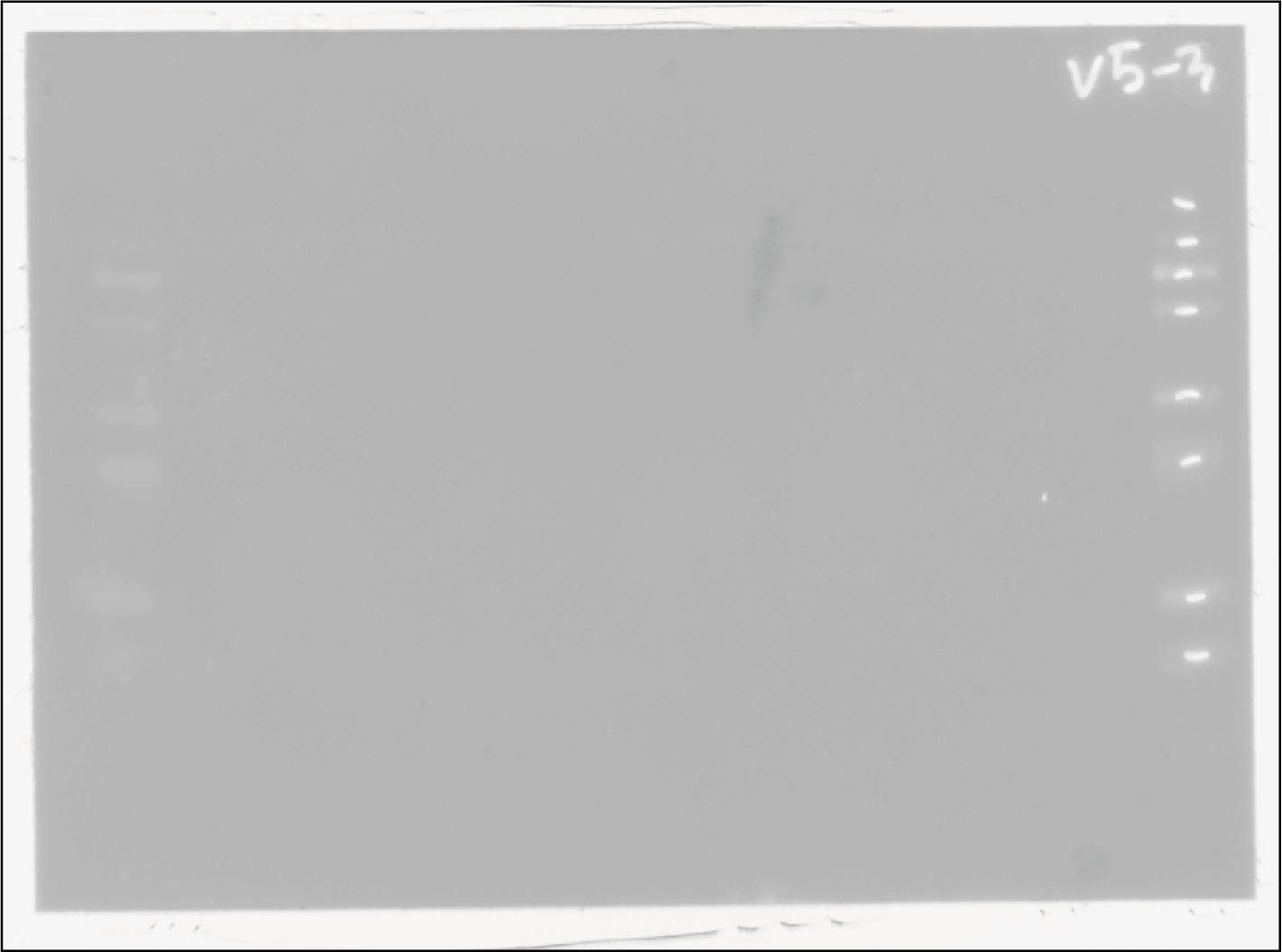

Supplement: Supplementary file 4 — Source Data for Expanded View [file EMBJ-42-e111484-s002.zip › EMBOJ-2022-11484_SourceData/Figure EV2/EV2B/Western V5 Marker.tif]

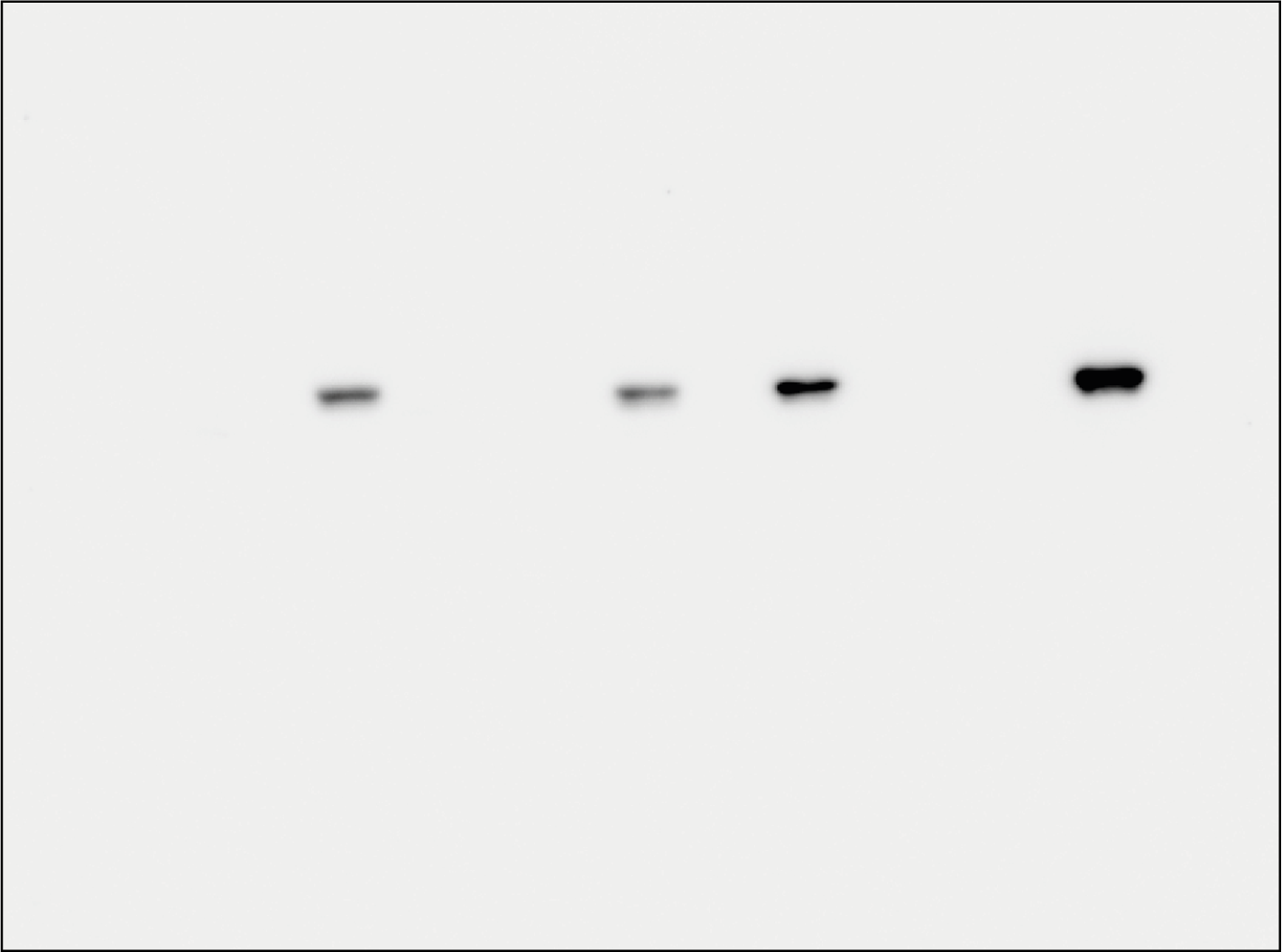

Supplement: Supplementary file 4 — Source Data for Expanded View [file EMBJ-42-e111484-s002.zip › EMBOJ-2022-11484_SourceData/Figure EV2/EV2B/Western V5.tif]

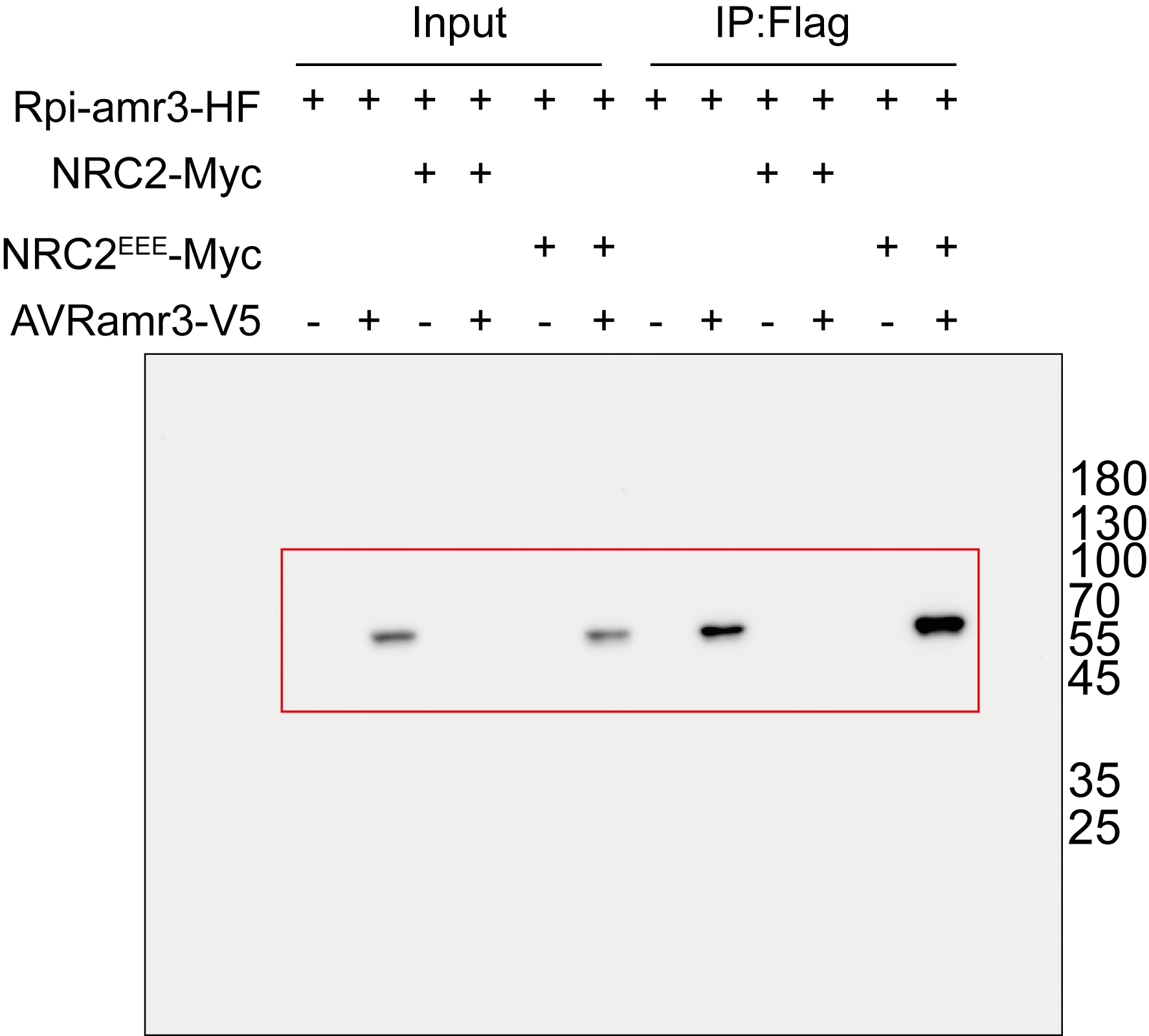

Supplement: Supplementary file 4 — Source Data for Expanded View [file EMBJ-42-e111484-s002.zip › EMBOJ-2022-11484_SourceData/Figure EV2/EV2B/Western V5_annotation.tif]

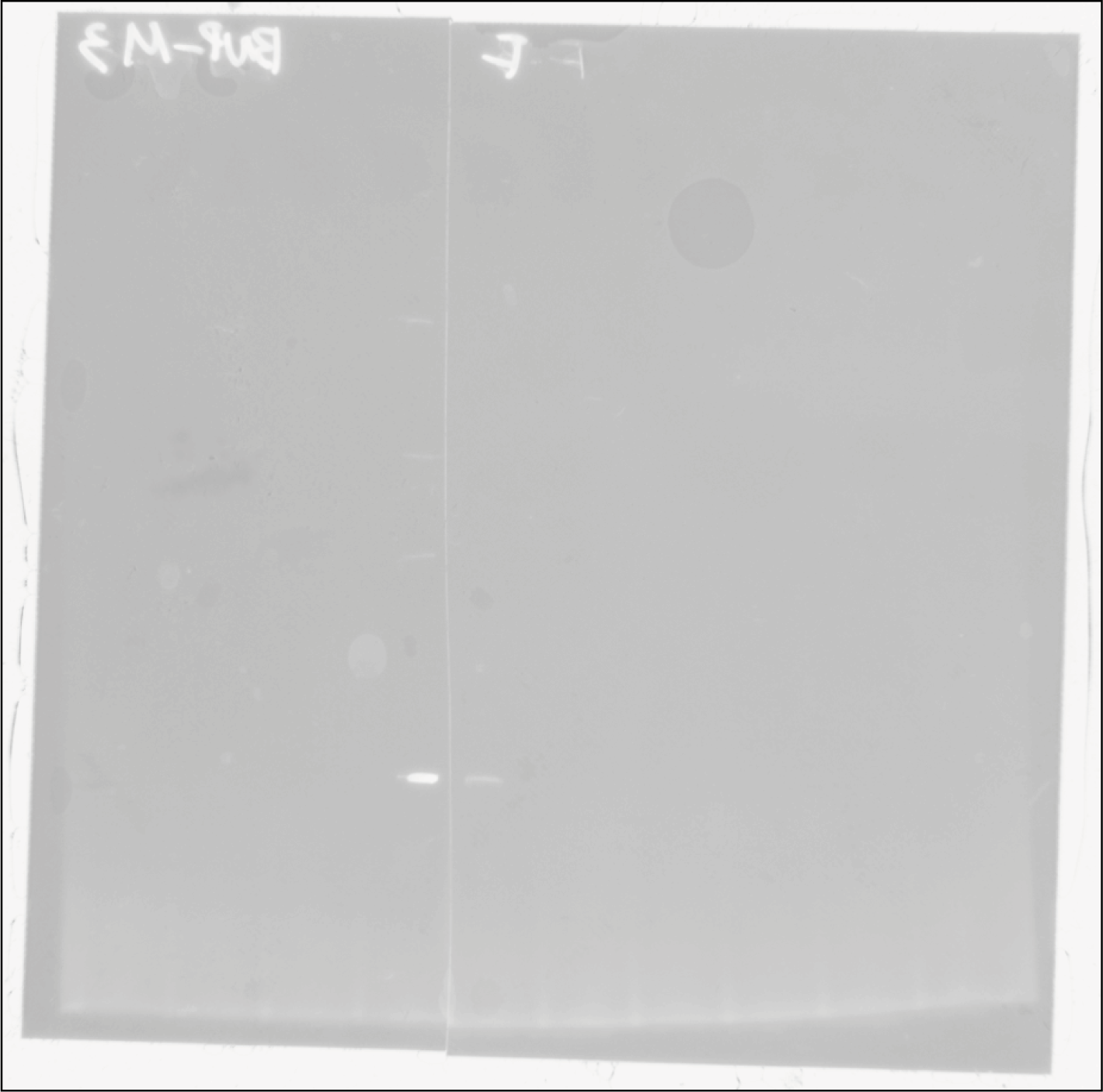

Supplement: Supplementary file 4 — Source Data for Expanded View [file EMBJ-42-e111484-s002.zip › EMBOJ-2022-11484_SourceData/Figure EV2/EV2C/BNP Western Flag Marker.tif]

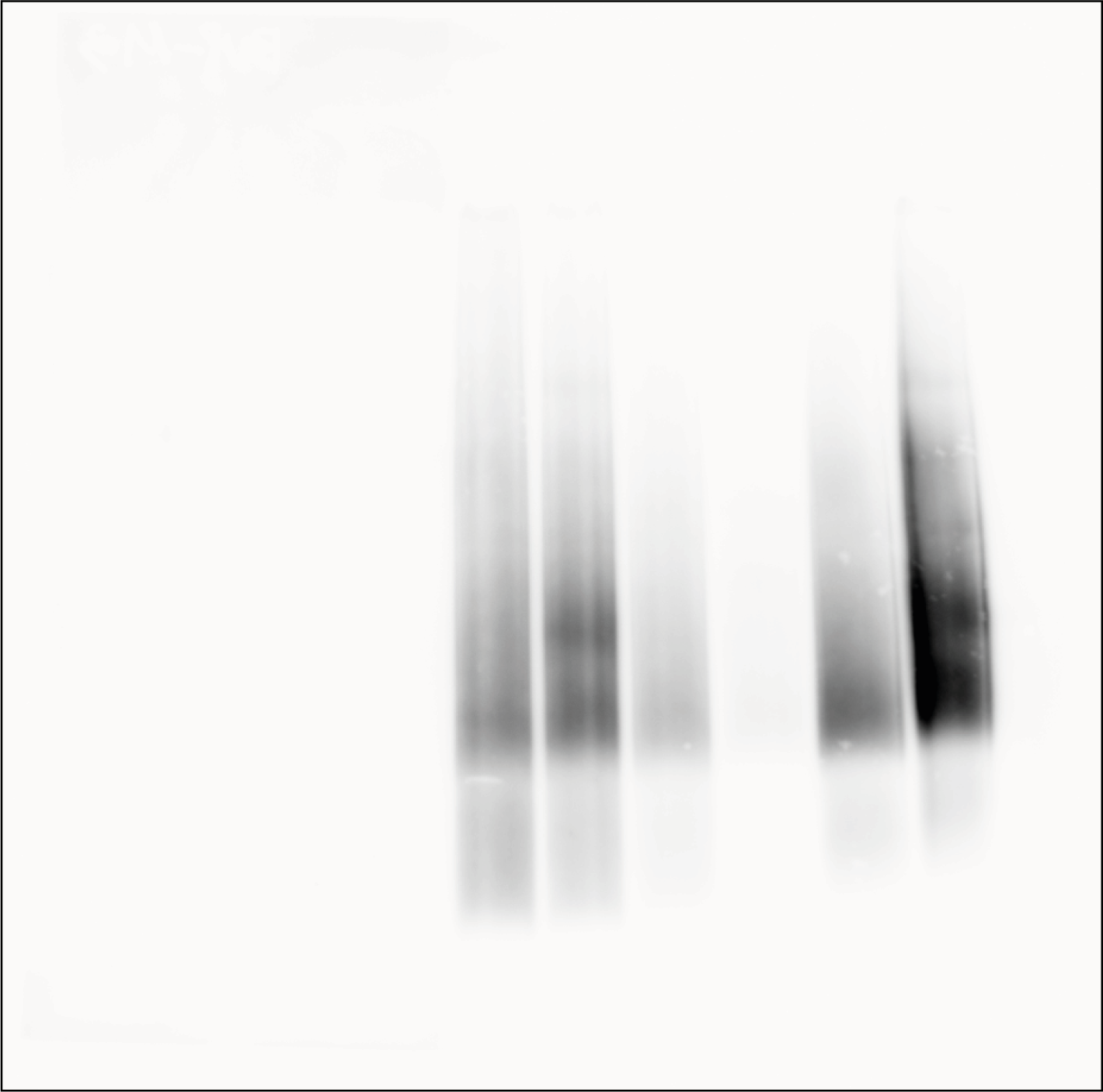

Supplement: Supplementary file 4 — Source Data for Expanded View [file EMBJ-42-e111484-s002.zip › EMBOJ-2022-11484_SourceData/Figure EV2/EV2C/BNP Western Flag.tif]

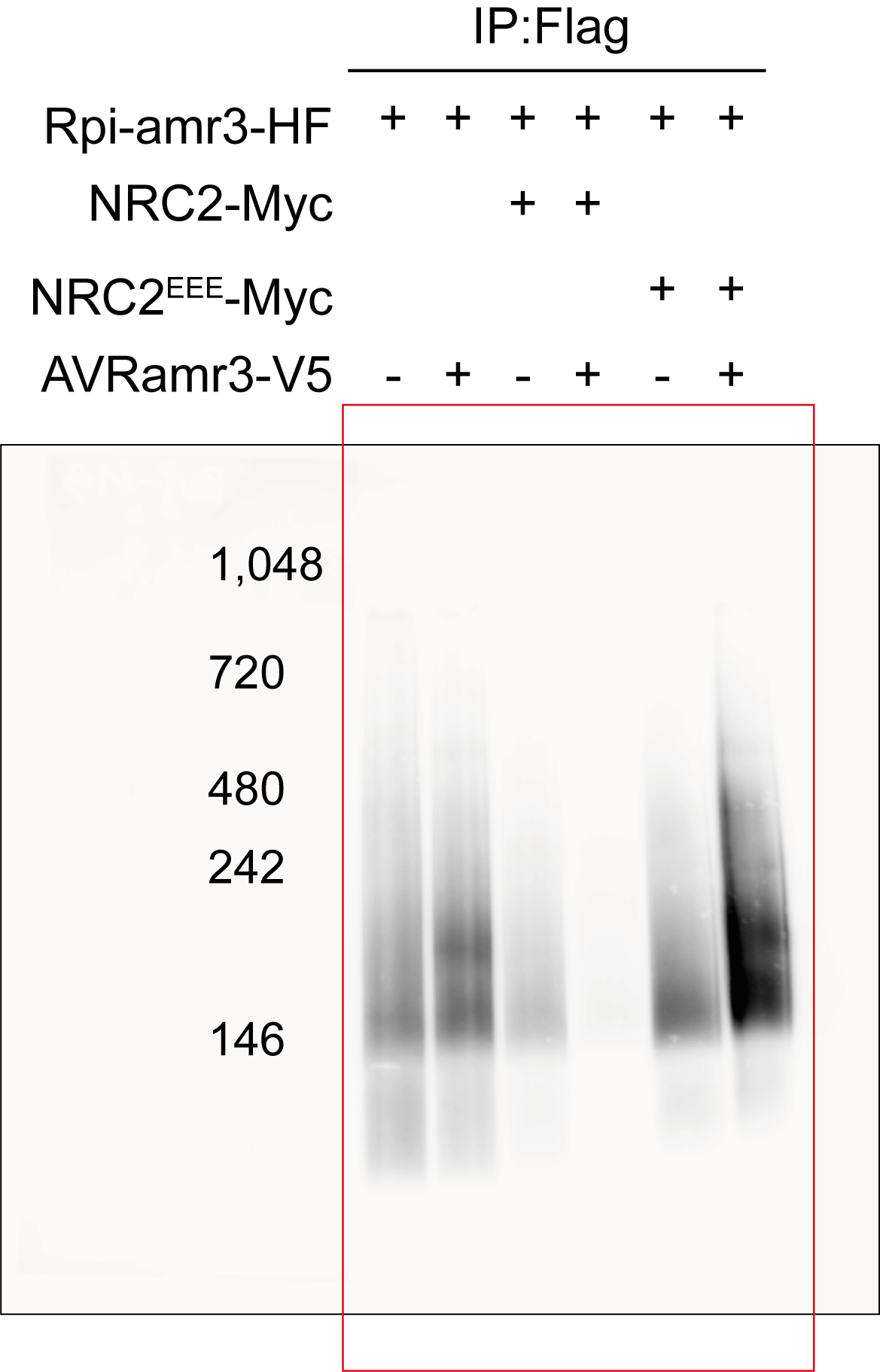

Supplement: Supplementary file 4 — Source Data for Expanded View [file EMBJ-42-e111484-s002.zip › EMBOJ-2022-11484_SourceData/Figure EV2/EV2C/BNP Western Flag_annotation.tif]

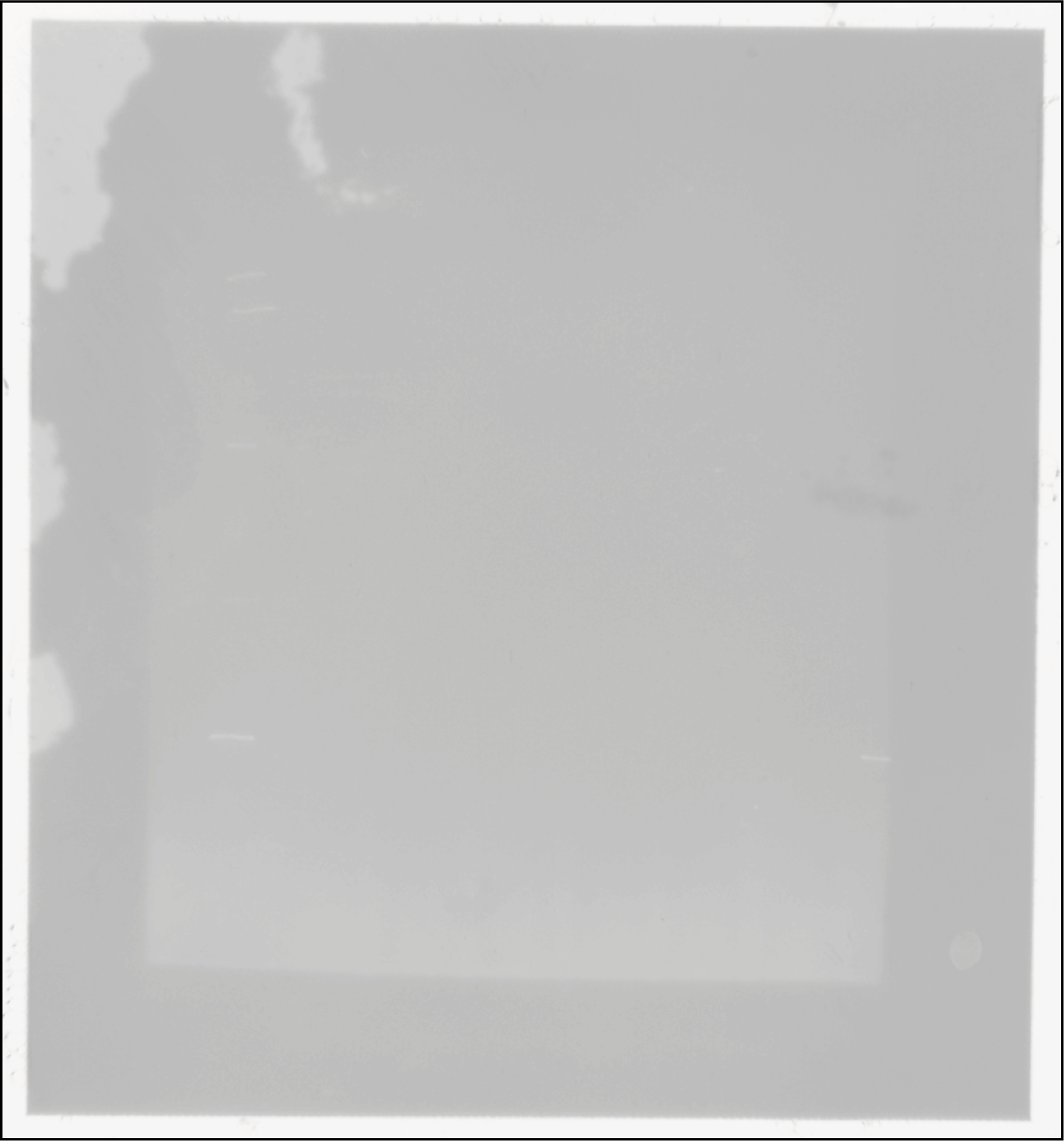

Supplement: Supplementary file 4 — Source Data for Expanded View [file EMBJ-42-e111484-s002.zip › EMBOJ-2022-11484_SourceData/Figure EV2/EV2C/BNP Western V5 Marker.tif]

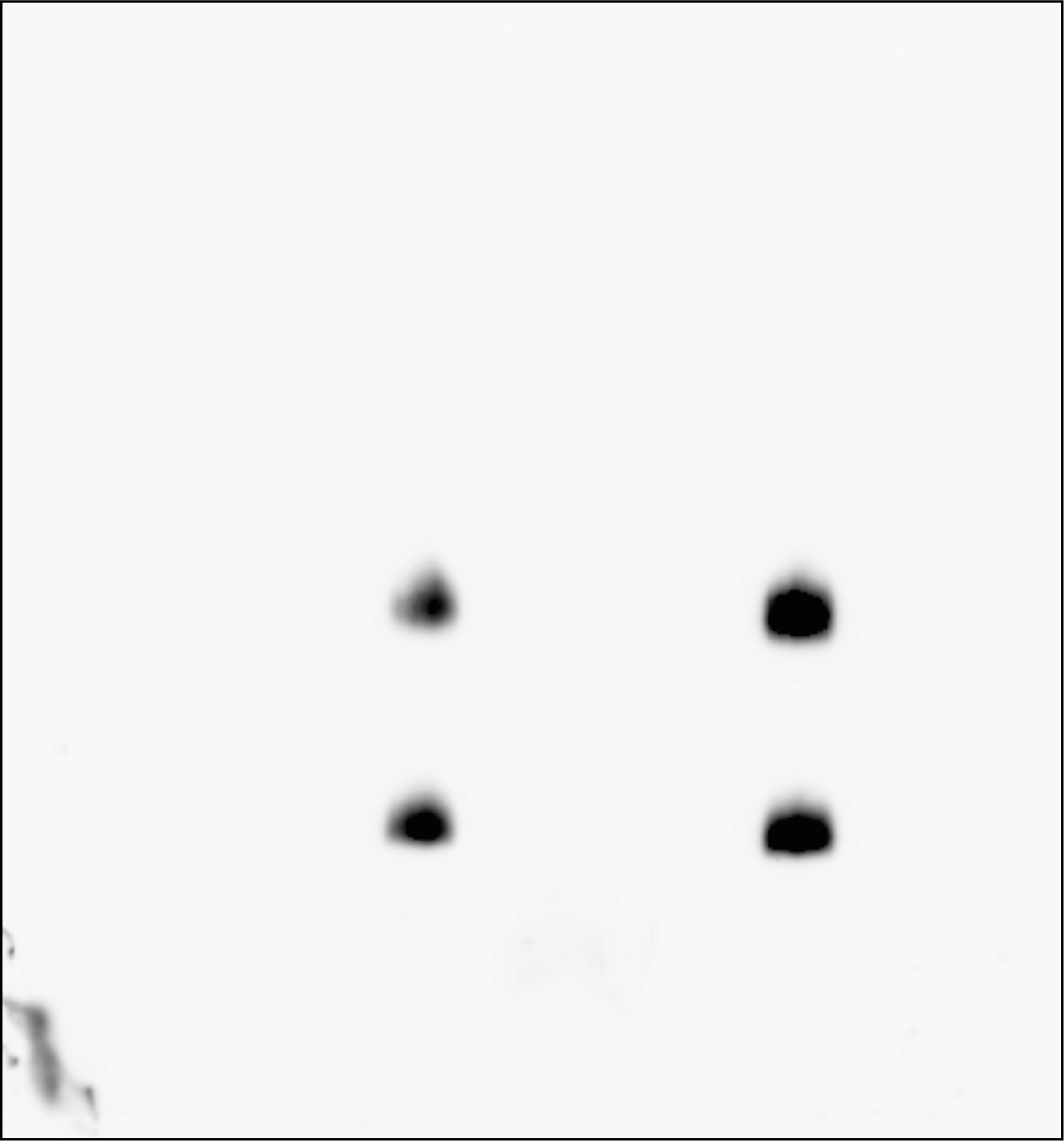

Supplement: Supplementary file 4 — Source Data for Expanded View [file EMBJ-42-e111484-s002.zip › EMBOJ-2022-11484_SourceData/Figure EV2/EV2C/BNP Western V5.tif]

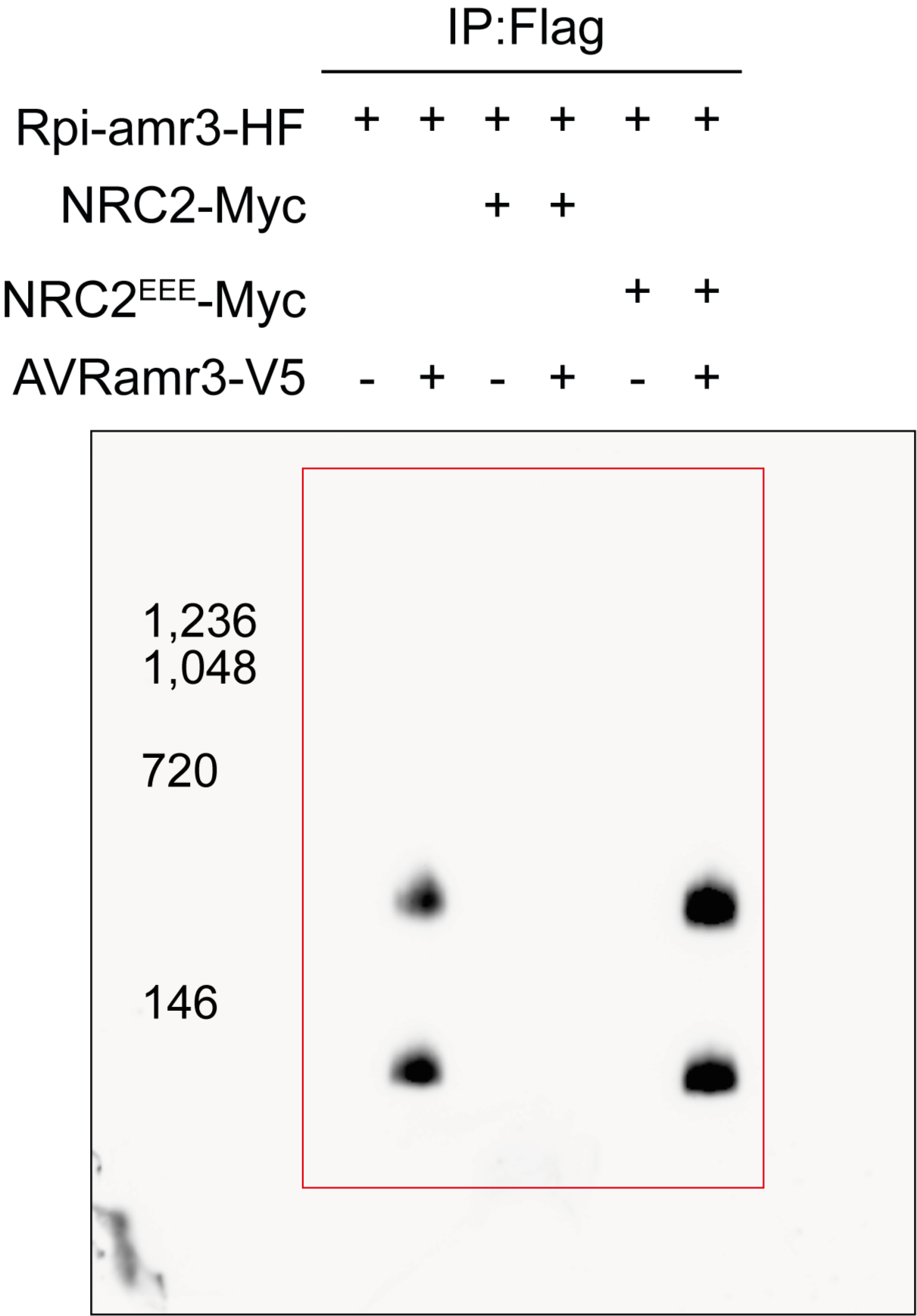

Supplement: Supplementary file 4 — Source Data for Expanded View [file EMBJ-42-e111484-s002.zip › EMBOJ-2022-11484_SourceData/Figure EV2/EV2C/BNP Western V5_annotations.tif]

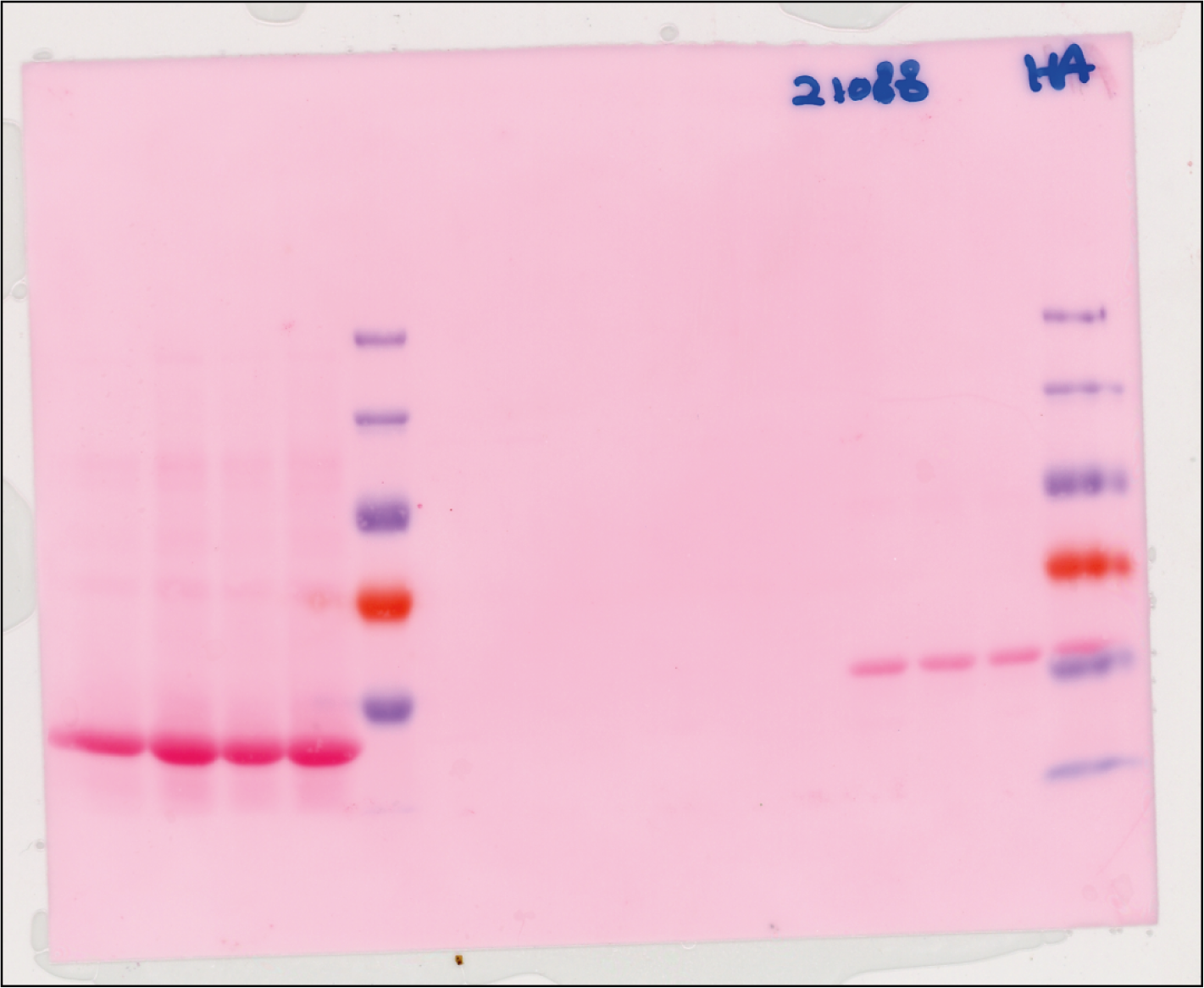

Supplement: Supplementary file 4 — Source Data for Expanded View [file EMBJ-42-e111484-s002.zip › EMBOJ-2022-11484_SourceData/Figure EV3/EV3A/SDS Ponceau.tif]

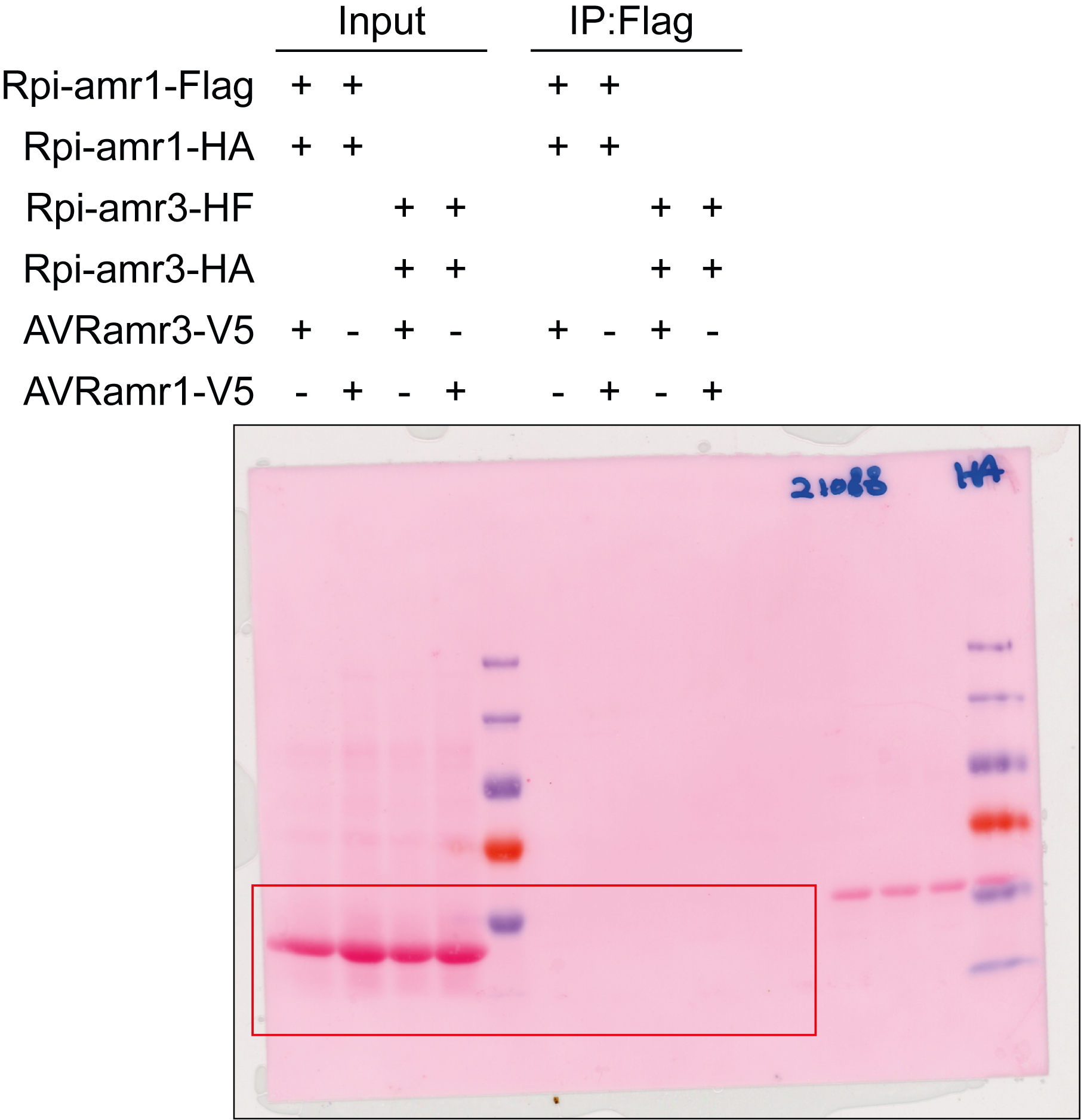

Supplement: Supplementary file 4 — Source Data for Expanded View [file EMBJ-42-e111484-s002.zip › EMBOJ-2022-11484_SourceData/Figure EV3/EV3A/SDS Ponceau_annotations.tif]

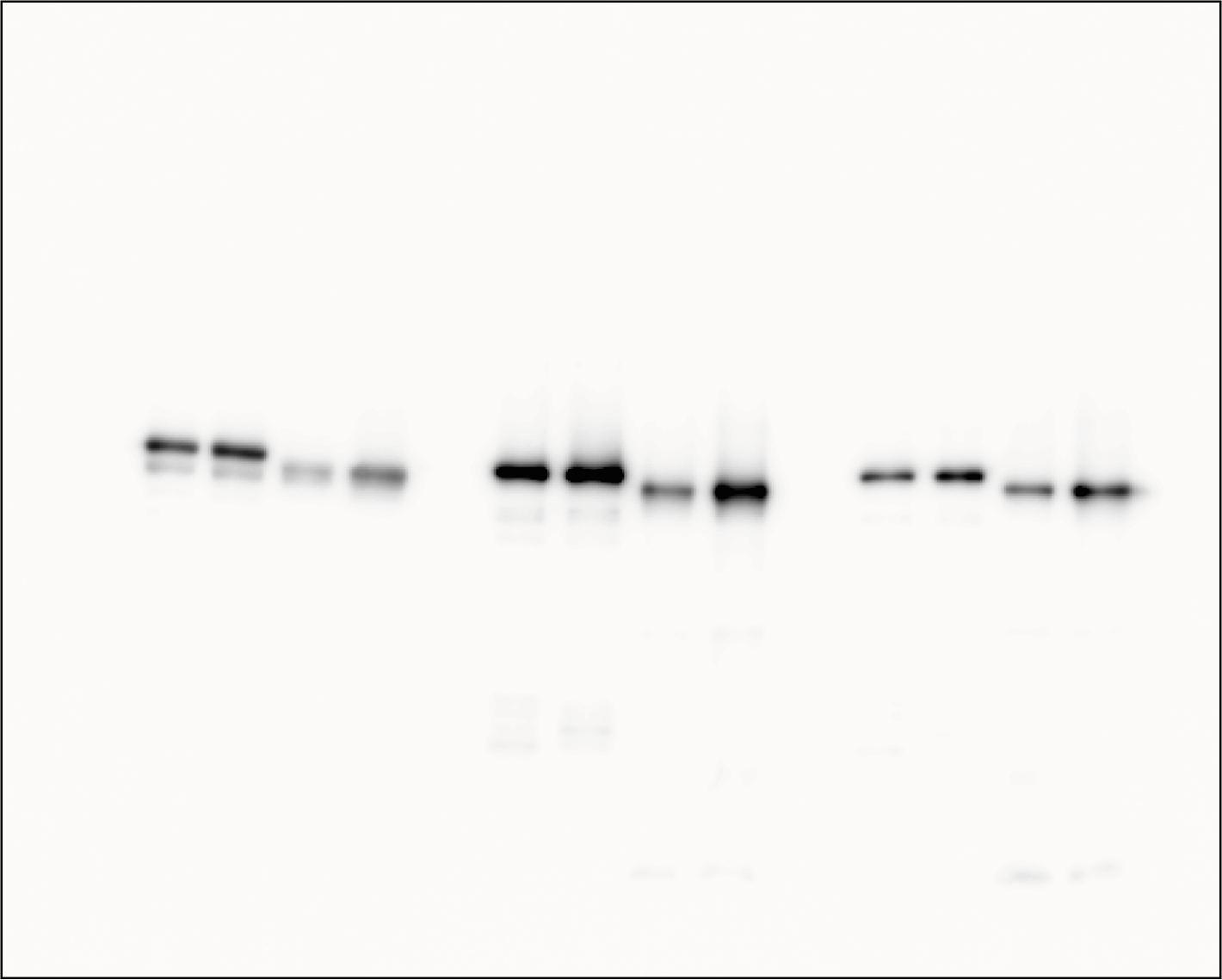

Supplement: Supplementary file 4 — Source Data for Expanded View [file EMBJ-42-e111484-s002.zip › EMBOJ-2022-11484_SourceData/Figure EV3/EV3A/Western Flag.tif]

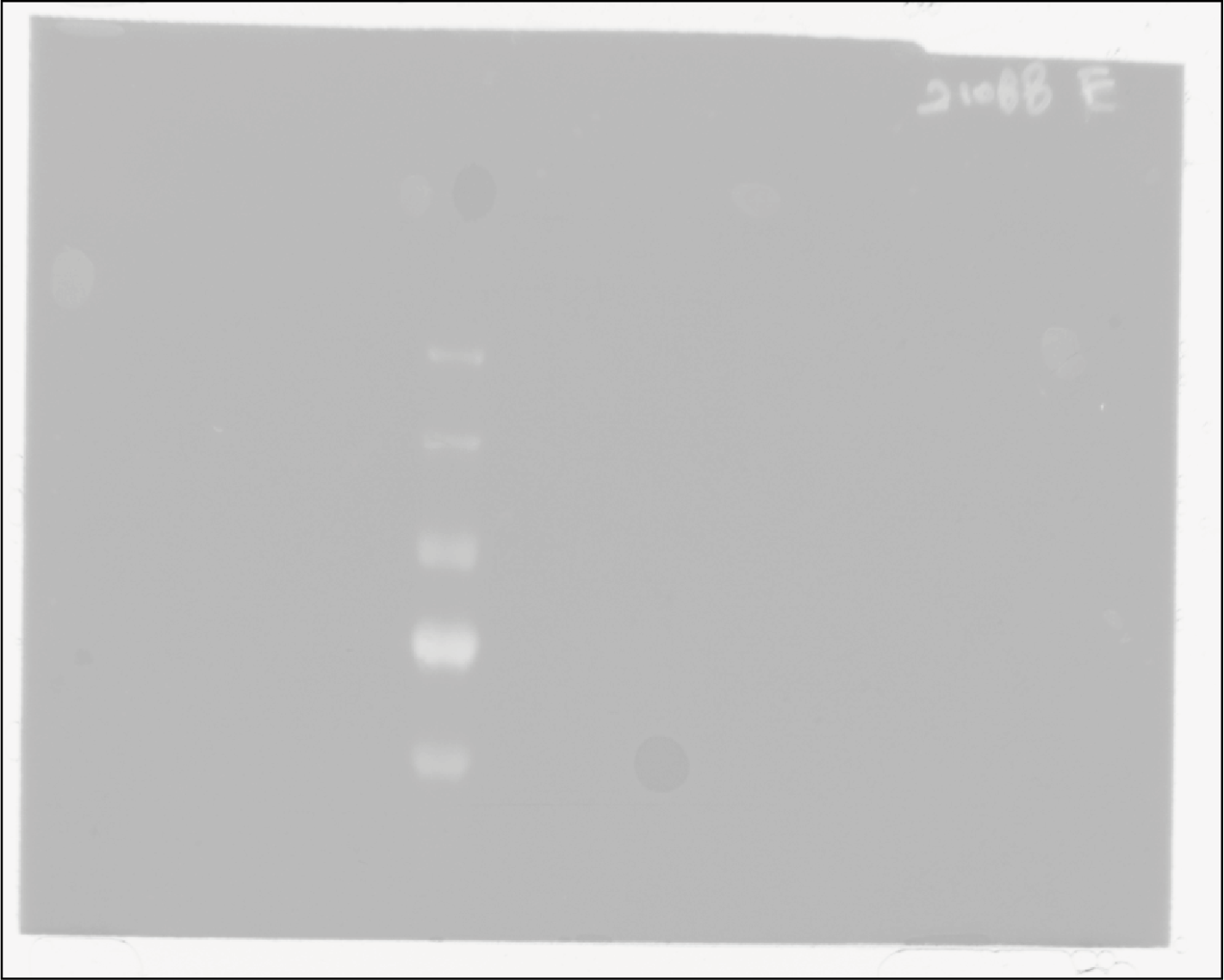

Supplement: Supplementary file 4 — Source Data for Expanded View [file EMBJ-42-e111484-s002.zip › EMBOJ-2022-11484_SourceData/Figure EV3/EV3A/Western Flag_Marker.tif]

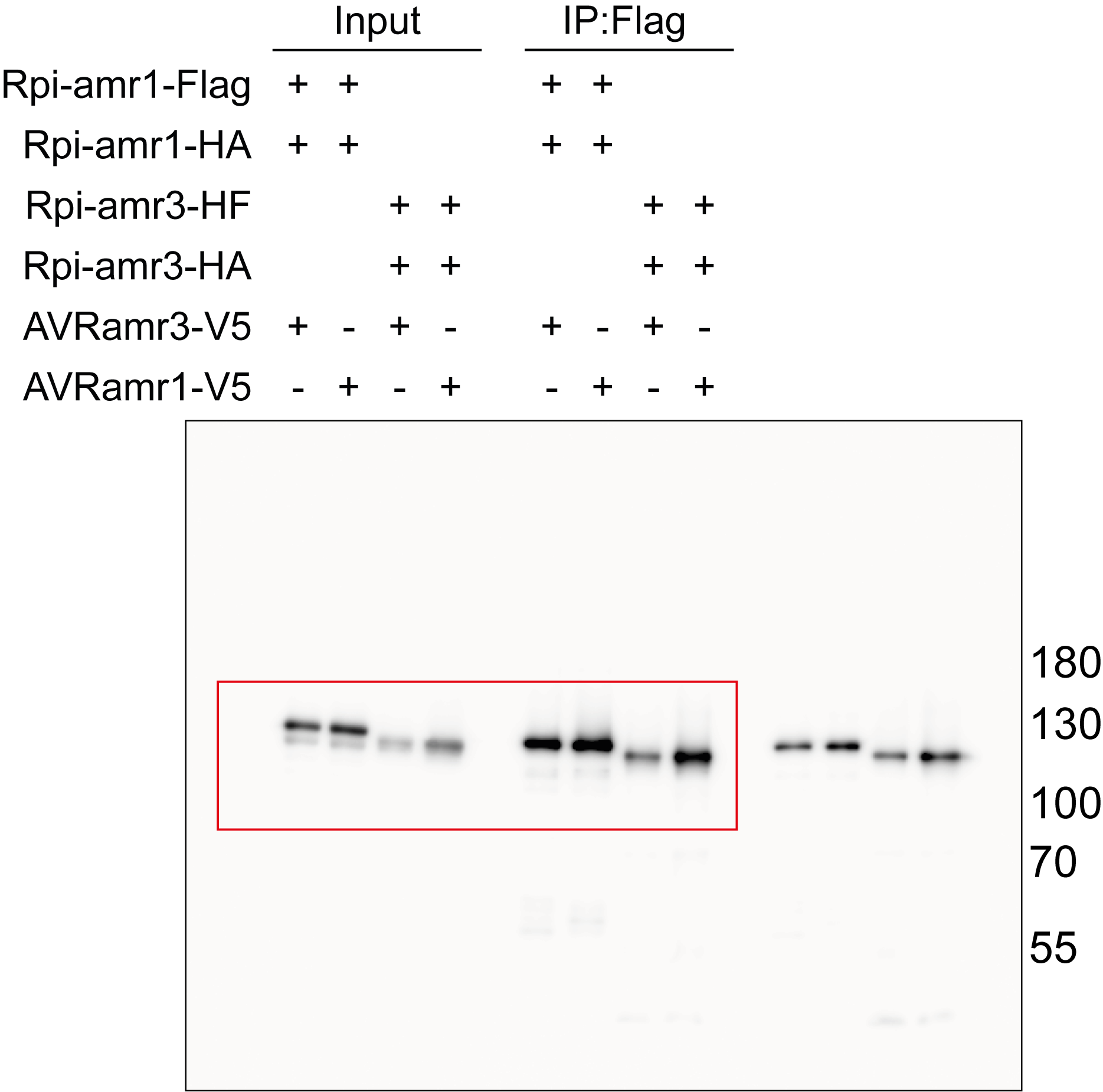

Supplement: Supplementary file 4 — Source Data for Expanded View [file EMBJ-42-e111484-s002.zip › EMBOJ-2022-11484_SourceData/Figure EV3/EV3A/Western Flag_annotations.tif]

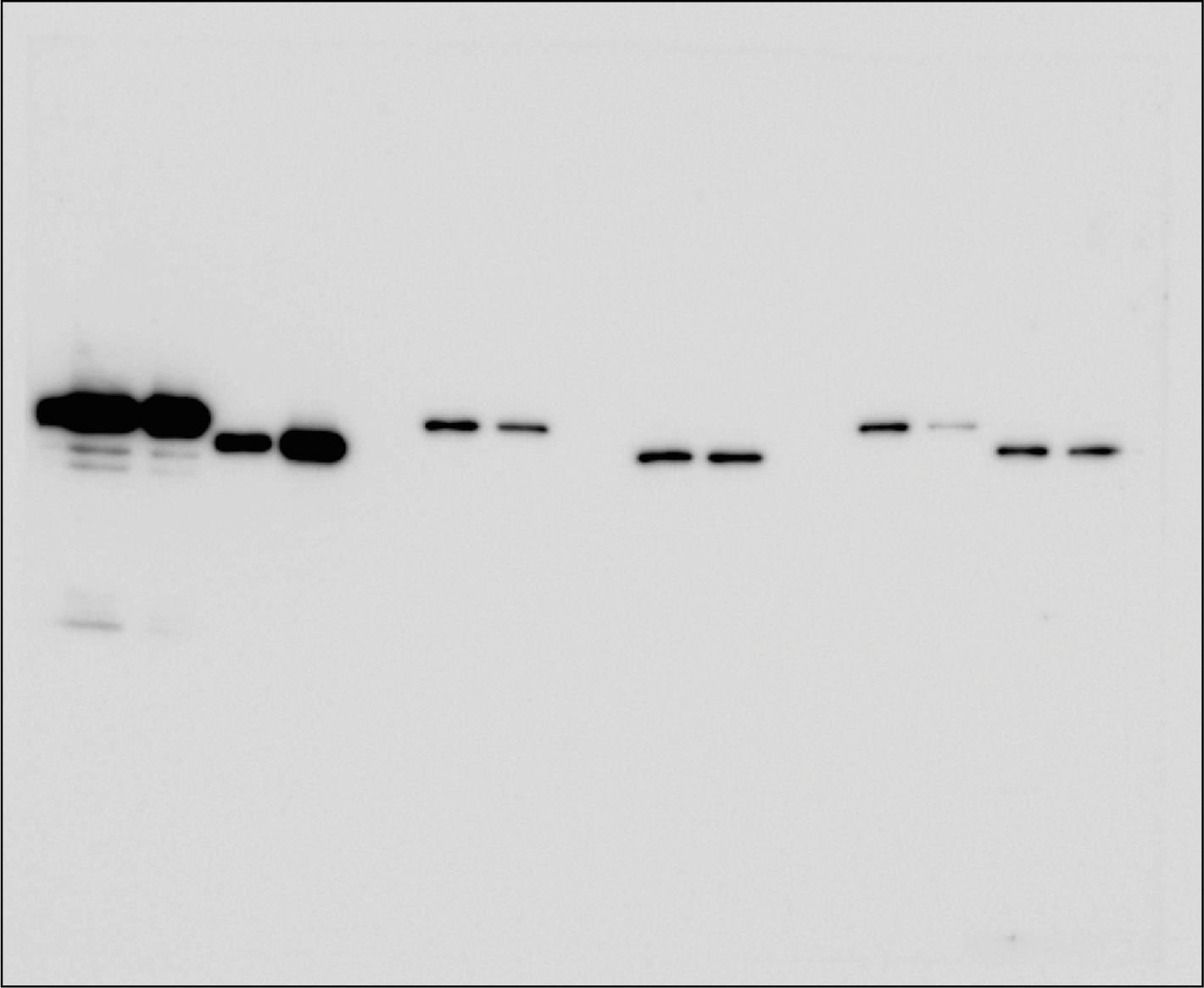

Supplement: Supplementary file 4 — Source Data for Expanded View [file EMBJ-42-e111484-s002.zip › EMBOJ-2022-11484_SourceData/Figure EV3/EV3A/Western HA HIGH.tif]

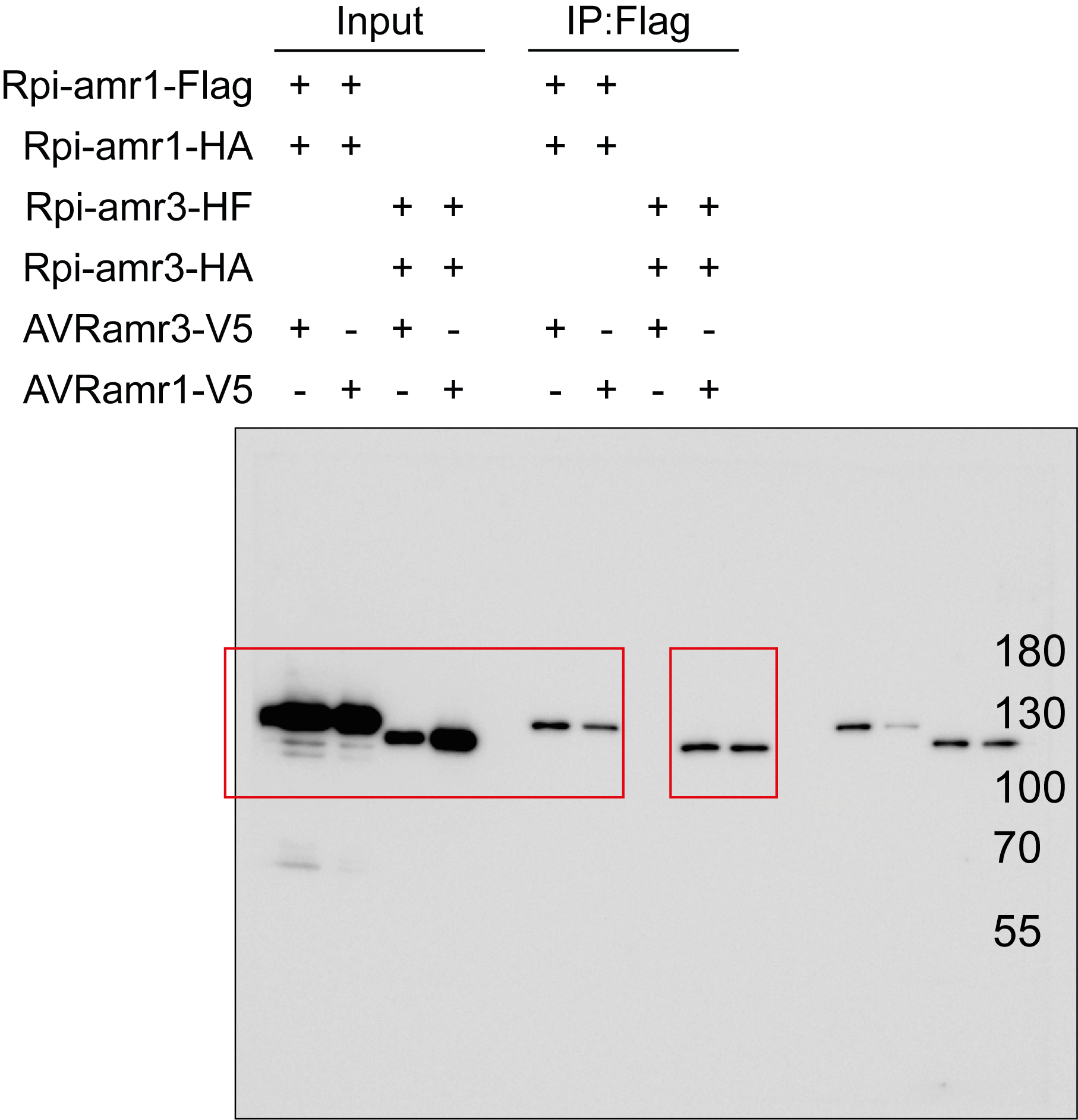

Supplement: Supplementary file 4 — Source Data for Expanded View [file EMBJ-42-e111484-s002.zip › EMBOJ-2022-11484_SourceData/Figure EV3/EV3A/Western HA HIGH_annotations.tif]

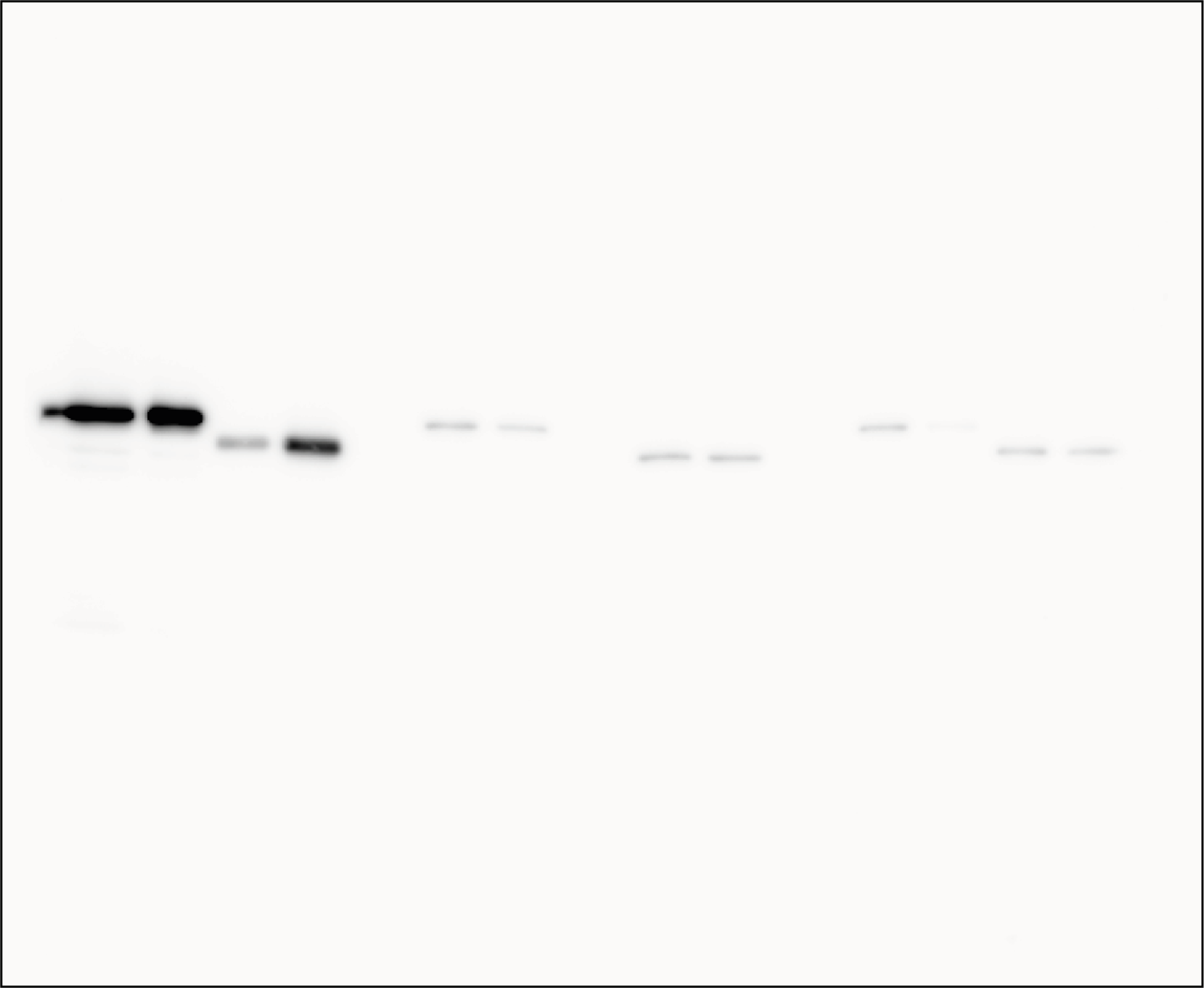

Supplement: Supplementary file 4 — Source Data for Expanded View [file EMBJ-42-e111484-s002.zip › EMBOJ-2022-11484_SourceData/Figure EV3/EV3A/Western HA LOW.tif]

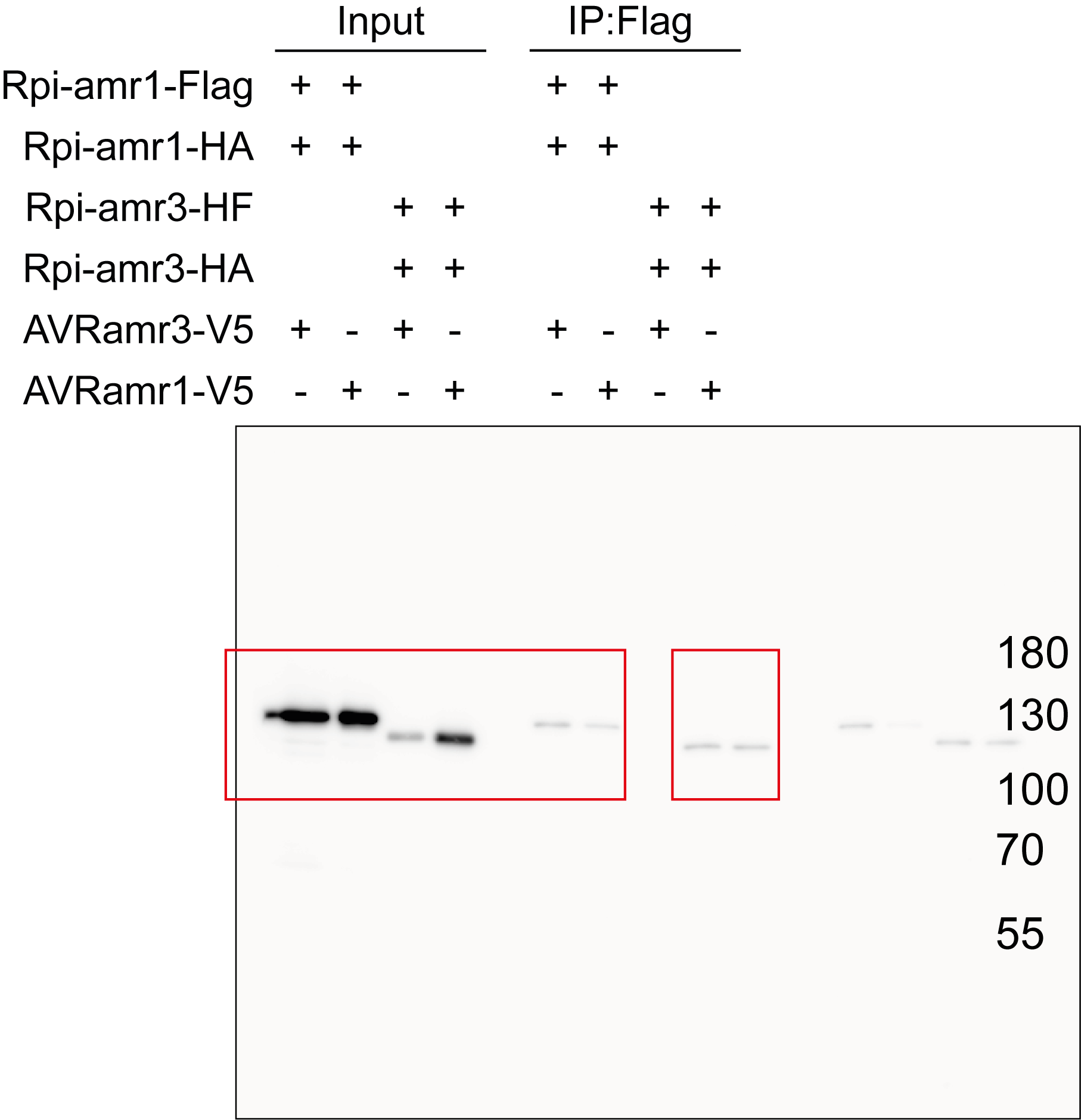

Supplement: Supplementary file 4 — Source Data for Expanded View [file EMBJ-42-e111484-s002.zip › EMBOJ-2022-11484_SourceData/Figure EV3/EV3A/Western HA LOW_annotations.tif]

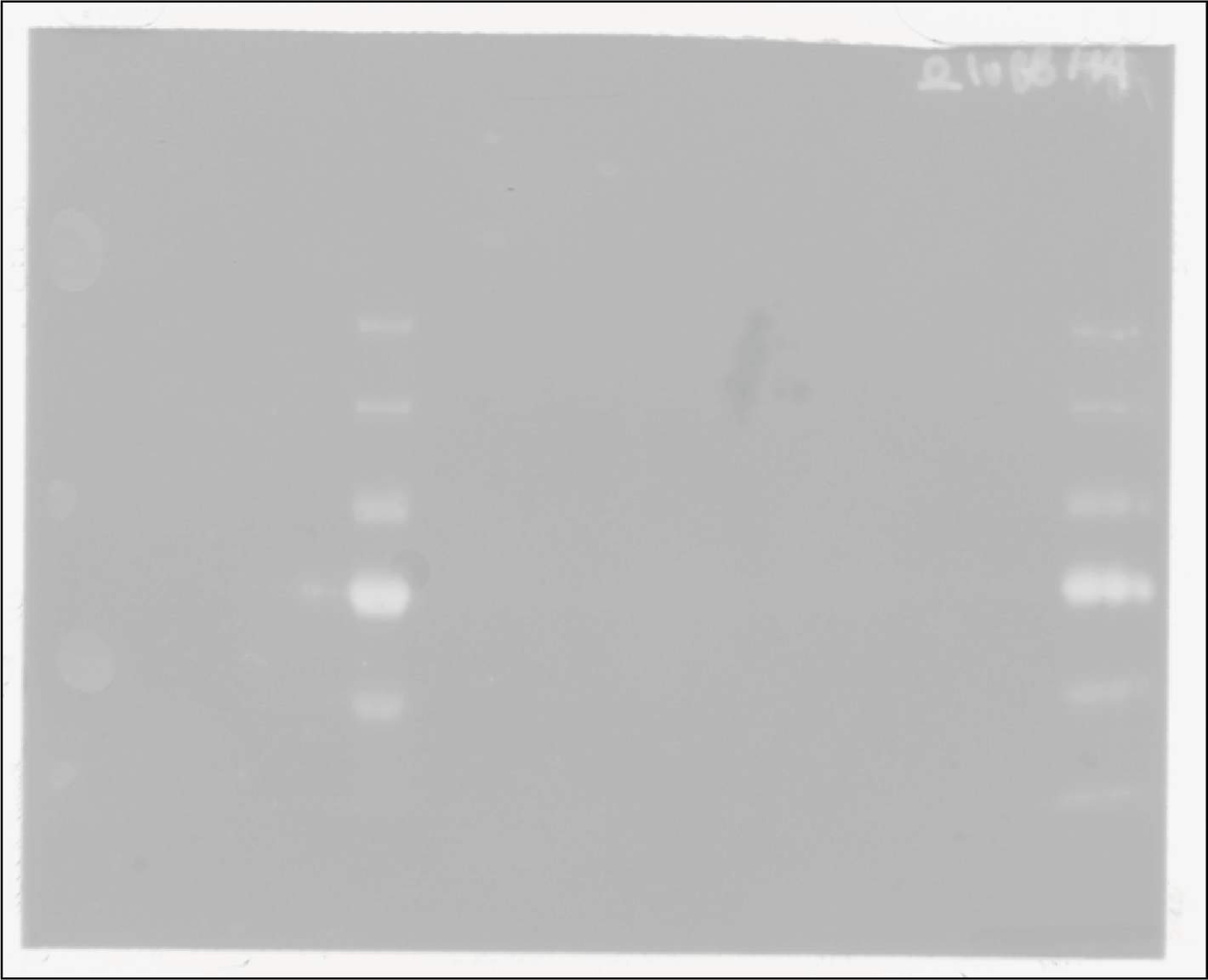

Supplement: Supplementary file 4 — Source Data for Expanded View [file EMBJ-42-e111484-s002.zip › EMBOJ-2022-11484_SourceData/Figure EV3/EV3A/Western HA Marker.tif]

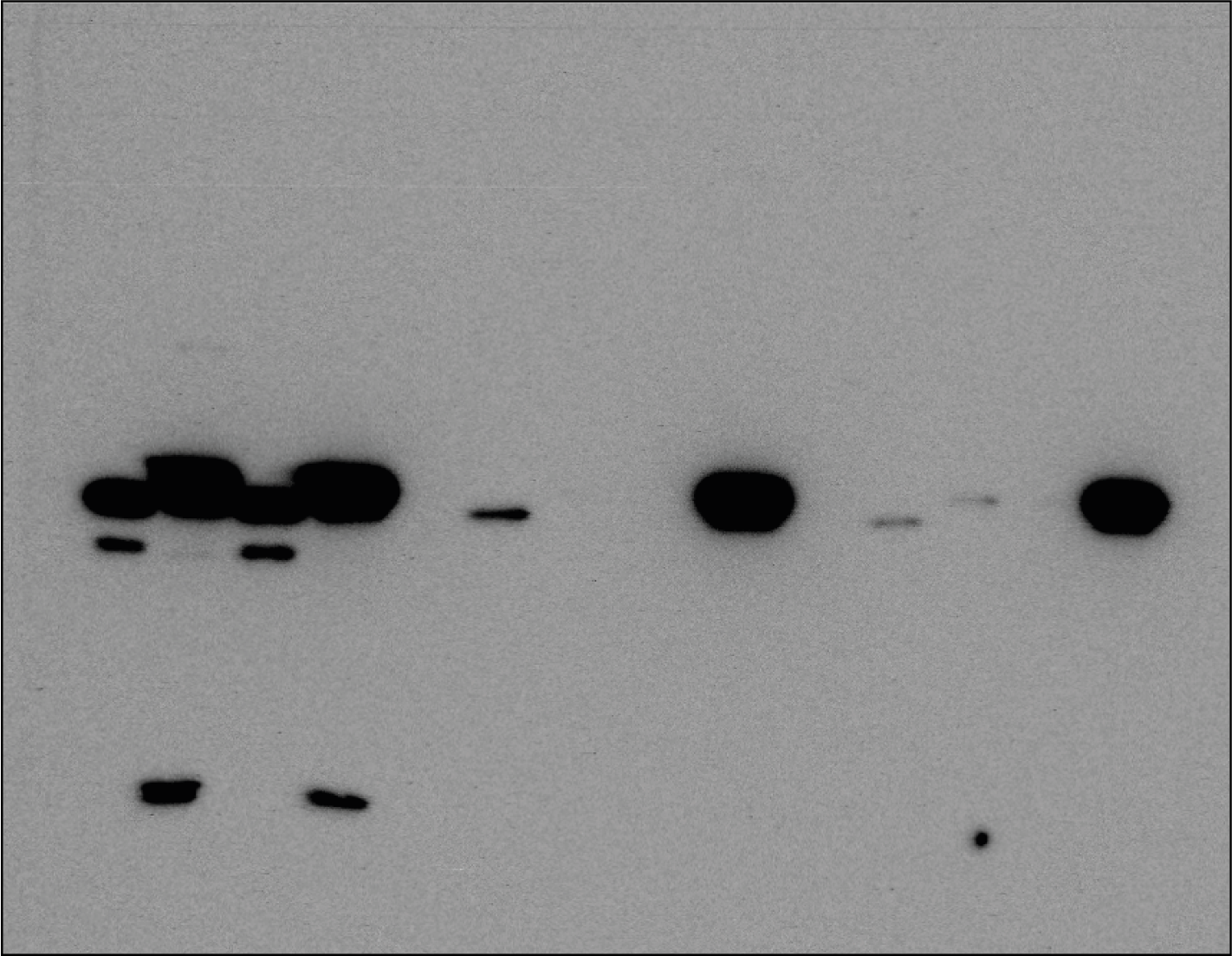

Supplement: Supplementary file 4 — Source Data for Expanded View [file EMBJ-42-e111484-s002.zip › EMBOJ-2022-11484_SourceData/Figure EV3/EV3A/Western V5 HIGH.tif]

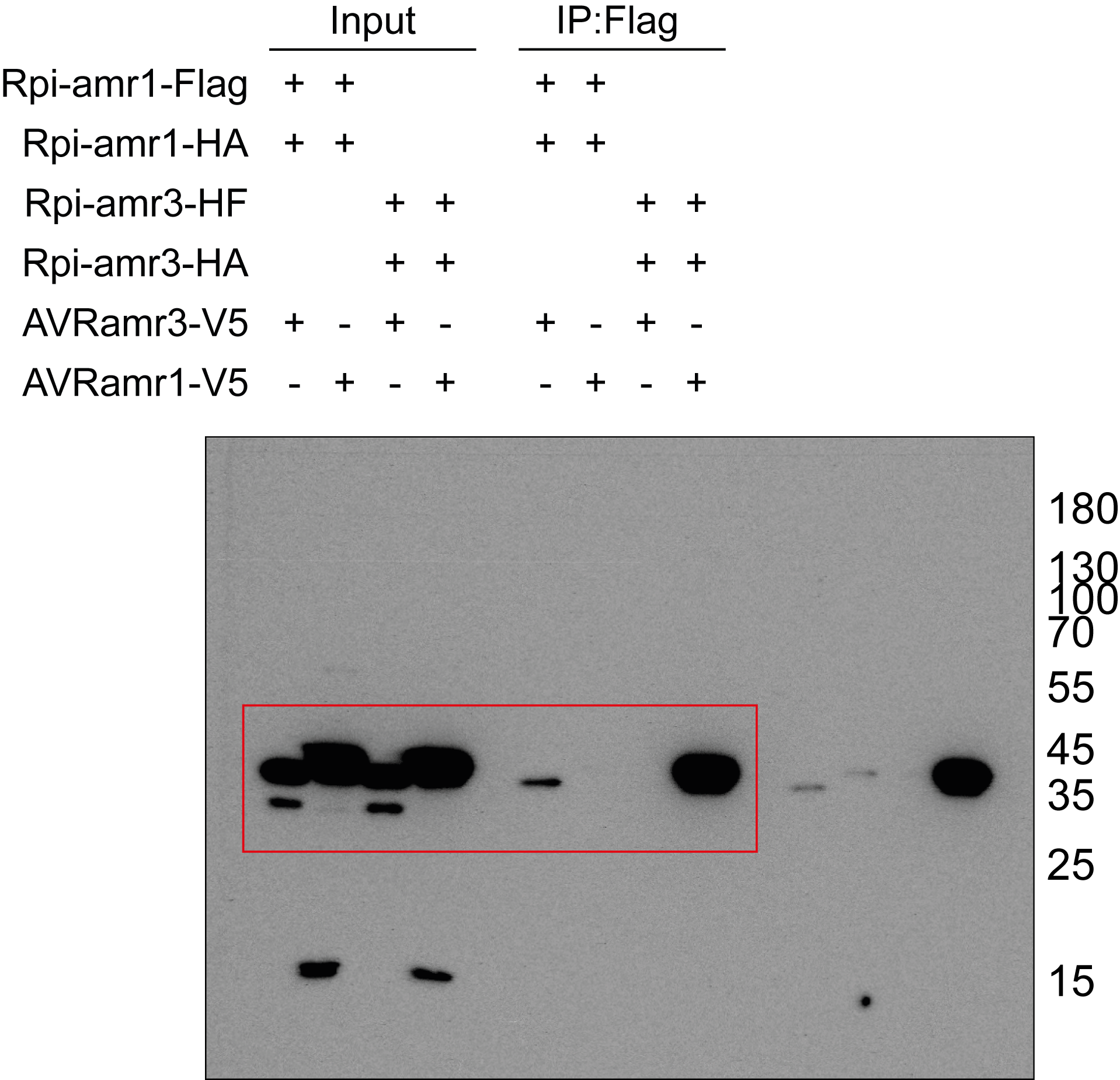

Supplement: Supplementary file 4 — Source Data for Expanded View [file EMBJ-42-e111484-s002.zip › EMBOJ-2022-11484_SourceData/Figure EV3/EV3A/Western V5 HIGH_annotations.tif]

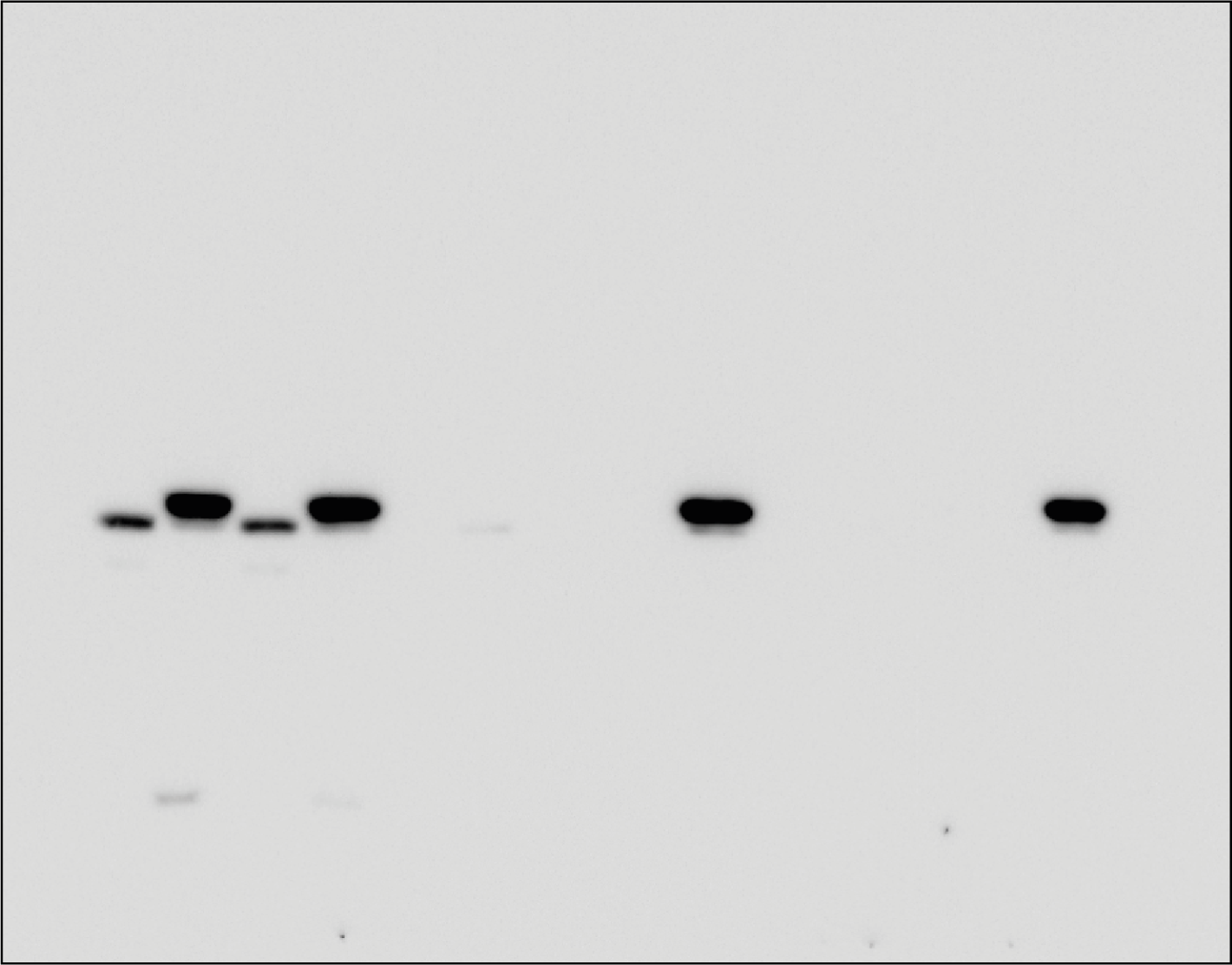

Supplement: Supplementary file 4 — Source Data for Expanded View [file EMBJ-42-e111484-s002.zip › EMBOJ-2022-11484_SourceData/Figure EV3/EV3A/Western V5 LOW.tif]

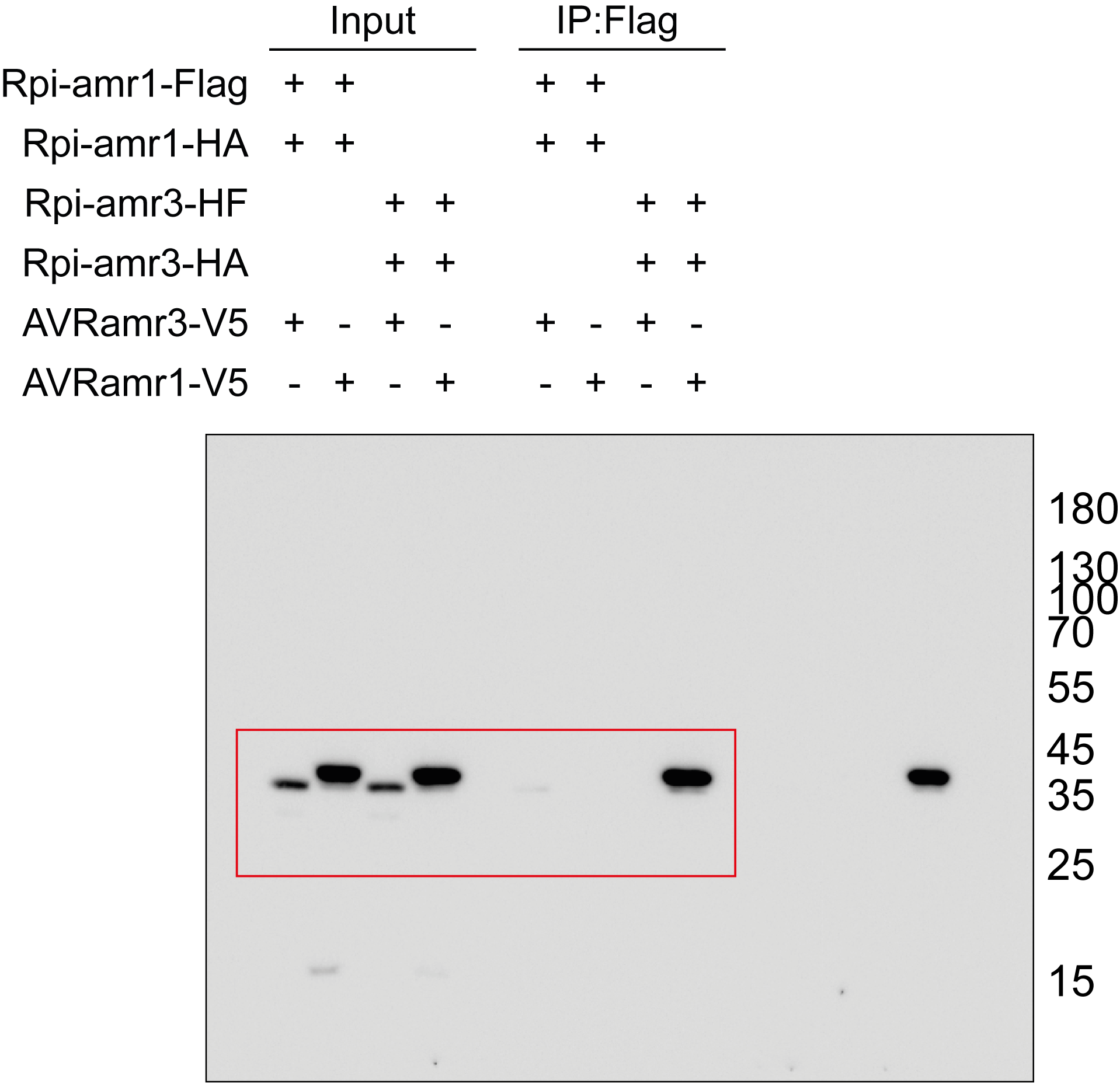

Supplement: Supplementary file 4 — Source Data for Expanded View [file EMBJ-42-e111484-s002.zip › EMBOJ-2022-11484_SourceData/Figure EV3/EV3A/Western V5 LOW_annotations.tif]

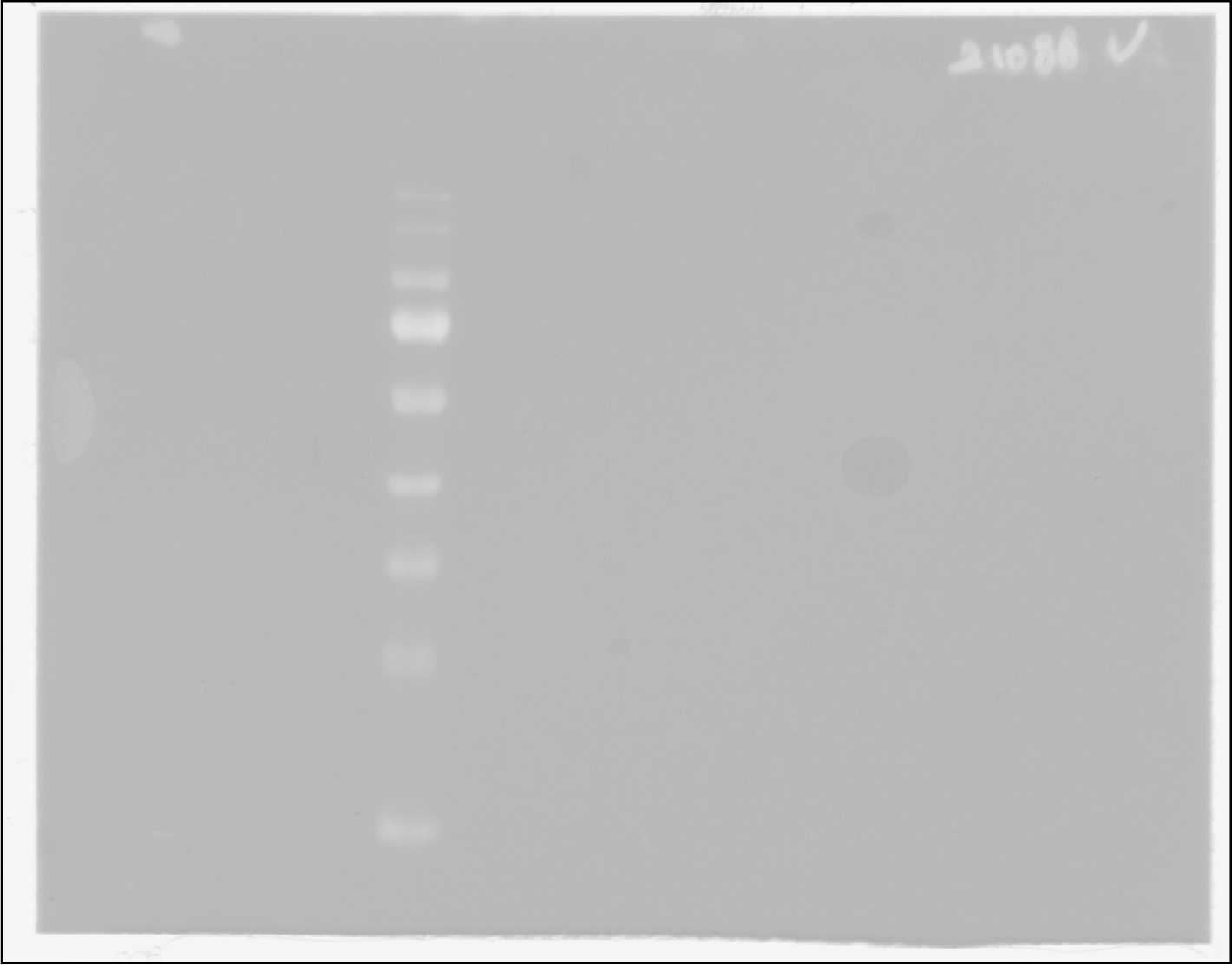

Supplement: Supplementary file 4 — Source Data for Expanded View [file EMBJ-42-e111484-s002.zip › EMBOJ-2022-11484_SourceData/Figure EV3/EV3A/Western V5 Marker.tif]

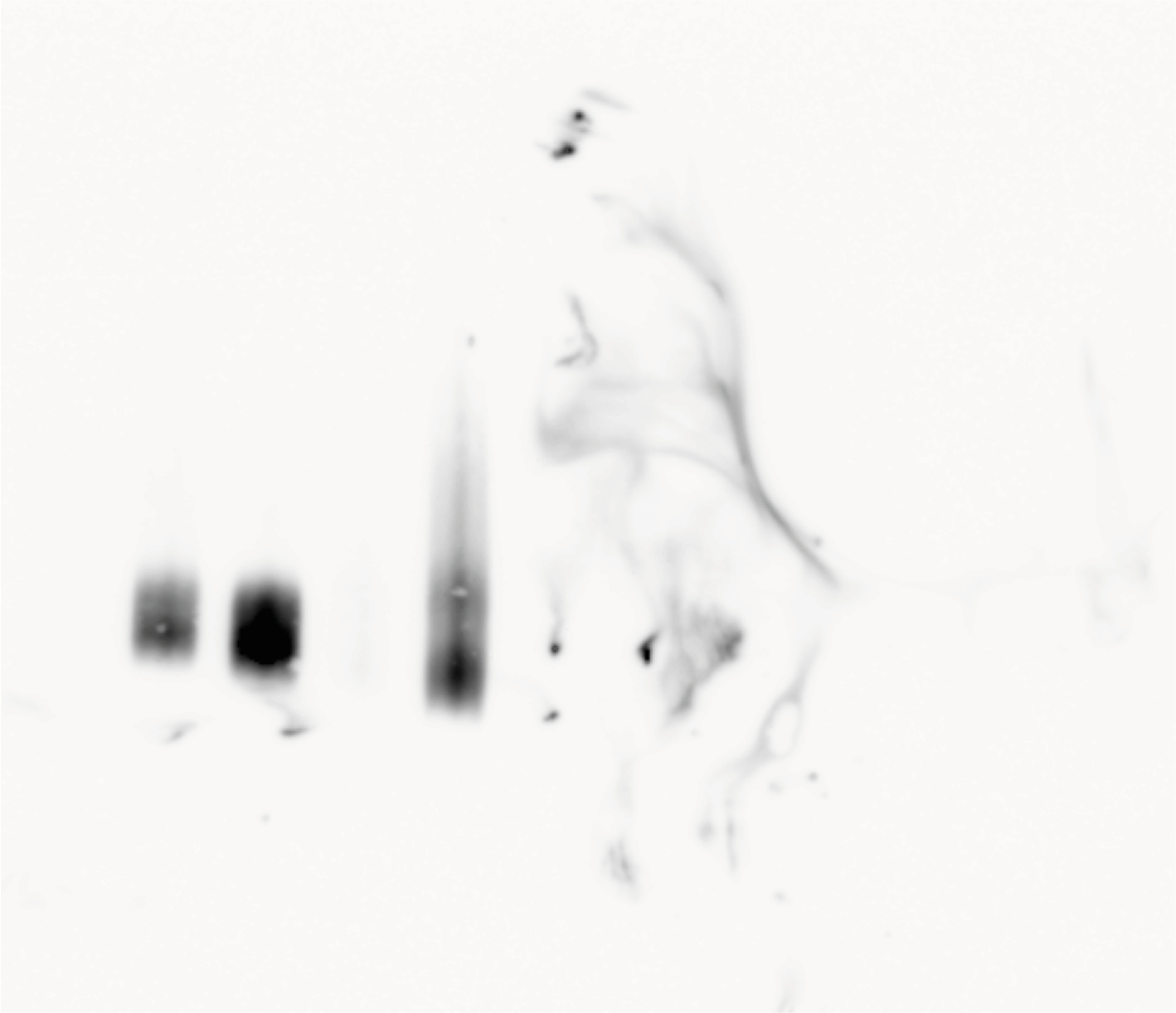

Supplement: Supplementary file 4 — Source Data for Expanded View [file EMBJ-42-e111484-s002.zip › EMBOJ-2022-11484_SourceData/Figure EV3/EV3B/BNP Western Flag LOW (3B).tif]

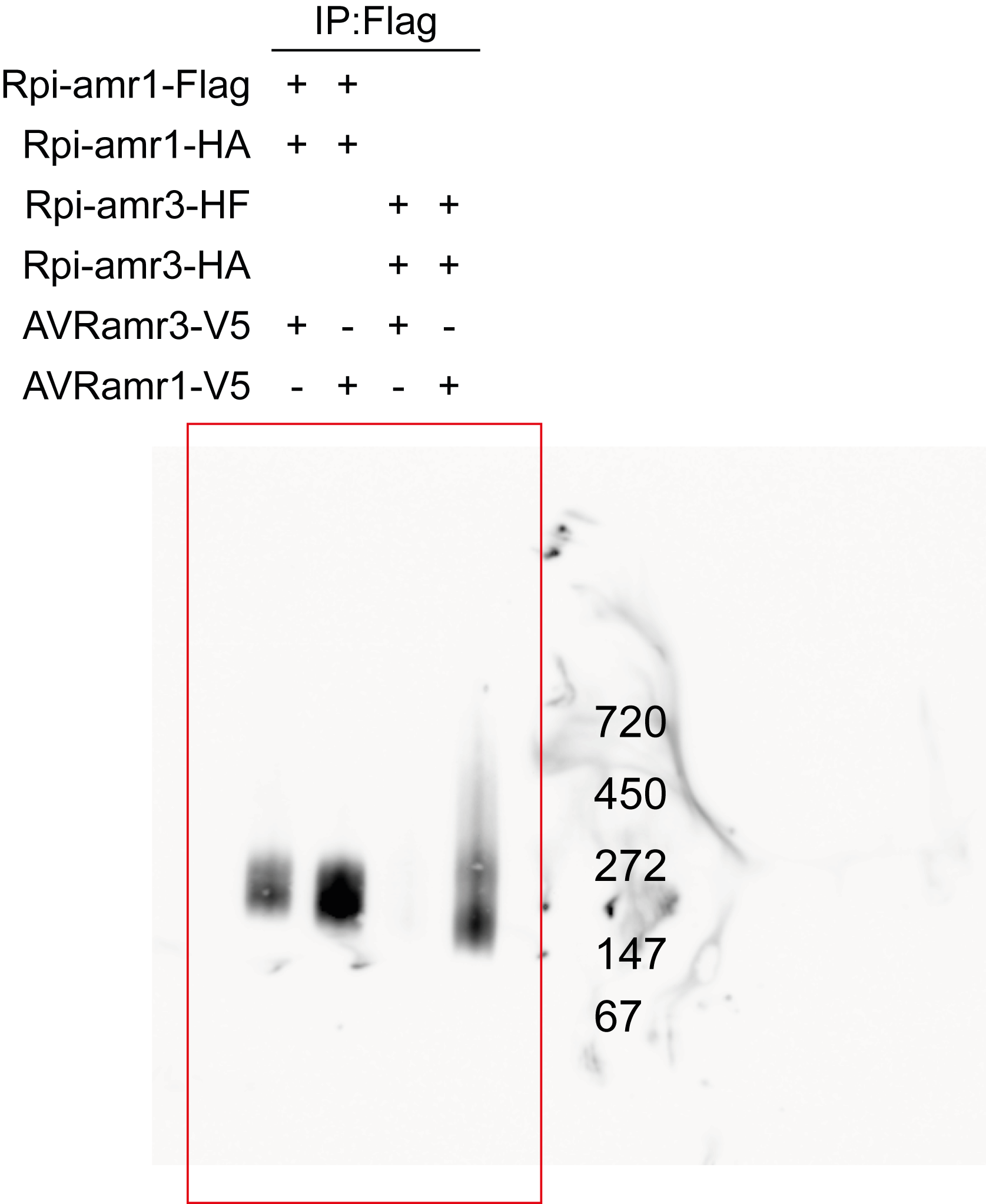

Supplement: Supplementary file 4 — Source Data for Expanded View [file EMBJ-42-e111484-s002.zip › EMBOJ-2022-11484_SourceData/Figure EV3/EV3B/BNP Western Flag LOW_annotation.tif]

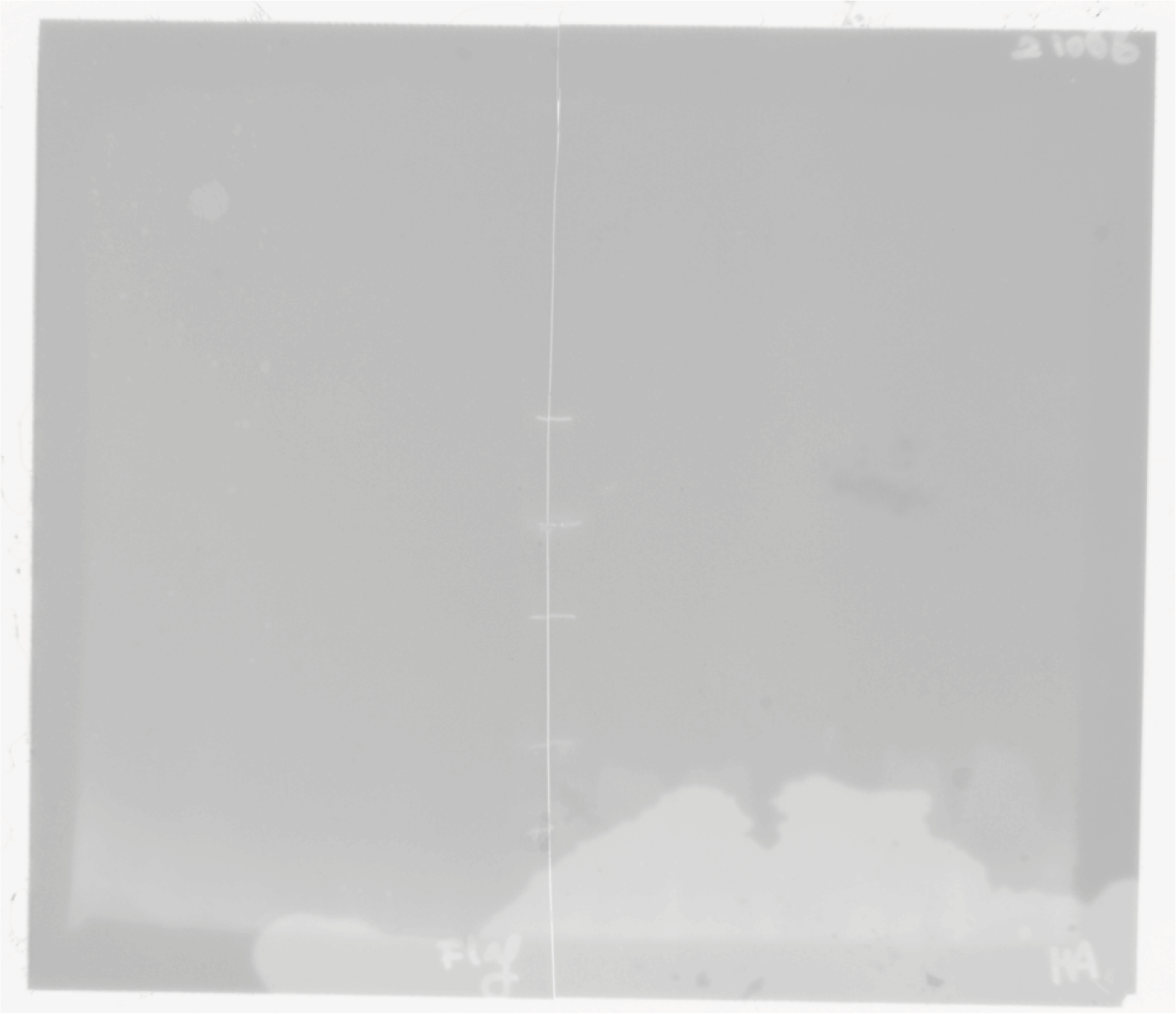

Supplement: Supplementary file 4 — Source Data for Expanded View [file EMBJ-42-e111484-s002.zip › EMBOJ-2022-11484_SourceData/Figure EV3/EV3B/BNP Western Marker Flag.tif]

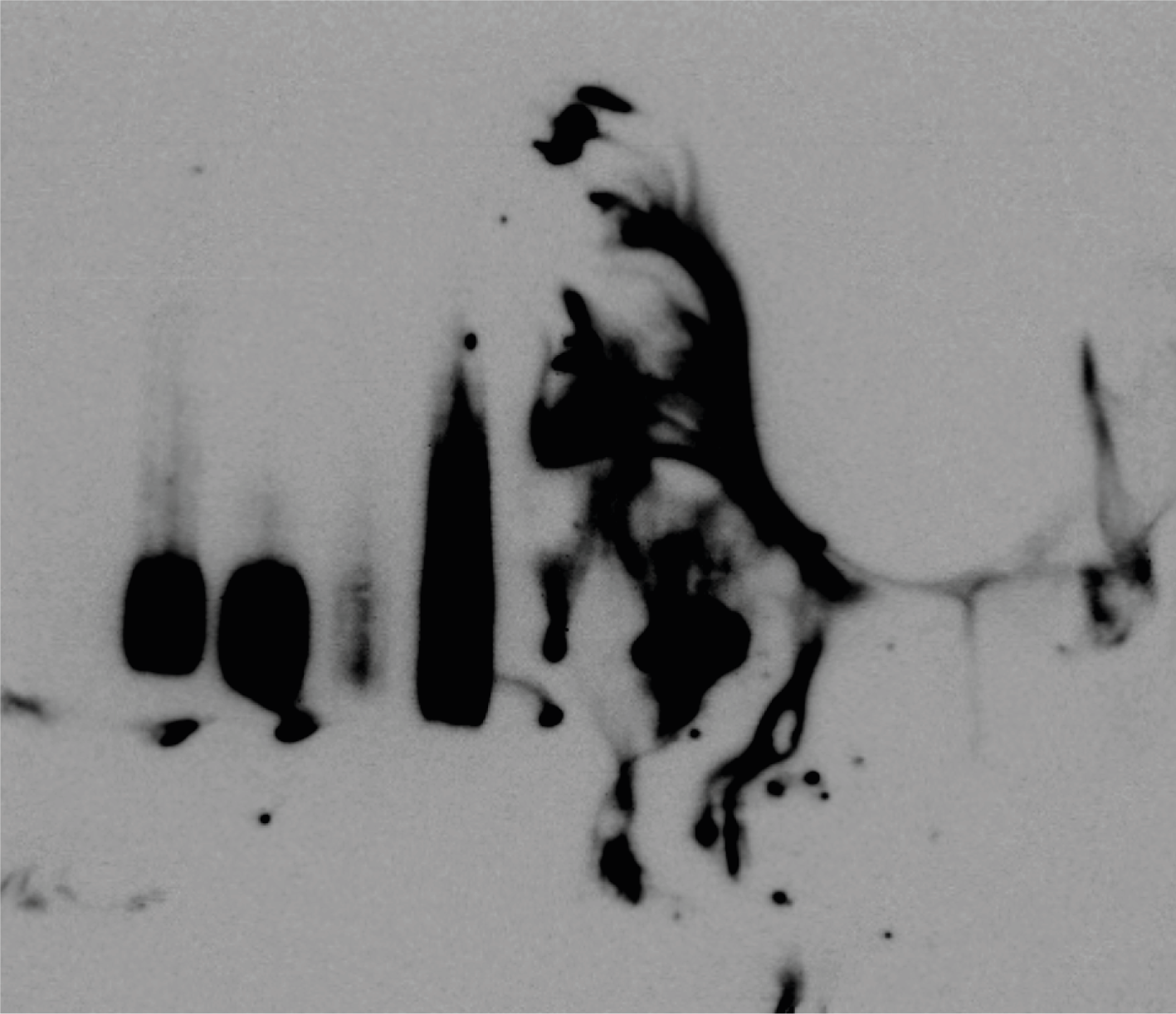

Supplement: Supplementary file 4 — Source Data for Expanded View [file EMBJ-42-e111484-s002.zip › EMBOJ-2022-11484_SourceData/Figure EV3/EV3C/BNP Western Flag HIGH (3C).tif]

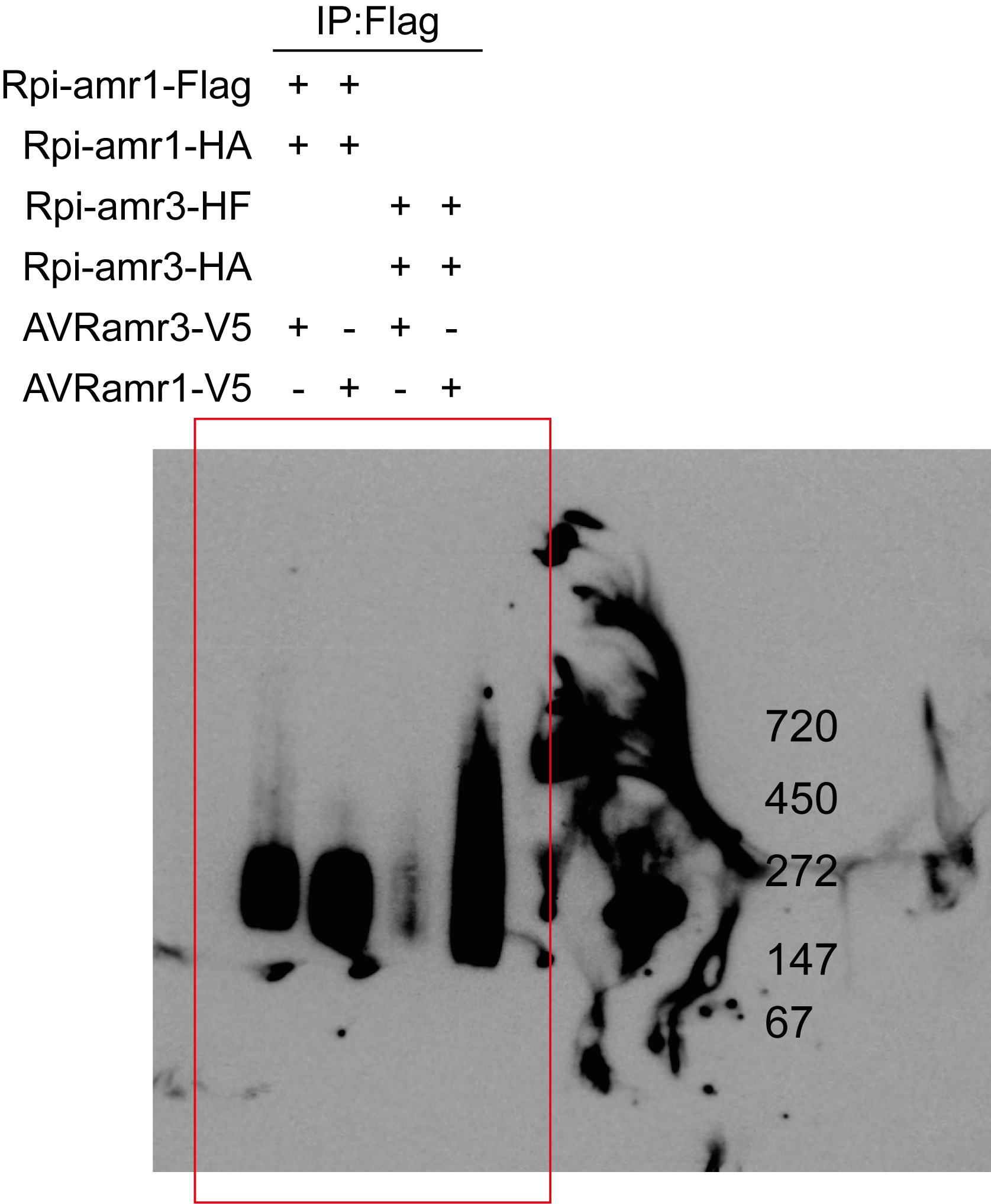

Supplement: Supplementary file 4 — Source Data for Expanded View [file EMBJ-42-e111484-s002.zip › EMBOJ-2022-11484_SourceData/Figure EV3/EV3C/BNP Western Flag HIGH_annotation.tif]

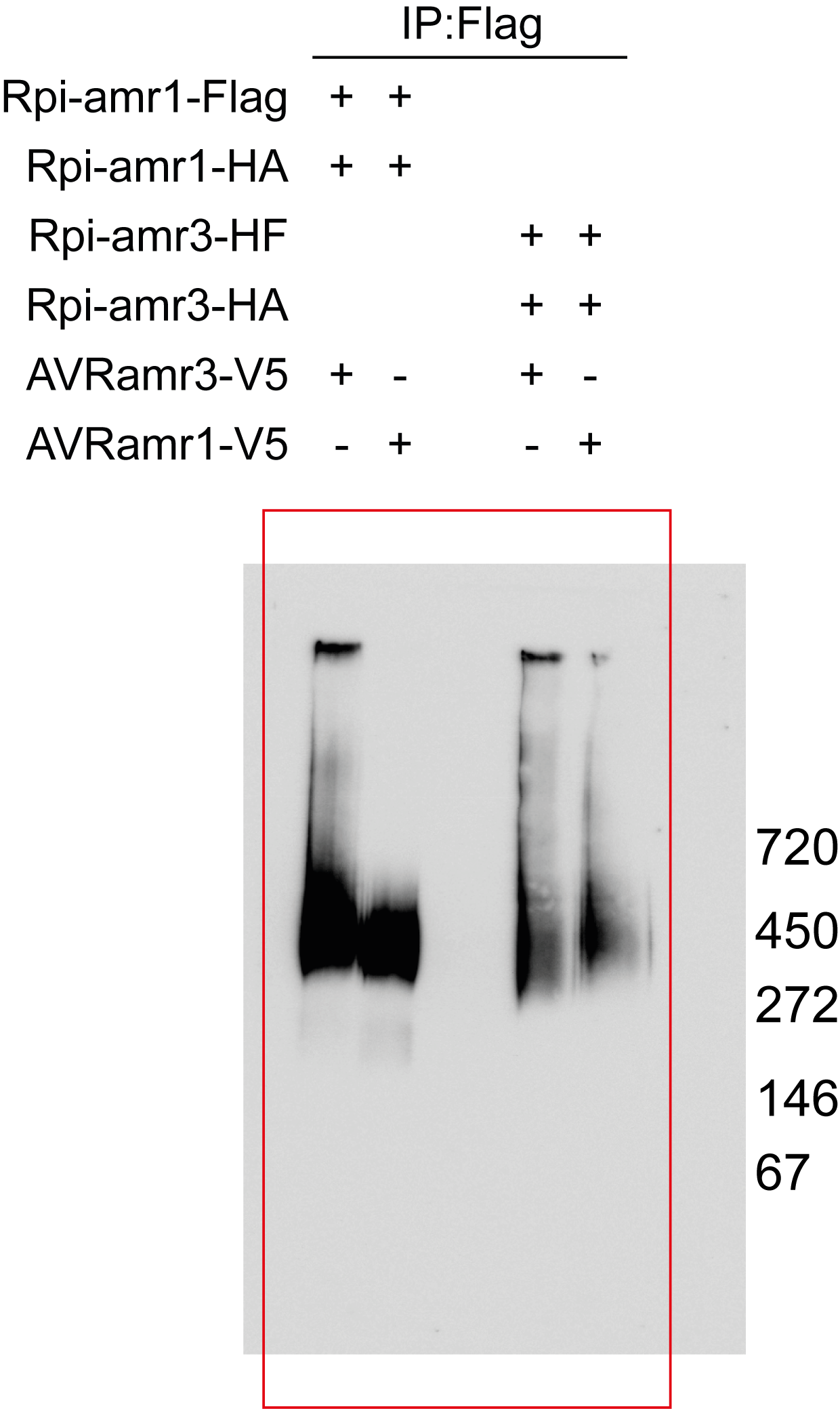

Supplement: Supplementary file 4 — Source Data for Expanded View [file EMBJ-42-e111484-s002.zip › EMBOJ-2022-11484_SourceData/Figure EV3/EV3D/BNP Western HA_annotations.tif]

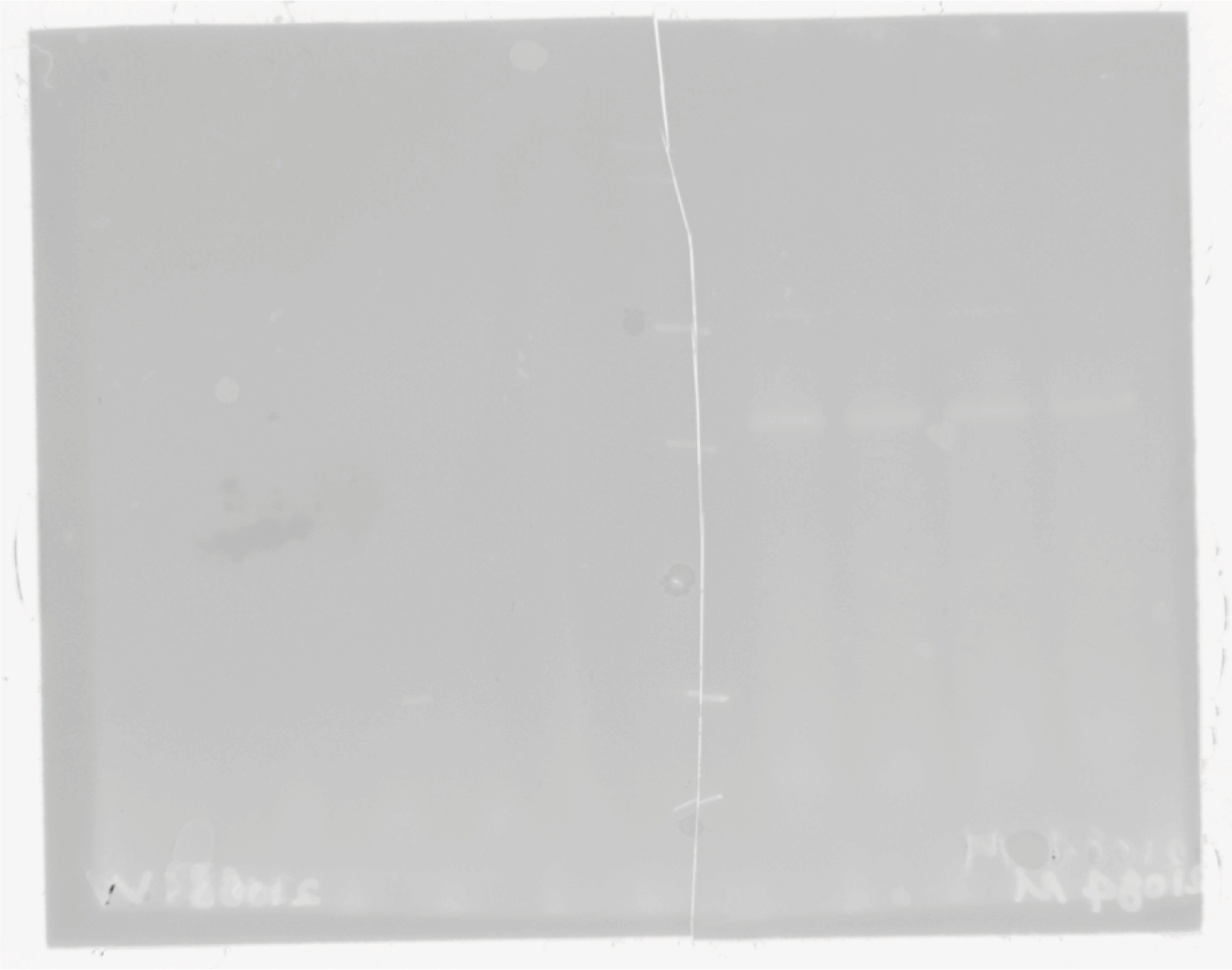

Supplement: Supplementary file 4 — Source Data for Expanded View [file EMBJ-42-e111484-s002.zip › EMBOJ-2022-11484_SourceData/Figure EV3/EV3E/BNP Western V5 Marker.tif]

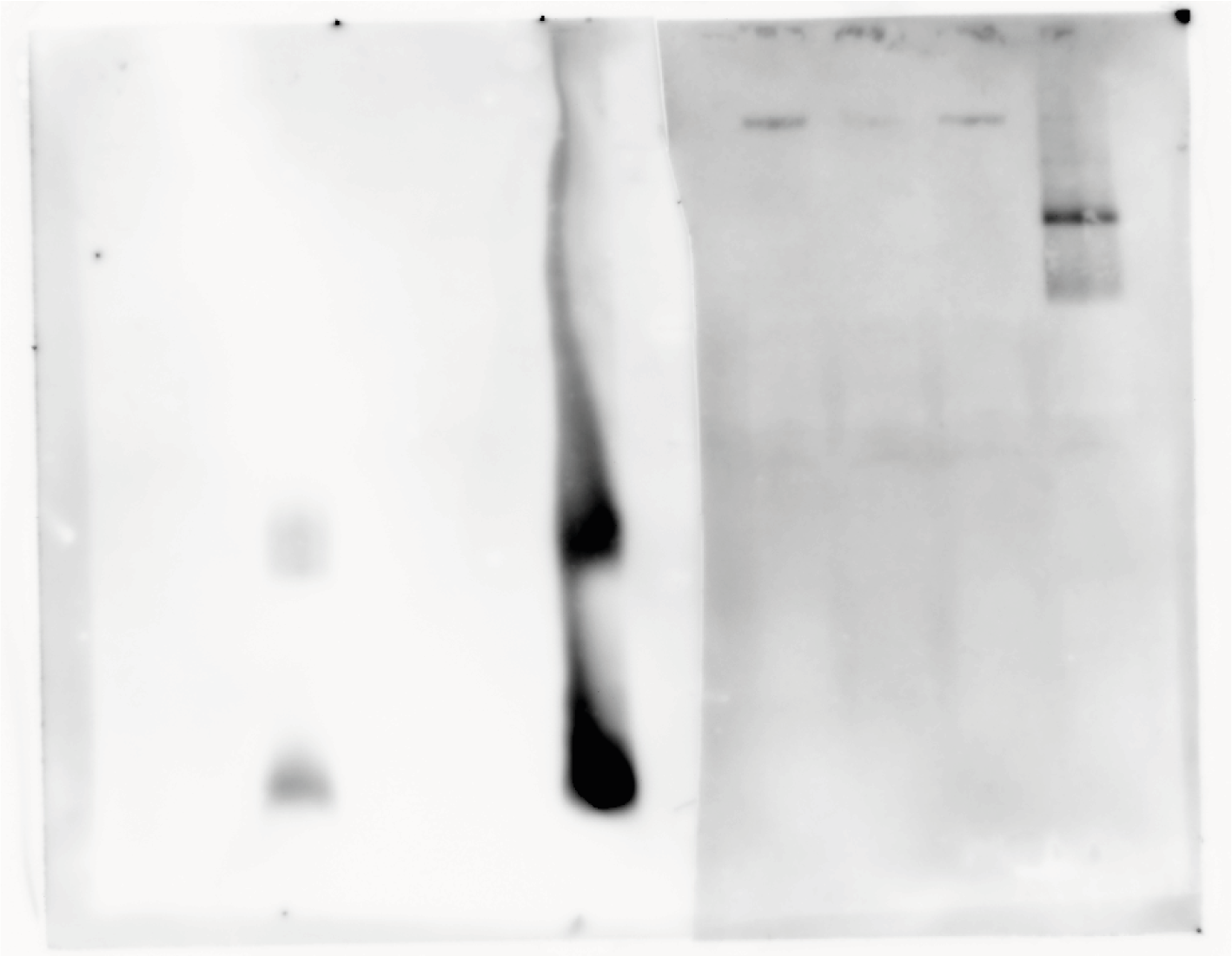

Supplement: Supplementary file 4 — Source Data for Expanded View [file EMBJ-42-e111484-s002.zip › EMBOJ-2022-11484_SourceData/Figure EV3/EV3E/BNP Western V5.tif]

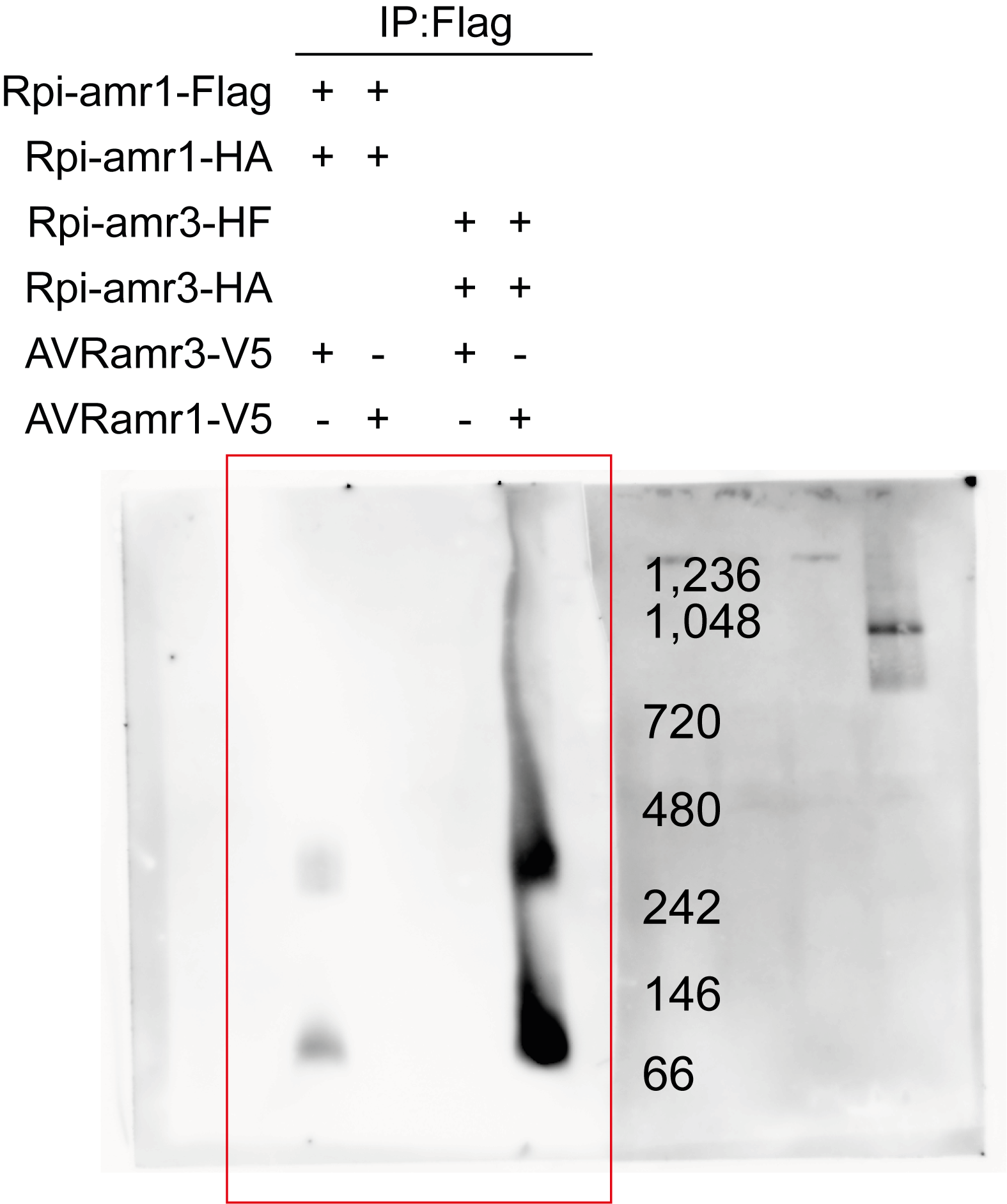

Supplement: Supplementary file 4 — Source Data for Expanded View [file EMBJ-42-e111484-s002.zip › EMBOJ-2022-11484_SourceData/Figure EV3/EV3E/BNP Western V5_annotations.tif]

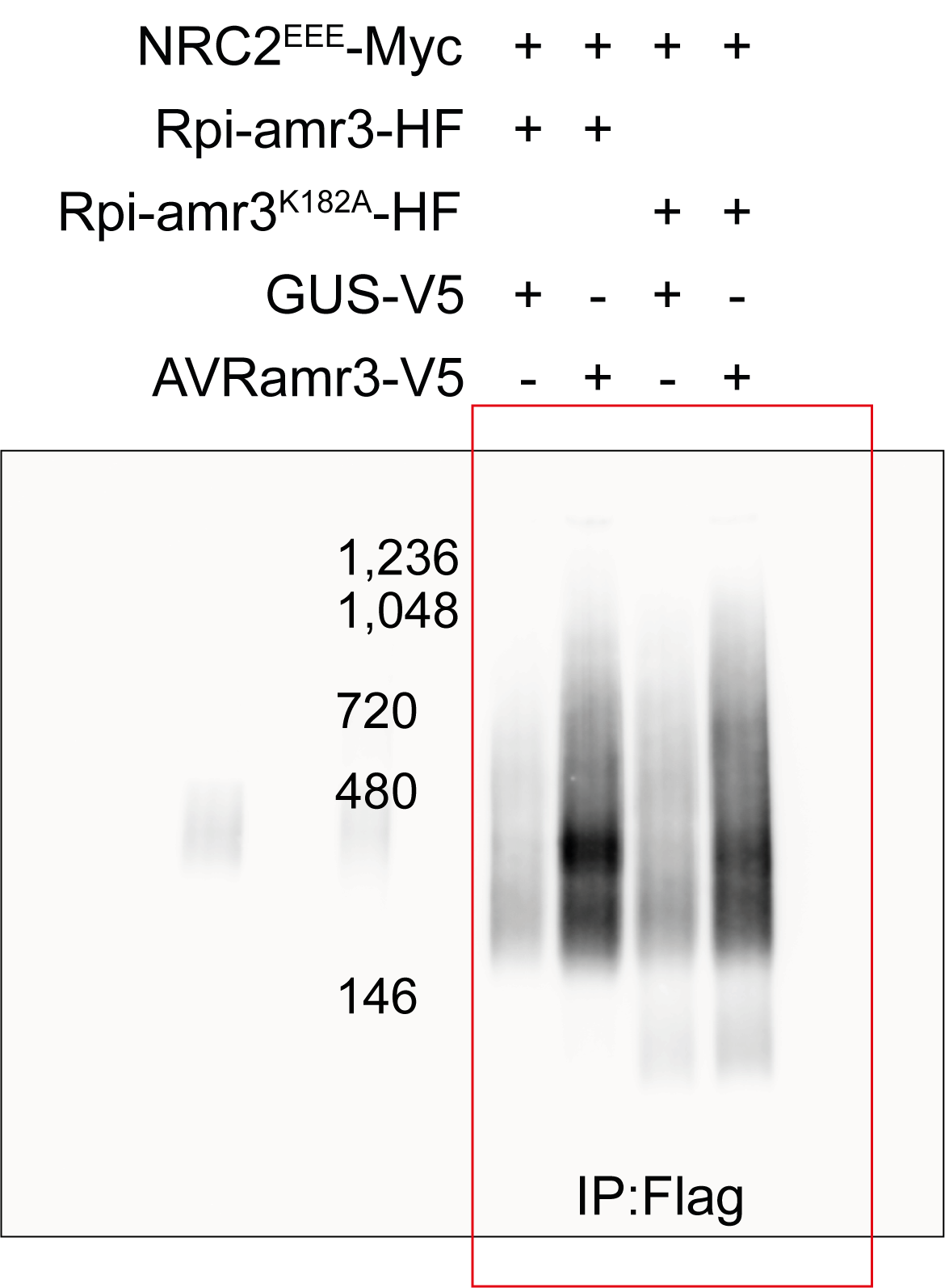

Supplement: Supplementary file 4 — Source Data for Expanded View [file EMBJ-42-e111484-s002.zip › EMBOJ-2022-11484_SourceData/Figure EV4/EV4A/BNP Western Flag_annotations.tif]

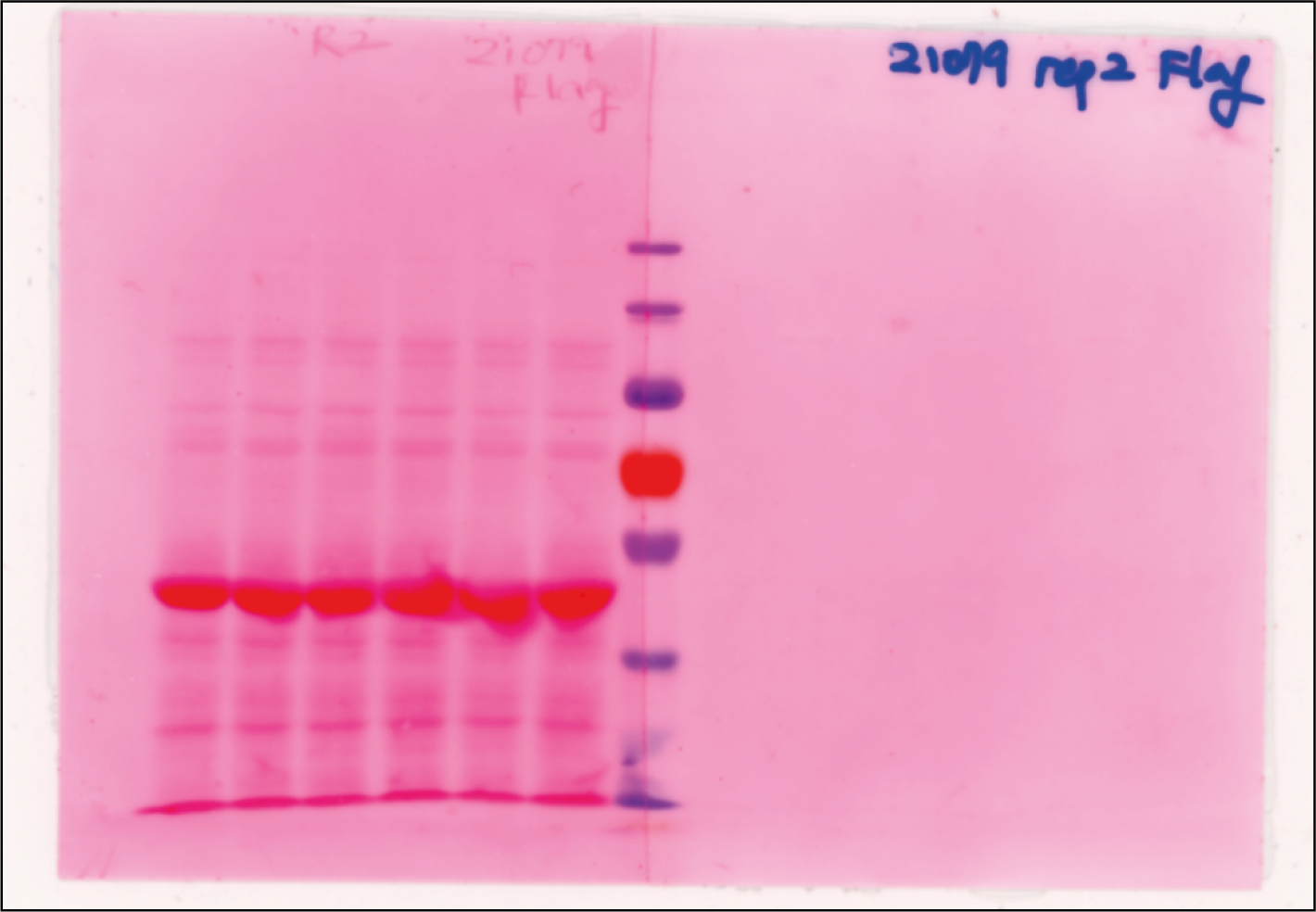

Supplement: Supplementary file 4 — Source Data for Expanded View [file EMBJ-42-e111484-s002.zip › EMBOJ-2022-11484_SourceData/Figure EV4/EV4A/SDS Western Ponceau.tif]

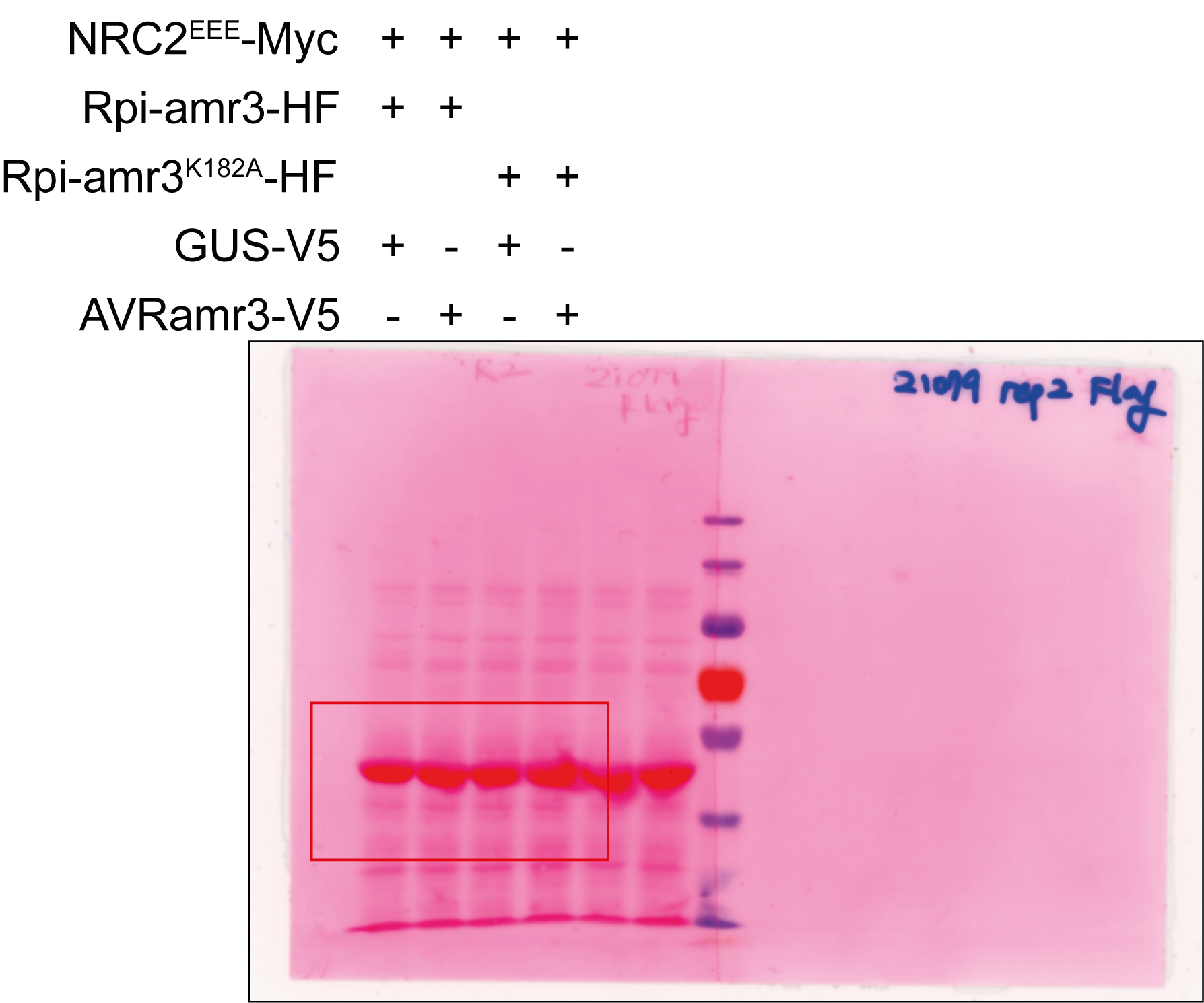

Supplement: Supplementary file 4 — Source Data for Expanded View [file EMBJ-42-e111484-s002.zip › EMBOJ-2022-11484_SourceData/Figure EV4/EV4A/SDS Western Ponceau_annotations.tif]

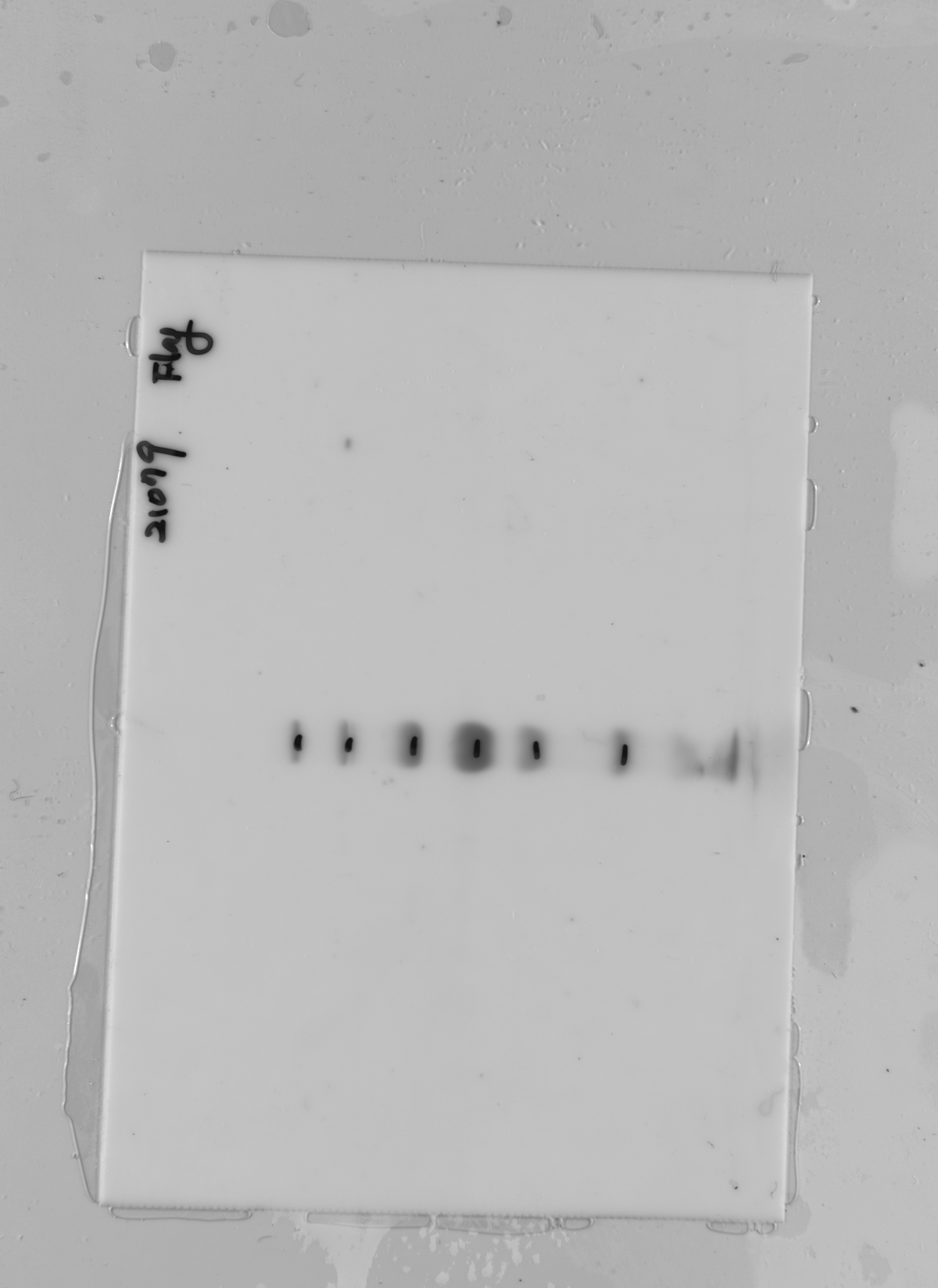

Supplement: Supplementary file 4 — Source Data for Expanded View [file EMBJ-42-e111484-s002.zip › EMBOJ-2022-11484_SourceData/Figure EV4/EV4A/Western Flag IP Marker.tif]

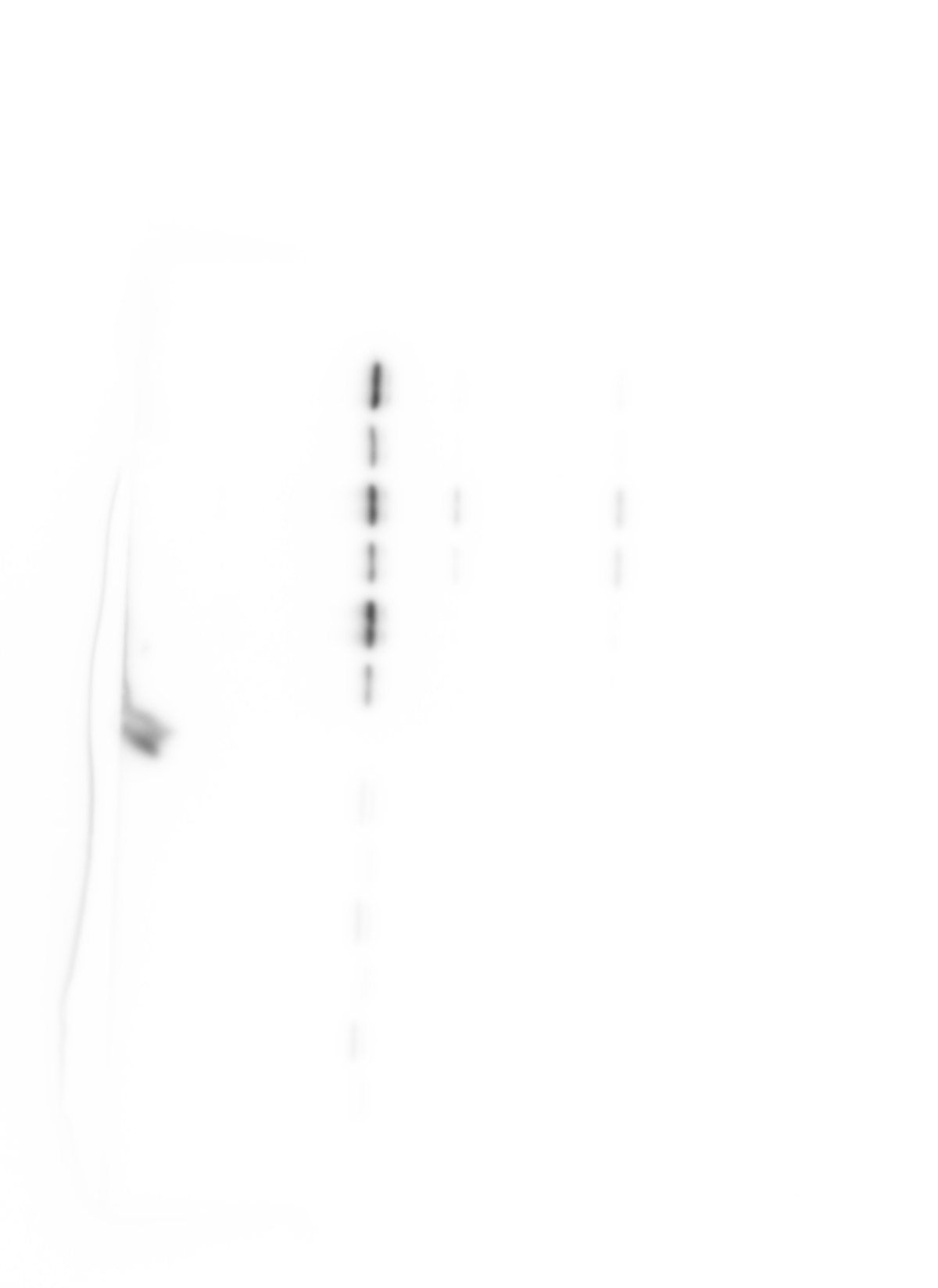

Supplement: Supplementary file 4 — Source Data for Expanded View [file EMBJ-42-e111484-s002.zip › EMBOJ-2022-11484_SourceData/Figure EV4/EV4A/Western Flag IP.tif]

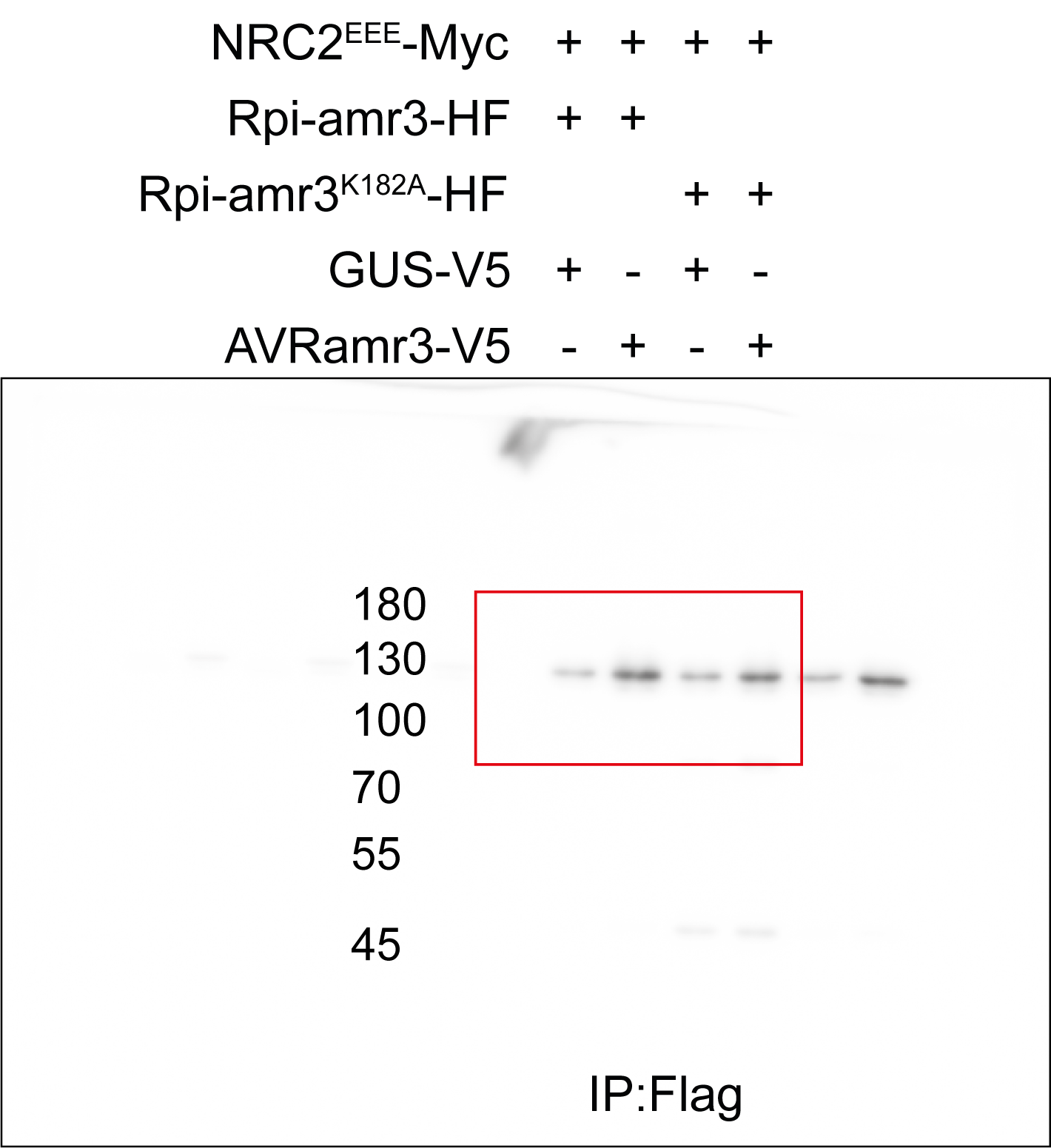

Supplement: Supplementary file 4 — Source Data for Expanded View [file EMBJ-42-e111484-s002.zip › EMBOJ-2022-11484_SourceData/Figure EV4/EV4A/Western Flag IP_annotations.tif]

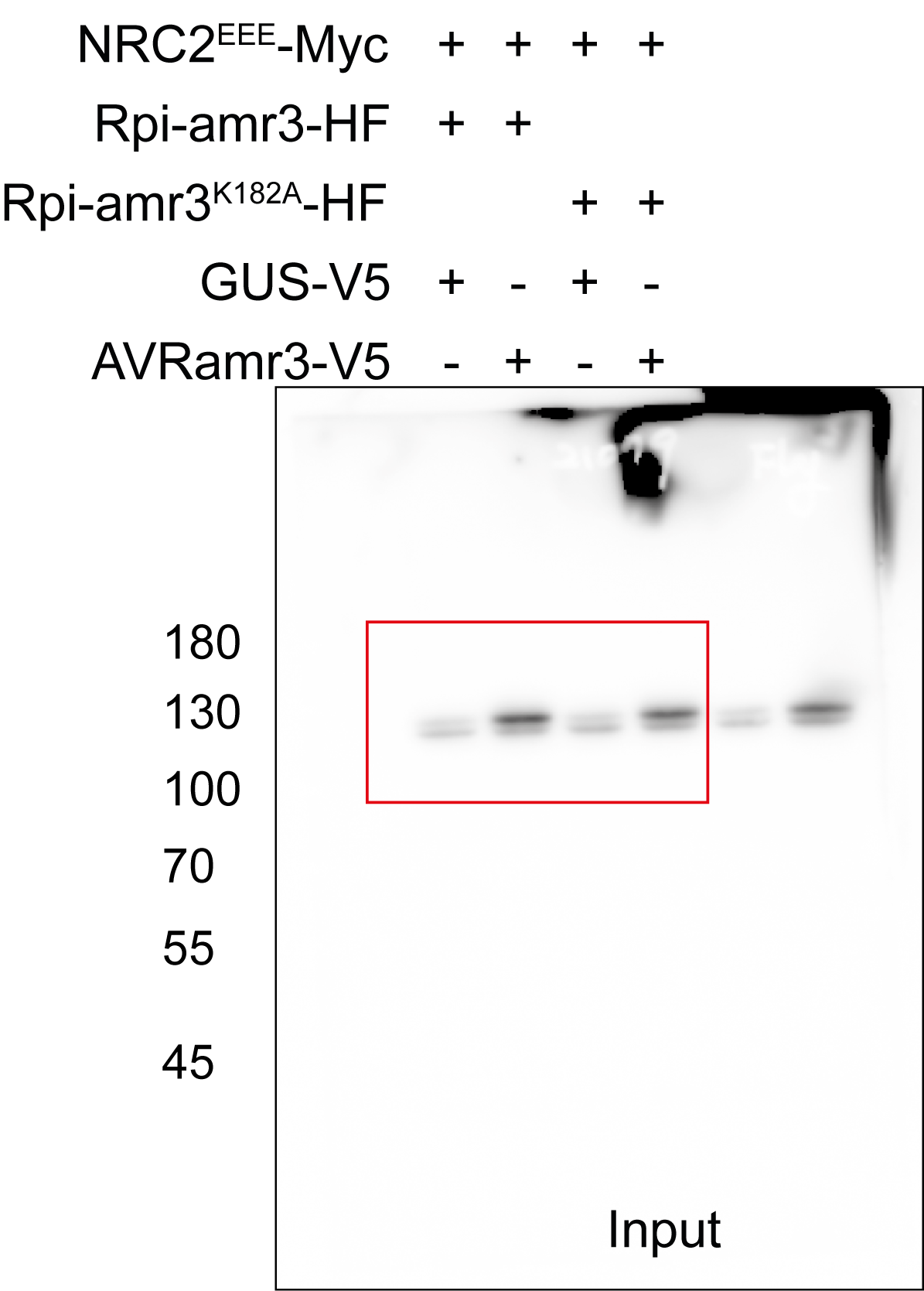

Supplement: Supplementary file 4 — Source Data for Expanded View [file EMBJ-42-e111484-s002.zip › EMBOJ-2022-11484_SourceData/Figure EV4/EV4A/Western Flag Input_annotations.tif]

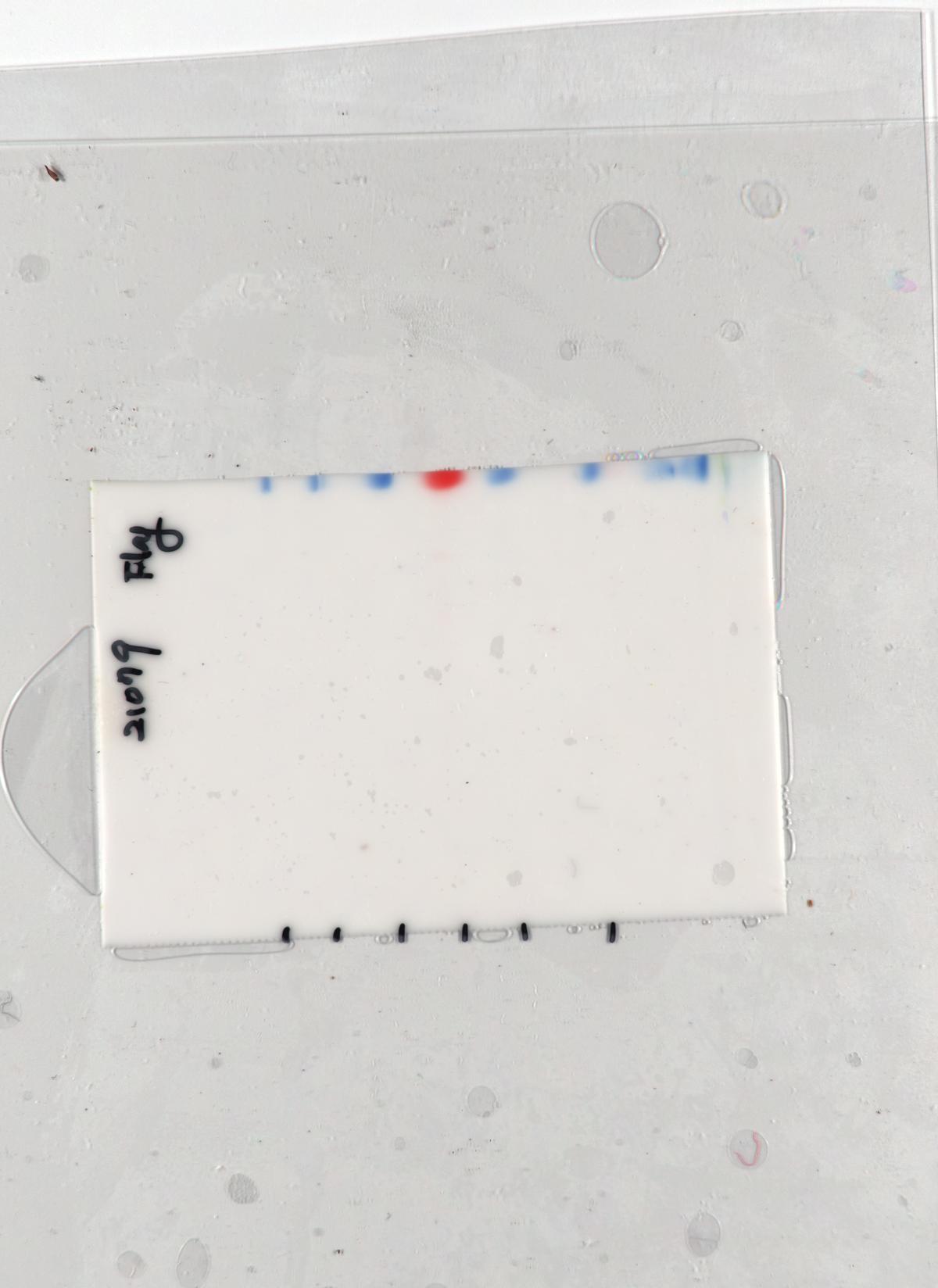

Supplement: Supplementary file 4 — Source Data for Expanded View [file EMBJ-42-e111484-s002.zip › EMBOJ-2022-11484_SourceData/Figure EV4/EV4A/Western Flag input Marker.jpg]

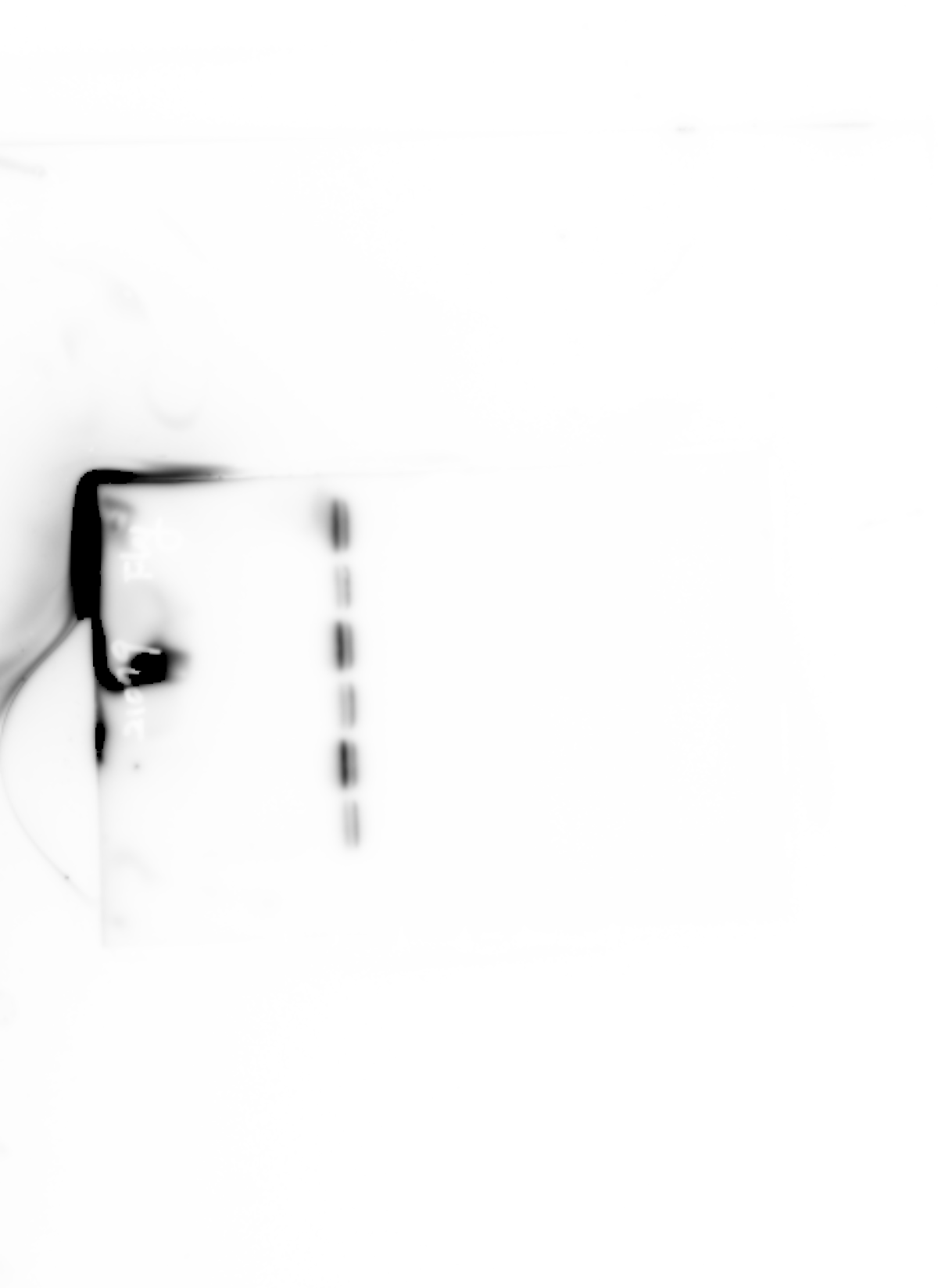

Supplement: Supplementary file 4 — Source Data for Expanded View [file EMBJ-42-e111484-s002.zip › EMBOJ-2022-11484_SourceData/Figure EV4/EV4A/Western Flag input.tif]

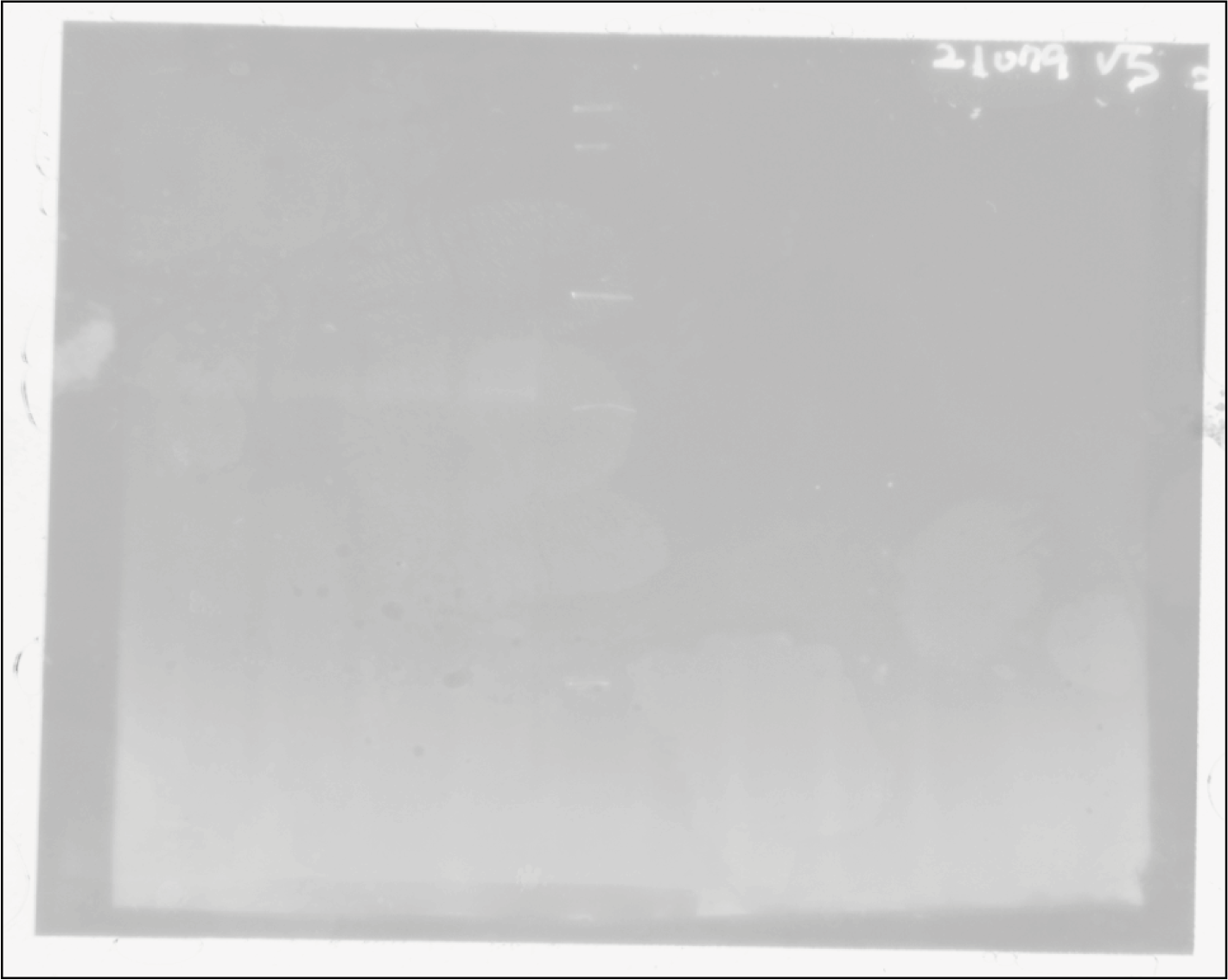

Supplement: Supplementary file 4 — Source Data for Expanded View [file EMBJ-42-e111484-s002.zip › EMBOJ-2022-11484_SourceData/Figure EV4/EV4B/BNP Western V5 Marker.tif]

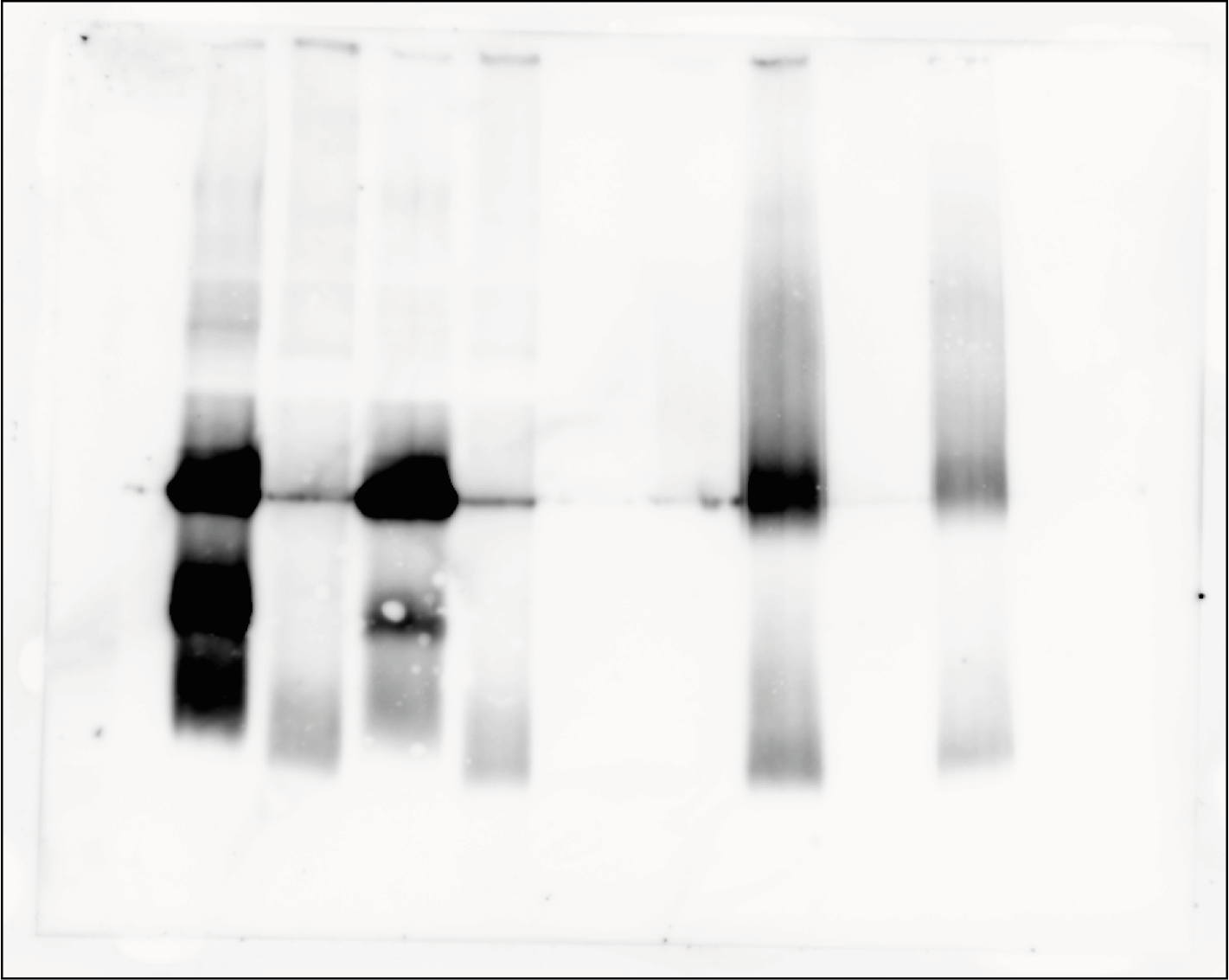

Supplement: Supplementary file 4 — Source Data for Expanded View [file EMBJ-42-e111484-s002.zip › EMBOJ-2022-11484_SourceData/Figure EV4/EV4B/BNP Western V5.tif]

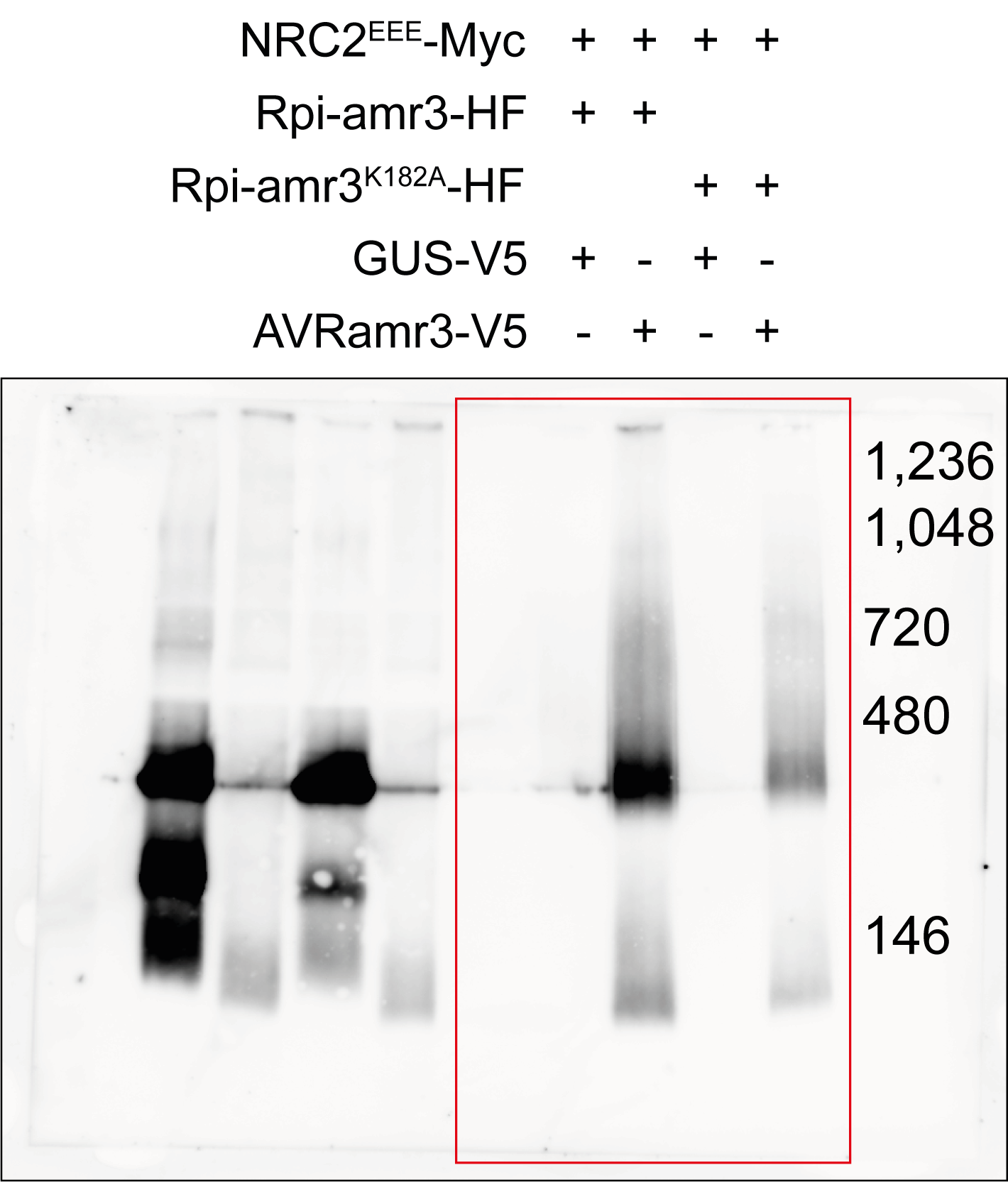

Supplement: Supplementary file 4 — Source Data for Expanded View [file EMBJ-42-e111484-s002.zip › EMBOJ-2022-11484_SourceData/Figure EV4/EV4B/BNP Western V5_annotations.tif]

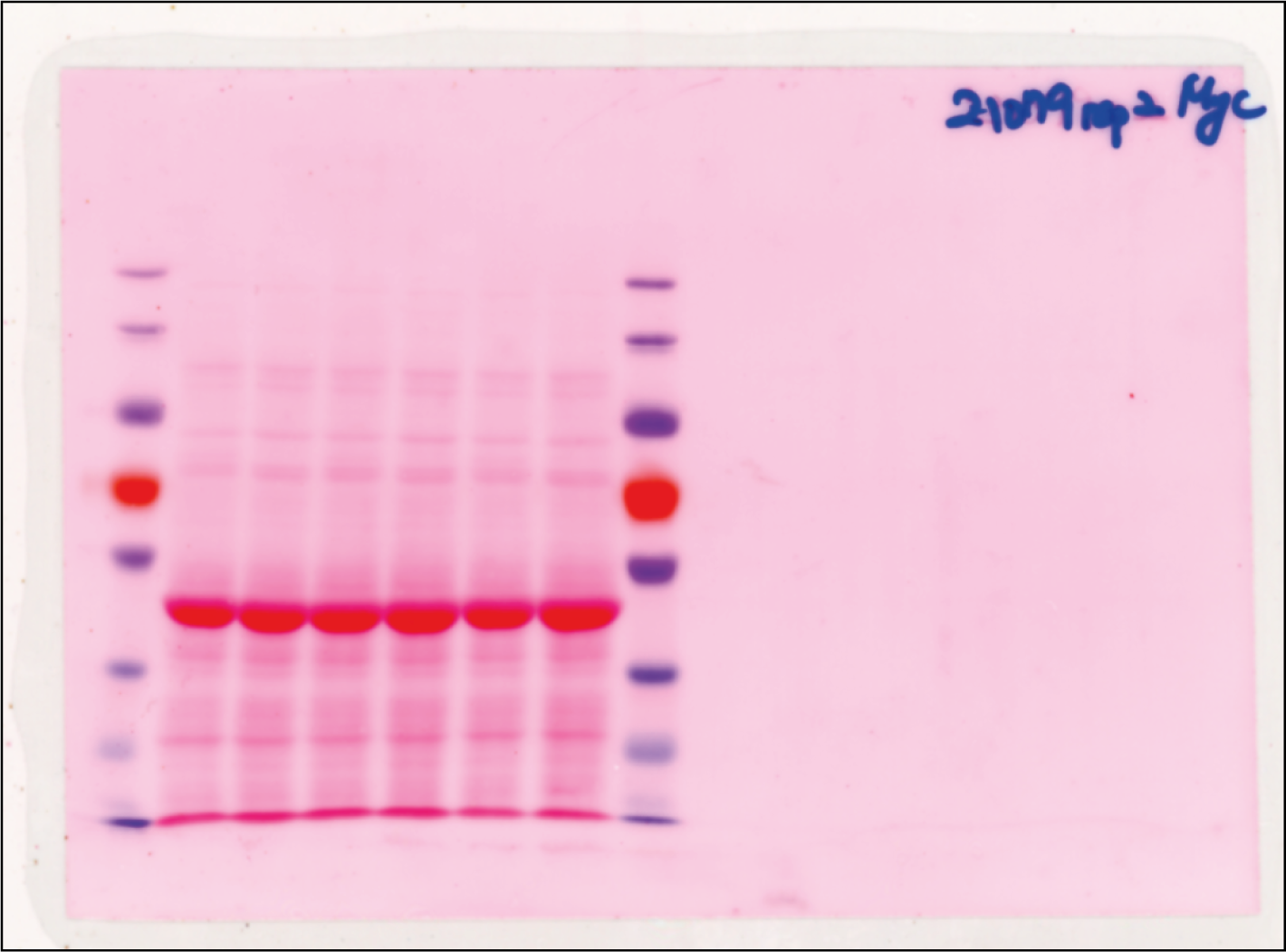

Supplement: Supplementary file 4 — Source Data for Expanded View [file EMBJ-42-e111484-s002.zip › EMBOJ-2022-11484_SourceData/Figure EV4/EV4B/SDS Western Ponceau.tif]

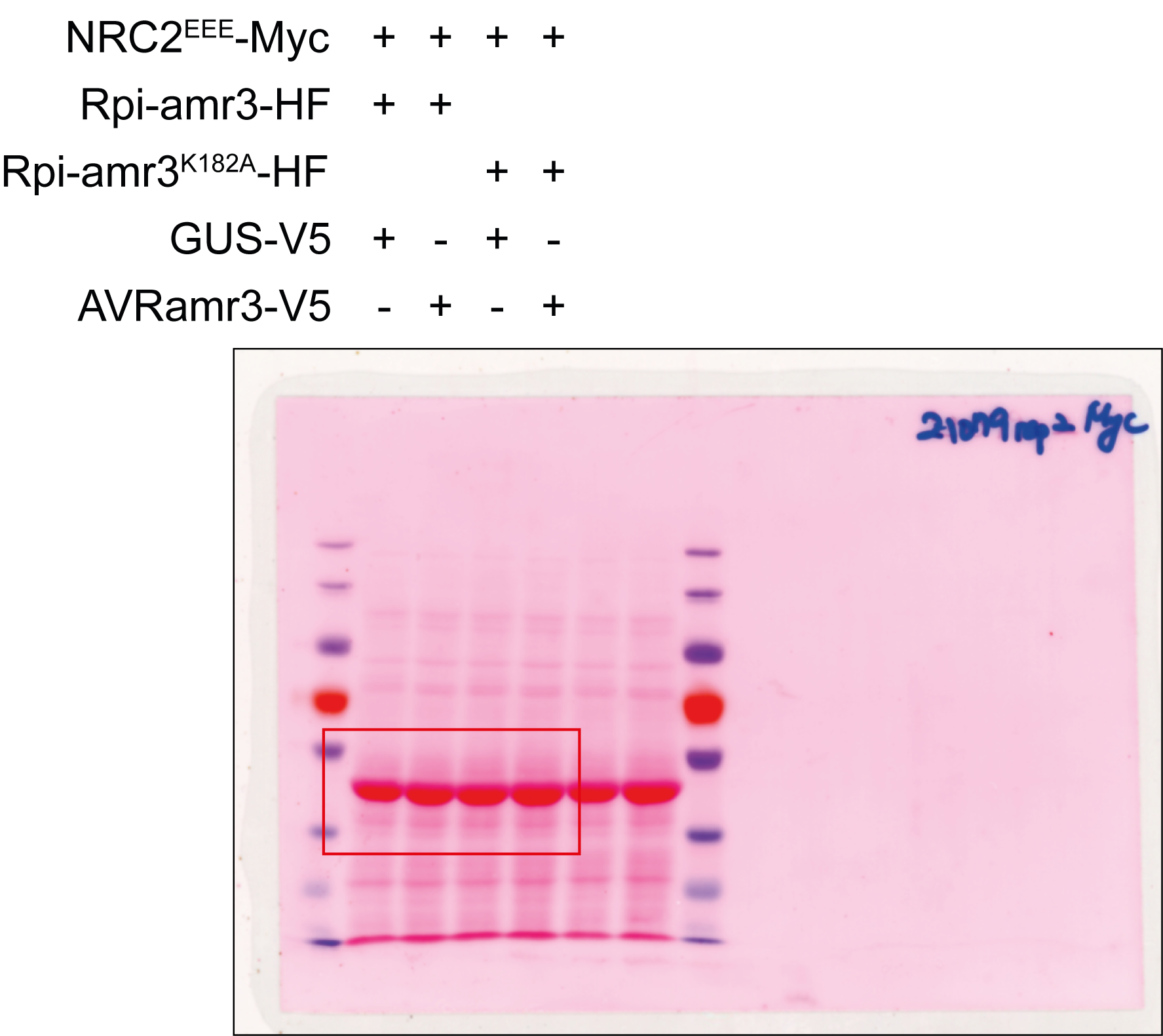

Supplement: Supplementary file 4 — Source Data for Expanded View [file EMBJ-42-e111484-s002.zip › EMBOJ-2022-11484_SourceData/Figure EV4/EV4B/SDS Western Ponceau_annotation.tif]

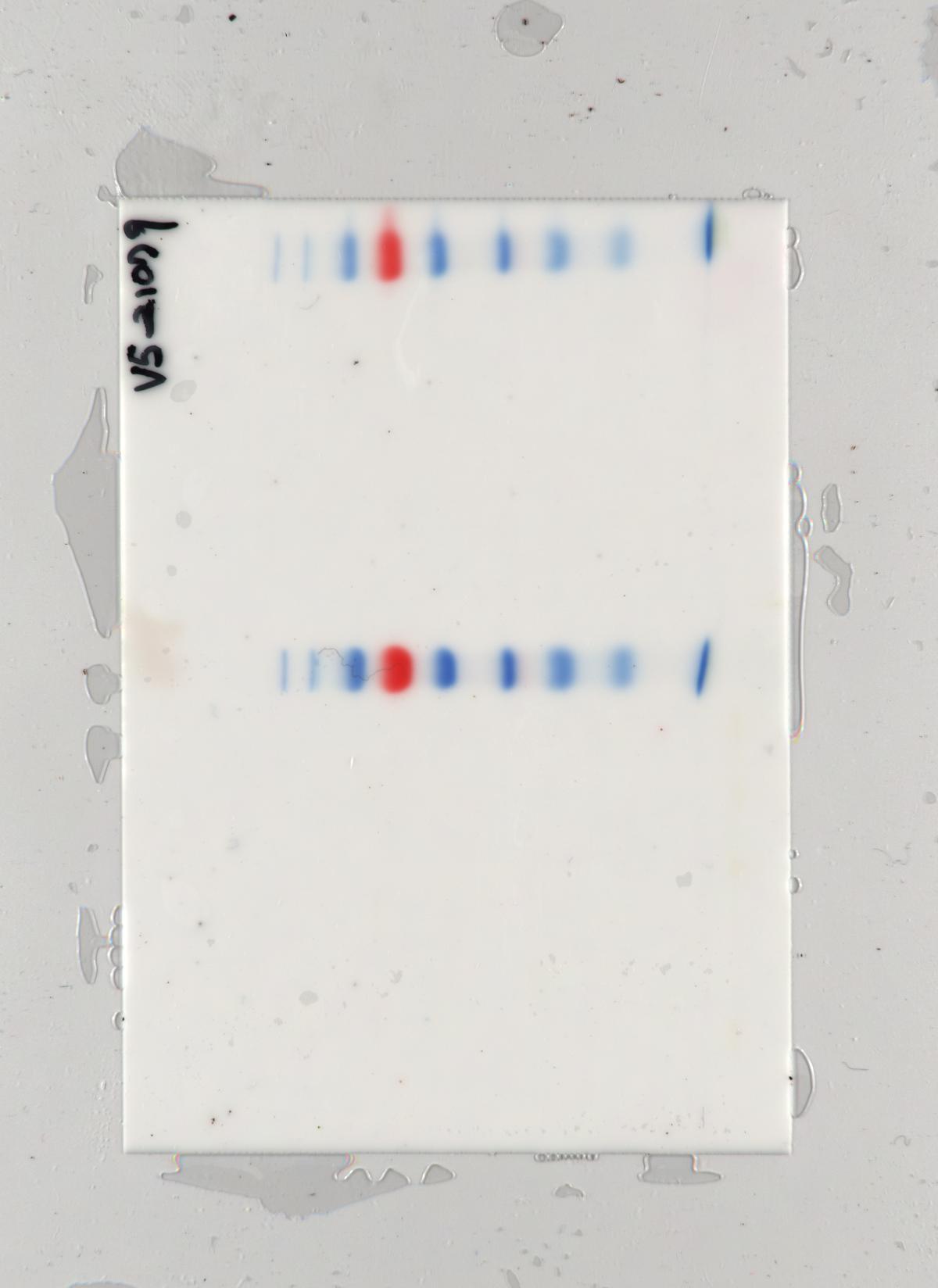

Supplement: Supplementary file 4 — Source Data for Expanded View [file EMBJ-42-e111484-s002.zip › EMBOJ-2022-11484_SourceData/Figure EV4/EV4B/SDS Western V5 Marker.jpg]

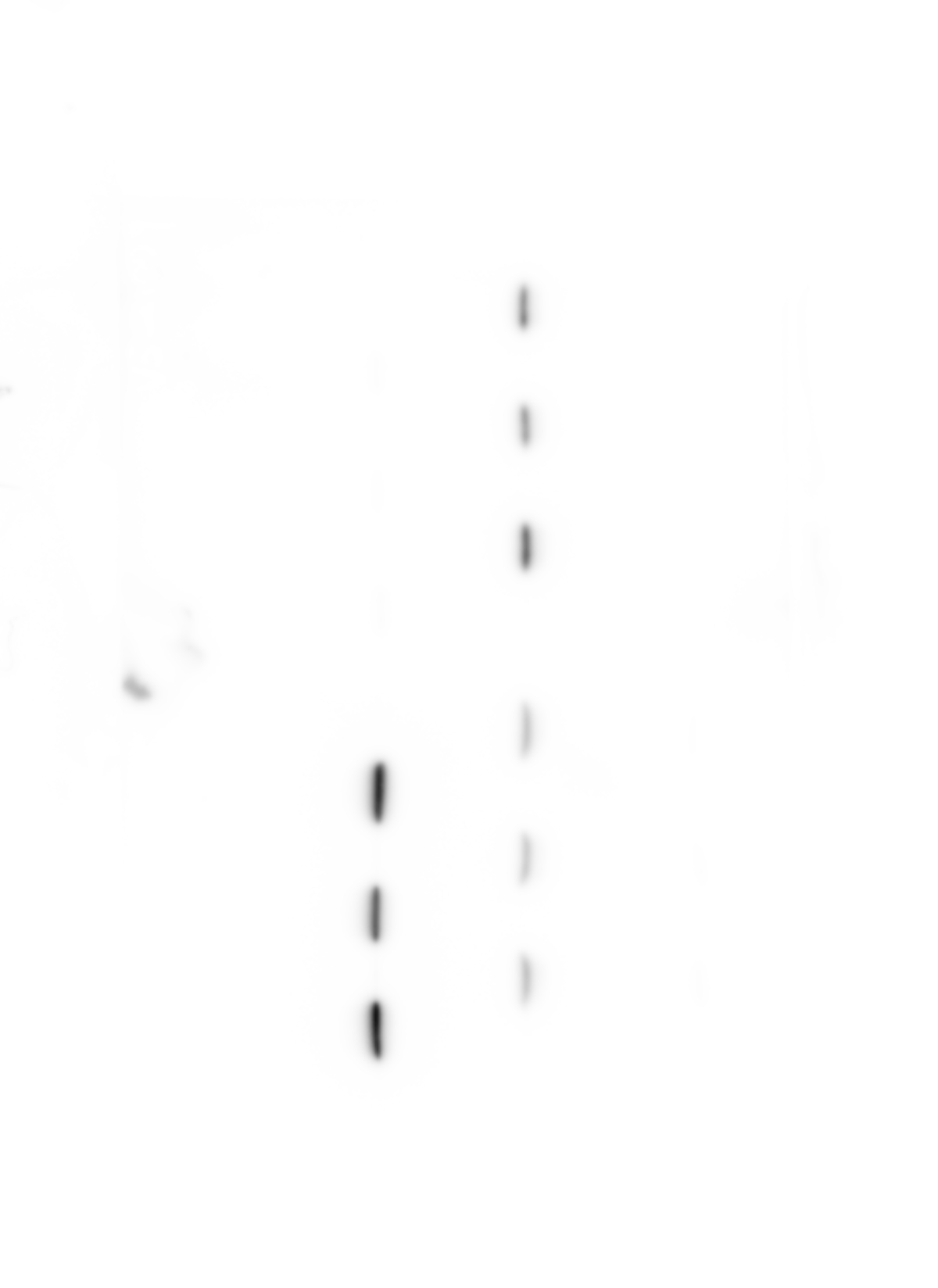

Supplement: Supplementary file 4 — Source Data for Expanded View [file EMBJ-42-e111484-s002.zip › EMBOJ-2022-11484_SourceData/Figure EV4/EV4B/SDS Western V5.tif]

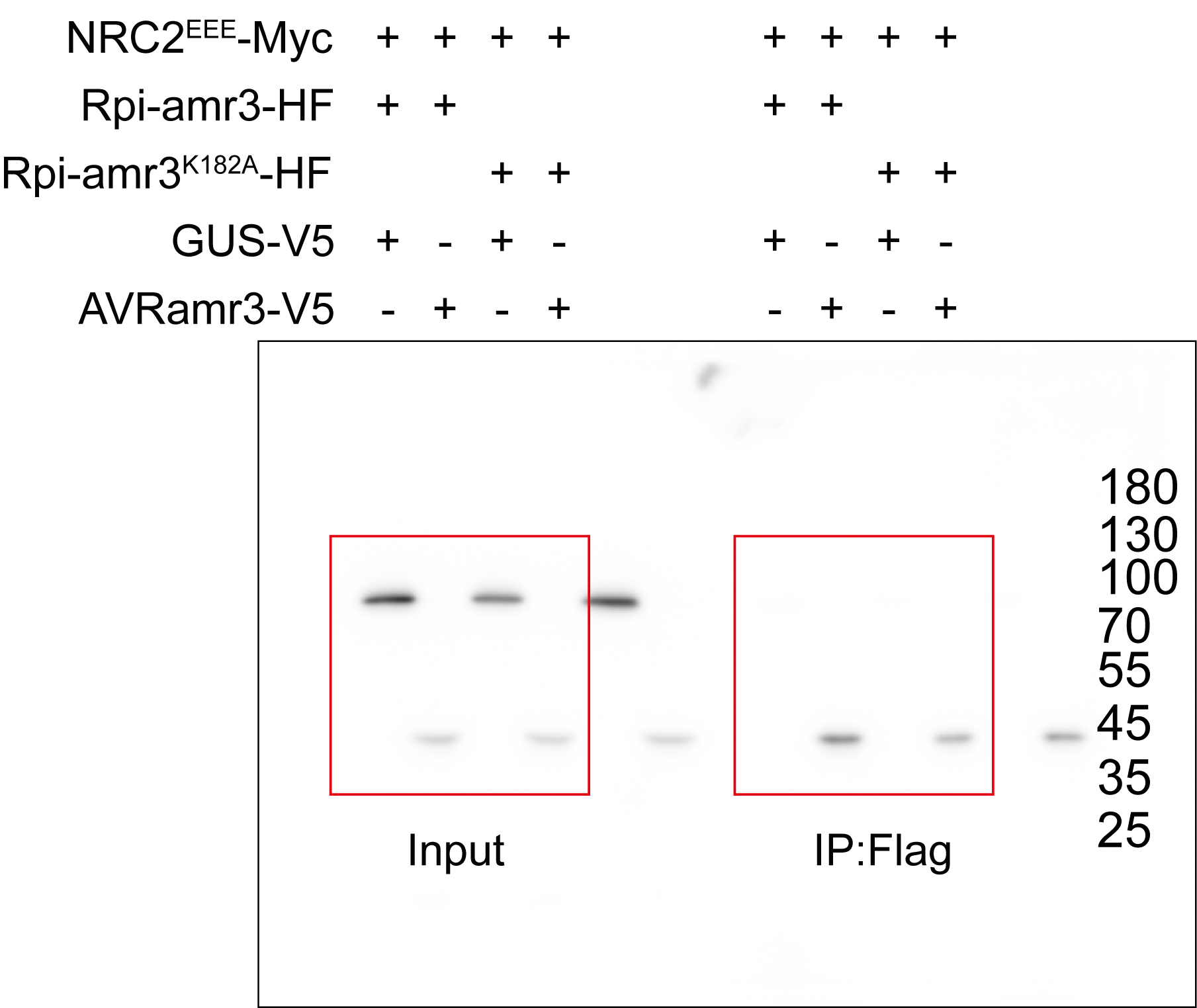

Supplement: Supplementary file 4 — Source Data for Expanded View [file EMBJ-42-e111484-s002.zip › EMBOJ-2022-11484_SourceData/Figure EV4/EV4B/SDS Western V5_annotations.tif]

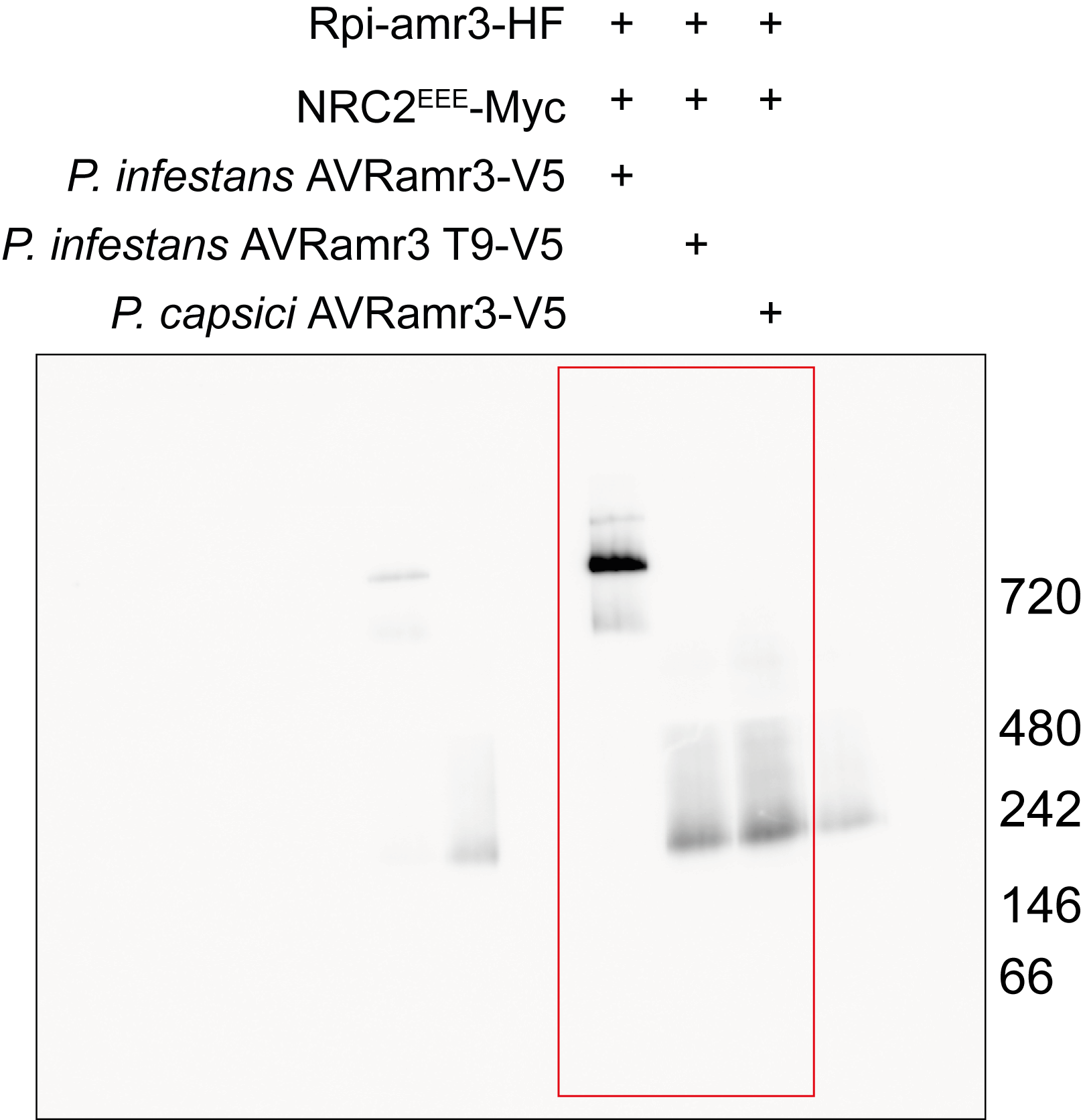

Supplement: Supplementary file 4 — Source Data for Expanded View [file EMBJ-42-e111484-s002.zip › EMBOJ-2022-11484_SourceData/Figure EV5/EV5B/BNP Western Myc_annotations.tif]

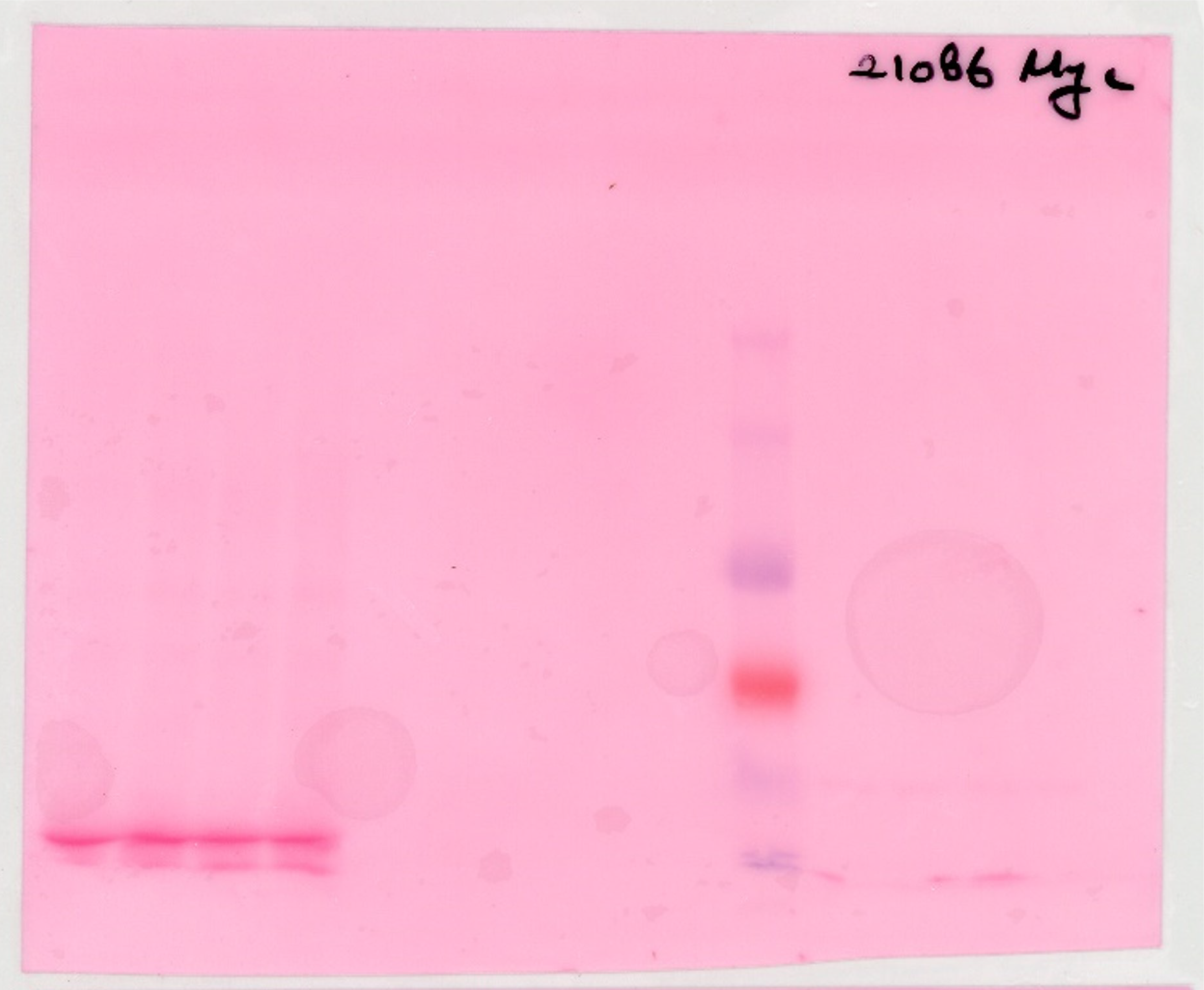

Supplement: Supplementary file 4 — Source Data for Expanded View [file EMBJ-42-e111484-s002.zip › EMBOJ-2022-11484_SourceData/Figure EV5/EV5B/SDS Ponceau.tif]

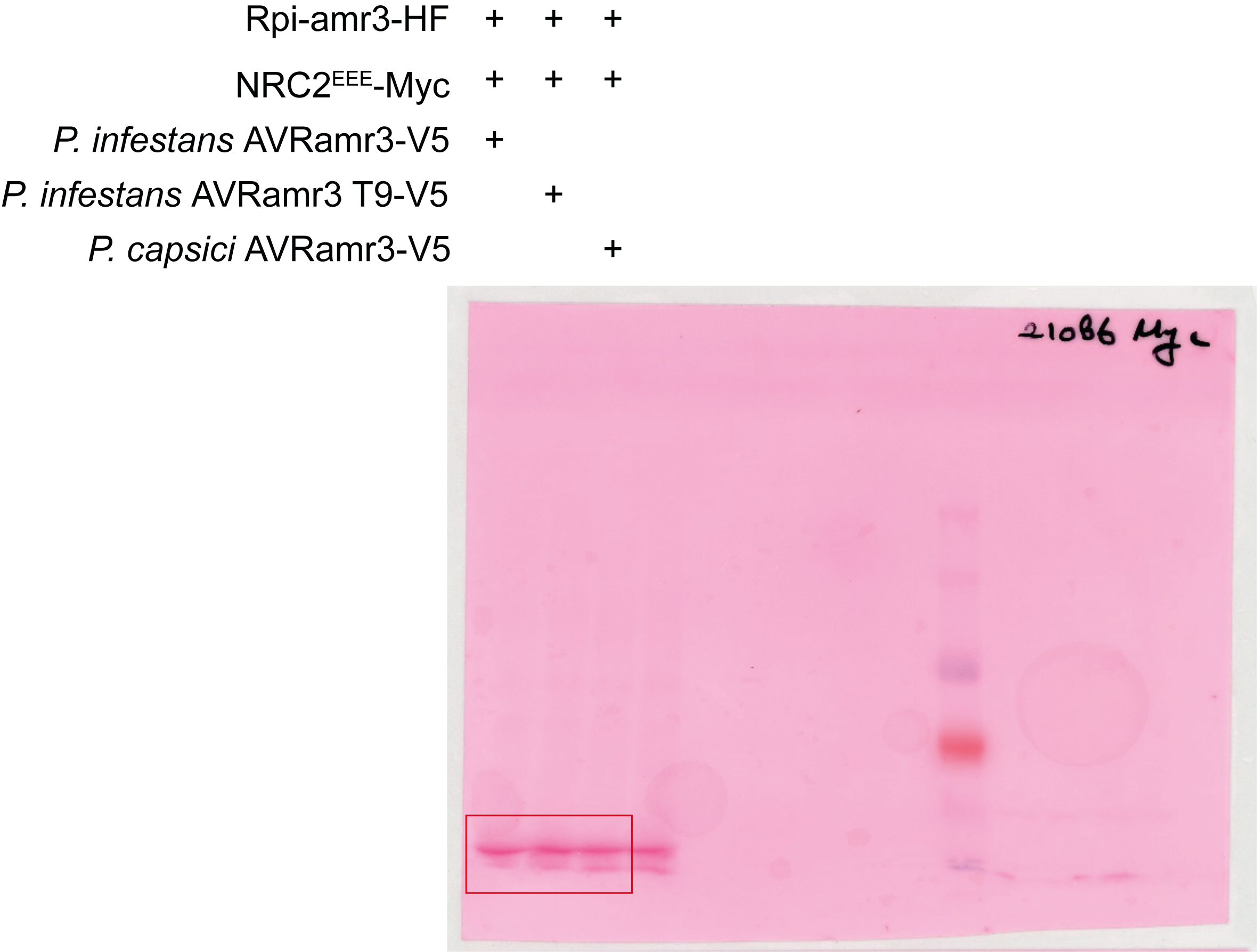

Supplement: Supplementary file 4 — Source Data for Expanded View [file EMBJ-42-e111484-s002.zip › EMBOJ-2022-11484_SourceData/Figure EV5/EV5B/SDS Ponceau_annotations.tif]

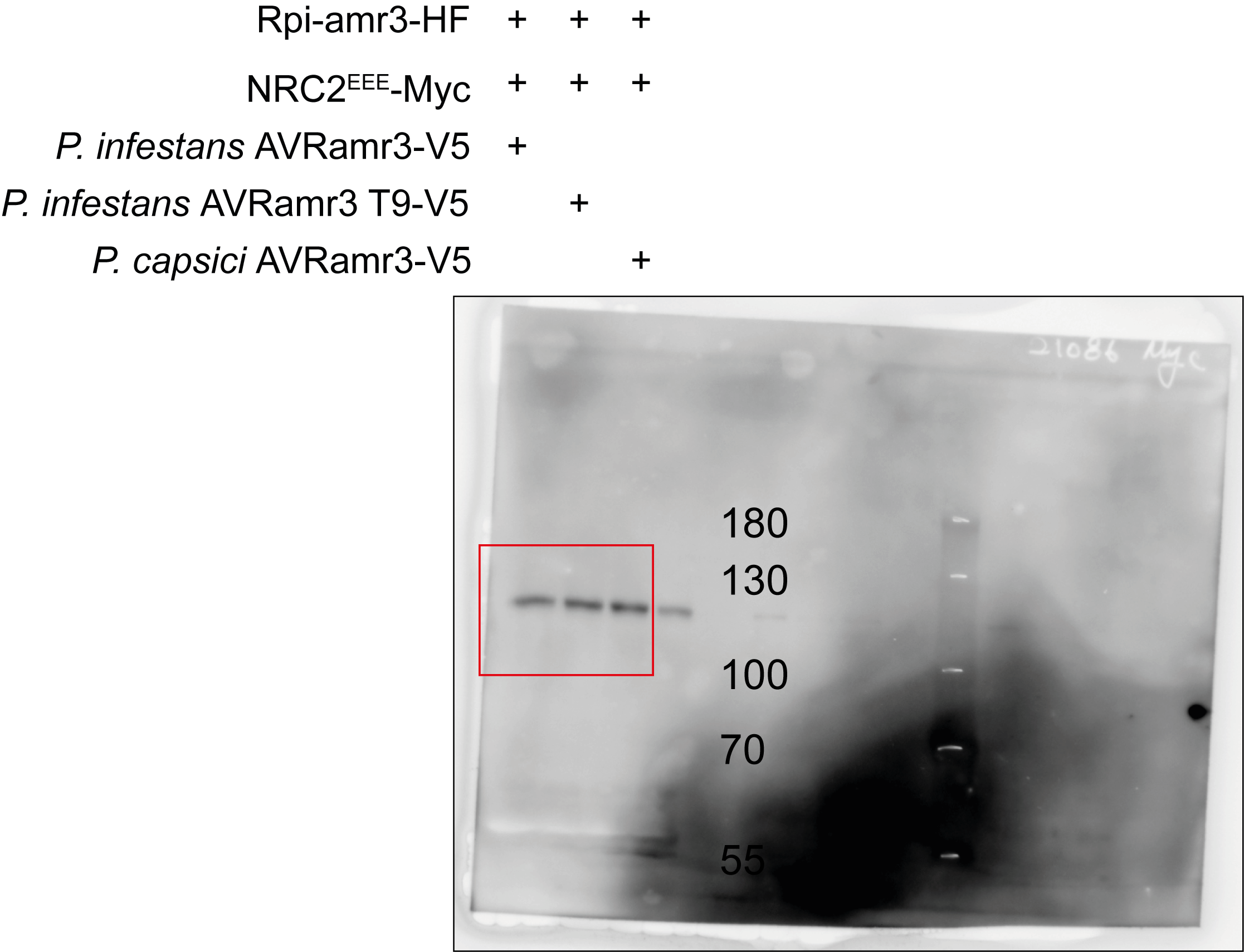

Supplement: Supplementary file 4 — Source Data for Expanded View [file EMBJ-42-e111484-s002.zip › EMBOJ-2022-11484_SourceData/Figure EV5/EV5B/SDS Western Myc_annotations.tif]

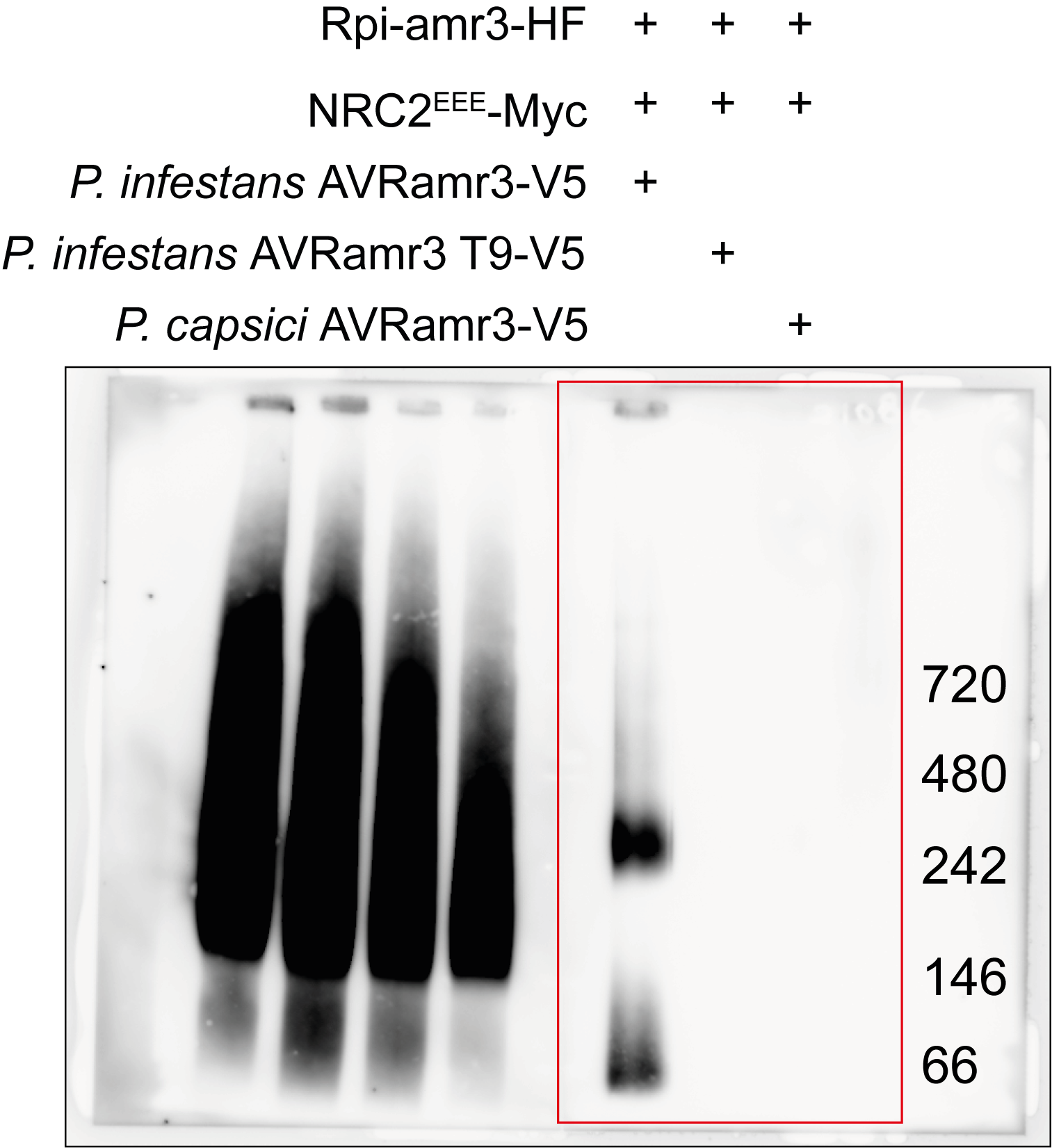

Supplement: Supplementary file 4 — Source Data for Expanded View [file EMBJ-42-e111484-s002.zip › EMBOJ-2022-11484_SourceData/Figure EV5/EV5C/BNP Western V5_annotations.tif]

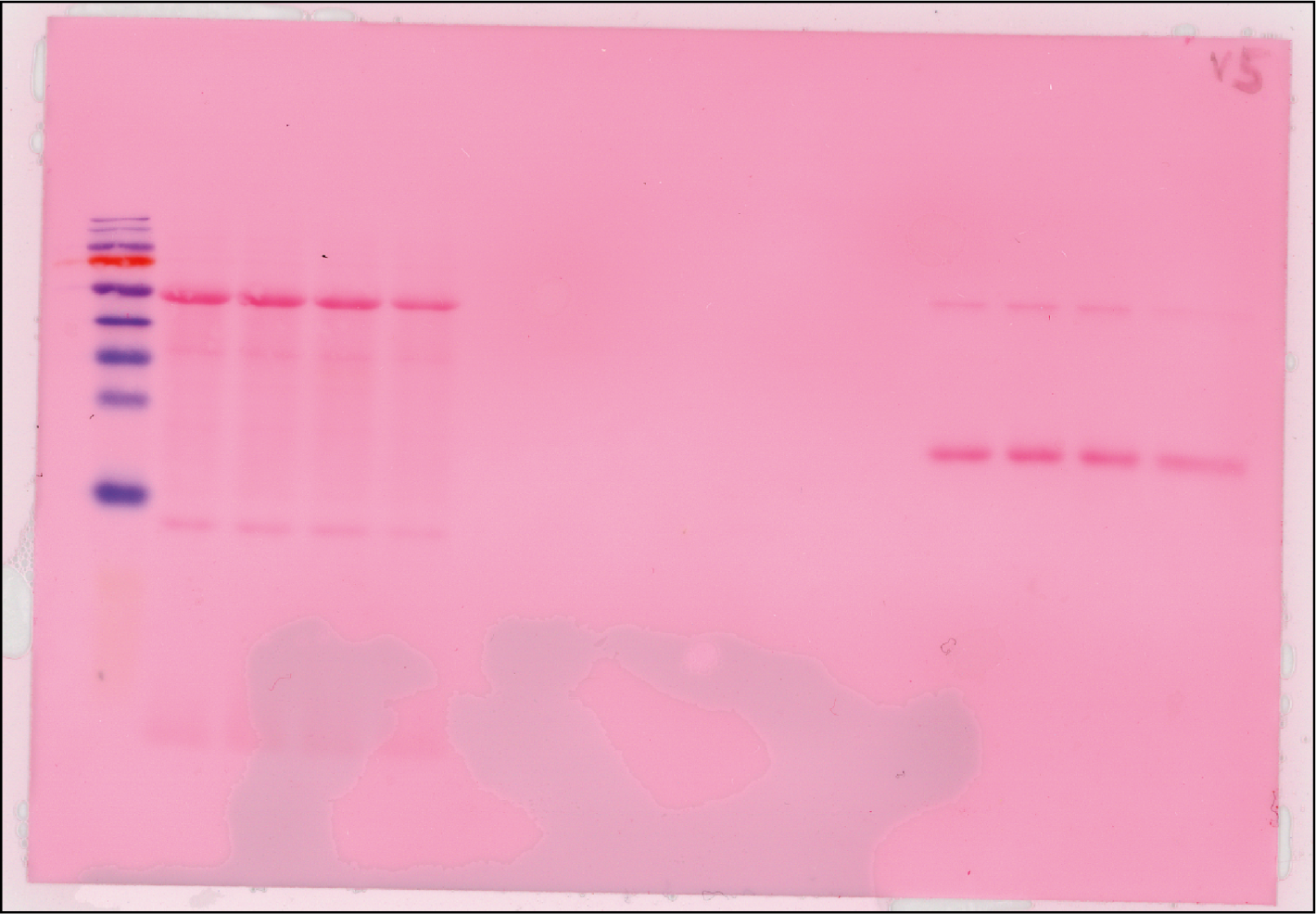

Supplement: Supplementary file 4 — Source Data for Expanded View [file EMBJ-42-e111484-s002.zip › EMBOJ-2022-11484_SourceData/Figure EV5/EV5C/SDS Ponceau.tif]

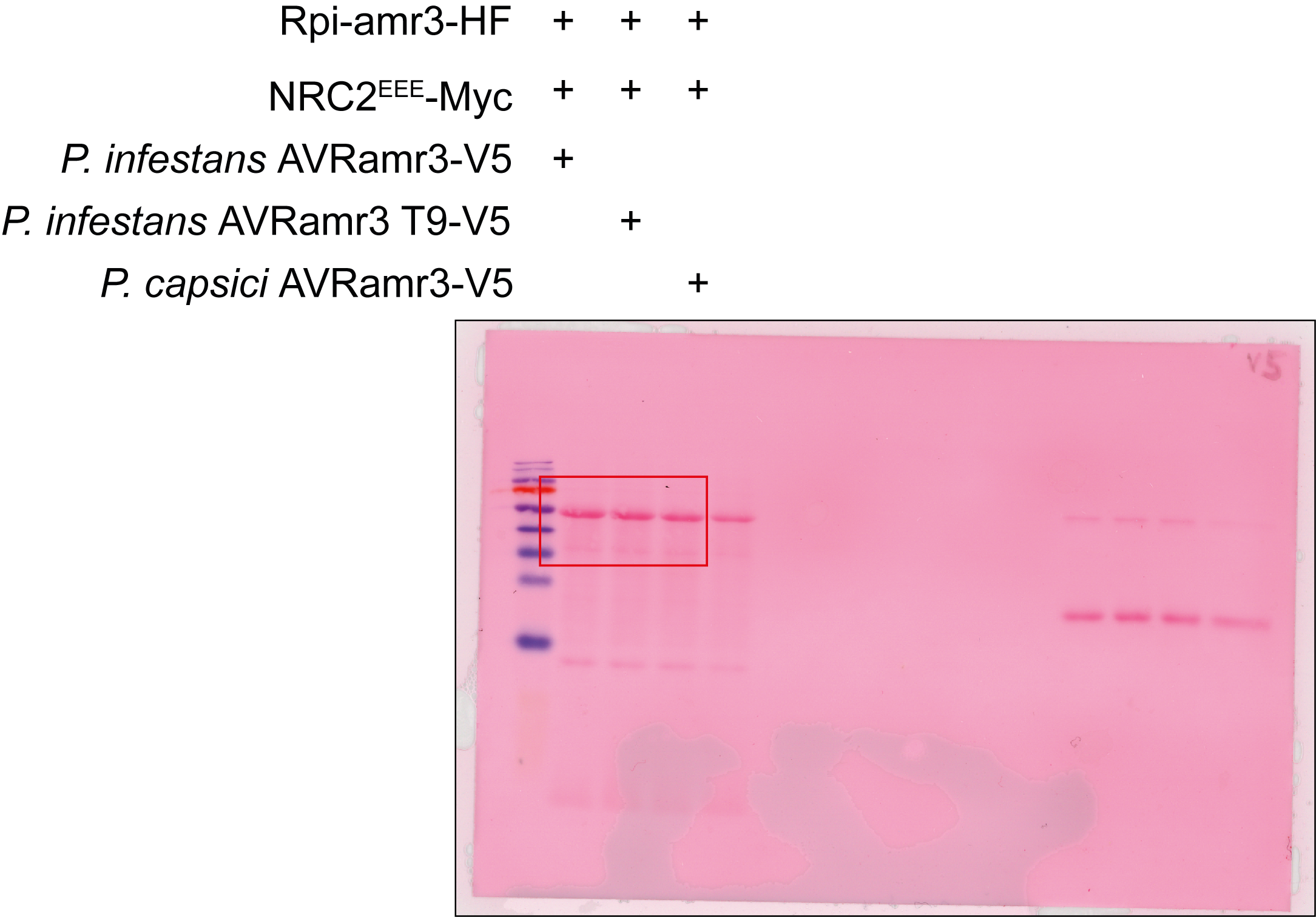

Supplement: Supplementary file 4 — Source Data for Expanded View [file EMBJ-42-e111484-s002.zip › EMBOJ-2022-11484_SourceData/Figure EV5/EV5C/SDS Ponceau_annotations.tif]

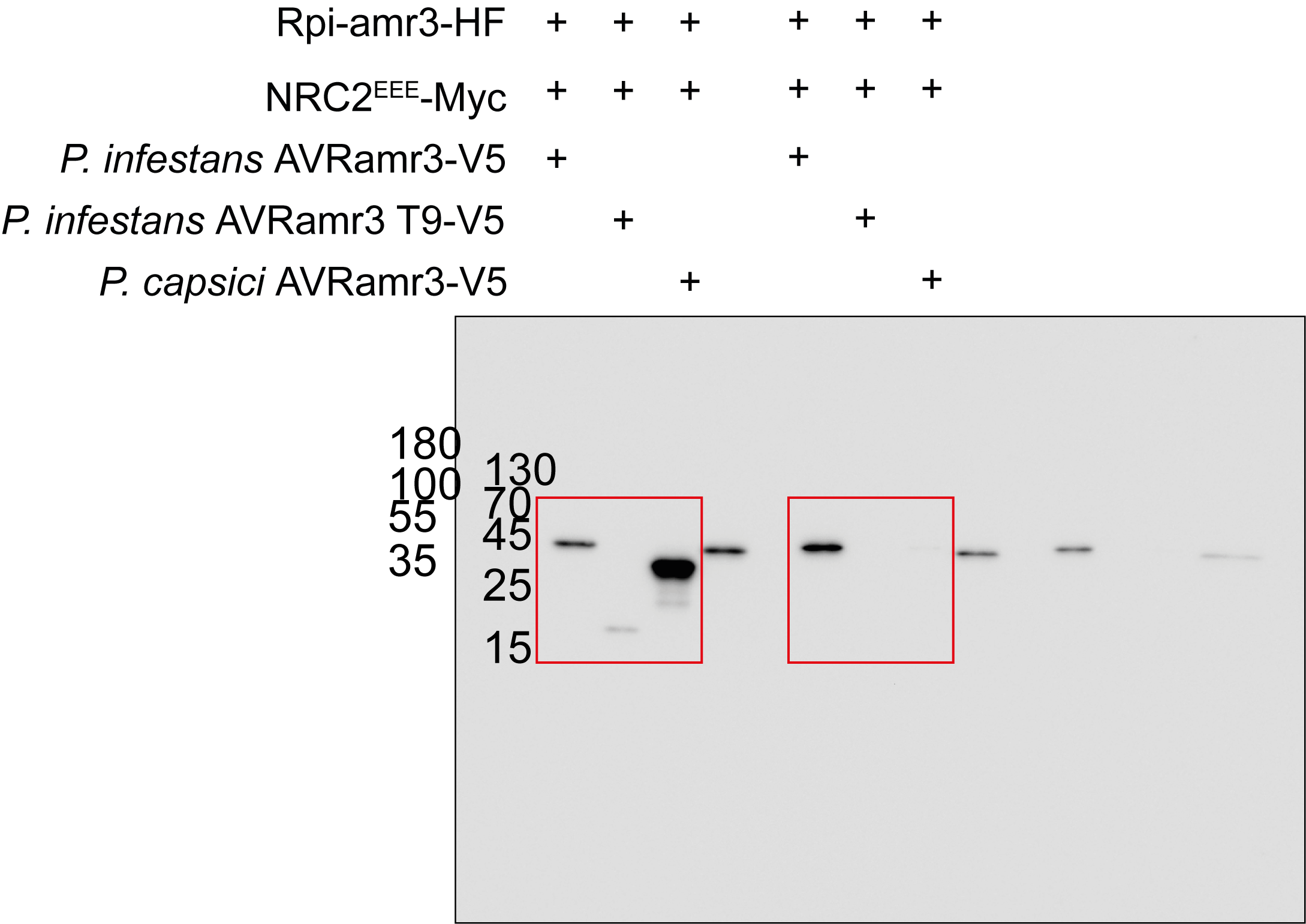

Supplement: Supplementary file 4 — Source Data for Expanded View [file EMBJ-42-e111484-s002.zip › EMBOJ-2022-11484_SourceData/Figure EV5/EV5C/SDS Western V5_annotations.tif]

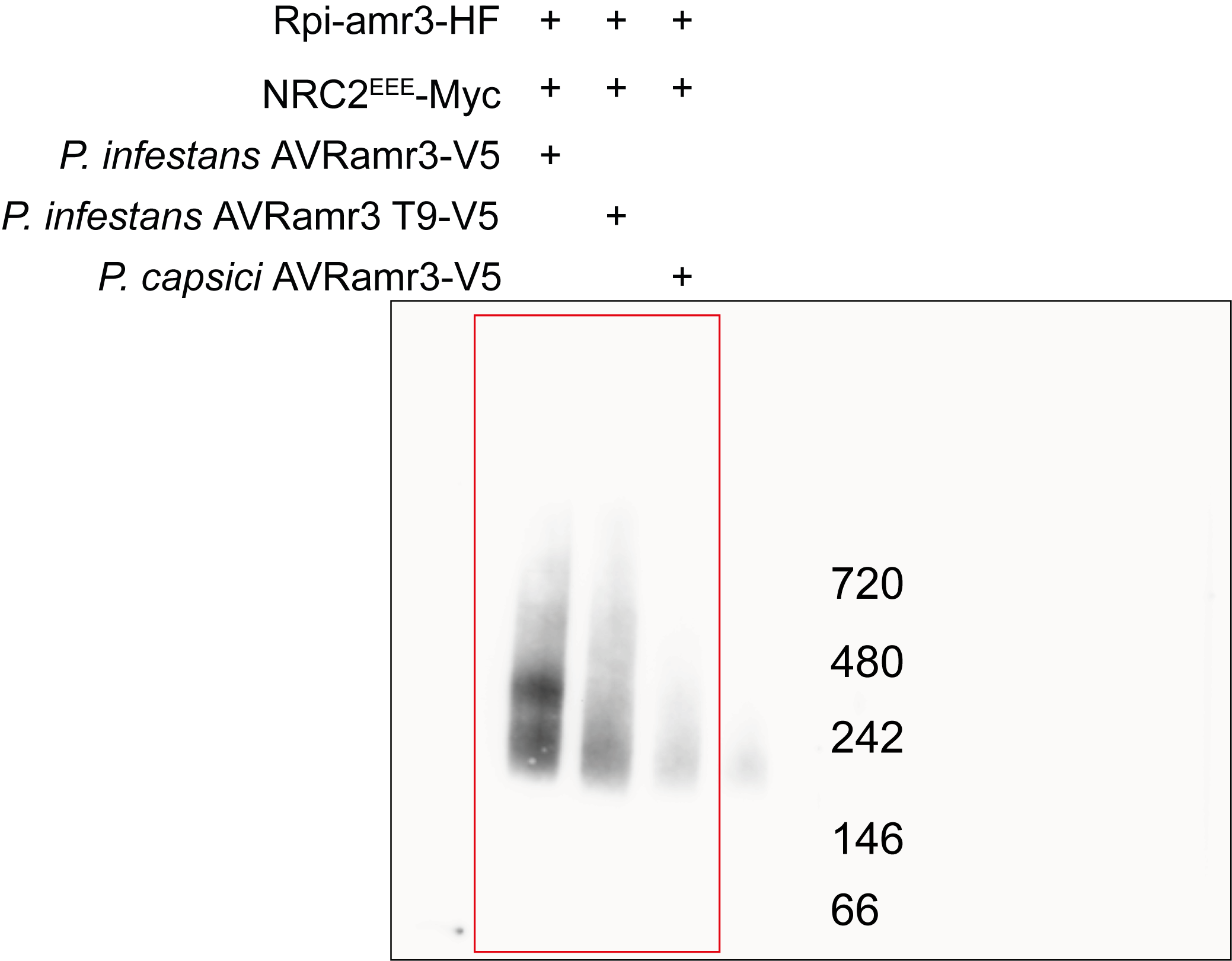

Supplement: Supplementary file 4 — Source Data for Expanded View [file EMBJ-42-e111484-s002.zip › EMBOJ-2022-11484_SourceData/Figure EV5/EV5D/BNP Western Flag_annotations.tif]

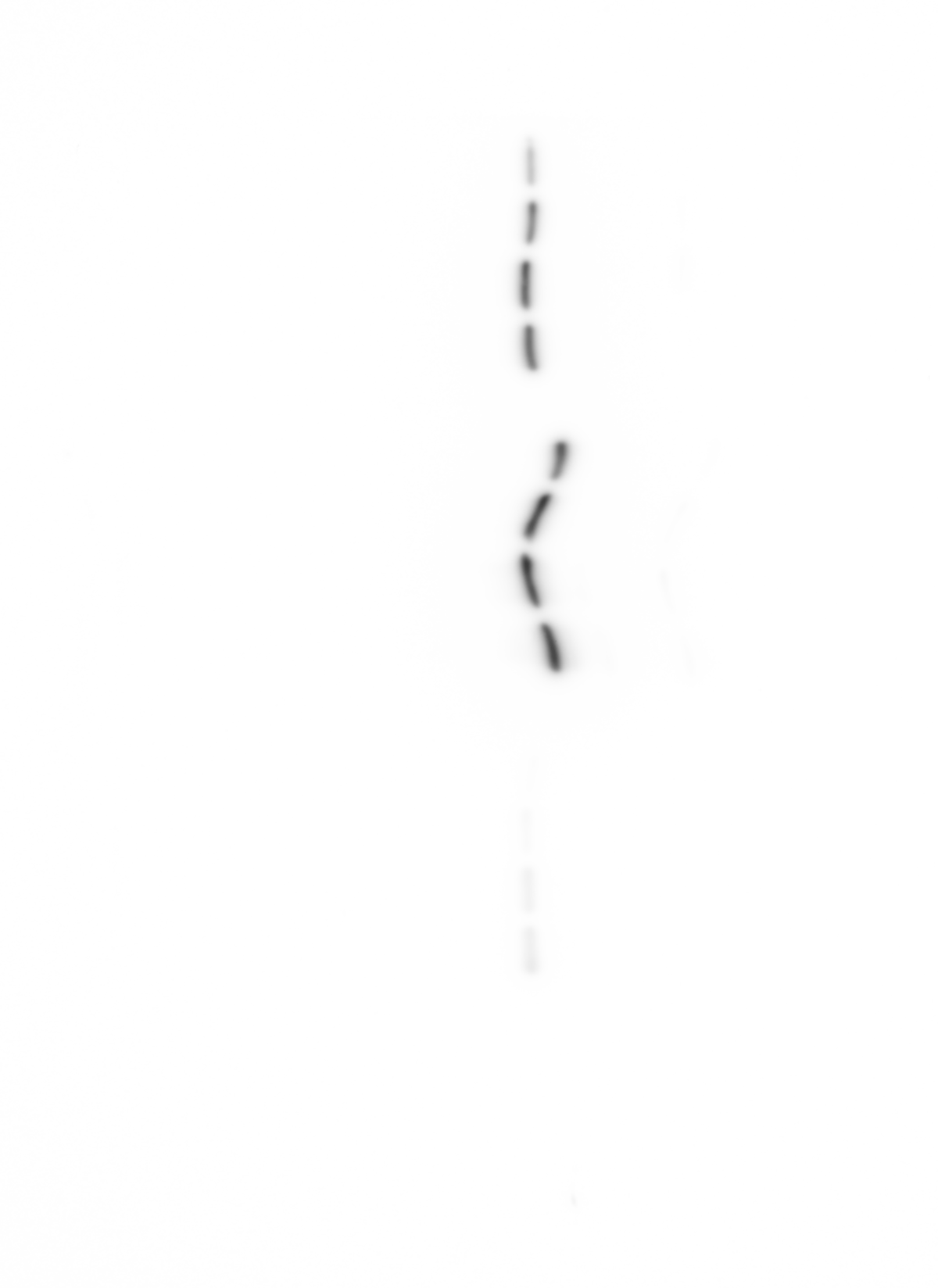

Supplement: Supplementary file 4 — Source Data for Expanded View [file EMBJ-42-e111484-s002.zip › EMBOJ-2022-11484_SourceData/Figure EV5/EV5D/SDS Western Flag IP.tif]

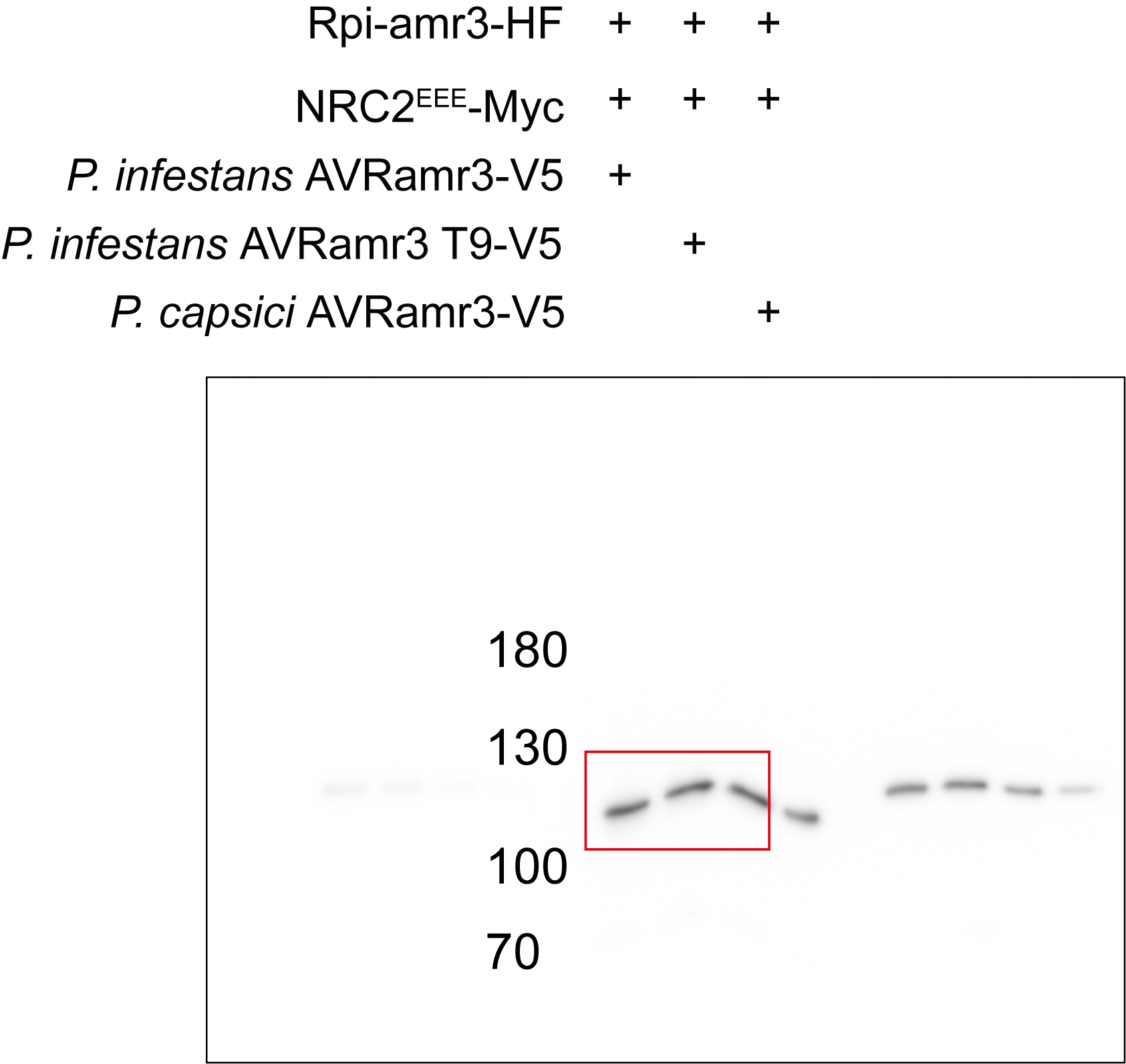

Supplement: Supplementary file 4 — Source Data for Expanded View [file EMBJ-42-e111484-s002.zip › EMBOJ-2022-11484_SourceData/Figure EV5/EV5D/SDS Western Flag IP_annotations.tif]

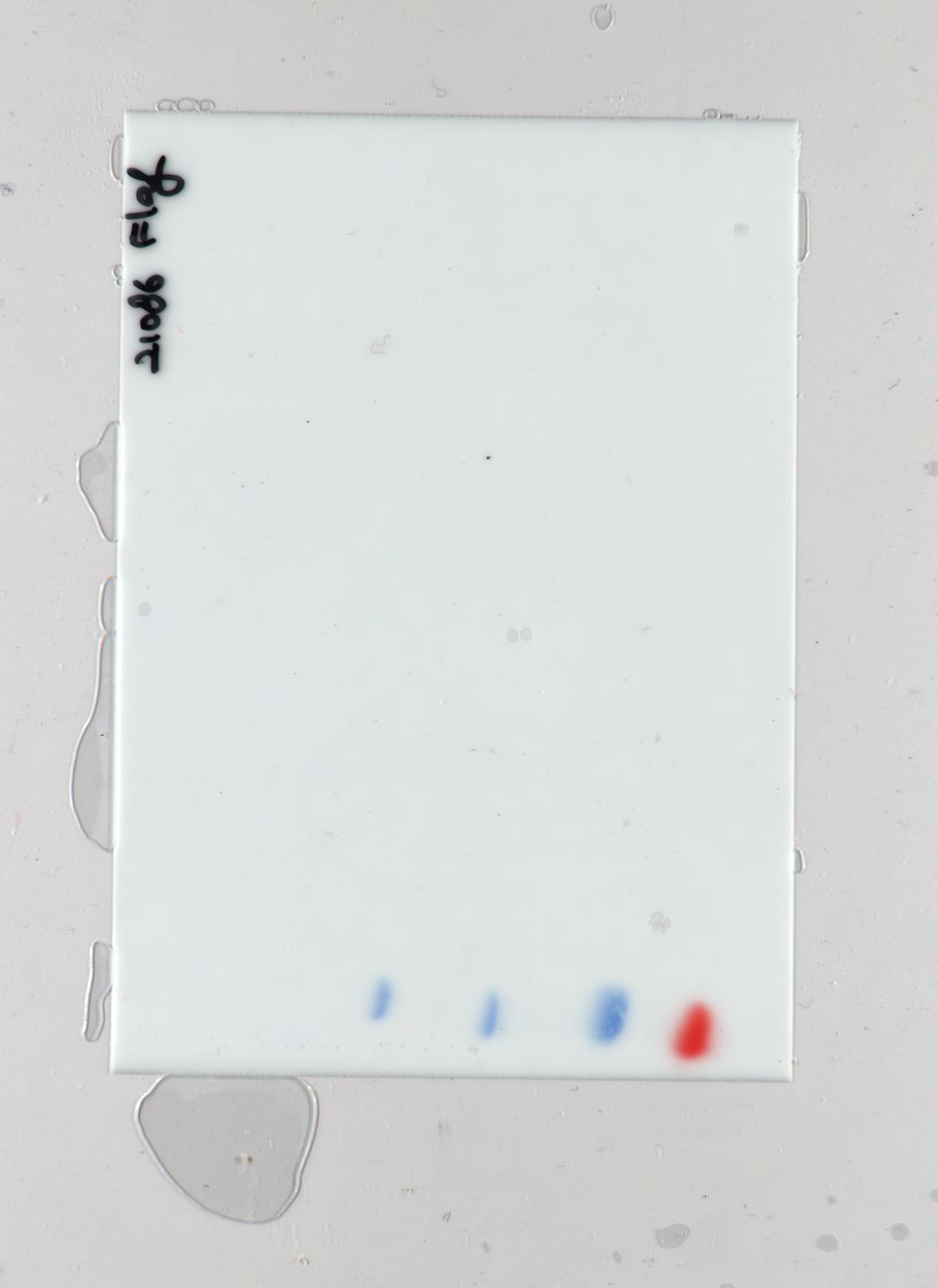

Supplement: Supplementary file 4 — Source Data for Expanded View [file EMBJ-42-e111484-s002.zip › EMBOJ-2022-11484_SourceData/Figure EV5/EV5D/SDS Western Flag Marker.jpg]

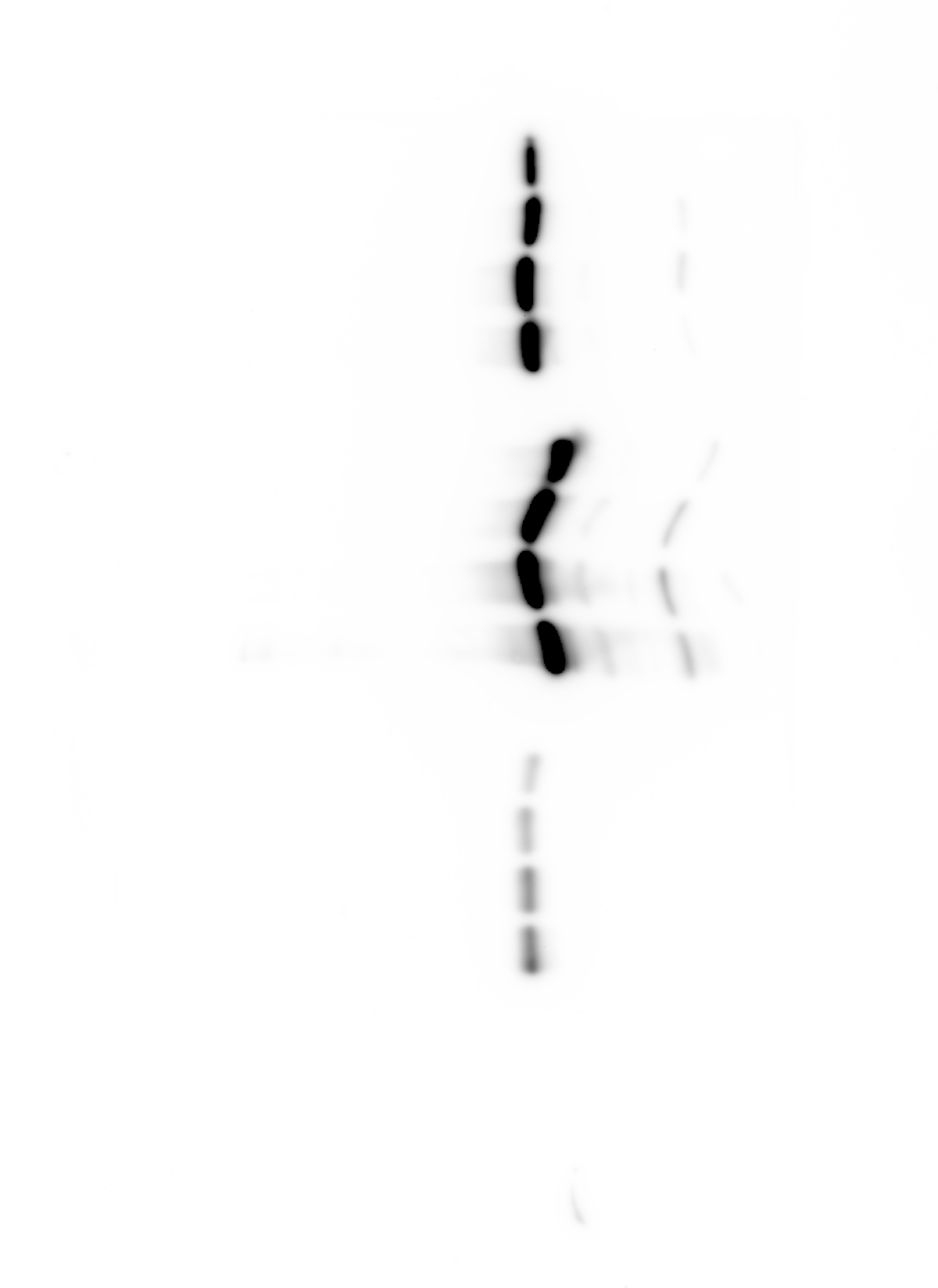

Supplement: Supplementary file 4 — Source Data for Expanded View [file EMBJ-42-e111484-s002.zip › EMBOJ-2022-11484_SourceData/Figure EV5/EV5D/SDS Western Flag input.tif]

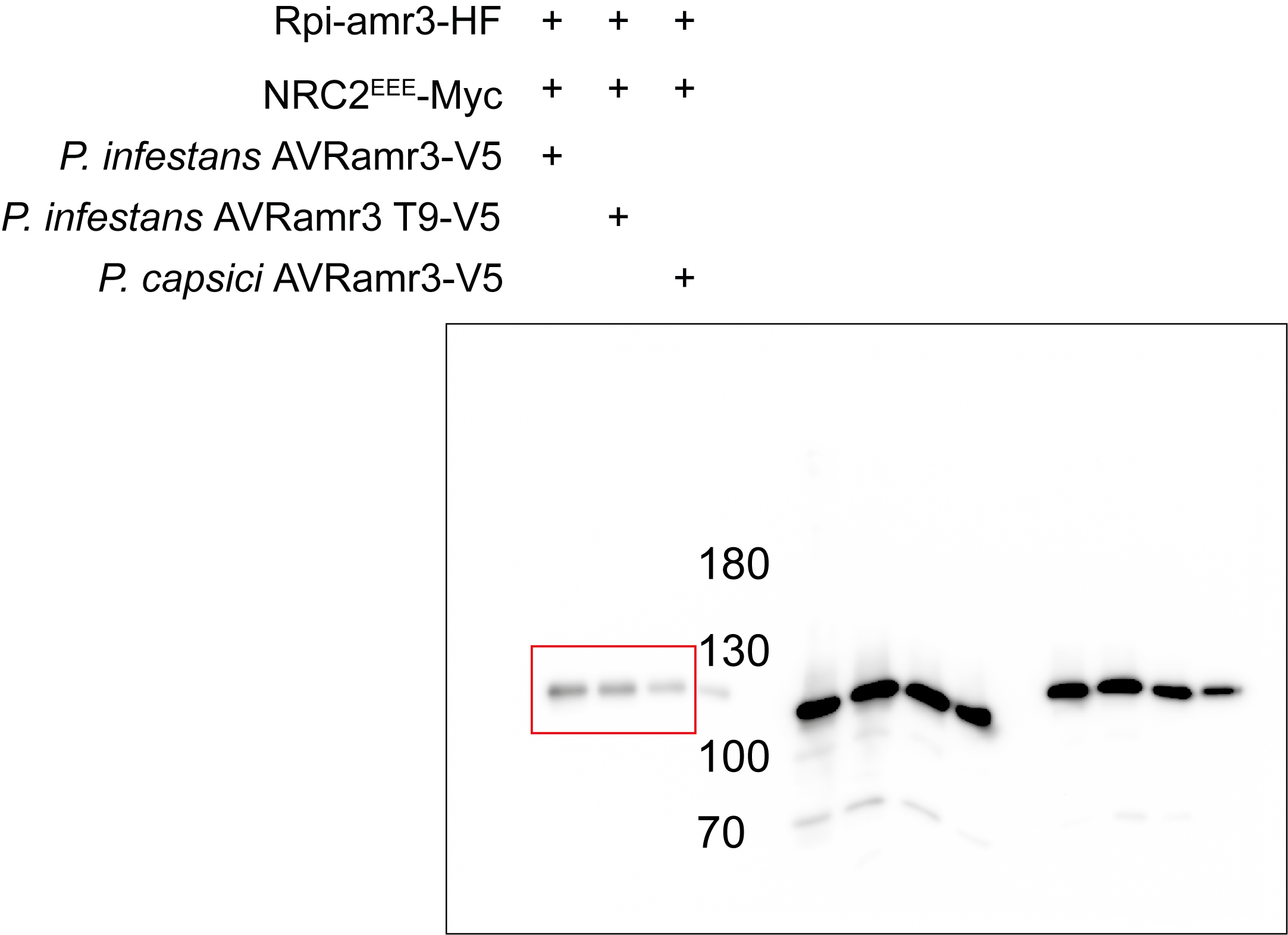

Supplement: Supplementary file 4 — Source Data for Expanded View [file EMBJ-42-e111484-s002.zip › EMBOJ-2022-11484_SourceData/Figure EV5/EV5D/SDS Western Flag input_annotations.tif]

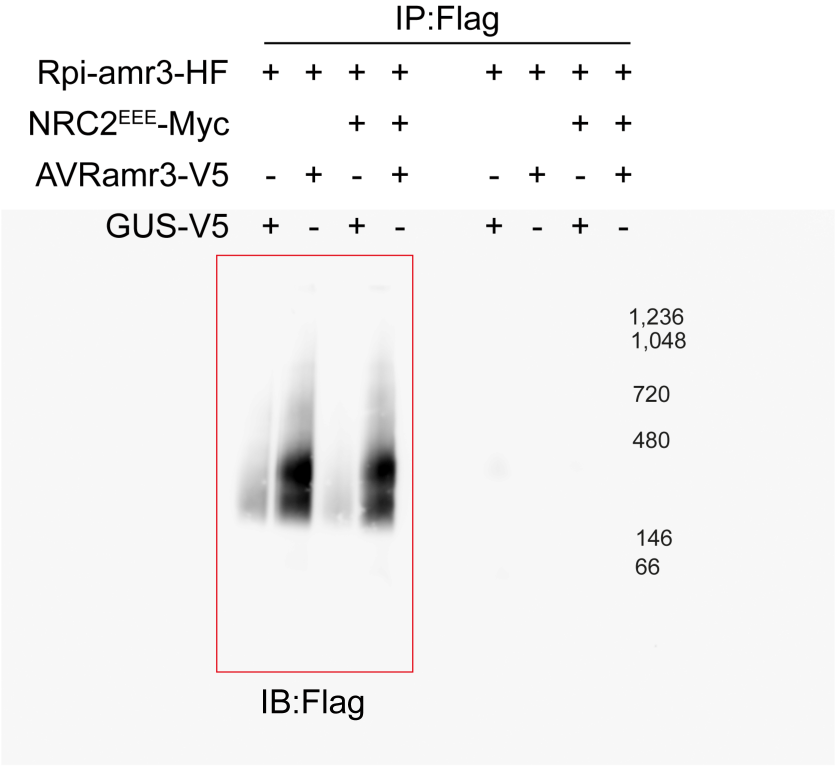

Supplement: Supplementary file 6 — Source Data for Figure 1 [file EMBJ-42-e111484-s005.zip › Figure 1/1B/Western Flag_annotated.tif]

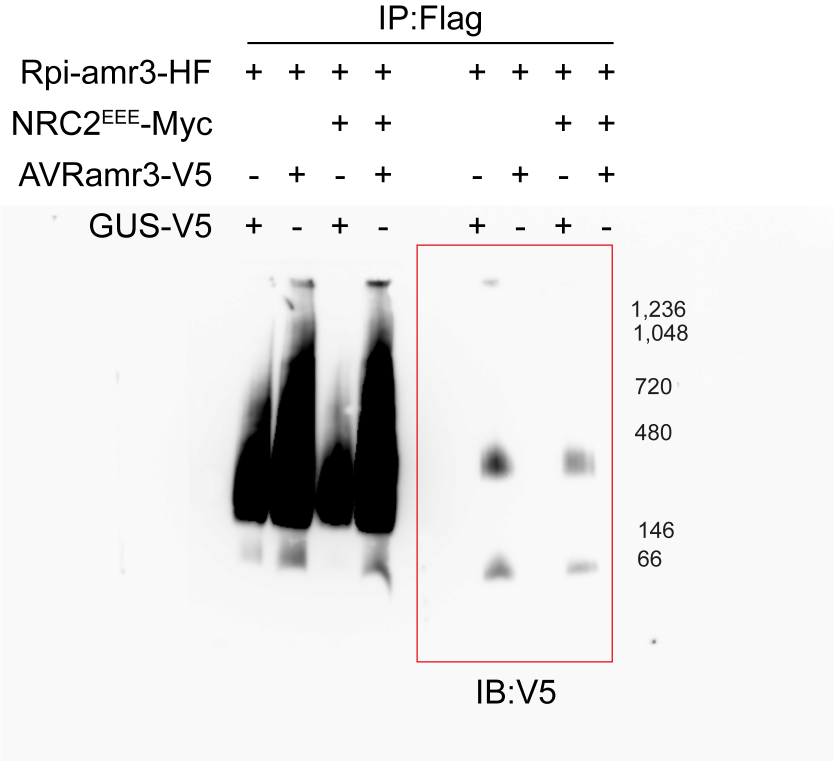

Supplement: Supplementary file 6 — Source Data for Figure 1 [file EMBJ-42-e111484-s005.zip › Figure 1/1B/Western V5_annotated.tif]

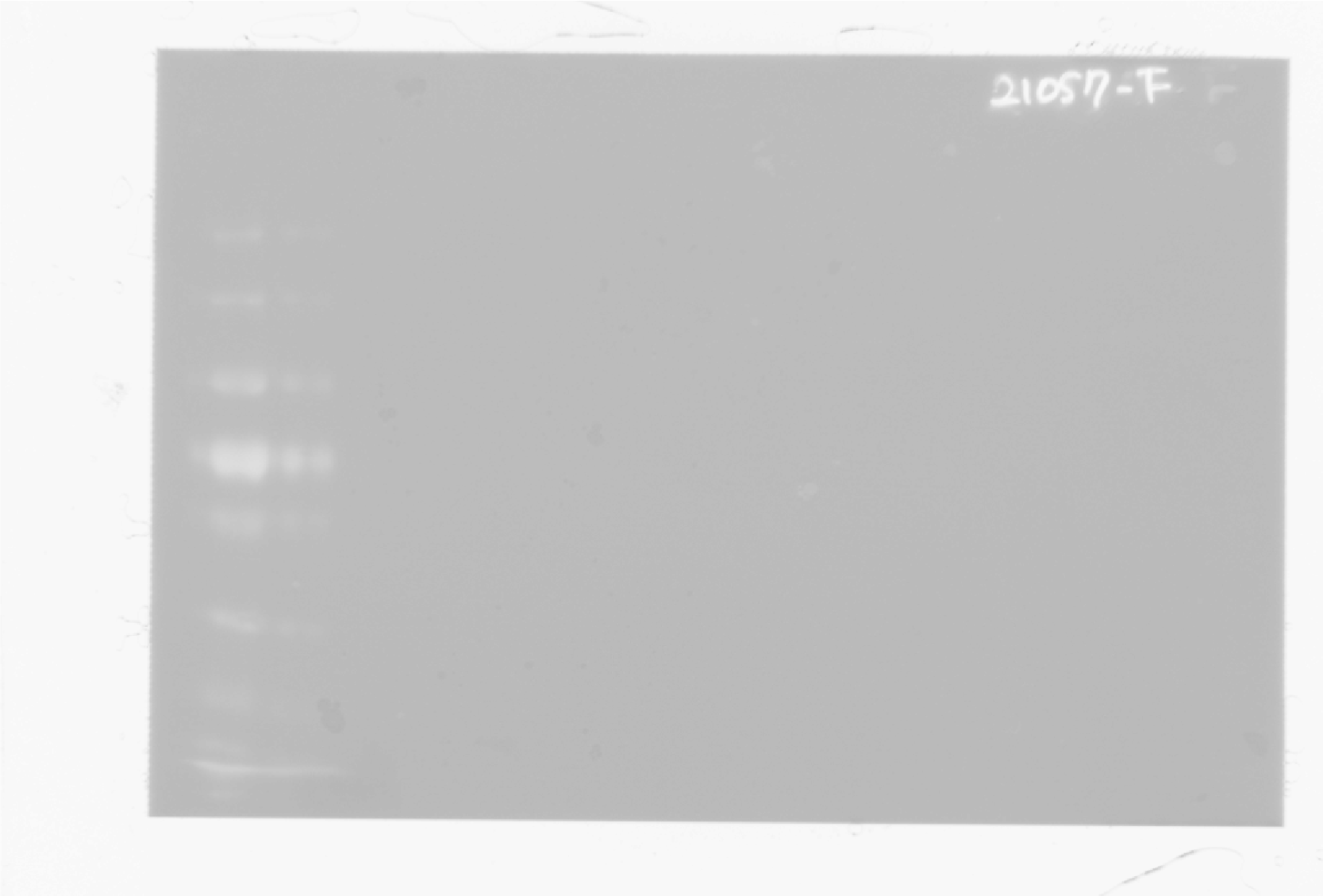

Supplement: Supplementary file 6 — Source Data for Figure 1 [file EMBJ-42-e111484-s005.zip › Figure 1/1C/Western Flag marker.tif]

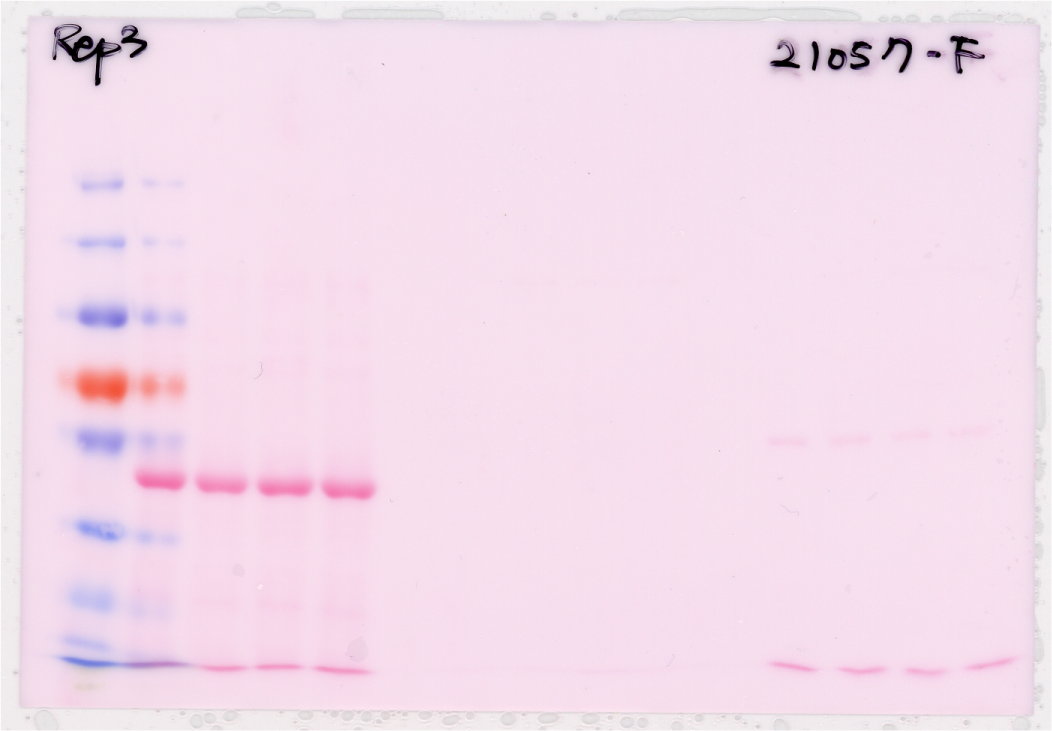

Supplement: Supplementary file 6 — Source Data for Figure 1 [file EMBJ-42-e111484-s005.zip › Figure 1/1C/Western Flag Ponceau.tif]

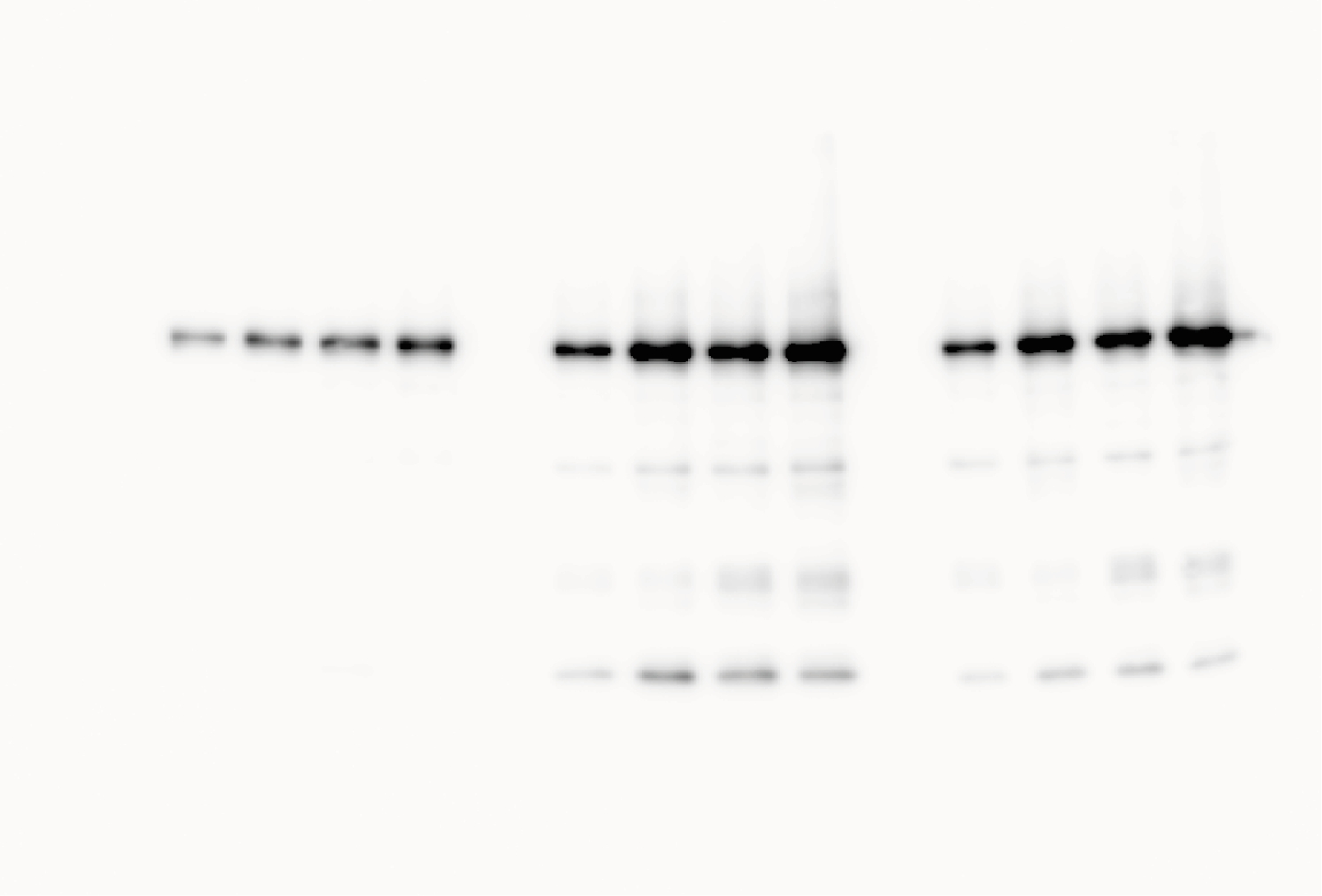

Supplement: Supplementary file 6 — Source Data for Figure 1 [file EMBJ-42-e111484-s005.zip › Figure 1/1C/Western Flag.tif]

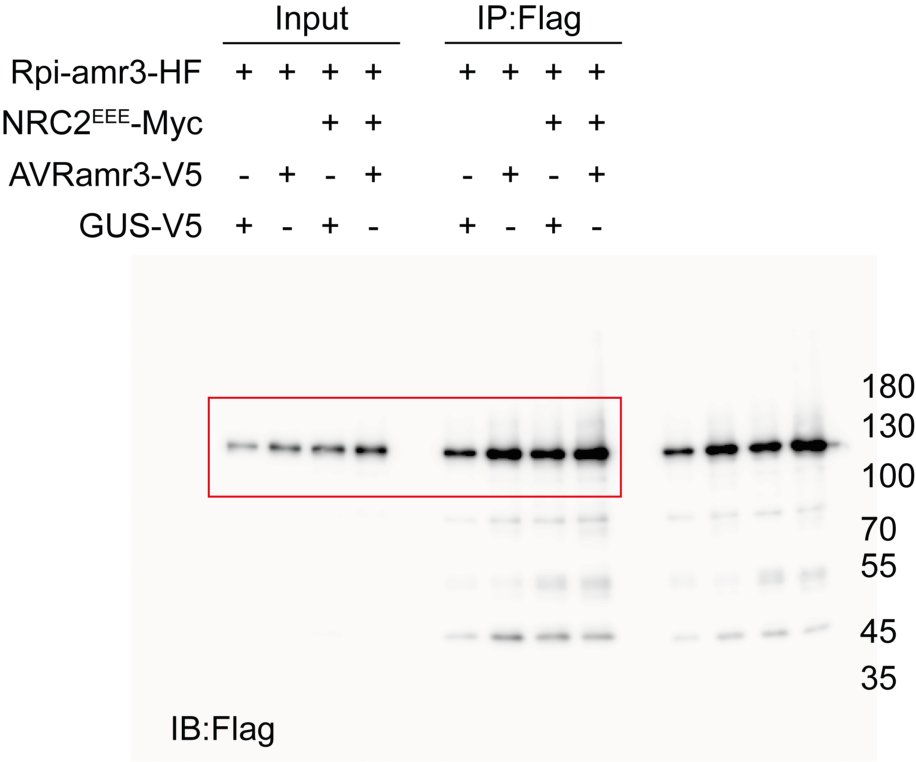

Supplement: Supplementary file 6 — Source Data for Figure 1 [file EMBJ-42-e111484-s005.zip › Figure 1/1C/Western Flag_annotated.tif]

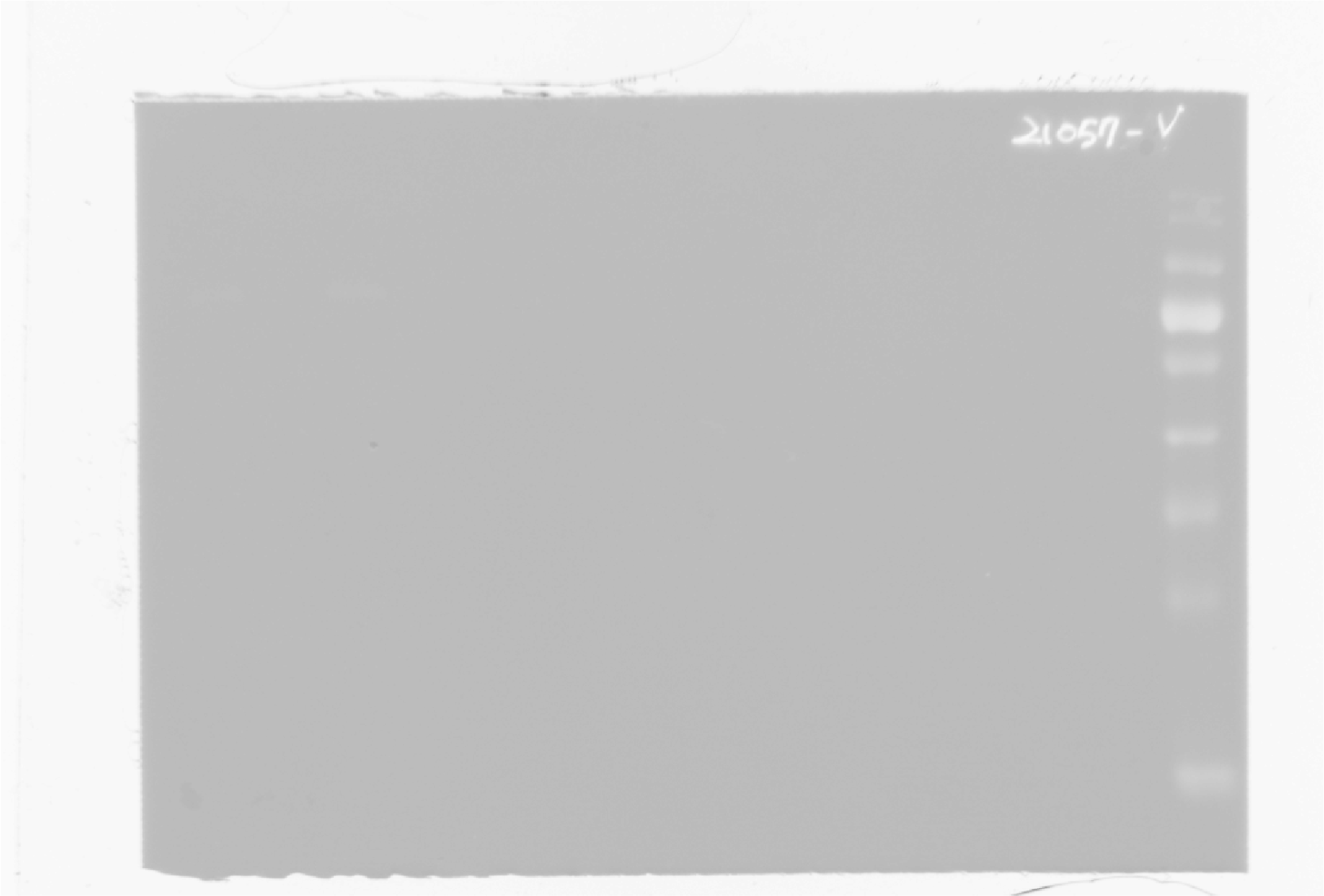

Supplement: Supplementary file 6 — Source Data for Figure 1 [file EMBJ-42-e111484-s005.zip › Figure 1/1C/Western V5 marker.tif]

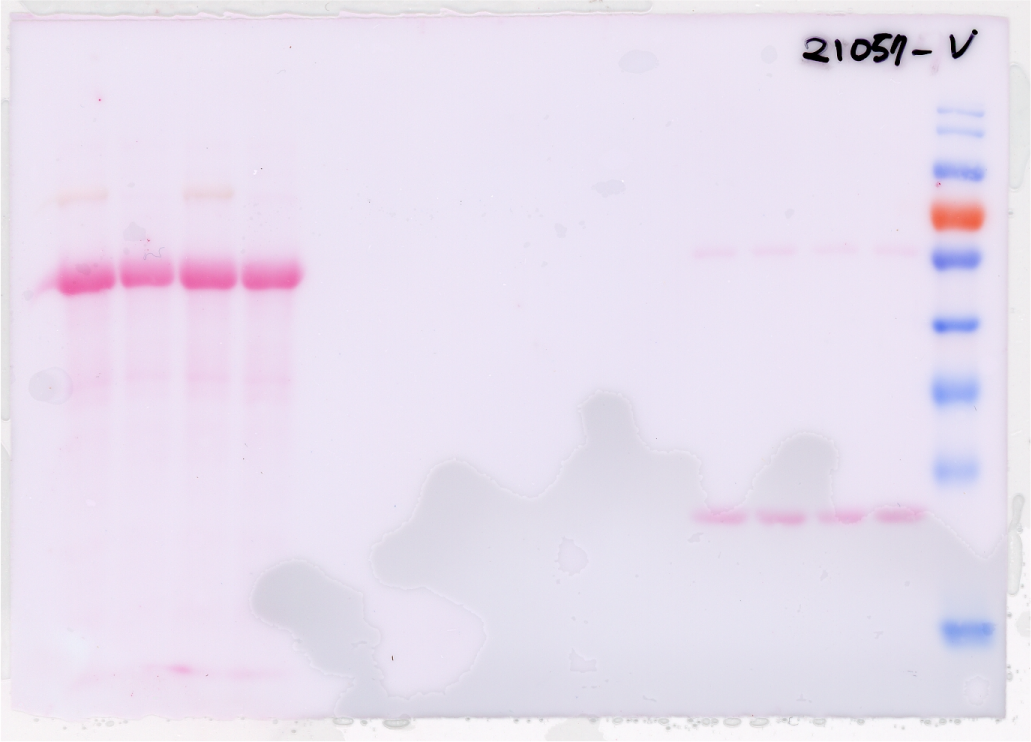

Supplement: Supplementary file 6 — Source Data for Figure 1 [file EMBJ-42-e111484-s005.zip › Figure 1/1C/Western V5 Ponceau.tif]

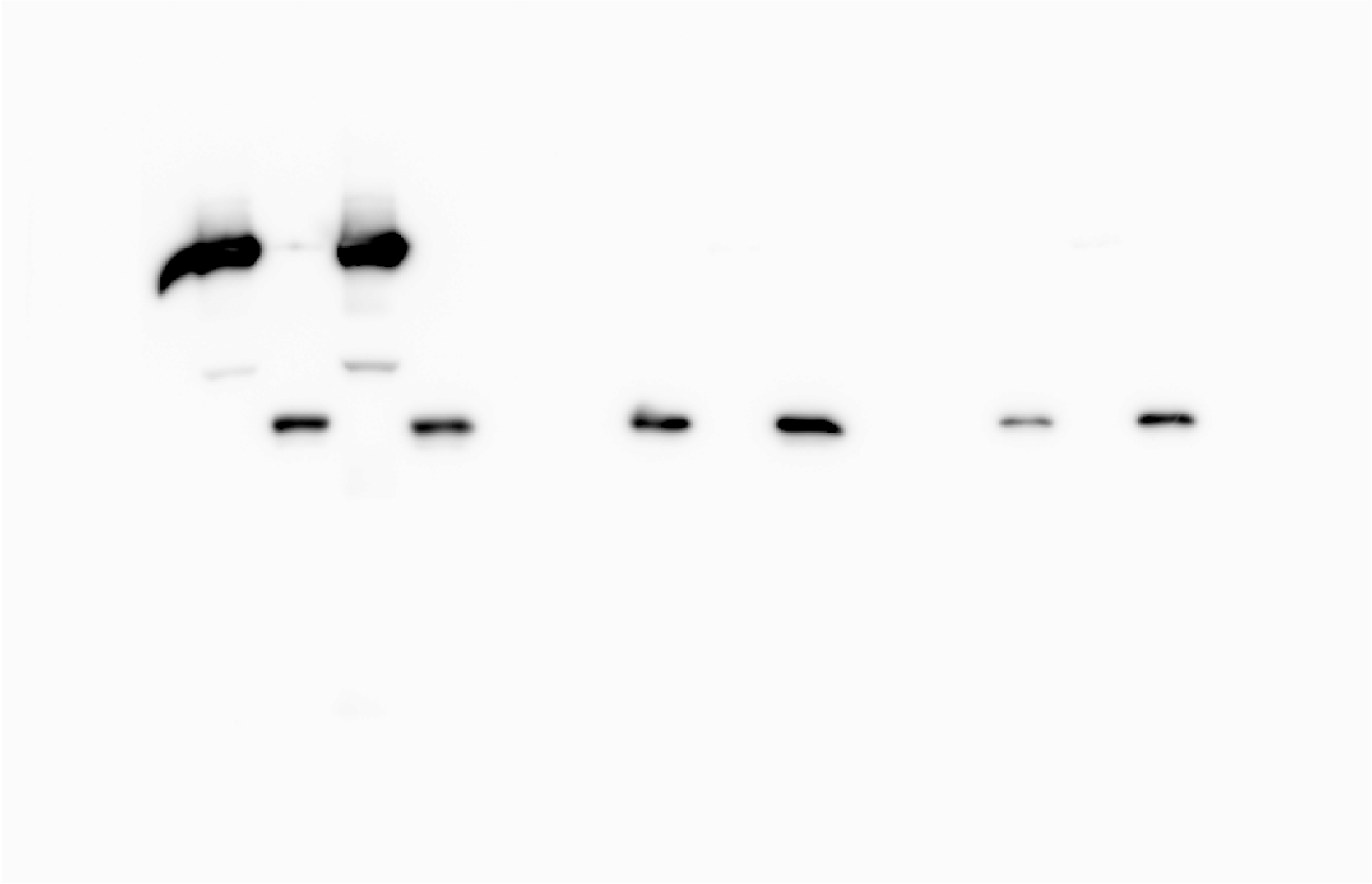

Supplement: Supplementary file 6 — Source Data for Figure 1 [file EMBJ-42-e111484-s005.zip › Figure 1/1C/Western V5.tif]

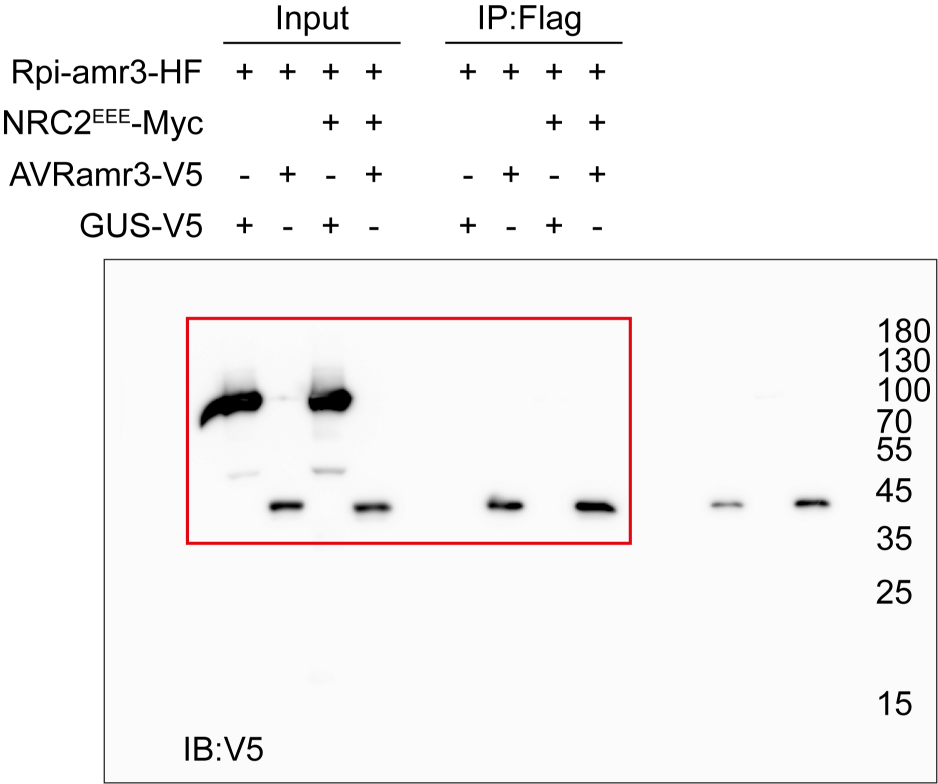

Supplement: Supplementary file 6 — Source Data for Figure 1 [file EMBJ-42-e111484-s005.zip › Figure 1/1C/Western V5_annotated.tif]

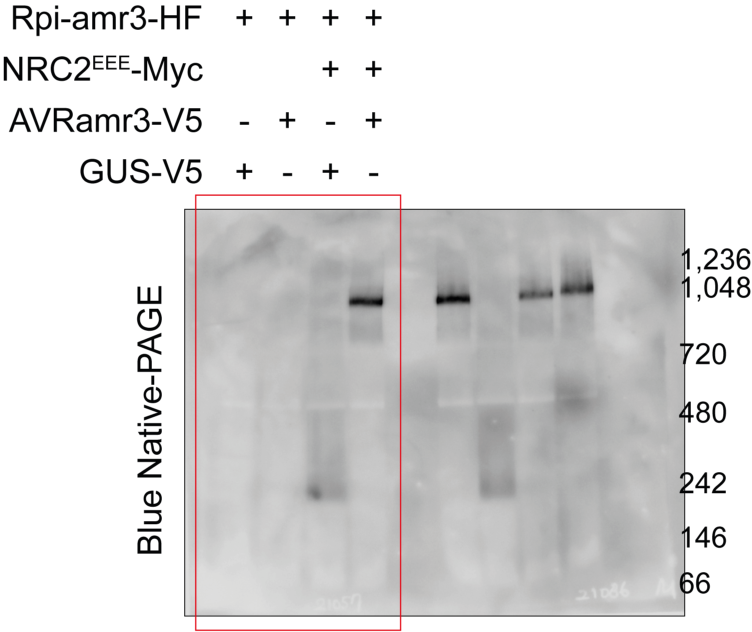

Supplement: Supplementary file 7 — Source Data for Figure 2 [file EMBJ-42-e111484-s011.zip › Figure 2/2A/BNP Western Myc_annotation.tif]

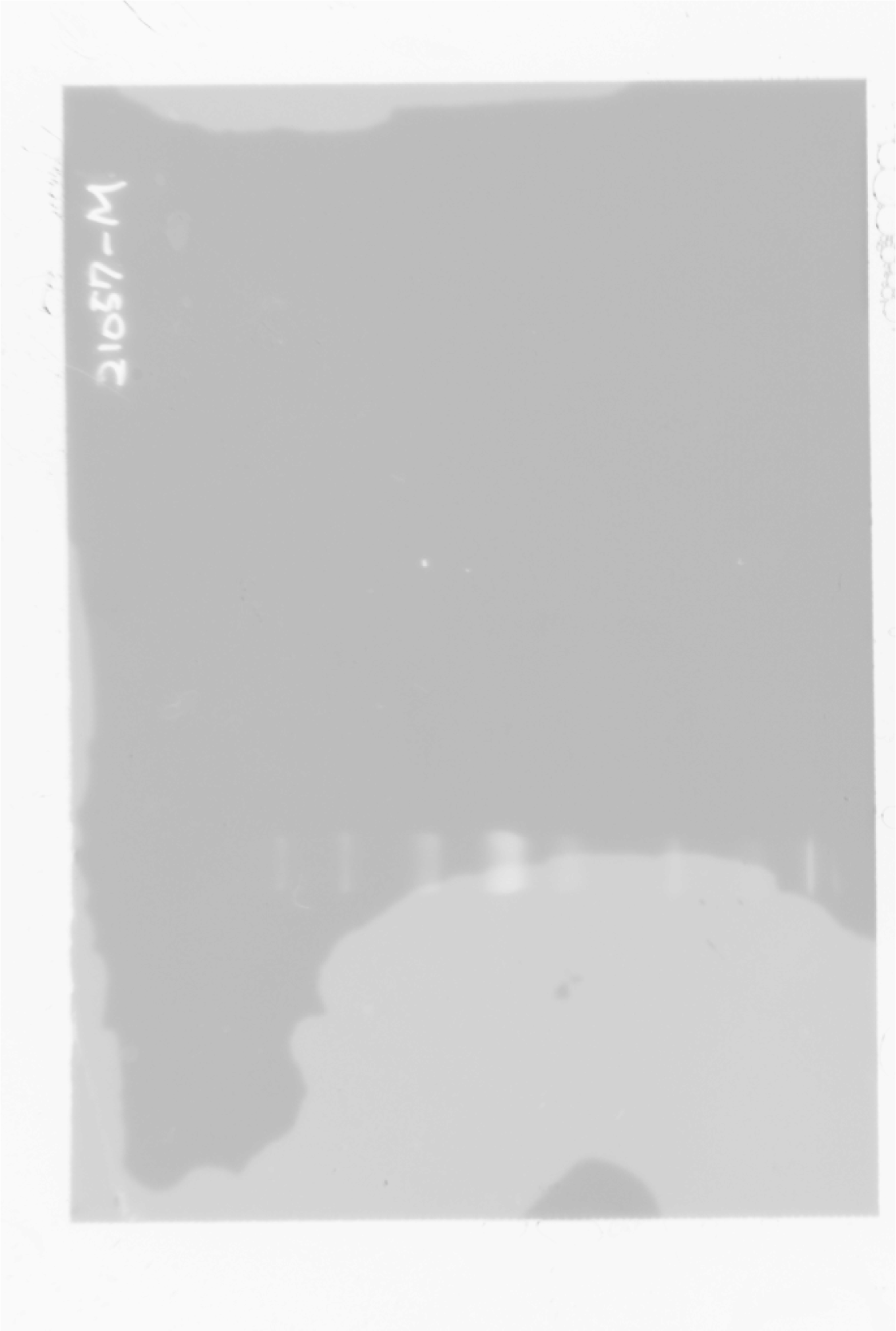

Supplement: Supplementary file 7 — Source Data for Figure 2 [file EMBJ-42-e111484-s011.zip › Figure 2/2A/SDS Western marker.tif]

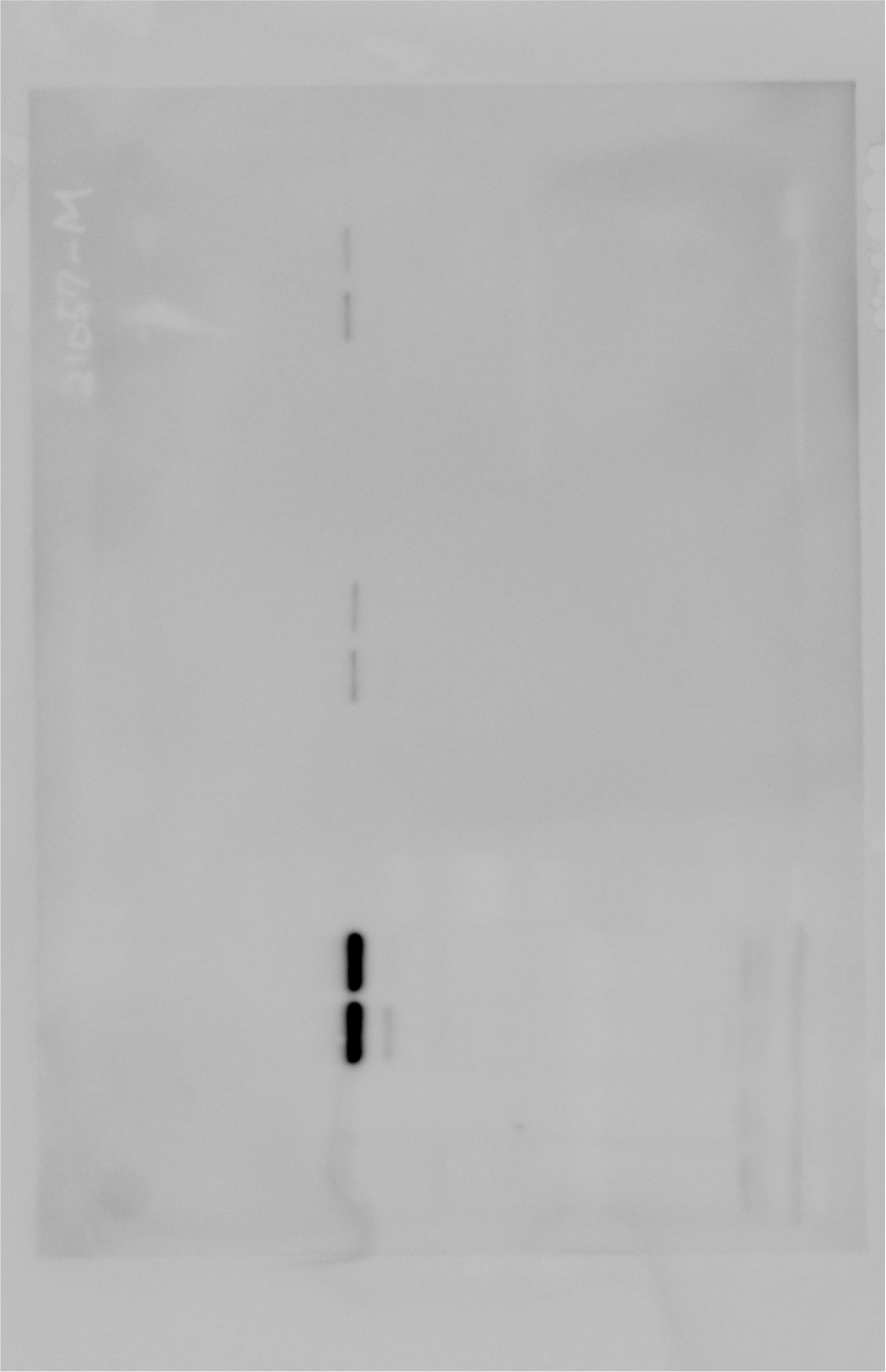

Supplement: Supplementary file 7 — Source Data for Figure 2 [file EMBJ-42-e111484-s011.zip › Figure 2/2A/SDS Western Myc.tif]

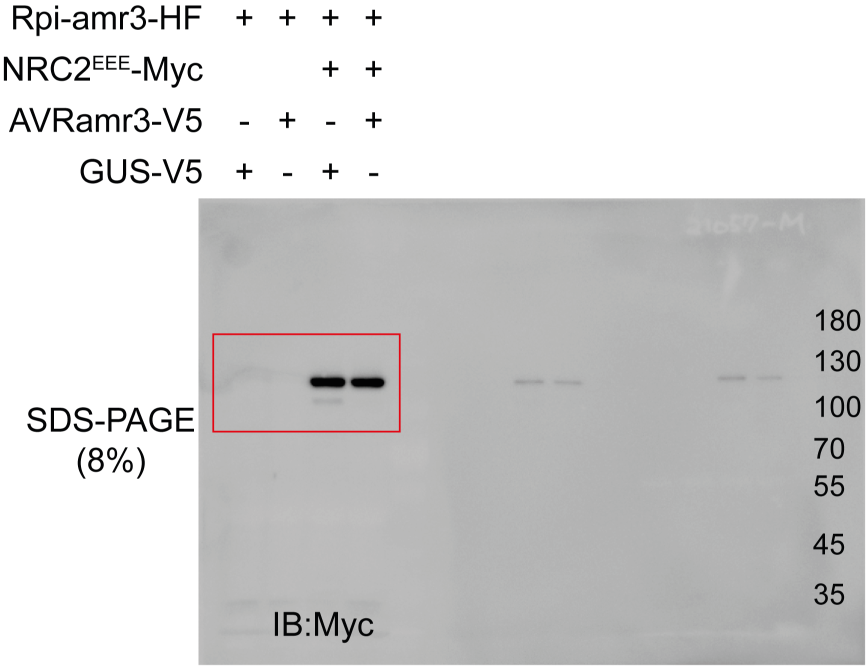

Supplement: Supplementary file 7 — Source Data for Figure 2 [file EMBJ-42-e111484-s011.zip › Figure 2/2A/SDS Western Myc_annotations.tif]

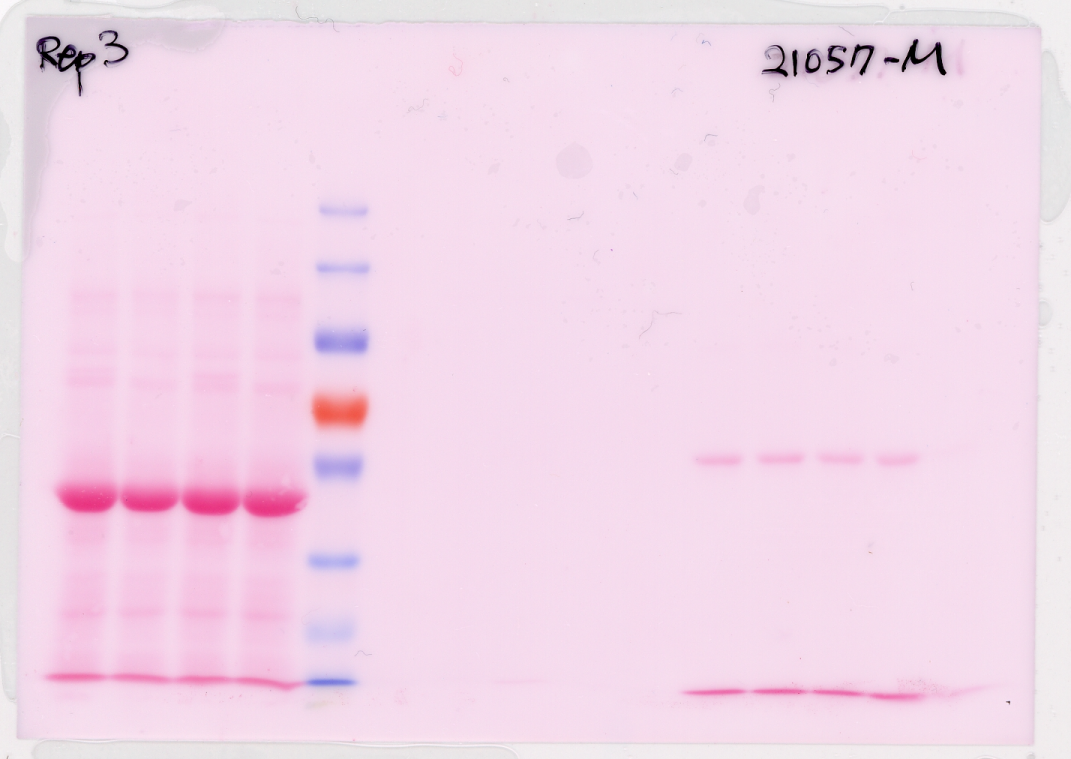

Supplement: Supplementary file 7 — Source Data for Figure 2 [file EMBJ-42-e111484-s011.zip › Figure 2/2A/Western Myc Ponceau.tif]
