# Supplementary material for: Effector‐dependent activation and oligomerization of plant NRC class helper NLRs by sensor NLR immune receptors Rpi‐amr3 and Rpi‐amr1
Source: EMBO J. 2023 Jan 2;42(5):e111484. doi: 10.15252/embj.2022111484 (PMC9975942; doi:10.15252/embj.2022111484)
Supplement: Supplementary file 5 — PDF+ [file EMBJ-42-e111484-s010.pdf]

Article

SOURCE DATA TRANSPARENT PROCESS OPEN ACCESS

THE EMBO JOURNAL

Effector-dependent activation and oligomerization of plant NRC class helper NLRs by sensor NLR immune receptors Rpi-amr3 and Rpi-amr1

Hee-Kyung Ahn[1,†], Xiao Lin[1,†], Andrea Carolina Olave-Achury[1,‡], Lida Derevnina[1,§], Mauricio P Contreras[1], Jiorgos Kourelis[1], Chih-Hang Wu[2], Sophien Kamoun[1] & Jonathan D G Jones[1,*]

Abstract

Plant pathogens compromise crop yields. Plants have evolved robust innate immunity that depends in part on intracellular Nucleotide-binding, Leucine rich-Repeat (NLR) immune receptors that activate defense responses upon detection of pathogen-derived effectors. Most "sensor" NLRs that detect effectors require the activity of "helper" NLRs, but how helper NLRs support sensor NLR function is poorly understood. Many Solanaceae NLRs require NRC (NLR-Required for Cell death) class of helper NLRs. We show here that Rpi-amr3, a sensor NLR from *Solanum americanum*, detects AVRamr3 from the potato late blight pathogen, *Phytophthora infestans*, and activates oligomerization of helper NLRs NRC2 and NRC4 into high-molecular-weight resistosomes. In contrast, recognition of *P. infestans* effector AVRamr1 by another sensor NLR Rpi-amr1 induces formation of only the NRC2 resistosome. The activated NRC2 oligomer becomes enriched in membrane fractions. ATP-binding motifs of both Rpi-amr3 and NRC2 are required for NRC2 resistosome formation, but not for the interaction of Rpi-amr3 with its cognate effector. NRC2 resistosome can be activated by Rpi-amr3 upon detection of AVRamr3 homologs from other *Phytophthora* species. Mechanistic understanding of NRC resistosome formation will underpin engineering crops with durable disease resistance.

Keywords blue native-PAGE; NLR activation; NRC; plant immunity; *Rpi-amr*
Subject Category Plant Biology
DOI 10.15252/embj.2022111484 | Received 25 April 2022 | Revised 30 November 2022 | Accepted 2 December 2022 | Published online 2 January 2023
The EMBO Journal (2023) 42: e111484

Introduction

Plants have powerful defense mechanisms, but to be effective, these must be rapidly activated at sites of attempted pathogen ingress. Activation of defense requires detection, both by cell surface receptors that usually detect relatively conserved pathogen-derived molecules such as flagellin or chitin (Lee *et al*, 2021), and by intracellular nucleotide-binding, leucine-rich repeat (NLR) receptors which detect effectors that often function for the pathogen to attenuate plant defenses (Jones *et al*, 2016).

Natural plant populations carry extensive genetic variation in immune receptor repertoires (Ngou *et al*, 2022b). Plant breeders have long exploited this genetic variation to elevate crop varietal resistance by introgression of multiple disease *Resistance* (*R*) genes from wild relatives. *R* genes usually encode NLR immune receptors (Meyers *et al*, 1999). Some plant species carry scores or even hundreds of different NLR immune receptor genes, with extensive allelic diversity and presence/absence polymorphism (Barragan & Weigel, 2021). These NLR immune receptors can confer resistance to bacteria, fungi, oomycetes, viruses and even invertebrates (Ngou *et al*, 2022a), triggering broad interest in how these immune receptors can activate defense upon recognition of molecules from such diverse sources.

NLRs are broadly categorized into three subclasses, based on their N-terminal domains. TIR-NLRs, CC-NLRs, and CC_R-NLRs have N-terminal Toll-like, interleukin-1 receptor, resistance (TIR) domains, coiled-coil (CC) domains, and RPW8-like (CC_R) domains, respectively (Meyers *et al*, 1999; Collier *et al*, 2011). The N-terminal domains of the NLRs have direct roles in signaling upon effector-dependent oligomerization of the NLRs. For example, the TIR-NLRs ROQ1 and RPP1 form a tetramer upon detection of their cognate recognized effectors, activating an NADase activity by forming a tetramer of the TIR domain (Ma *et al*, 2020; Martin *et al*, 2020). The enzymatic activity generates small molecules that are required for

1 The Sainsbury Laboratory, University of East Anglia, Norwich, UK
2 Institute of Plant and Microbial Biology, Academia Sinica, Taipei, Taiwan
*Corresponding author. Tel: +44 01603 450100; E-mail: jonathan.jones@tsl.ac.uk
†These authors contributed equally to this work
‡Present address: Earlham Institute, Norfolk, UK
§Present address: Department of Plant Sciences, Crop Science Centre, University of Cambridge, Cambridge, UK

© 2022 The Authors. Published under the terms of the CC BY 4.0 license

downstream signaling via EDS1 (Huang *et al*, 2022; Jia *et al*, 2022). The oligomerization of ZAR1 and Sr35 into a pentamer upon effector detection induces assembly of oligomers of the N-terminal α-helix in the CC domain to form a cation channel in the plasma membrane that is required for signaling (Wang *et al*, 2019a; Bi *et al*, 2021; Förderer *et al*, 2022; Zhao *et al*, 2022). The CC_R-NLR NRG1.1 and ADR1 also oligomerize and localize to the plasma membrane and can mediate calcium ion influx (Jacob *et al*, 2021, preprint: Feehan *et al*, 2022). However, whether all NLRs form resistosomes upon activation is unclear.

NLRs can function independently as singletons, but accumulating evidence suggests that many NLRs function in pairs or networks. The effector-detecting NLR is often named a "sensor" NLR, whereas the downstream signaling NLRs that convert recognition into immune activation are called "helper" NLRs (Feehan *et al*, 2020). Paired NLRs, such as RRS1/RPS4 or RGA4/RGA5, are divergently transcribed, and one of the NLRs often carries an integrated domain (ID) for effector detection, while the other signals upon recognition (Cesari *et al*, 2014). In contrast, helper NLRs of the CC_R-NLR and NRC classes map to different genomic loci from the "sensor" NLRs and are required for the activity of multiple "sensor" NLRs (Wu *et al*, 2018; Jubic *et al*, 2019; Feehan *et al*, 2020). The first CC_R-type helper NLR, NRG1 (Peart *et al*, 2005), was found to be required for the function of TIR-NLRs (Qi *et al*, 2018; Castel *et al*, 2019; Wu *et al*, 2019), and the related ADR1 helper NLRs can contribute to both TIR-NLR and CC-NLR function (Saile *et al*, 2020).

The NRC class of helper NLR was discovered in the Solanaceae and is widespread in the asterid but not the rosid clade of angiosperms (Wu *et al*, 2017). The NRC class of helper NLRs are phylogenetically related to their corresponding sensor NLRs in the asterid plant family, and ~50% of Solanaceae NLRs are either NRC-dependent sensor NLRs or NRCs (Wu *et al*, 2017). Different sensor NLRs depend on different combinations of helper NLRs to activate the immune response (Wu *et al*, 2018). A conserved N-terminal MADA motif was found in the NRC family and in ~20% of other CC-NLRs, including ZAR1 from *Arabidopsis* (Adachi *et al*, 2019), which suggests that NRCs might activate defense via similar mechanisms to ZAR1. Mutating the MADA motifs of NRC2, NRC3 or NRC4 from *N. benthamiana* results in loss of function, and the corresponding N-terminal α1 helix can be swapped with the equivalent region of ZAR1 (Adachi *et al*, 2019; Duggan *et al*, 2021; Kourelis *et al*, 2022). This suggests that the MADA motif of NRCs might form a cation-selective channel to activate immune signaling and cell death, as does ZAR1 (Bi *et al*, 2021). However, how the NRC-dependent immune response is activated upon effector detection by sensor NLRs remains unknown.

Phytophthora diseases cause yield loss for many important crop plants (Kamoun *et al*, 2015). These diseases are mainly controlled by agrichemical sprays (Cooke *et al*, 2011). Many *R* genes against *P. infestans* (*Rpi*) genes were cloned from wild potatoes (Vleeshouwers *et al*, 2011). We cloned the *Rpi-amr3* and *Rpi-amr1* genes from *Solanum americanum* for resistance against *P. infestans*, and both *Rpi* genes confer broad-spectrum resistance against late blight in potato (Witek *et al*, 2016, 2021). We also defined the cognate effector genes *Avramr3* and *Avramr1* from *P. infestans* (Lin *et al*, 2020, 2022b). Rpi-amr3 and Rpi-amr1 can also recognize AVRamr3 and AVRamr1 homologs from other *Phytophthora* pathogens (Witek *et al*, 2021; Lin *et al*, 2022b). Rpi-amr3 and Rpi-amr1 are NRC2/3/4- and NRC2/3-dependent, respectively (Witek *et al*, 2021; Lin *et al*, 2022b).

Here, we used *nrc2/3/4* CRISPR knockout *N. benthamiana* line (Wu *et al*, 2020) and transient expression of Rpi-amr3/AVRamr3 or Rpi-amr1/AVRamr1, and of MADA motif mutants of NRC2 ($NRC2^{EEE}$) or NRC4 ($NRC4^{AAA}$) to study the effector-dependent activation of the helper NLRs via sensor NLRs. We found that upon effector recognition, both Rpi-amr3 and Rpi-amr1 activate formation of a high-molecular-weight complex of NRC2, dependent on a functional ATP-binding motif of both NLRs. We also found, consistent with previous data (Witek *et al*, 2021; Lin *et al*, 2022b), that NRC4 is not oligomerized by activation of Rpi-amr1. Intriguingly, some AVRamr3 homologs from other *Phytophthora* pathogens such as *P. parasitica* can also activate NRC2 resistosome formation through Rpi-amr3. This finding could be pivotal for breaking the restricted taxonomic functionality (RTF) of some *NLR* genes and elevating disease resistance in crops that lack *NRC* genes (Tai *et al*, 1999).

Results

Rpi-amr3 and AVRamr3 form an NRC-independent protein complex

Rpi-amr3 and Rpi-amr1 are canonical CC-NLRs in *S. americanum* that recognize *P. infestans* effectors AVRamr3 and AVRamr1, respectively (Witek *et al*, 2016, 2021; Lin *et al*, 2022b; Fig 1A). Multiple NRC helper NLRs in *N. benthamiana* support immune activation by Rpi-amr3 and Rpi-amr1 (Witek *et al*, 2021; Lin *et al*, 2022b), and effector-dependent activation of Rpi-amr3 and Rpi-amr1 leads to cell death, or HR (hypersensitive response) in *N. benthamiana* leaves. Therefore, we chose transient *Agrobacterium* infiltration in *N. benthamiana nrc2/3/4 knockout* (Wu *et al*, 2020) as a model system to test the immune activation mechanism of Rpi-amr3 or Rpi-amr1 via NRC2.

Effectors of *Phytophthora* species commonly carry the RXLR (Arg-X-Leu-Arg) motif, which is also found in AVRamr3 and AVRamr1 (Lin *et al*, 2020, 2022b). RXLR effectors often contain tandem repeats of the structural WY domains with conserved Trp (W) and Tyr (Y) residues (Boutemy *et al*, 2011; He *et al*, 2019). Previously, we showed that AVRamr3 had 4 predicted WY domains (Lin *et al*, 2022b). In this study, we show that AVRamr1 is predicted to have 3 WY domains, with the third WY domain (WY3) showing conserved structure and conserved Trp and Tyr residues with AVR3a11 (Fig EV1A–D; Boutemy *et al*, 2011). Similar to Rpi-amr3 and AVRamr3 recognition, Rpi-amr1 interacts with AVRamr1 *in planta* (Figs EV1F and EV3A).

We tested whether Rpi-amr3 and AVRamr3 associate to form high-molecular-weight protein complexes with and without NRC. HisFlag (HF)-tagged Rpi-amr3 was co-expressed with V5-tagged AVRamr3 (Fig 1B and C) in *nrc2/3/4* knockout *N. benthamiana* leaves. Immunoprecipitates of Rpi-amr3-HF were then analyzed with blue native-PAGE (Fig 1B).

We used non-denaturing PAGE methods to monitor the presence of protein complexes. The use of Coomassie G250 dye in blue native-PAGE enables protein complexes to migrate towards the anode according to their size (Wittig *et al*, 2010). This method was used to identify composition of mitochondrial membrane protein

 © 2022 The Authors

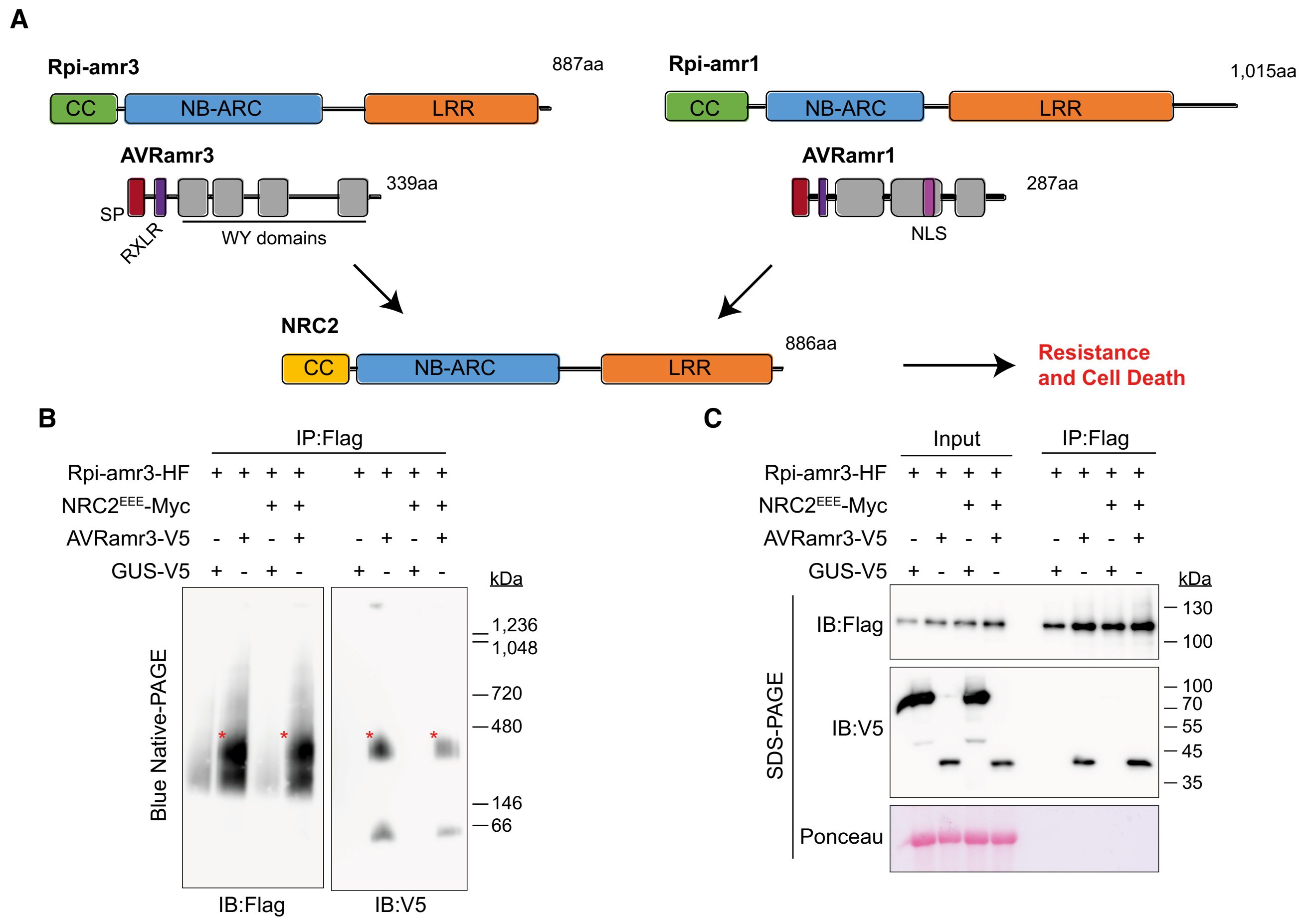

Figure 1. Rpi-amr3 and AVRamr3 form protein complexes unaltered by NRC2^{EEE}-Myc.

A Schematic model of NRC2-dependent resistance by sensor NLRs Rpi-amr3, Rpi-amr1 and cognate effectors AVRamr3 and AVRamr1, respectively. Each domain is labeled and represented with a different color. CC, coiled-coil; NB-ARC, nucleotide binding domain shared by APAF-1, R genes, CED-4; LRR, Leucine-rich repeat; SP, signal peptide; RXLR, conserved motif found in *Phytophthora* effectors; WY domains, domains in RXLR effectors with conserved Trp (W) and Tyr (Y) residues; NLS, nuclear localization signal.

B Blue native-PAGE loading of protein extracts from *nrc2/3/4 knockout N. benthamiana* plants after immunoprecipitation with anti-Flag antibody. Co-migration of Rpi-amr3-HF and AVRamr3-V5 are indicated (*, red). Same samples were loaded twice on one blue native-PAGE gel, transferred onto one membrane, and then the membrane was cut into two and immunoblotted separately. GUS-V5, β-glucuronidase fused with V5 tag.

C NRC2^{EEE}-Myc does not alter association between Rpi-amr3 and AVRamr3. Samples of (B) were SDS-boiled and loaded on SDS–PAGE. Input samples were taken prior to immunoprecipitation to show expression of all proteins.

Data information: Ponceau S staining of rubisco large subunit serve as loading control for panel (C). Molecular weight markers (in kilodaltons, kDa) are shown on the right. Experiments were done with at least three biological replicates with similar results. Representative images are shown.
Source data are available online for this figure.

complexes and photosynthetic protein complexes in plants (Eubel *et al*, 2005). Recently, this method has been widely used to reveal oligomeric changes of NLRs in plants (Li *et al*, 2019; Hu *et al*, 2020; Na Ayutthaya *et al*, 2020; Jacob *et al*, 2021; preprint: Feehan *et al*, 2022). We used the blue native-PAGE method to investigate not only protein–protein interactions, but also the approximate size of protein complexes *in vivo*.

Rpi-amr3-HF expression was stabilized upon co-expression with cognate effector AVRamr3 (Fig 1B). We detected a slow-migrating protein form of Rpi-amr3-HF when AVRamr3-V5 was co-expressed (Fig 1B, red asterisk). AVRamr3-V5 that had been co-immunoprecipitated with Rpi-amr3-HF migrated as two different species on blue native-PAGE (Fig 1B). However, AVRamr3 expressed alone migrated faster than when expressed with Rpi-amr3. (Fig EV2A). This shows that AVRamr3 forms a protein complex with Rpi-amr3 that migrates slower than either Rpi-amr3 and AVRamr3 alone. However, neither slow-migrating forms were of the size expected of an Rpi-amr pentamer in complex with a cognate effector, in contrast to the size of the ZAR1 resistosome (Hu *et al*, 2020).

NRC2 and NRC3 from *N. benthamiana* can both support Rpi-amr3 and Rpi-amr1 function, but NRC2 proteins express better in transient assays compared with NRC3 (Derevnina *et al*, 2021). However, co-expression of NRC2-Myc with Rpi-amr3-HF and AVRamr3-V5 in the *nrc2/3/4 N. benthamiana knockout* mutant led to HR in plants and subsequent degradation of Rpi-amr3 and

© 2022 The Authors

AVRamr3, which precluded further analysis (Fig EV2B and C). To avoid cell death, we generated an NRC2 MADA mutant, in which the conserved leucine residues (L9, L13, L17) of NRC2 were mutated into glutamates (NRC2^{EEE}); this NRC2^{EEE} mutant does not lead to HR or protein degradation when co-expressed with Rpi-amr3/AVRamr3 in *nrc2/3/4 knockout N. benthamiana* (Fig EV2B and C). This enabled us to use NRC2^{EEE}-Myc to study the biochemical changes resulting from AVRamr3-dependent Rpi-amr3 activation. Interestingly, co-expression of NRC2^{EEE}-Myc did not alter protein migration patterns of Rpi-amr3-HF or AVRamr3-V5 on blue native-PAGE (Figs 1B and EV2C). The interaction between Rpi-amr3-HF and AVRamr3-V5 was also independent of NRC2^{EEE}-Myc co-expression (Figs 1C and EV2B). NRC2 protein migration was not affected by AVRamr3 co-expression (Fig EV2A). These data indicate that Rpi-amr3 and AVRamr3 form NRC2-independent protein complexes.

We observed that Rpi-amr3 appeared to migrate at ~200 kDa instead of the predicted monomer molecular weight of ~120 kDa in the absence of cognate effector (Figs 1B and EV2C). We tested if either Rpi-amr3 or Rpi-amr1 can self-associate. We co-expressed Rpi-amr1 or Rpi-amr3 fused with Flag/HF or HA tags, immunoprecipitated Rpi-amr1-Flag or Rpi-amr3-HF and detected HA-tagged Rpi-amr signal (Fig EV3A). We found that both Rpi-amr1 and Rpi-amr3 have a very weak capacity to self-associate. However, in comparison to the majority of Rpi-amr1-Flag and Rpi-amr3-HF protein that migrates with a size of ~270 kDa, the co-immunoprecipitated Rpi-amr1-HA or Rpi-amr3-HA migrates slower in blue native-PAGE at a size above ~450 kDa (Fig EV3B–D). This indicates that the majority of the Rpi-amr1 or Rpi-amr3 protein does not self-associate *in vivo*. Furthermore, self-associating Rpi-amr1 and Rpi-amr3 migrate with larger mass than the Rpi-amr1/AVRamr1 or Rpi-amr3/AVRamr3 complexes (Fig EV3B–E). In contrast to Rpi-amr3, migration of Rpi-amr1 on blue native-PAGE is unaltered by cognate effector co-expression (Fig EV3B and C). Thus, the presence or absence of effector does not change self-association of Rpi-amr1 and Rpi-amr3 (Fig EV3D and E). This is consistent with effector-independent self-association observed for other CC-NLRs, such as RPM1 and MLA1 (Maekawa *et al*, 2011; El Kasmi *et al*, 2017). Therefore, we conclude that Rpi-amr1 and Rpi-amr3 protein is predominantly monomeric and forms heterodimers with AVRamr1 or AVRamr3. However, additional interactors with these proteins cannot be excluded. The role (if any) of self-associated Rpi-amr1 or Rpi-amr3 remains unclear.

NRC2 oligomerizes upon AVRamr3-dependent activation of Rpi-amr3 and AVRamr1-dependent activation of Rpi-amr1

Next, using protein lysates of Fig 1C, we observed that, in the pre-activation state, NRC2^{EEE} migrates as a single protein species of ~180 kDa. However, upon co-expression with Rpi-amr3 and AVRamr3, there was a pronounced shift in migration of NRC2 protein to slower migrating form of ~900 kDa (Fig 2A, red asterisk). There were no changes in the size of NRC2^{EEE}-Myc protein itself in SDS–PAGE gels (Fig 2A). This suggests that NRC2 proteins undergo protein oligomerization when Rpi-amr3 recognizes AVRamr3.

To determine whether this phenomenon occurs with other sensor NLRs, we tested migration change of NRC2 in blue native-

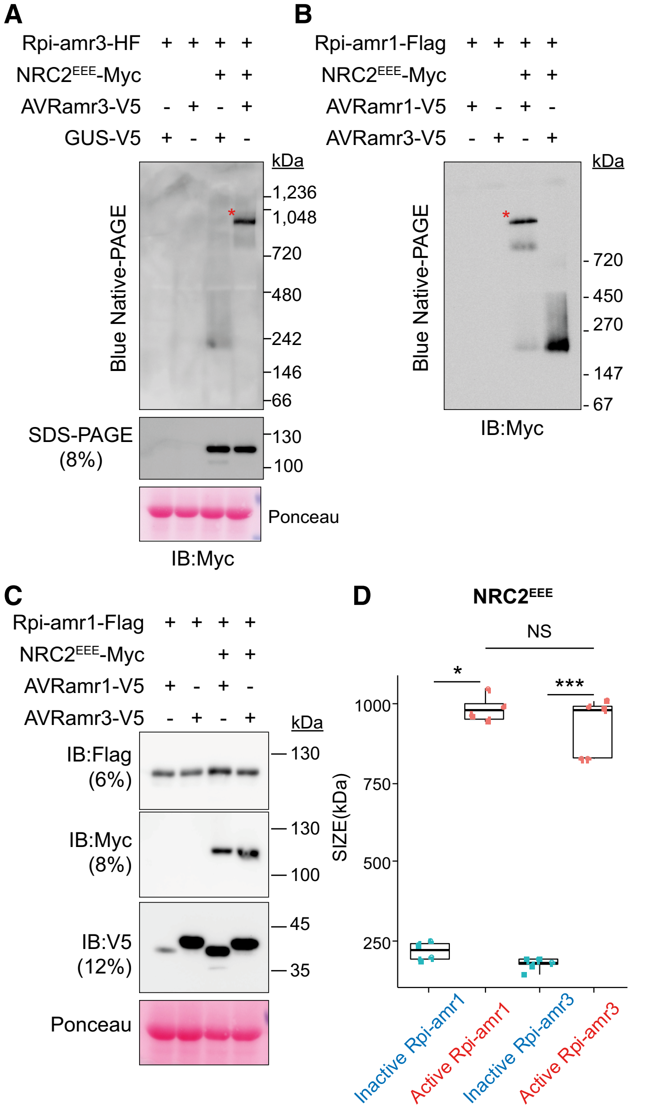

Figure 2. NRC2^{EEE} oligomerizes upon effector detection by Rpi-amr3 or Rpi-amr1.

A NRC2^{EEE}-Myc is oligomerized upon effector-dependent activation of Rpi-amr3. Protein lysates from Fig 1B and C were loaded on blue native-PAGE. SDS-boiled protein lysate samples serve as control for actual size of NRC2^{EEE}-Myc. Oligomerized NRC2^{EEE}-Myc is indicated (*, red).

B NRC2^{EEE}-Myc oligomerizes upon effector-dependent activation of Rpi-amr1. Protein lysates from *nrc2/3/4* knockout *N. benthamiana* plants were loaded on blue native-PAGE. Oligomerized NRC2^{EEE}-Myc is indicated (*, red).

C Samples from (B) were SDS-boiled and loaded on SDS–PAGE. Protein accumulation of Rpi-amr1-Flag, NRC2^{EEE}-Myc, AVRamr1-V5 and AVRamr3-V5 are shown.

D Semi-log plots of NRC2^{EEE}-Myc proteins loaded on blue native-PAGE gels. At least three biological replicates were used for analysis of each sample and data points are plotted on the boxplot (blue, inactive; red, active). Wilcoxon test was conducted in a pairwise manner, and statistical significance is indicated (*, $P < 0.05$; **, $P < 0.01$, ***, $P < 0.001$).

Data information: Ponceau S staining serve as loading control for panels (A and C). Molecular weight markers are shown on the right. Experiments were done with at least three biological replicates with similar results. Standard curves of protein migration derived from semi-log plots of molecular weight markers on individual gels can be found in Dataset EV2.
Source data are available online for this figure.

© 2022 The Authors

PAGE upon Rpi-amr1 activation. As with Rpi-amr3/AVRamr3-mediated NRC2 oligomerization, we observed high-molecular-weight complexes of NRC2^{EEE}-Myc in the presence of AVRamr1 and Rpi-amr1, but not in the presence of AVRamr3 and Rpi-amr1, a non-cognate effector of Rpi-amr1 (Fig 2B and C). Thus, we conclude that although Rpi-amr3 and Rpi-amr1 are different sensor NLRs that recognize AVRamr3 and AVRamr1, respectively, the resulting change of NRC2 is strikingly similar. In the companion paper by preprint: Contreras *et al* (2022), distinct sensor NLRs such as Bs2 and Rx also induce similar changes in NRC2 protein migration on blue native-PAGE. Interestingly, the NRC oligomers formed upon activation of Rpi-amr3 or Rpi-amr1 were indistinguishable in estimated size (Fig 2D). Also, both Rpi-amr3- and Rpi-amr1-dependent NRC2 oligomers migrate at a ~5-fold larger size compared with inactive NRC2 proteins (Fig 2D). This leads to an intriguing hypothesis that sensor NLRs may not be incorporated into helper NLR oligomers.

Helper NLR NRC2 proteins behave differently in terms of oligomerization and subcellular localization from sensor NLRs Rpi-amr3 and Rpi-amr1

To confirm that the high-molecular-weight complex of NRC2 observed upon Rpi-amr1/AVRamr1 or Rpi-amr3/AVRamr3-mediated activation was indeed NRC2, and did not contain the sensor NLR, we conducted 2-dimensional PAGE. We analyzed protein extracts from Agro-infiltrated *N. benthamiana* leaf samples on blue native-PAGE as a first dimension, and then performed SDS–PAGE as a second dimension analysis to dissociate protein complexes into individual protein components (Fig 3A). Without Rpi-amr3 activation, most of the NRC2^{EEE} protein migrated at ~180 kDa, and migration patterns were similar for Rpi-amr3. The majority of the NRC2^{EEE} and Rpi-amr3 proteins were detected in the faster migrating portion (Fig 3B). When AVRamr3 activated Rpi-amr3, NRC2^{EEE}-Myc migrated with a significant shift to ~900 kDa (Fig 3C, red asterisk). Migration of Rpi-amr3 shifted towards ~480 kDa (Fig 3C, blue asterisk), but we did not observe co-migration of Rpi-amr3 with NRC2 oligomer (Fig 3C) at any of the time points we investigated. Our results indicate that the helper NLR NRC2 forms a high-molecular-weight complex upon activation and that this high-molecular-weight NRC2 complex does not contain the sensor NLR Rpi-amr3.

ZAR1 upon activation is known to form a cation channel at the plasma membrane (Bi *et al*, 2021). Therefore, activation of ZAR1 leads to its re-localization to the plasma membrane (Wang *et al*, 2019a). NRC4 was also shown to re-localize to the plasma membrane upon activation (Duggan *et al*, 2021). We tested whether NRC2 activated by Rpi-amr1 or Rpi-amr3 re-localizes by performing membrane fractionation assays. Helper NLR NRC2 was enriched in the pellet (P) fractions upon sensor activation (Fig 3D and E). Interestingly, Rpi-amr1 did not change in localization upon activation, (Fig 3D), whereas Rpi-amr3 was enriched in fraction P upon activation (Fig 3E). Both AVRamr1 and AVRamr3 appeared predominantly in P fractions (Fig 3D and E). This shows that subcellular localization of helper NLRs and sensor NLRs differ, further corroborating the hypothesis that sensor NLRs are not included in the NRC helper NLR complex.

The NB-ARC domain of Rpi-amr3 and NRC2 is required for NRC2 oligomerization upon AVRamr3-dependent activation of Rpi-amr3

The NB-ARC (Nucleotide-binding domain shared by APAF1, R protein and CED-4) domain of NLRs is known to bind the β-phosphate group of ATP and is required for function (Tameling *et al*, 2002; Wang *et al*, 2019a, 2019b). In ZAR1, the replacement of ADP with ATP in this NB-ARC domain is crucial for oligomerization (Wang *et al*, 2019a, 2019b). We tested whether mutation of the ATP-binding activity of Rpi-amr3 and NRC2 impairs NRC2 oligomerization. The K182 residue of Rpi-amr3 (Rpi-amr3 P-loop), and K188 residue of NRC2 (NRC2 P-loop) lie within the P-loop and mutating this residue to alanine (K182A) or arginine (K188R) leads to loss of HR in the presence of AVRamr3 (Fig 4A and B).

We tested for NRC2 oligomerization upon co-expression of either P-loop mutants of Rpi-amr3 or NRC2. Co-expression of AVRamr3-V5 with Rpi-amr3-HF, but not with the Rpi-amr3 P-loop mutant, induced NRC2^{EEE}-Myc oligomerization (Fig 4C). Therefore, the ATP-binding motif of the sensor NLR is required for activating NRC2. We also observed low-abundance NRC2 forms that migrate at intermediate sizes when co-expressed with Rpi-

Figure 3. Rpi-amr3 and Rpi-amr1 are not present in the oligomerized NRC2 protein complex.

A Experimental design for 2D-PAGE (blue native-PAGE/SDS–PAGE). Agro-infiltrated *nrc2/3/4 knockout N. benthamiana* plants were collected at 3 dpi for protein extraction. Protein extracts were loaded on blue native-PAGE (1D) to separate high molecular weight (HMW) protein complexes (hypothesized as a pentamer) from low-molecular-weight (LMW) protein complexes. Subsequently, blue native-PAGE gels were loaded on SDS–PAGE (2D) for separation of protein complexes into individual proteins.

B NRC2^{EEE} and Rpi-amr3 migration in the absence of effector. *N. benthamiana nrc2/3/4 knockout* plants were transiently infiltrated with Rpi-amr3-HF, NRC2^{EEE}-Myc and GUS-V5 followed by 2D-PAGE. NRC2^{EEE}-Myc proteins (*, red) and Rpi-amr3-HF proteins (*, blue) is indicated.

C NRC2^{EEE} and Rpi-amr3 migration in the presence of effector. *N. benthamiana nrc2/3/4 knockout* plants were transiently infiltrated with Rpi-amr3-HF, NRC2^{EEE}-Myc and AVRamr3-V5 followed by 2D-PAGE. NRC2^{EEE}-Myc proteins (*, red) and Rpi-amr3-HF protein (*, blue) is indicated.

D Re-localization of NRC2^{EEE}-Myc upon effector-dependent activation of Rpi-amr1. Lysates of *nrc2/3/4 knockout* plants transiently expressing Rpi-amr1-Flag, NRC2^{EEE}-Myc with AVRamr3-V5 or AVRamr1-V5 were fractionated into total (T), soluble (S), and pellet (P) fractions.

E Re-localization of NRC2^{EEE}-Myc upon effector-dependent activation of Rpi-amr3. Lysates of *nrc2/3/4 knockout* plants transiently expressing Rpi-amr3-HF, NRC2^{EEE}-Myc with AVRamr3-V5 or AVRamr1-V5 were fractionated into total (T), soluble (S), and pellet (P) fractions.

Data information: Protein lysates boiled in SDS were loaded on the same gel as control of protein size (SDS input) for panels (B and C). Molecular weight markers for blue native-PAGE are labeled on top for panels (B and C), and SDS–PAGE markers are labeled on the right. Ponceau S staining of rubisco large subunit serves as control for panels (B and C). MPK6, which localizes mainly to cytosol, and H^+-ATPase, which localize mainly to membrane fractions serve as fractionation control in panels (D and E). Experiments were repeated with three biological replicates with similar results.
Source data are available online for this figure.

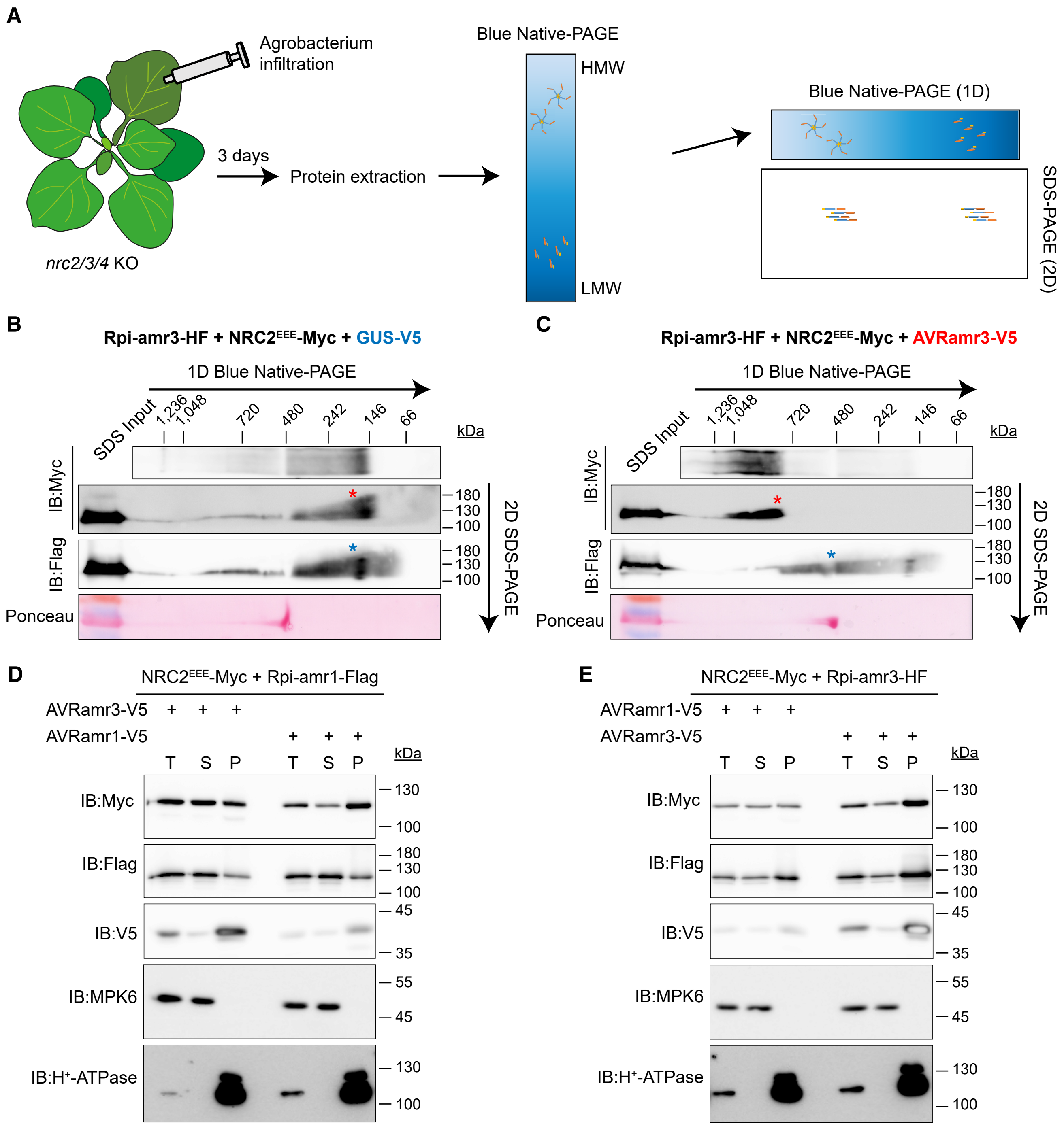

Figure 3.

amr3 P-loop mutant, smaller than those seen upon defense activation by a functional sensor NLR (Fig 4C). Conceivably, these might represent intermediate states of NRC2 activation. Furthermore, the NRC2^{EEE} P-loop mutant also lost its capacity to oligomerize upon sensor NLR activation (Fig 4C). NRC2 was stabilized by co-expression of cognate effector AVRamr3, but we could not observe the oligomerized NRC2 complex as seen with NRC2^{EEE} mutant. This shows that the ATP-binding motif of both sensor and helper NLRs are required for defense activation.

When Rpi-amr3-HF immunoprecipitated samples were used to perform blue native-PAGE, Rpi-amr3 P-loop mutant showed two major protein complexes, indistinguishable from wild-type Rpi-amr3 (Fig EV4A). Co-immunoprecipitated AVRamr3-V5 also migrated to a similar position in the gel as Rpi-amr3-HF and its P-loop mutant (Fig EV4B). Therefore, interaction between Rpi-amr3 and AVRamr3 is not impaired by mutation in the P-loop of Rpi-amr3, indicating that sensor NLR-effector interaction is necessary but insufficient for defense activation.

 © 2022 The Authors

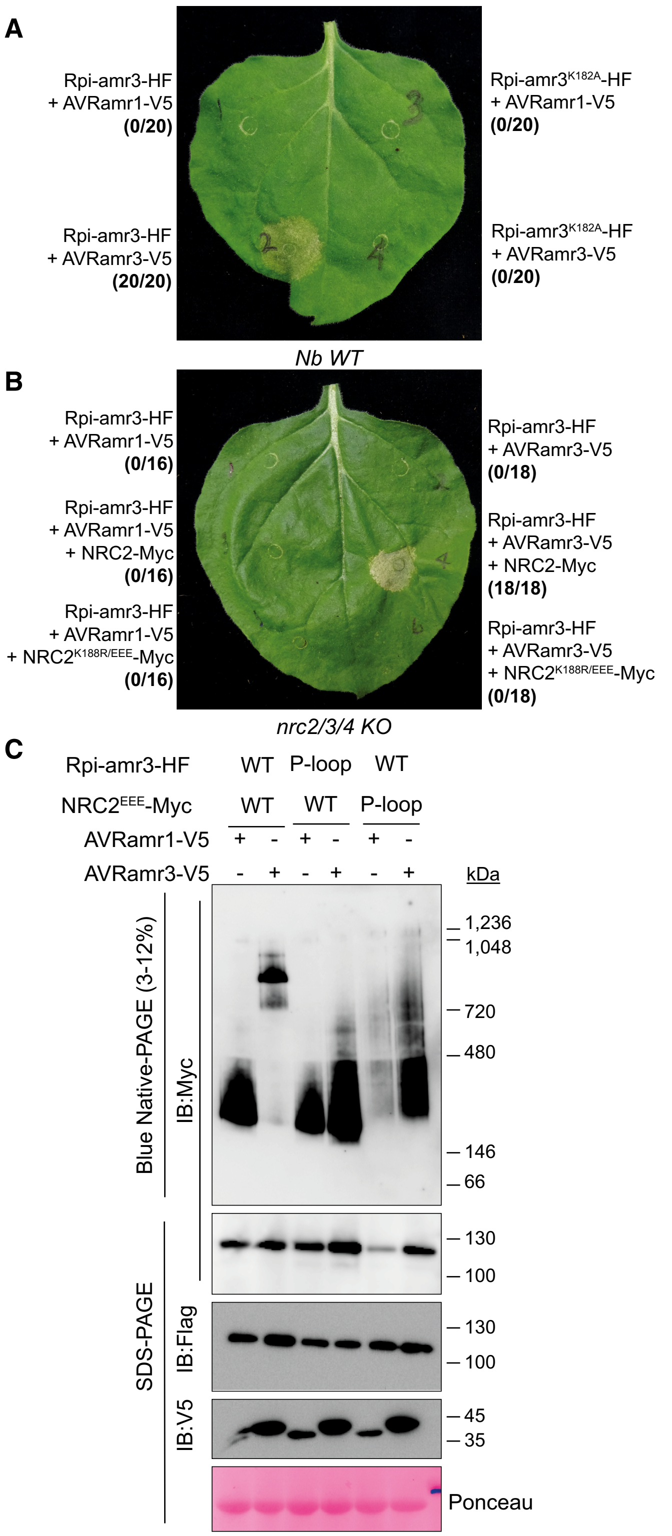

Figure 4. P-loop of Rpi-amr3 and NRC2^{EEE} is required for NRC2^{EEE} oligomerization.

A P-loop of Rpi-amr3 is required for AVRamr3-dependent HR in *N. benthamiana*. Representative leaf phenotype of HR (hypersensitive response) in wild-type *N. benthamiana* is shown. The number of leaves tested and occurrences of HR are indicated in parentheses.

B P-loop of NRC2 is required for AVRamr3-dependent HR in *N. benthamiana nrc2/3/4 knockout* plants. Representative leaf phenotype of HR is shown. The number of leaves tested and occurrences of HR are indicated in parentheses.

C NRC2^{EEE}-Myc requires functional P-loop of both Rpi-amr3 and NRC2 for oligomerization. Protein lysates from *N. benthamiana nrc2/3/4 knockout* plants expressing wild-type or P-loop mutants of Rpi-amr3 or NRC2^{EEE} were loaded for blue native-PAGE. Membranes were immunoblotted with anti-Myc.

Data information: At least three biological replicates with multiple technical replicates were tested for panels (A and B). SDS-boiled samples of the protein lysates were loaded onto SDS–PAGE for control in panel (C). Ponceau S staining serves as loading control. Molecular weight markers are shown on the right. Experiments were done with at least three biological replicates with similar results for panel (C).
Source data are available online for this figure.

NRC4 is oligomerized by effector-dependent activation of Rpi-amr3 but not Rpi-amr1

Solanaceae species have multiple homologs in the NRC helper NLR clade. We have previously shown that Rpi-amr3 signals via NRC2, NRC3, and NRC4 (Lin *et al*, 2022b), whereas Rpi-amr1 signals via NRC2 and NRC3 (Witek *et al*, 2021; Fig 5A). Comparing NRC homologs from different solanaceous species, NRC4 has diverged from the NRC2 and NRC3 clade (preprint: Lin *et al*, 2022a). NRC2 and NRC4 also have different properties in cells. In particular, NRC4 relocalizes to the extrahaustorial membrane upon *P. infestans* infection of *N. benthamiana*, but no change is observed for NRC2 localization (Duggan *et al*, 2021).

To test whether the distinct genetic requirement of helper NLRs by sensor NLRs is correlated with oligomerization, we extracted plant lysates expressing NRC4^{AAA}-Myc upon co-expression with sensor NLRs with and without their cognate effectors. In the absence of activated sensor NLR, NRC4^{AAA} migrates to ~150 kDa (Fig 5B). When Rpi-amr3 and AVRamr3 were co-expressed with NRC4^{AAA}, oligomerization of NRC4^{AAA} was observed. However, co-expression of Rpi-amr1 and AVRamr1 does not induce oligomerization of NRC4^{AAA} (Fig 5B). These data show that sensor NLR activation can only oligomerize helper NLRs that genetically act downstream of effector detection.

Multiple AVRamr3 alleles recognized by Rpi-amr3 trigger NRC2 oligomerization

AVRamr3 is a conserved RXLR effector found in multiple *Phytophthora* species and Rpi-amr3 can recognize multiple homologs of AVRamr3 from different *Phytophthora* species (Lin *et al*, 2022b). For example, AVRamr3 from *P. parasitica* is recognized by Rpi-amr3, but AVRamr3 from *P. capsici* is not recognized by Rpi-amr3 (Lin *et al*, 2022b; Figs 6A and EV5A).

Here, to study whether other recognized AVRamr3 homologs can also activate the NRC2 resistosome, we tested for NRC2 oligomerization upon Rpi-amr3 activation with AVRamr3 alleles from different *Phytophthora* species. NRC2^{EEE}-Myc oligomerizes in the presence of Rpi-amr3-HF with *P. infestans* and *P. parasitica* AVRamr3-GFP (Fig 6B) but not with *P. capsici* AVRamr3-GFP. The inactive truncations of AVRamr3 allele AVRamr3-T9-V5 (Fig EV5A) also did not induce NRC2 oligomerization (Fig EV5B). This shows

© 2022 The Authors

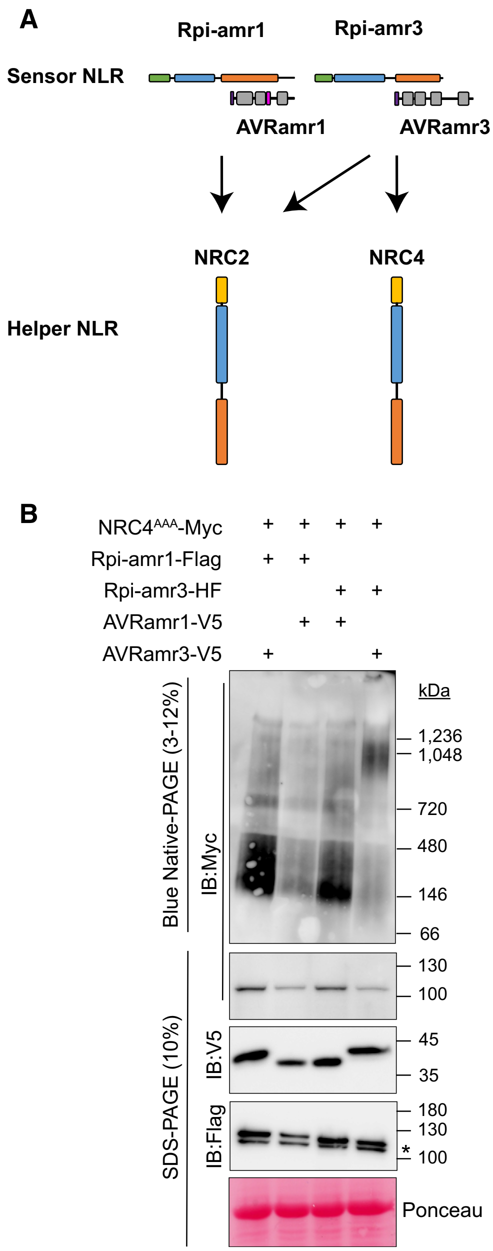

Figure 5. type="main">NRC4^{AAA}-Myc is not oligomerized by activation of Rpi-amr1.

A Cartoon depicting genetic requirement of Rpi-amr1 and Rpi-amr3-dependent activation of NRCs. Arrows are shown to illustrate which NRC helper NLR supports each sensor NLR.

B Protein lysates expressing NRC4^{AAA}-Myc with Rpi-amr3-HF or Rpi-amr1-Flag in the presence and absence of cognate effector were loaded on blue native-PAGE. Proteins were transiently expressed in *N. benthamiana nrc2/3/4 knockout* plants. Non-specific bands are indicated with *.

Data information: At least three biological replicates with technical replicates were tested. SDS-boiled samples of the protein lysates were loaded onto SDS–PAGE for control. Ponceau S staining serves as loading control. Molecular weight markers are shown on the right. Experiments were done with more than three biological replicates with similar results for panel (B).
Source data are available online for this figure.

that NRC2 oligomerization is correlated with recognition of AVRamr3 alleles by Rpi-amr3.

Previously, we showed that the recognized alleles of AVRamr3-GFP from *P. parasitica* could be co-immunoprecipitated by Rpi-amr3-HF, whereas AVRamr3-GFP from *P. capsici* could not be co-immunoprecipitated (Lin *et al*, 2022b). To test whether recognized alleles of AVRamr3 can form a complex with Rpi-amr3, Rpi-amr3-HF was immunoprecipitated from samples co-expressing AVRamr3-GFP from *P. parasitica*, and *P. capsici*. When these immunoprecipitates were analyzed by blue native-PAGE, *P. infestans* AVRamr3-GFP and *P. parasitica* AVRamr3-GFP migrated similarly, at both ~66 kDa and ~300 kDa (Fig 6C). However, no signals were detected for *P. capsici* AVRamr3 on blue native-PAGE, as Rpi-amr3-HF could not co-immunoprecipitate the *P. capsici* allele of AVRamr3 (Figs 6C and EV5C). Rpi-amr3-HF co-expressed with non-recognized alleles of AVRamr3 also migrated as single protein species on blue native-PAGE (Fig EV5D), further confirming that non-recognized homolog or truncation of AVRamr3 do not interact with Rpi-amr3.

Discussion

Here, we report that effector-dependent sensor NLR activation leads to oligomerization of cognate NRC helper NLRs. NRC2 oligomerization upon activation illustrates the conserved mechanism of NLRs in forming high-molecular-weight assemblies upon effector recognition. NRC2 oligomerization is dependent on a functional P-loop in both the sensor and the helper NLR, and on the cognate recognized effector. However, at the time points we analyzed, the sensor NLR does not itself oligomerize upon interaction with the recognized effector. This indicates an important difference in mode of activation between the sensor NLR and the helper NLR.

We used here the transient expression of the *S. americanum* sensor NLRs Rpi-amr1 and Rpi-amr3 with their cognate effectors AVRamr1 and AVRamr3 from *P. infestans* in *N. benthamiana*. NRC proteins from *N. benthamiana* can support HR upon AVRamr3 recognition by Rpi-amr3 and AVRamr1 recognition by Rpi-amr1 (Witek *et al*, 2021; Lin *et al*, 2022b). To prevent cell death upon effector-dependent sensor NLR activation, we used *N. benthamiana* NRC2 mutated within its N-terminal MADA motif. NRC4^{L9E} mutated at the MADA motif activated either in an effector-dependent manner or by autoactive mutation forms punctate structures in the cell membranes (Duggan *et al*, 2021). This implies that the N-terminal MADA motif mutation of L9E/L13E/L17E of NRC2 is likely to only affect any possible channel activity and prevent cell death but should not compromise oligomerization or re-localization to cellular membranes. We show oligomerization and re-localization of NRC2^{EEE}-Myc upon sensor NLR-mediated activation. Many sensor NLRs in the NRC-dependent superclade lack the MADA motif including Rpi-amr1 and Rpi-amr3 (Witek *et al*, 2016, 2021; Adachi *et al*, 2019). Therefore, absence of the MADA motif in sensor NLRs is consistent with our observation that sensor NLRs do not participate in resistosome formation and in potential channel formation in the membrane.

Rpi-amr1 and Rpi-amr3 belong to the NRC-dependent superclade and both can use NRC2 to execute cell death and resistance (Witek

© 2022 The Authors

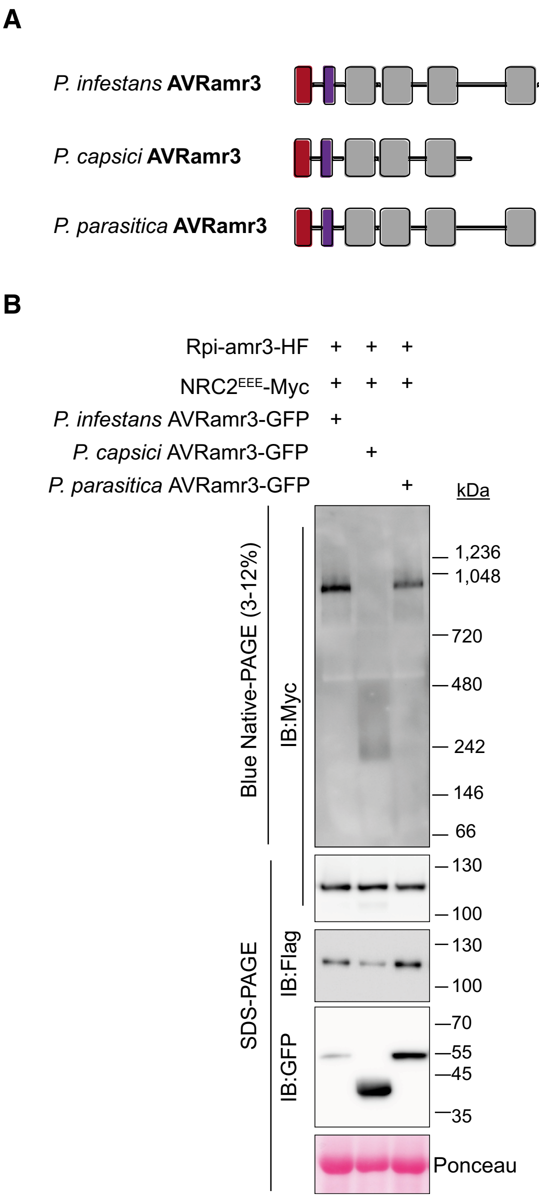

Figure 6. Recognized AVRamr3 alleles from different *Phytophthora* species can trigger oligomerization of NRC2^{EEE}.

A Cartoon depicting different alleles of AVRamr3 from different *Phytophthora* species, *P. infestans*, *P. capsici*, and *P. parasitica*. Response of the corresponding alleles by Rpi-amr3, and thus occurrence of HR is indicated as + (recognition) or – (no recognition).

B NRC2^{EEE}-Myc oligomerizes in response-dependent manner. Protein lysates from *N. benthamiana nrc2/3/4 knockout* transiently expressing NRC2^{EEE}-Myc, Rpi-amr3-HF and AVRamr3 alleles from *P. infestans*, *P. capsici*, and *P. parasitica* were loaded on blue native-PAGE and blotted for NRC2^{EEE} (anti-Myc).

C Recognition is correlated with interaction and protein complex formation of AVRamr3 with Rpi-amr3. Protein extracts from Fig 5B were immunoprecipitated with anti-Flag antibody, separated on blue native-PAGE, and AVRamr3-GFP proteins of *P. infestans*, *P. capsici*, and *P. parasitica* were visualized.

Data information: SDS-boiled input and IP eluates were loaded onto SDS–PAGE as control and blotted for Rpi-amr3 (anti-Flag), and AVRamr3 alleles (anti-GFP). Ponceau S staining serves as loading control. Molecular markers are indicated on the right. Experiments were done with more than three biological replicates with similar results. Source data are available online for this figure.

et al, 2021; Lin *et al*, 2022b). We found that both Rpi-amr1 and Rpi-amr3 interact with their cognate effector independent of NRC2, and this interaction with cognate effector induces NRC2 oligomerization. The NRC2^{EEE}-Myc oligomerization patterns resulting from Rpi-amr1 and Rpi-amr3 activation were indistinguishable (Fig 2B and D). In a companion paper (preprint: Contreras *et al*, 2022), the NRC-dependent CC-NLRs Bs2 and Rx were also shown to activate NRC2 oligomerization into slower migrating forms indistinguishable from what we report here. This indicates that NRC2 oligomerization to ~900 kDa is a universal mode of action for NRC-dependent sensor NLRs, and supports the conclusion that sensor NLRs are not included in the NRC2 resistosome. Our analysis with 2D-PAGE provides further evidence that Rpi-amr3 is not incorporated in the NRC2 resistosome (Fig 3C).

© 2022 The Authors

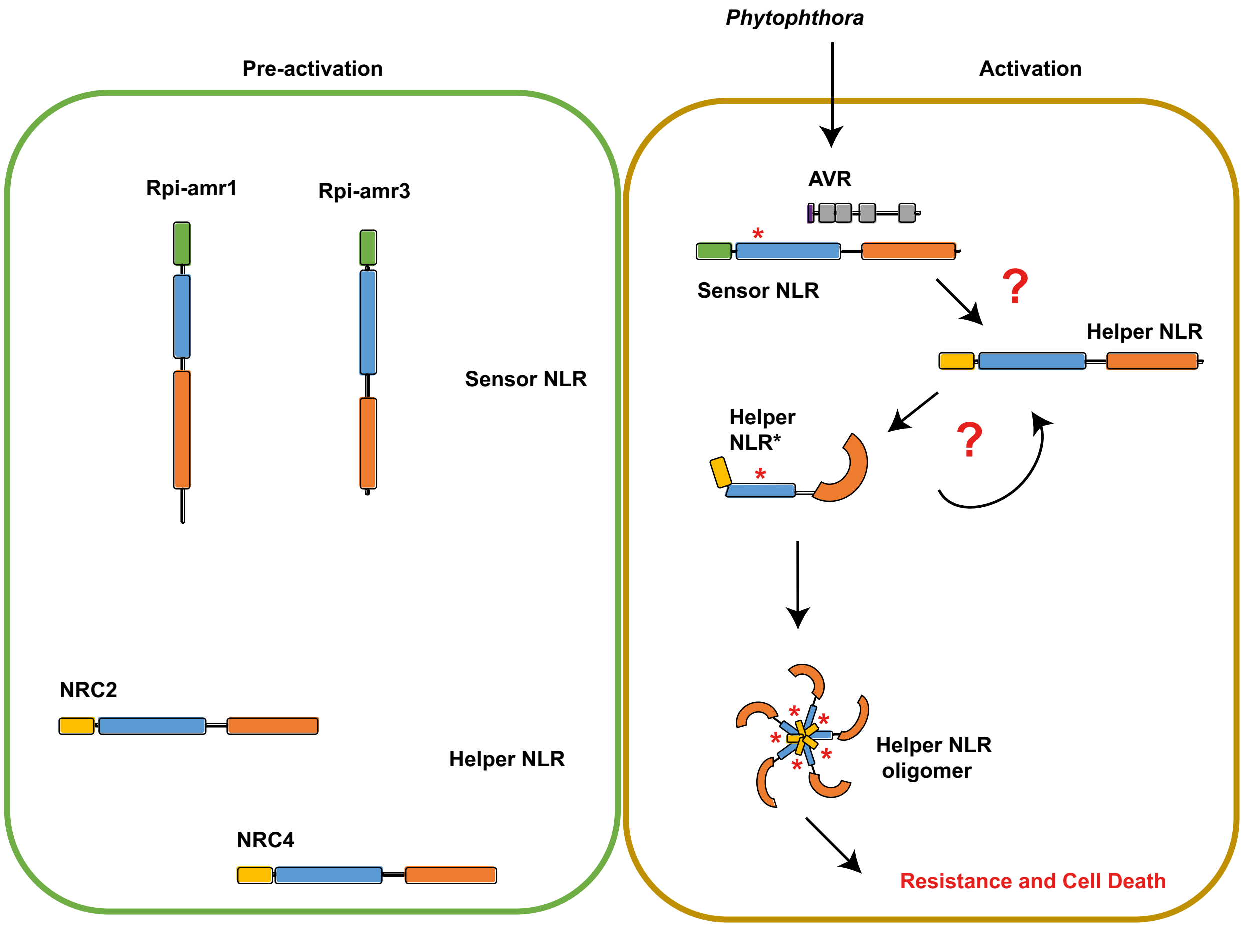

Figure 7. Model for activation of helper NLR NRCs upon recognition of AVRamr by sensor NLRs.

In the pre-activated state, the sensor NLRs, such as Rpi-amr1 and Rpi-amr3, reside in the cells mainly as monomers. Conceivably, additional proteins, such as chaperones, could be bound to these monomeric NLRs. Interaction with AVRamr converts Rpi-amr into an activated form (indicated by an *) that can interact with and activate NRC2 into an activated form. These conversion of NRC2 by activated Rpi-amr may lead to oligomerization, or alternatively, additional NRC2 protomers may be subsequently oligomerized by Rpi-amr-activated NRC2 protomer.

The NB-ARC domain is required for NLR function (Tameling *et al*, 2002). The ATP-binding P-loop motif is known to be required for activation of Rx (Bendahmane *et al*, 2002), another NRC-dependent sensor NLR. We found that similar to Rx, the P-loop motif of Rpi-amr3 is required for effector-dependent cell death. Oligomerization of NRC2^{EEE} in the presence of AVRamr3 and Rpi-amr3 was lost when we tested the P-loop mutant of Rpi-amr3 (Fig 4C). However, we also found that the Rpi-amr3 P-loop mutant retains its interaction with AVRamr3 (Fig EV4A and B). Therefore, a mutation in the P-loop region does not alter the sensor NLR's capacity to interact with its cognate effector, but instead affects its downstream signaling that provokes oligomerization of helper NLRs. We also show that oligomerization of NRC2^{EEE} is lost upon mutation of its P-loop motif. Recent structural analysis of CC-NLRs has shed further light on the role of the NB-ARC domain in its oligomerization. The release of ADP in exchange for ATP is required for the NB-ARC domain-mediated packing of both ZAR1 and Sr35 molecules into resistosomes (Wang *et al*, 2019a; Förderer *et al*, 2022; Zhao *et al*, 2022). Importantly, this indicates that the ATP-binding is required for conformational changes that drive oligomerization of the helper NLR, and the ATP-binding motif of sensor NLR may be involved in the initial activation of conformational change of helper NLRs.

In mammalian cells, NLRC4 is activated by NAIP2-mediated recognition of bacterial flagellin, or NAIP5/6-mediated recognition of bacterial PrgJ (Zhao *et al*, 2011). The NAIP is incorporated into the inflammasome and co-migrates with NLRC4 in non-denaturing PAGE (Kofoed & Vance, 2011). Ligand-bound NAIP undergoes a conformational change that leads to interaction and subsequent intermolecular autoactivation of NLRC4 (Hu *et al*, 2015; Zhang *et al*, 2015). This may explain the near-complete conversion of NLRC4 molecules to inflammasome (Kofoed & Vance, 2011). We also observed near complete conversion of the majority of NRC2 molecules into oligomers (Figs 2A and B, and 3C). This may indicate that the conformational change in NRC2 upon Rpi-amr3 activation is similar to NLRC4 oligomerization. The conformational change of NRC2 induced by Rpi-amr3/AVRamr3 protein complex may trigger self-propagating "chain reaction" of interaction and formation of a

 © 2022 The Authors

complete NRC2 resistosome. On the other hand, it is also conceivable that each NRC requires a sensor NLR to activate it prior to assembly into a resistosome. These two possibilities are highlighted in Fig 7. However, we favor the hypothesis that a transient interaction of activated Rpi-amr3 with NRC2 converts inactive NRC2 into activated NRC2 that can subsequently activate additional NRC2 protomers that in turn can activate additional inactive NRC2 protomer in a chain reaction. This is consistent with the near-complete conversion of $NRC2^{EEE}$ protomers into the oligomeric form upon activation. The ATP-binding P-loop motif of both sensor and helper NLR is required for activation of NRC2, conceivably due to the conformational change of the NB-ARC domain required for Rpi-amr3-mediated activation of NRC2 and its activation of other NRC2 proteins. By analogy with ZAR1 (Wang *et al*, 2019a), the activated NRC2 may form a pentamer, but further work will be required to establish this beyond doubt.

There are multiple NRC paralogs in Solanaceae species, and not all paralogs are conserved in each species. Each sensor NLR may be supported by a different suite of NRC paralogs (Wu *et al*, 2017). Rpi-amr1 signals via NRC2 and NRC3 (Witek *et al*, 2021), whereas Rpi-amr3 signals via NRC2, NRC3 and NRC4 in *N. benthamiana* (Lin *et al*, 2022b). We show here that effector-dependent activation of Rpi-amr1 is not supported by *N. benthamiana* NRC4 (Fig 5B). This indicates that sensor NLR activation can provoke oligomerization of specific paralogs of helper NLRs. Several effectors that target and suppress NRC helper NLRs were discovered from multiple pathogens (Derevnina *et al*, 2021). Interestingly, the evolution of NRC NLRs in *S. americanum* is distinct from that in *N. benthamiana*, particularly in the expansion of NRC4 paralogs, For example, NRC1 is absent in *N. benthamiana*, and NRC1 from *S. americanum* supports Rpi-amr3 but not Rpi-amr1 (preprint: Lin *et al*, 2022a).

Unlike some genes encoding surface immune receptors, e.g. *EFR*, which retain function when transferred between plant families (Lacombe *et al*, 2010), NLR based resistance engineering can be constrained by restricted taxonomic functionality (RTF; Tai *et al*, 1999). In many cases, *NLR*-encoding genes can only confer resistance in closely related plant species. For example, the NRC-dependent bacterial spot disease resistance gene *Bs2* from pepper is not functional in *Arabidopsis* (Tai *et al*, 1999). The identification of the NRC network (Wu *et al*, 2017) and our finding on NRC activation suggests approaches to break RTF and enable breeders to deploy *NLR* genes across different plant families. Co-delivering of *Rpi-amr3* and *NRC* genes into plants that lack NRCs might help to protect them against other *Phytophthora* pathogens. Understanding the mechanism of helper NLR-dependent immune activation might greatly enrich the breeders' toolbox by assisting redeployment of cloned *NLR* genes against different diseases in different crops.

Materials and Methods

Plant materials and growth conditions

The wild-type *Nicotiana benthamiana* and *NRC2*, *NRC3* and *NRC4* knockout *N. benthamiana* line *nrc2/3/4.210.4.3* were used in this study (Wu *et al*, 2020). The plants were grown in a controlled environment room (CER), with 16 h photoperiod, at 22°C and 45–65% humidity.

Constructs

To clone the genes with different tags, all the CDS (coding sequences) without stop codon were cloned into a golden gate compatible level 0 vector (pICSL01005). Then these were fused with different C-terminus tags and shuffled into a binary vector pICSL86977OD (with 35S promoter and Ocs Terminator). The C-terminus tags used in this study are C-HisFlag (PICSL50001), C-V5 (PICSL50012), C-Myc (PICSL50010), C-HA (PICSL50009), C-GFP (PICSL50008), C-3xFlag (PICSL50007), NLUC-Flag (pICSL50047) and CLUC-Flag (pICSL50048). All the constructs used in this study are listed in Dataset EV1. The NRC^{EEE}-Myc construct was cloned into pJK268c (Kourelis *et al*, 2020).

Agrobacterium infiltration

The binary constructs were transformed into *Agrobacterium* strain GV3101-pMP90 and stored in a −80°C freezer with 20% glycerol. Two days before the *Agrobacterium* infiltration, the constructs were streaked out on solid L medium plate (with kanamycin and rifampicin) and grown in a 28°C incubator. For the *Agrobacterium* infiltration, 1 mM acetosyringone were added into the infiltration buffer ($MgCl_2$-MES, 10 mM $MgCl_2$ and 10 mM MES, pH 5.6), then the *Agrobacterium* were re-suspended into infiltration buffer, the OD_{600} was adjusted to 0.5 and the infiltration was performed 1 h later. For the co-expression experiments, the *Agrobacterium* suspension were equally mixed before infiltration.

HR assay

For the HR assay, 4-week-old *N. benthamiana* were used, the constructs in *Agrobacterium* were infiltrated or co-infiltrated into the abaxial surface of *N. benthamiana* leaves. The HR phenotype were scored and the photos were taken 3–4 days post *Agrobacterium* infiltration (dpi).

Split luciferase assay

The split-luciferase assay was described previously (Lin *et al*, 2022b). In brief, p35S::*Rpi-amr1*-Cluc::OcsT and p35S::*Avramr1*-Nluc::OcsT constructs were made and transformed into *Agrobacterium* strain GV3101-pMP90. p35S::*Rpi-amr3*-Cluc::OcsT and p35S::*Avramr3*-Nluc::OcsT constructs were used as controls. The constructs were expressed or co-expressed in *nrc2/3/4* knockout *N. benthamiana* plants, OD_{600} = 0.5. The leaves were infiltrated with 0.4 mM luciferin on 100 mM sodium citrate buffer (pH 5.6) at 3 dpi, then the leaves were picked for imaging with NightOWL II LB 983 *In Vivo* Imaging System. Two leaves were used for each test and three independent experiments were performed with same results.

Structure prediction using AlphaFold

Protein structure of AVRamr1 was predicted using AlphaFold (Jumper *et al*, 2021), via https://github.com/deepmind/alphafold/. The structure was visualized with ChimeraX (Pettersen *et al*, 2020), developed by the Resource for Biocomputing, Visualization, and Informatics at the University of California, San Francisco, with support from National Institutes of Health R01-GM129325 and the

© 2022 The Authors

Office of Cyber Infrastructure and Computational Biology, National Institute of Allergy and Infectious Diseases.

Protein extraction

Agrobacterium-infiltrated leaves were sampled with cork borer 3 dpi. Ten disks were collected for each sample and put into 2-ml eppendorf tubes with two tungsten beads, which were immediately frozen in liquid nitrogen. Frozen samples were then ground in Geno/Grinder® (SPEX SamplePrep) at 1,200 rpm for 2 min. Protein extraction buffer for NRC2 (Tris-Cl pH 7.5 50 mM, NaCl 50 mM, Glycerol 10%, $MgCl_2$ 5 mM, 10 mM DTT, 0.2% NP-40, protease inhibitor cocktail) and NRC4 (HEPES pH 7.5 50 mM, NaCl 50 mM, Glycerol 10%, $MgCl_2$ 5 mM, 10 mM DTT, 1% Digitonin, protease inhibitor cocktail) was added in equal volume across samples to ensure equal protein concentration. Stock concentration of 10% Digitonin was dissolved in DMSO. Centrifugation was performed at 18,000 *g* for 15 and 5 min subsequently to remove cell debris. For subsequent blue native-PAGE analysis, sample aliquots were immediately frozen in liquid nitrogen.

Immunoprecipitation and elution

Flag-M2 beads (Sigma, A2220) were added to the protein extract after centrifugation and incubated at 4°C for 2 h. After incubation, washing step with protein extraction buffer was performed five times (two times with extraction buffer containing NP-40 0.4%, then three times with extraction buffer containing NP-40 0.2%) to ensure removal of non-specific binding proteins. For elution of proteins, 3xFlag peptide (Sigma, F4799) were added to beads at concentration of 0.2 mg/ml and incubated for 1 h. Sample aliquots were made for blue native-PAGE analysis and were immediately frozen in liquid nitrogen.

SDS–PAGE and immunoblot

Protein samples were incubated at 70°C for 10 min after adding 3× SDS sample buffer (stock concentration 30% glycerol, 3% SDS, 93.75 mM Tris-Cl pH 6.8, 0.06% bromophenol blue). These samples were loaded on SDS–PAGE gels (8 or 12%) and run at 90 V. After dye front reached the end, these gels were transferred with Trans-Blot (Biorad) at conditions of 1.0 mA for 30 min onto PVDF membranes. Transferred membranes were blocked with 5% skim milk in TBST, and antibodies were added subsequently and incubated overnight at 4°C. The following antibodies were used; Flag-HRP (Sigma, A8592), Myc-HRP (Sigma, 16-213), V5-HRP (Sigma, V2260), HA-HRP (Roche, 12013819001), MPK6 (Agrisera, AS12 2633), H^+-ATPase (Agrisera, AS07 260), and GFP-HRP (Abcam, ab6663). Signals were detected using ECL substrates (Thermo Fisher, 34580). After detection, membranes were stained with Ponceau S solution (Sigma, P7170) to use as loading control. PageRuler™ Prestained Protein Ladder (Thermo Scientific, 26616) was used as molecular weight markers.

Blue native-PAGE

Blue native-PAGE was performed as indicated by the manufacturer. Protein extracts and immunoprecipitation (IP) eluates were added with 4× NativePAGE™ Sample Buffer (Invitrogen™, BN2003) and NativePAGE™ 5% G-250 Sample Additive (Invitrogen™, BN2004) to a final concentration of 0.125%. Then, samples were loaded on NativePAGE™ Novex® 3–12% Bis-Tris Gels (Invitrogen™, BN1001) and run at 150 V in cathode buffer containing Coomassie G-250 (by adding NativePAGE™ Cathode Buffer Additive to 1/200 dilution, Invitrogen™, BN2002 to NativePAGE™ Running Buffer, Invitrogen™, BN2001). NativeMark™ Unstained Protein Standard (Invitrogen™, LC0725) or SERVA Native Marker (SERVA, 39219.01) were loaded to predict the size of detected protein species.

2D-PAGE

Samples were run on blue native-PAGE and gel strips were cut for each lane. Gel strips were put in 15 ml conical tubes and incubated with 3× SDS sample buffer containing 50 mM DTT for 15 min. These gel strips are then loaded onto 8% SDS–PAGE resolution gel. Corresponding protein extracts prepared for SDS–PAGE were loaded together to serve as control. After SDS–PAGE, transfer to PVDF membranes and immunoblots were performed as described above.

Membrane fractionation

Membrane fractionation was performed as described (Abas & Luschnig, 2010). Agrobacterium-infiltrated leaf samples (10 leaf disks) were frozen in liquid nitrogen and then ground in Geno/Grinder® (SPEX SamplePrep) at 1,200 rpm for 2 min. 1.7 ml Fractionation buffer (50 mM Tris-Cl pH 7.5, 250 mM Sucrose, 5% Glycerol, 5 mM MgCl2, 50 mM NaCl, 0.5% PVPP, 10 mM DTT, protease inhibitor) was added and samples were precleared at 600 *g* for 3 min. Supernatant was collected and equal volume of water was added. Total (T) samples were taken. Diluted supernatants were aliquoted into 200 μl then centrifuged at 21,000 *g* for 1 h. After centrifugation, the supernatant (Soluble, S) samples were taken. The remaining Pellet (P) samples were washed once more and resuspended for further use.

Statistical analysis

For each blue native-PAGE gel, the migrating distance of molecular weight markers were measured and plotted with Log10 value of its molecular weight. All standard curve generated has R^2 values of above 0.95 (Dataset EV2). Then, the migration of different protein complexes was plotted onto these standard curves to estimate the size of each protein complex. At least three biological replicates were measured, and non-parametric Wilcoxon test was used and statistical significance in p values are indicated. Data points that are not within the range of standard curves were omitted. No statistical methods were used to estimate sample size, and blinding was not applied. R Studio was used to generate plots and for statistical tests.

Data availability

Reagents, tools, and materials generated in this study are available from the corresponding author upon request.

Expanded View for this article is available online.

 © 2022 The Authors

Acknowledgements

We thank members of the Jones lab and the Kamoun lab for their helpful comments and discussions. H-KA thanks Adam Bentham for his support on protein structure prediction data analysis. We would like to thank Timothy Wells, from Horticultural Services at John Innes Centre, for excellent care of the plants. We also wish to thank the TSL Synbio team and the TSL bioinformatics team for their support as well as all TSL support services. H-KA was supported by the ERC Advanced Grant "ImmunitybyPairDesign". XL and ACO-A were supported by BBSRC grant BB/P021646/1. JDGJ received core funding from Gatsby Charitable Foundation.

Author contributions

Hee-Kyung Ahn: Conceptualization; resources; data curation; formal analysis; validation; investigation; visualization; methodology; writing – original draft; writing – review and editing. **Xiao Lin:** Conceptualization; resources; data curation; formal analysis; investigation; visualization; methodology; writing – original draft; writing – review and editing. **Andrea Carolina Olave-Achury:** Data curation; formal analysis; validation; investigation; writing – review and editing. **Lida Derevnina:** Conceptualization; resources; methodology; writing – review and editing. **Mauricio P Contreras:** Resources; formal analysis; investigation; methodology; writing – review and editing. **Jiorgos Kourelis:** Resources; formal analysis; investigation; methodology; writing – review and editing. **Chih-Hang Wu:** Resources; methodology. **Sophien Kamoun:** Supervision; project administration; writing – review and editing. **Jonathan DG Jones:** Resources; supervision; funding acquisition; project administration; writing – review and editing.

Disclosure and competing interests statement

SK and JK receives funding from industry and has filed patents on NLR biology.

References

Abas L, Luschnig C (2010) Maximum yields of microsomal-type membranes from small amounts of plant material without requiring ultracentrifugation. *Anal Biochem* 401: 217–227

Adachi H, Contreras MP, Harant A, Wu CH, Derevnina L, Sakai T, Duggan C, Moratto E, Bozkurt TO, Maqbool A *et al* (2019) An N-terminal motif in NLR immune receptors is functionally conserved across distantly related plant species. *Elife* 8: e49956

Barragan AC, Weigel D (2021) Plant NLR diversity: the known unknowns of pan-NLRomes. *Plant Cell* 33: 814–831

Bendahmane A, Farnham G, Moffett P, Baulcombe DC (2002) Constitutive gain-of-function mutants in a nucleotide binding site-leucine rich repeat protein encoded at the Rx locus of potato. *Plant J* 32: 195–204

Bi G, Su M, Li N, Liang Y, Dang S, Xu J, Hu M, Wang J, Zou M, Deng Y *et al* (2021) The ZAR1 resistosome is a calcium-permeable channel triggering plant immune signaling. *Cell* 184: 3528–3541

Boutemy LS, King SRF, Win J, Hughes RK, Clarke TA, Blumenschein TMA, Kamoun S, Banfield MJ (2011) Structures of *Phytophthora* RXLR effector proteins: a conserved but adaptable fold underpins functional diversity. *J Biol Chem* 286: 35834–35842

Castel B, Ngou PM, Cevik V, Redkar A, Kim DS, Yang Y, Ding P, Jones JDG (2019) Diverse NLR immune receptors activate defence via the RPW8-NLR NRG1. *New Phytol* 222: 966–980

Cesari S, Bernoux M, Moncuquet P, Kroj T, Dodds PN (2014) A novel conserved mechanism for plant NLR protein pairs: the "integrated decoy" hypothesis. *Front Plant Sci* 5: 606

Collier SM, Hamel LP, Moffett P (2011) Cell death mediated by the N-terminal domains of a unique and highly conserved class of NB-LRR protein. *Mol Plant Microbe Interact* 24: 918–931

Contreras MP, Pai H, Tumtas Y, Duggan C, Him Yuen EL, Cruces AV, Kourelis J, Ahn H-K, Wu C-H, Bozkurt TO *et al* (2022) Sensor NLR immune proteins activate oligomerization of their NRC helper. *bioRxiv* https://doi.org/10.1101/2022.04.25.489342 [PREPRINT]

Cooke LR, Schepers HTAM, Hermansen A, Bain RA, Bradshaw NJ, Ritchie F, Shaw DS, Evenhuis A, Kessel GJT, Wander JGN *et al* (2011) Epidemiology and integrated control of potato late blight in Europe. *Potato Res* 54: 183–222

Derevnina L, Contreras MP, Adachi H, Upson J, Vergara Cruces A, Xie R, Sklenar J, Menke FLH, Mugford ST, MacLean D *et al* (2021) Plant pathogens convergently evolved to counteract redundant nodes of an NLR immune receptor network. *PLoS Biol* 19: e3001136

Duggan C, Moratto E, Savage Z, Hamilton E, Adachi H, Wu CH, Leary AY, Tumtas Y, Rothery SM, Maqbool A *et al* (2021) Dynamic localization of a helper NLR at the plant-pathogen interface underpins pathogen recognition. *Proc Natl Acad Sci USA* 118: e2104997118

El Kasmi F, Chung EH, Anderson RG, Li J, Wan L, Eitas TK, Gao Z, Dangl JL (2017) Signaling from the plasma-membrane localized plant immune receptor RPM1 requires self-association of the full-length protein. *Proc Natl Acad Sci USA* 114: E7385–E7394

Eubel H, Braun HP, Millar AH (2005) Blue-native PAGE in plants: a tool in analysis of protein-protein interactions. *Plant Methods* 1: 11

Feehan JM, Castel B, Bentham AR, Jones JD (2020) Plant NLRs get by with a little help from their friends. *Curr Opin Plant Biol* 56: 99–108

Feehan JM, Wang J, Sun X, Choi J, Ahn H-K, Ngou BPM, Parker JE, Jones JDG (2022) Oligomerisation of a plant helper NLR requires cell-surface and intracellular immune receptor activation. *bioRxiv* https://doi.org/10.1101/2022.06.16.496440 [PREPRINT]

Förderer A, Li E, Lawson AW, Deng Y, Sun Y, Logemann E, Zhang X, Wen J, Han Z, Chang J *et al* (2022) A wheat resistosome defines common principles of immune receptor channels. *Nature* 610: 532–539

He J, Ye W, Choi DS, Wu B, Zhai Y, Guo B, Duan S, Wang Y, Gan J, Ma W *et al* (2019) Structural analysis of *Phytophthora* suppressor of RNA silencing 2 (PSR2) reveals a conserved modular fold contributing to virulence. *Proc Natl Acad Sci USA* 116: 8054–8059

Hu Z, Zhou Q, Zhang C, Fan S, Cheng W, Zhao Y, Shao F, Wang HW, Sui SF, Chai J (2015) Structural and biochemical basis for induced self-propagation of NLRC4. *Science* 350: 399–404

Hu M, Qi J, Bi G, Zhou JM (2020) Bacterial effectors induce Oligomerization of immune receptor ZAR1 in vivo. *Mol Plant* 13: 793–801

Huang S, Jia A, Song W, Hessler G, Meng Y, Sun Y, Xu L, Laessle H, Jirschitzka J, Ma S *et al* (2022) Identification and receptor mechanism of TIR-catalyzed small molecules in plant immunity. *Science* 377: eabq3297

Jacob P, Kim NH, Wu F, El-Kasmi F, Chi Y, Walton WG, Furzer OJ, Lietzan AD, Sunil S, Kempthorn K *et al* (2021) Plant "helper" immune receptors are Ca (2+)-permeable nonselective cation channels. *Science* 373: 420–425

Jia A, Huang S, Song W, Wang J, Meng Y, Sun Y, Xu L, Laessle H, Jirschitzka J, Hou J *et al* (2022) TIR-catalyzed ADP-ribosylation reactions produce signaling molecules for plant immunity. *Science* 377: eabq8180

Jones JD, Vance RE, Dangl JL (2016) Intracellular innate immune surveillance devices in plants and animals. *Science* 354: aaf6395

© 2022 The Authors

Jubic LM, Saile S, Furzer OJ, El Kasmi F, Dangl JL (2019) Help wanted: helper NLRs and plant immune responses. *Curr Opin Plant Biol* 50: 82 – 94

Jumper J, Evans R, Pritzel A, Green T, Figurnov M, Ronneberger O, Tunyasuvunakool K, Bates R, Zidek A, Potapenko A *et al* (2021) Highly accurate protein structure prediction with AlphaFold. *Nature* 596: 583 – 589

Kamoun S, Furzer O, Jones JD, Judelson HS, Ali GS, Dalio RJ, Roy SG, Schena L, Zambounis A, Panabieres F *et al* (2015) The top 10 oomycete pathogens in molecular plant pathology. *Mol Plant Pathol* 16: 413 – 434

Kofoed EM, Vance RE (2011) Innate immune recognition of bacterial ligands by NAIPs determines inflammasome specificity. *Nature* 477: 592 – 595

Kourelis J, Contreras MP, Harant A, Pai H, Lüdke D, Adachi H, Derevnina L, Wu C-H, Kamoun S (2022) The helper NLR immune protein NRC3 mediates the hypersensitive cell death caused by the cell-surface receptor Cf-4. *PLoS Genet* 18: e1010414

Kourelis J, Malik S, Mattinson O, Krauter S, Kahlon PS, Paulus JK, van der Hoorn RAL (2020) Evolution of a guarded decoy protease and its receptor in solanaceous plants. *Nat Commun* 11: 4393

Lacombe S, Rougon-Cardoso A, Sherwood E, Peeters N, Dahlbeck D, van Esse HP, Smoker M, Rallapalli G, Thomma BPHJ, Staskawicz B *et al* (2010) Interfamily transfer of a plant pattern-recognition receptor confers broad-spectrum bacterial resistance. *Nat Biotechnol* 28: 365 – 369

Lee DH, Lee HS, Belkhadir Y (2021) Coding of plant immune signals by surface receptors. *Curr Opin Plant Biol* 62: 102044

Li J, Huang H, Zhu M, Huang S, Zhang W, Dinesh-Kumar SP, Tao X (2019) A plant immune receptor adopts a two-step recognition mechanism to enhance viral effector perception. *Mol Plant* 12: 248 – 262

Lin X, Song T, Fairhead S, Witek K, Jouet A, Jupe F, Witek AI, Karki HS, Vleeshouwers V, Hein I *et al* (2020) Identification of Avramr1 from *Phytophthora infestans* using long read and cDNA pathogen-enrichment sequencing (PenSeq). *Mol Plant Pathol* 21: 1502 – 1512

Lin X, Jia Y, Heal R, Prokchorchik M, Sindalovskaya M, Olave-Achury A, Makechemu M, Fairhead S, Noureen A, Heo J *et al* (2022a) The *Solanum americanum* pangenome and effectoromics reveal new resistance genes against potato late blight. *bioRxiv* https://doi.org/10.1101/2022.08.11.503608 [PREPRINT]

Lin X, Olave-Achury A, Heal R, Pais M, Witek K, Ahn HK, Zhao H, Bhanvadia S, Karki HS, Song T *et al* (2022b) A potato late blight resistance gene protects against multiple *Phytophthora* species by recognizing a broadly conserved RXLR-WY effector. *Mol Plant* 15: 1457 – 1469

Ma S, Lapin D, Liu L, Sun Y, Song W, Zhang X, Logemann E, Yu D, Wang J, Jirschitzka J *et al* (2020) Direct pathogen-induced assembly of an NLR immune receptor complex to form a holoenzyme. *Science* 370: eabe3069

Maekawa T, Cheng W, Spiridon LN, Toller A, Lukasik E, Saijo Y, Liu P, Shen QH, Micluta MA, Somssich IE *et al* (2011) Coiled-coil domain-dependent homodimerization of intracellular barley immune receptors defines a minimal functional module for triggering cell death. *Cell Host Microbe* 9: 187 – 199

Martin R, Qi T, Zhang H, Liu F, King M, Toth C, Nogales E, Staskawicz BJ (2020) Structure of the activated ROQ1 resistosome directly recognizing the pathogen effector XopQ. *Science* 370: eabd9993

Meyers BC, Dickerman AW, Michelmore RW, Sivaramakrishnan S, Sobral BW, Young ND (1999) Plant disease resistance genes encode members of an ancient and diverse protein family within the nucleotide-binding superfamily. *Plant J* 20: 317 – 332

Na Ayutthaya PP, Lundberg D, Weigel D, Li L (2020) Blue native polyacrylamide gel electrophoresis (BN-PAGE) for the analysis of protein oligomers in plants. *Curr Protoc Plant Biol* 5: e20107

Ngou BPM, Ding P, Jones JD (2022a) Thirty years of resistance: zig-Zag through the plant immune system. *Plant Cell* 34: 1447 – 1478

Ngou BPM, Heal R, Wyler M, Schmid MW, Jones JDG (2022b) Concerted expansion and contraction of immune receptor gene repertoires in plant genomes. *Nat Plants* 8: 1146 – 1152

Peart JR, Mestre P, Lu R, Malcuit I, Baulcombe DC (2005) NRG1, a CC-NB-LRR protein, together with N, a TIR-NB-LRR protein, mediates resistance against tobacco mosaic virus. *Curr Biol* 15: 968 – 973

Pettersen EF, Goddard TD, Huang CC, Meng EC, Couch GS, Croll TI, Morris JH, Ferrin TE (2020) UCSF ChimeraX: structure visualization for researchers, educators, and developers. *Protein Sci* 30: 70 – 82

Qi T, Seong K, Thomazella DPT, Kim JR, Pham J, Seo E, Cho MJ, Schultink A, Staskawicz BJ (2018) NRG1 functions downstream of EDS1 to regulate TIR-NLR-mediated plant immunity in *Nicotiana benthamiana*. *Proc Natl Acad Sci USA* 115: E10979 – E10987

Saile SC, Jacob P, Castel B, Jubic LM, Salas-Gonzales I, Backer M, Jones JDG, Dangl JL, El Kasmi F (2020) Two unequally redundant "helper" immune receptor families mediate *Arabidopsis thaliana* intracellular "sensor" immune receptor functions. *PLoS Biol* 18: e3000783

Tai TH, Dahlbeck D, Clark ET, Gajiwala P, Pasion R, Whalen MC, Stall RE, Staskawicz BJ (1999) Expression of the Bs2 pepper gene confers resistance to bacterial spot disease in tomato. *Proc Natl Acad Sci USA* 96: 14153 – 14158

Tameling WI, Elzinga SD, Darmin PS, Vossen JH, Takken FL, Haring MA, Cornelissen BJ (2002) The tomato R gene products I-2 and MI-1 are functional ATP binding proteins with ATPase activity. *Plant Cell* 14: 2929 – 2939

Vleeshouwers VG, Raffaele S, Vossen JH, Champouret N, Oliva R, Segretin ME, Rietman H, Cano LM, Lokossou A, Kessel G *et al* (2011) Understanding and exploiting late blight resistance in the age of effectors. *Annu Rev Phytopathol* 49: 507 – 531

Wang J, Hu M, Wang J, Qi J, Han Z, Wang G, Qi Y, Wang HW, Zhou JM, Chai J (2019a) Reconstitution and structure of a plant NLR resistosome conferring immunity. *Science* 364: eaav5870

Wang J, Wang J, Hu M, Wu S, Qi J, Wang G, Han Z, Qi Y, Gao N, Wang HW *et al* (2019b) Ligand-triggered allosteric ADP release primes a plant NLR complex. *Science* 364: eaav5868

Witek K, Jupe F, Witek AI, Baker D, Clark MD, Jones JD (2016) Accelerated cloning of a potato late blight-resistance gene using RenSeq and SMRT sequencing. *Nat Biotechnol* 34: 656 – 660

Witek K, Lin X, Karki HS, Jupe F, Witek AI, Steuernagel B, Stam R, van Oosterhout C, Fairhead S, Heal R *et al* (2021) A complex resistance locus in *Solanum americanum* recognizes a conserved *Phytophthora* effector. *Nat Plants* 7: 198 – 208

Wittig I, Beckhaus T, Wumaier Z, Karas M, Schagger H (2010) Mass estimation of native proteins by blue native electrophoresis: principles and practical hints. *Mol Cell Proteomics* 9: 2149 – 2161

Wu CH, Abd-El-Haliem A, Bozkurt TO, Belhaj K, Terauchi R, Vossen JH, Kamoun S (2017) NLR network mediates immunity to diverse plant pathogens. *Proc Natl Acad Sci USA* 114: 8113 – 8118

Wu CH, Derevnina L, Kamoun S (2018) Receptor networks underpin plant immunity. *Science* 360: 1300 – 1301

Wu Z, Li M, Dong OX, Xia S, Liang W, Bao Y, Wasteneys G, Li X (2019) Differential regulation of TNL-mediated immune signaling by redundant helper CNLs. *New Phytol* 222: 938 – 953

Wu CH, Adachi H, De la Concepcion JC, Castells-Graells R, Nekrasov V, Kamoun S (2020) NRC4 gene cluster is not essential for bacterial Flagellin-triggered immunity. *Plant Physiol* 182: 455 – 459

Zhang L, Chen S, Ruan J, Wu J, Tong AB, Yin Q, Li Y, David L, Lu A, Wang WL *et al* (2015) Cryo-EM structure of the activated NAIP2-NLRC4 inflammasome reveals nucleated polymerization. *Science* 350: 404 – 409

© 2022 The Authors

Zhao Y, Yang J, Shi J, Gong YN, Lu Q, Xu H, Liu L, Shao F (2011) The NLRC4 inflammasome receptors for bacterial flagellin and type III secretion apparatus. *Nature* 477: 596 – 600

Zhao YB, Liu MX, Chen TT, Ma X, Li ZK, Zheng Z, Zheng SR, Chen L, Li YZ, Tang LR *et al* (2022) Pathogen effector AvrSr35 triggers Sr35 resistosome assembly via a direct recognition mechanism. *Sci Adv* 8: eabq5108

License: This is an open access article under the terms of the Creative Commons Attribution License, which permits use, distribution and reproduction in any medium, provided the original work is properly cited.

Expanded View Figures

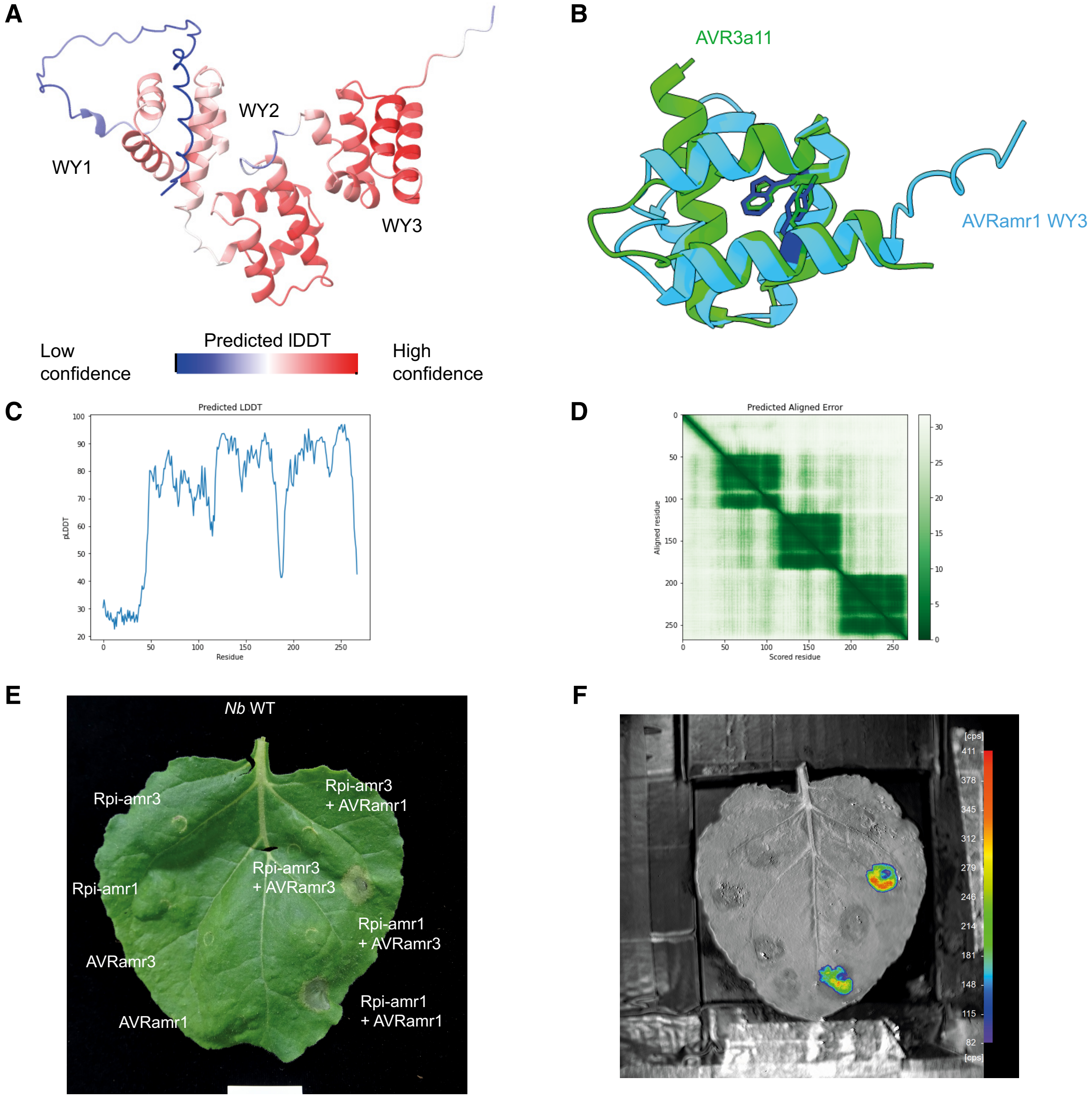

Figure EV1. AVRamr1 is an RXLR effector with WY domains interacting with Rpi-amr1.

A Predicted protein structure of AVRamr1 indicate 3 WY domains. Protein structure model was predicted using AlphaFold and visualized using ChimeraX software. Confidence level of b factors are indicated in colors.

B Third WY domain (WY3, blue) of AVRamr1 shows conserved structure of WY domains with four α-helices as well as the Trp (220W) and Tyr (252Y) residues. The WY3 domain and the WY residues overlap with previously identified WY domain structure of AVR3a11 (PDB ID: 3ZR8) (green).

C Predicted lDDT plot for AVRamr1 structure prediction.

D Predicted aligned error plot for AVRamr1 structure prediction.

E Rpi-amr1 recognizes AVRamr1 and induces HR. Wild-type *N. benthamiana* plants were transiently infiltrated, and leaf samples were imaged at 5 dpi for HR.

F Rpi-amr1 interacts with AVRamr1 *in planta*. Constructs with truncations of luciferase (Nluc or Cluc) were transiently expressed in *nrc2/3/4 KO* N. benthamiana plants and imaged at 3 dpi.

Data information: Experiments were performed with at least three biological replicates with similar results.
Source data are available online for this figure.

 © 2022 The Authors

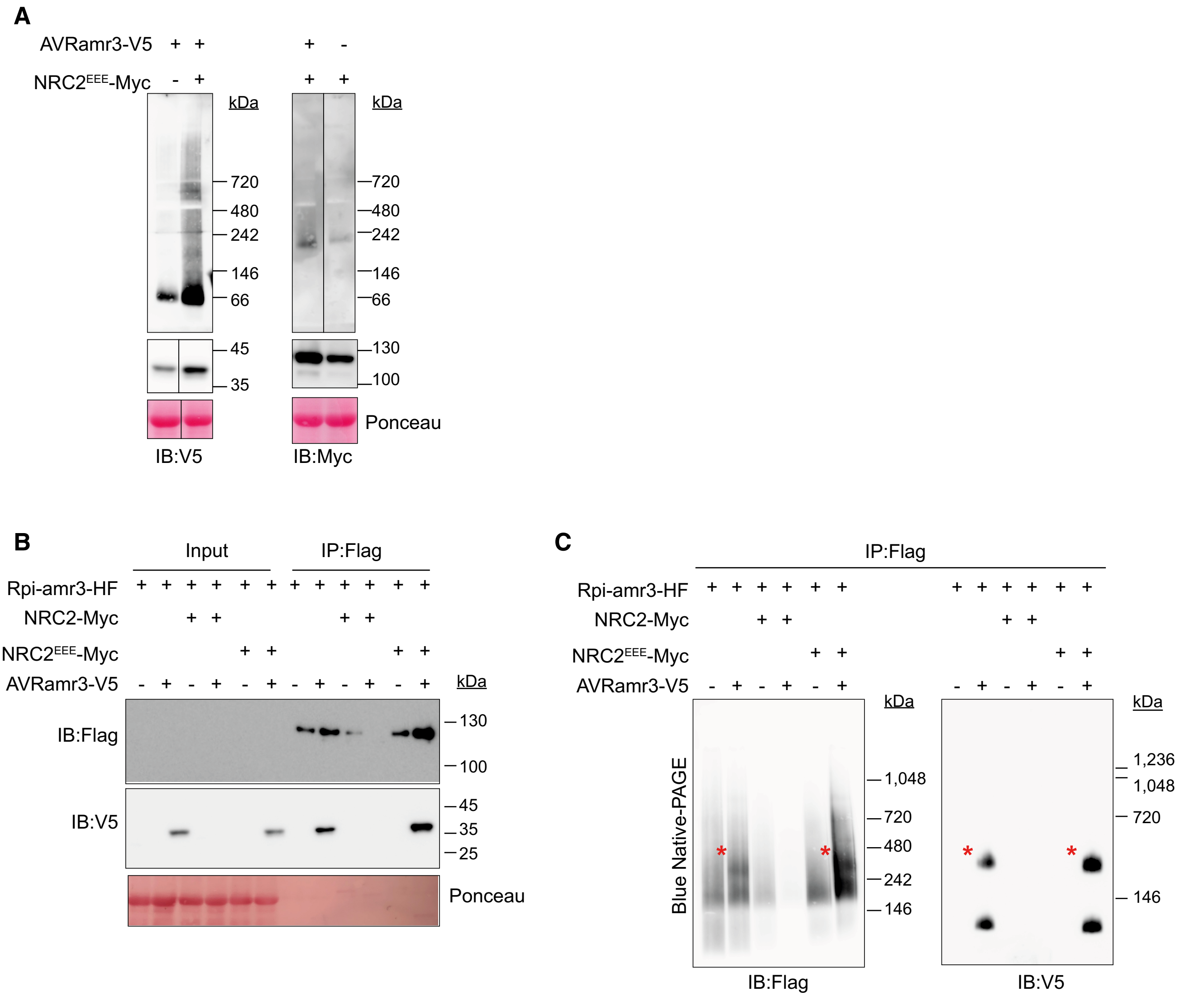

Figure EV2. NRC2 activation leads to degradation of Rpi-amr3 and AVRamr3.

A Protein extracts from *nrc2/3/4* knockout *N. benthamiana* plants expressing AVRamr3-V5 and/or NRC2^{EEE}-Myc were loaded on blue native-PAGE. Aliquot of the protein extracts that were SDS-boiled serve as control.

B NRC2-Myc co-expression leads to degradation of Rpi-amr3 and AVRamr3. Immunoprecipitation with anti-Flag antibody of protein extracts in *nrc2/3/4* knockout *N. benthamiana* plants. Aliquot of samples were SDS-boiled and loaded on SDS–PAGE.

C Immunoprecipitated and eluted samples from (B) were loaded on blue native-PAGE. Rpi-amr3 and AVRamr3 complex is indicated (*, red).

Data information: Ponceau S staining serves as loading control for panels (A and B). Molecular markers are shown on the right. Similar results were observed with at least three biological replicates.
Source data are available online for this figure.

© 2022 The Authors

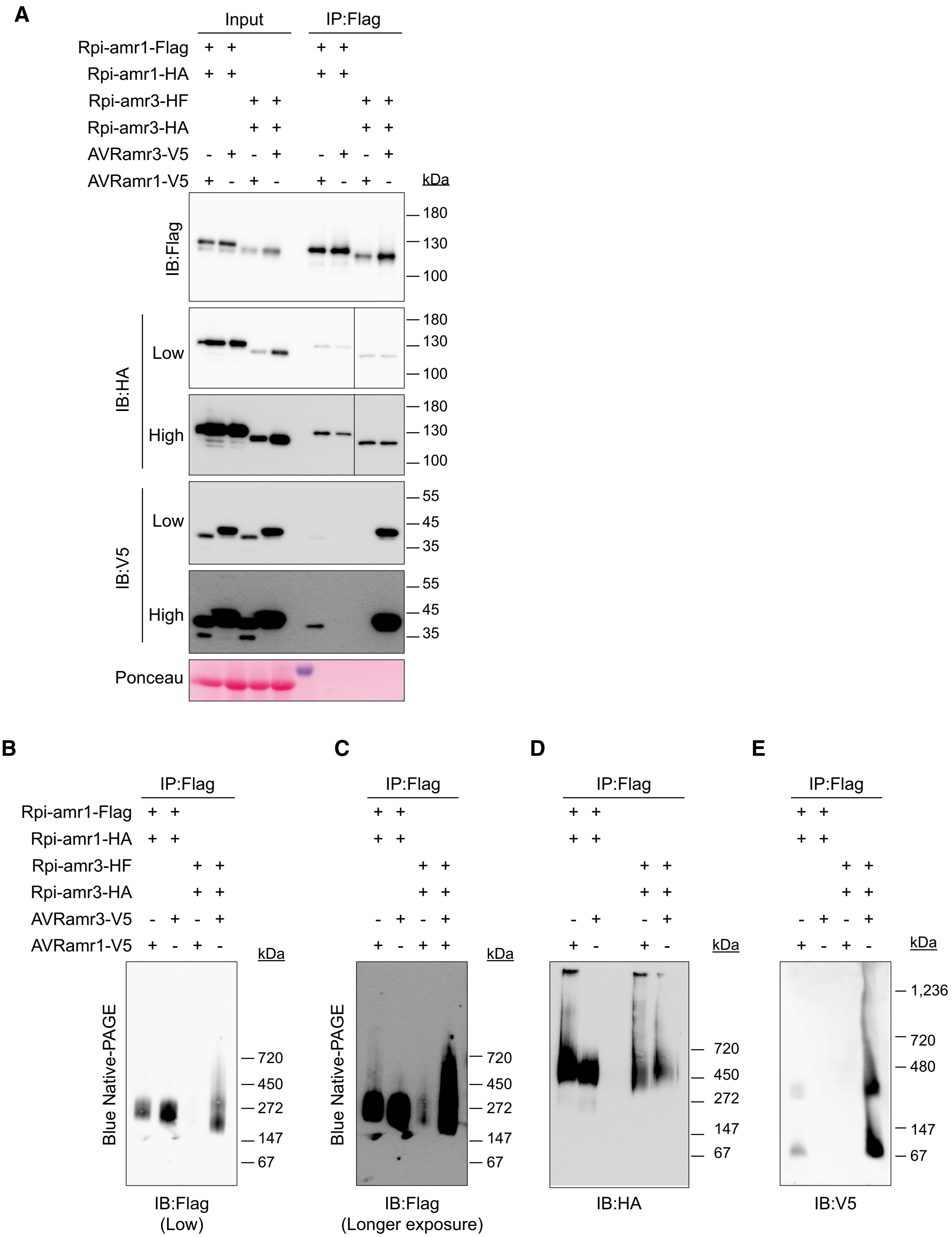

Figure EV3.

 © 2022 The Authors

Figure EV3. Small proportion of Rpi-amr1 or Rpi-amr3 can self-associate.

A Rpi-amr1 and Rpi-amr3 can self-associate weakly *in vivo* regardless of effector co-expression. Protein samples were immunoprecipitated with anti-FLAG antibodies and were blotted for HA-tagged Rpi-amr (anti-HA), and AVRamr (anti-V5). Both low and high exposures of the blots are shown. Solid line in membrane blotted with anti-HA indicate gaps between the samples.

B, C Rpi-amr1 and Rpi-amr3 are mostly monomers *in vivo*. Immunoprecipitated samples of (A) were loaded on blue native-PAGE. High exposure versions of (B) are shown in (C).

D Rpi-amr1 and Rpi-amr3 can self-associate weakly *in vivo* regardless of effector co-expression and form slower-migrating protein complexes. Protein samples were immunoprecipitated with anti-FLAG antibodies, loaded onto blue native-PAGE, and were blotted for HA-tagged Rpi-amr (anti-HA).

E AVRamr1 and AVRamr3 form a protein complex with Rpi-amr1 and Rpi-amr3, respectively. Protein extracts from *N. benthamiana nrc2/3/4 knockout* plants were immunoprecipitated with anti-Flag antibodies and loaded on blue native-PAGE.

Data information: Ponceau S staining serve as loading control in panel (A). Molecular weight markers are shown on the right. Experiments were done with at least three biological replicates with similar results.
Source data are available online for this figure.

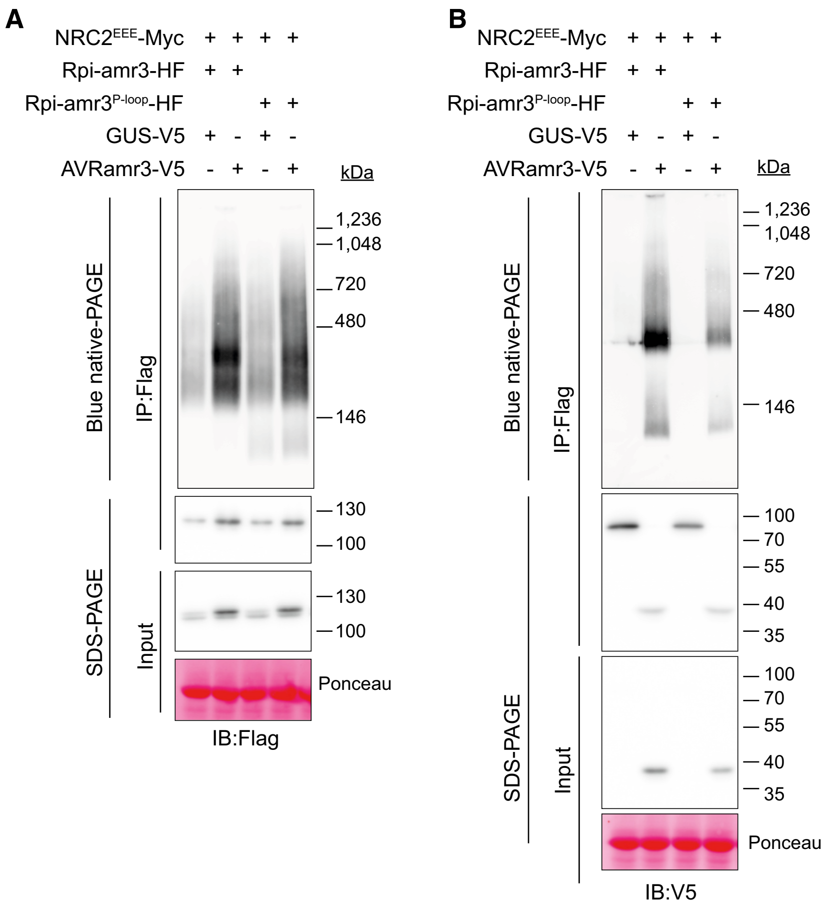

Figure EV4. Rpi-amr3 P-loop mutant interacts with and forms stable complex with AVRamr3.

A P-loop of Rpi-amr3 is dispensable for association with AVRamr3. Protein extracts from *N. benthamiana nrc2/3/4 knockout* plants were immunoprecipitated with anti-Flag antibody and blue native-PAGE was performed. Membranes were immunoblotted with anti-Flag.

B AVRamr3 associates with both wild-type Rpi-amr3 and Rpi-amr3$^{P\text{-}loop}$. Protein extracts from *N. benthamiana nrc2/3/4 knockout* plants were immunoprecipitated with anti-Flag antibody and blue native-PAGE was performed. Membranes were immunoblotted with anti-V5.

Data information: Ponceau S staining serves as loading control for panels (A and B). Molecular markers are shown on the right. Similar results were observed in at least three biological replicates.
Source data are available online for this figure.

© 2022 The Authors

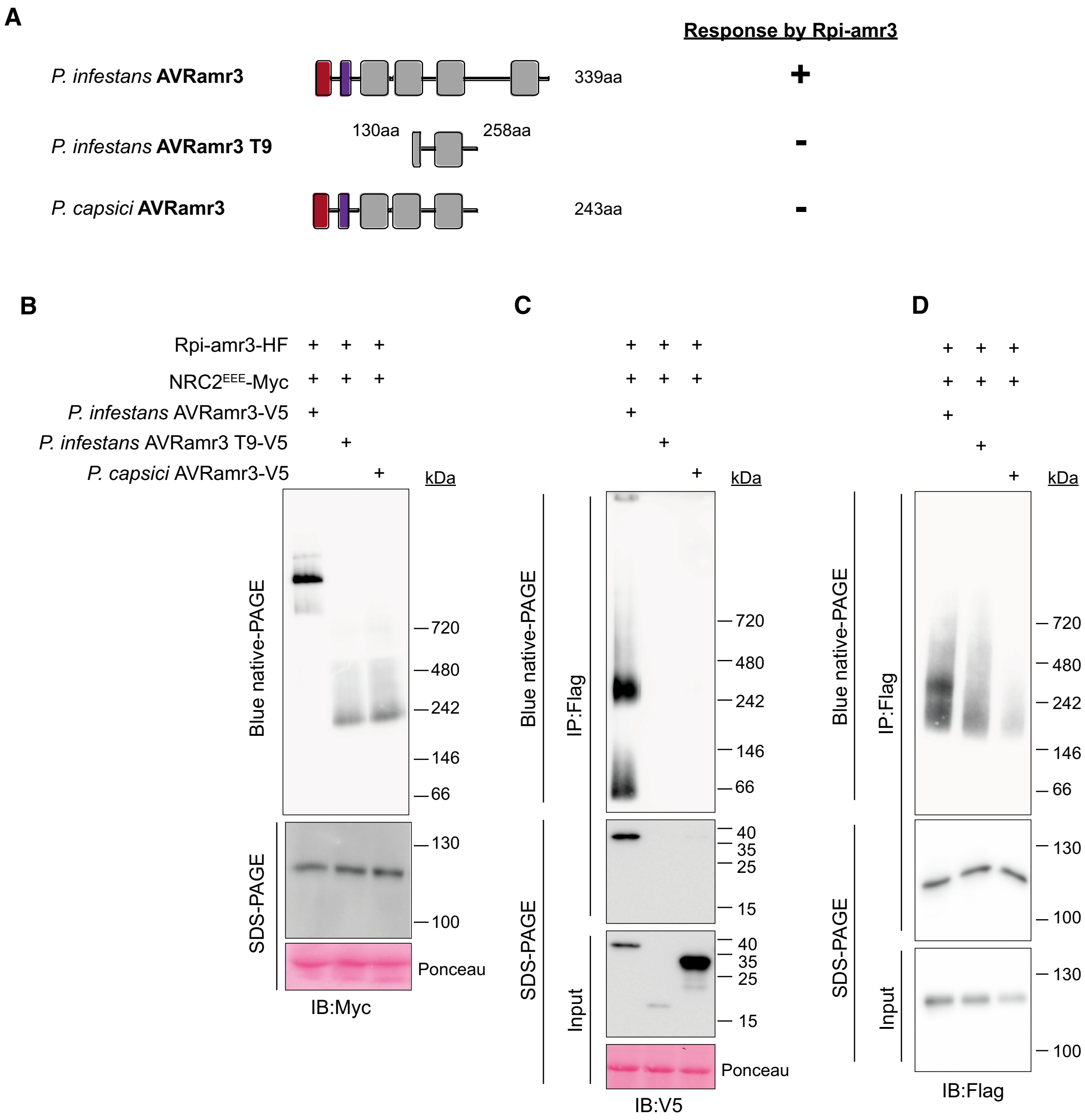

Figure EV5. Non-recognized alleles of AVRamr3 do not trigger NRC2 oligomerization and do not interact with Rpi-amr3.

A Schematic depiction of AVRamr3 from *Phytophthora infestans*, an unrecognized, truncated version of AVRamr3 (AVRamr3 T9), and AVRamr3 from *P. capsici*. Response of the corresponding alleles by Rpi-amr3, and thus occurrence of HR is indicated as + (recognition) or − (no recognition).

B $NRC2^{EEE}$-Myc oligomerizes in recognition-dependent manner. Protein extracts from *N. benthamiana nrc2/3/4 knockout* were loaded on blue native-PAGE, and blotted for Myc.

C Recognition is correlated with interaction and protein complex formation of AVRamr3 with Rpi-amr3. Protein extracts from (B) were immunoprecipitated with anti-Flag antibody and were loaded on blue native-PAGE and blotted for V5.

D Recognition is correlated with interaction and protein complex formation of Rpi-amr3 with AVRamr3. Protein extracts from (B) were immunoprecipitated with anti-Flag antibody and were loaded on blue native-PAGE and blotted for Flag.

Data information: SDS-boiled input and IP eluates were loaded onto SDS–PAGE as control. Ponceau S staining serves as loading control in panels (B and C). Molecular markers are indicated on the right. Experiments were done with at least three biological replicates with similar results. Ponceau S loading for (D) was same as with (C). Source data are available online for this figure.

 © 2022 The Authors
