# Supplementary material for: Effector‐dependent activation and oligomerization of plant NRC class helper NLRs by sensor NLR immune receptors Rpi‐amr3 and Rpi‐amr1
Source: EMBO J. 2023 Jan 2;42(5):e111484. doi: 10.15252/embj.2022111484 (PMC9975942; doi:10.15252/embj.2022111484)

Source Data for Figure 2D

NRC2 oligomerization by Rpi-amr1 activation

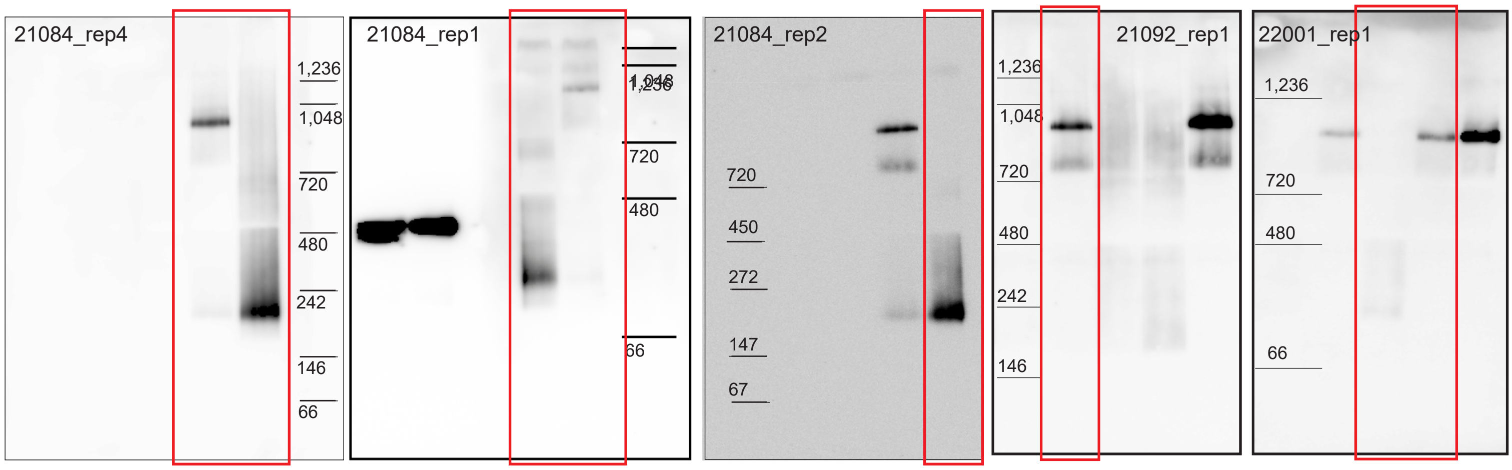

NRC2 oligomerization by Rpi-amr3 activation

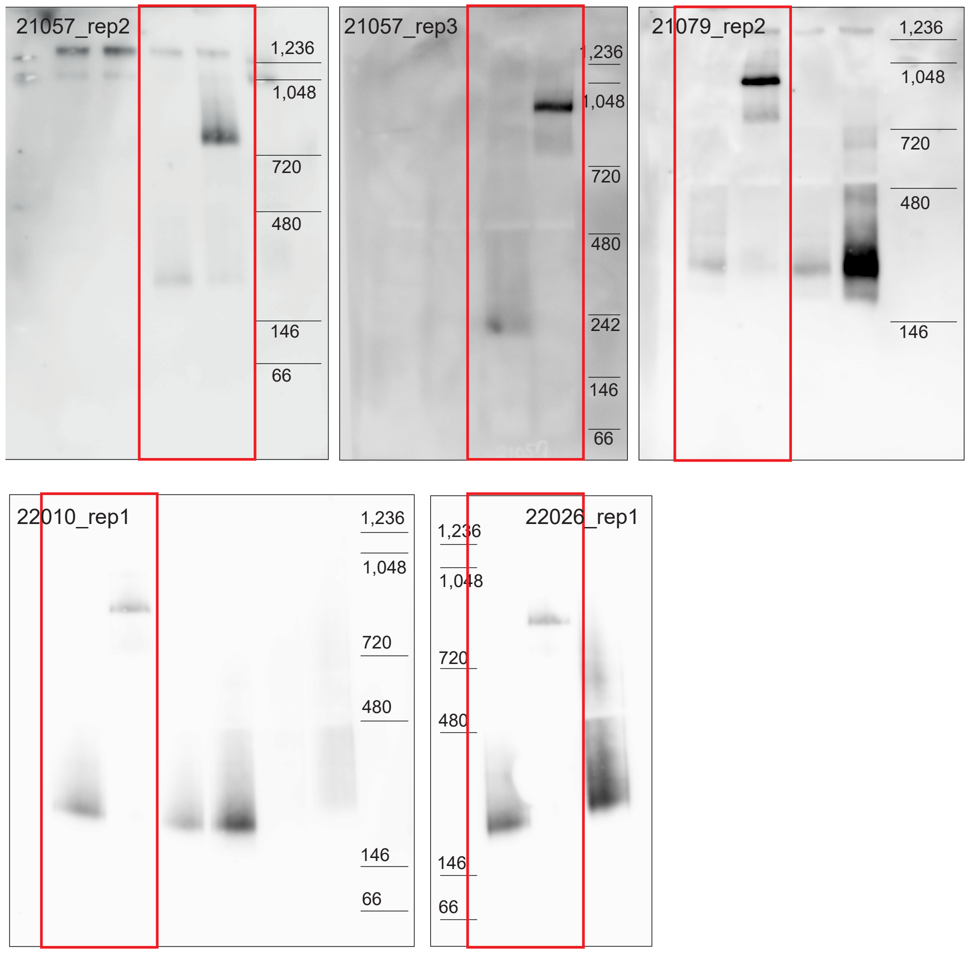

Supplement: Supplementary file 7 — Source Data for Figure 2 [file EMBJ-42-e111484-s011.zip › Figure 2/2D/BlotdataforFigure2D.pdf]
