# Supplementary figures and images for: Effector‐dependent activation and oligomerization of plant NRC class helper NLRs by sensor NLR immune receptors Rpi‐amr3 and Rpi‐amr1 (part 2 of 2)
Source: EMBO J. 2023 Jan 2;42(5):e111484. doi: 10.15252/embj.2022111484 (PMC9975942; doi:10.15252/embj.2022111484)

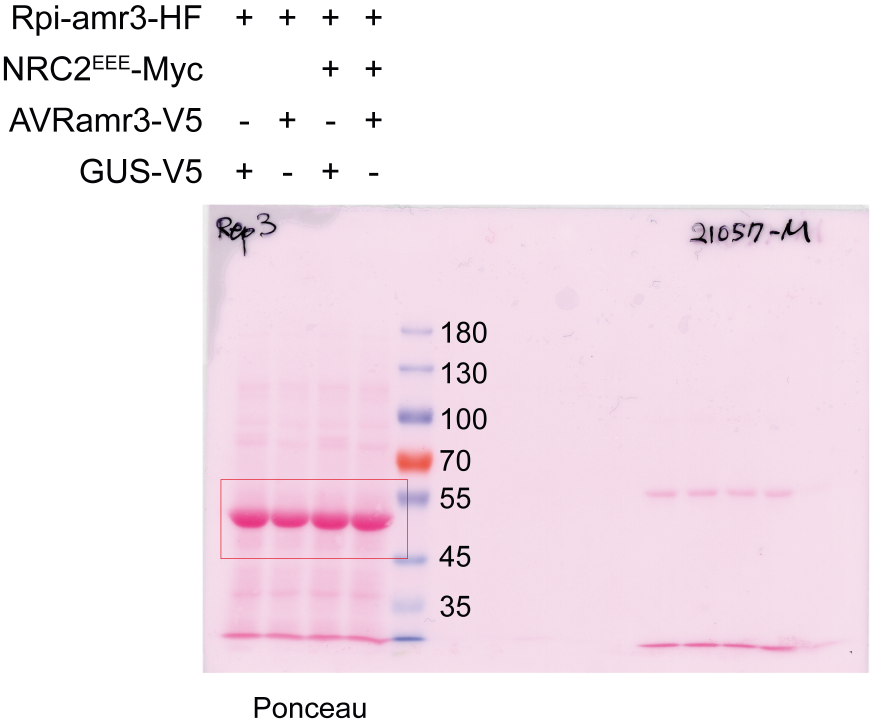

Supplement: Supplementary file 7 — Source Data for Figure 2 [file EMBJ-42-e111484-s011.zip › Figure 2/2A/Western Myc Ponceau_annotations.tif]

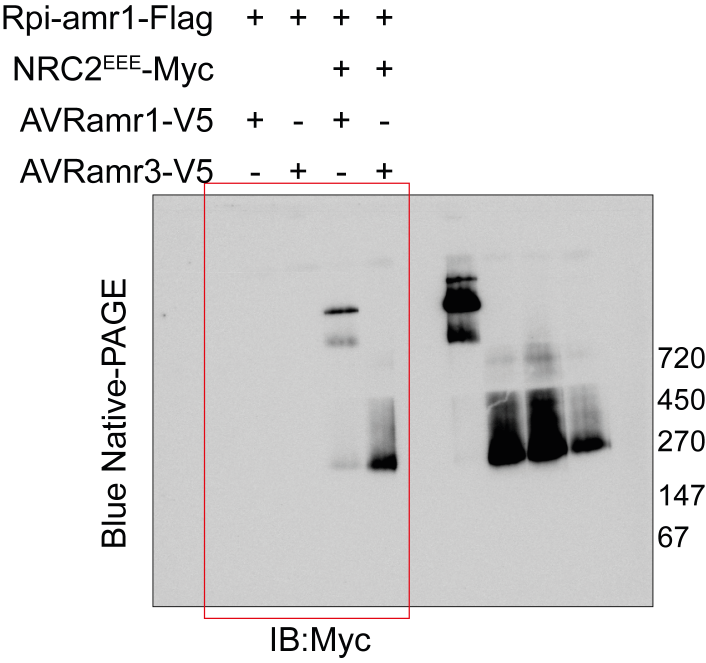

Supplement: Supplementary file 7 — Source Data for Figure 2 [file EMBJ-42-e111484-s011.zip › Figure 2/2B/Western Myc_annotations.tif]

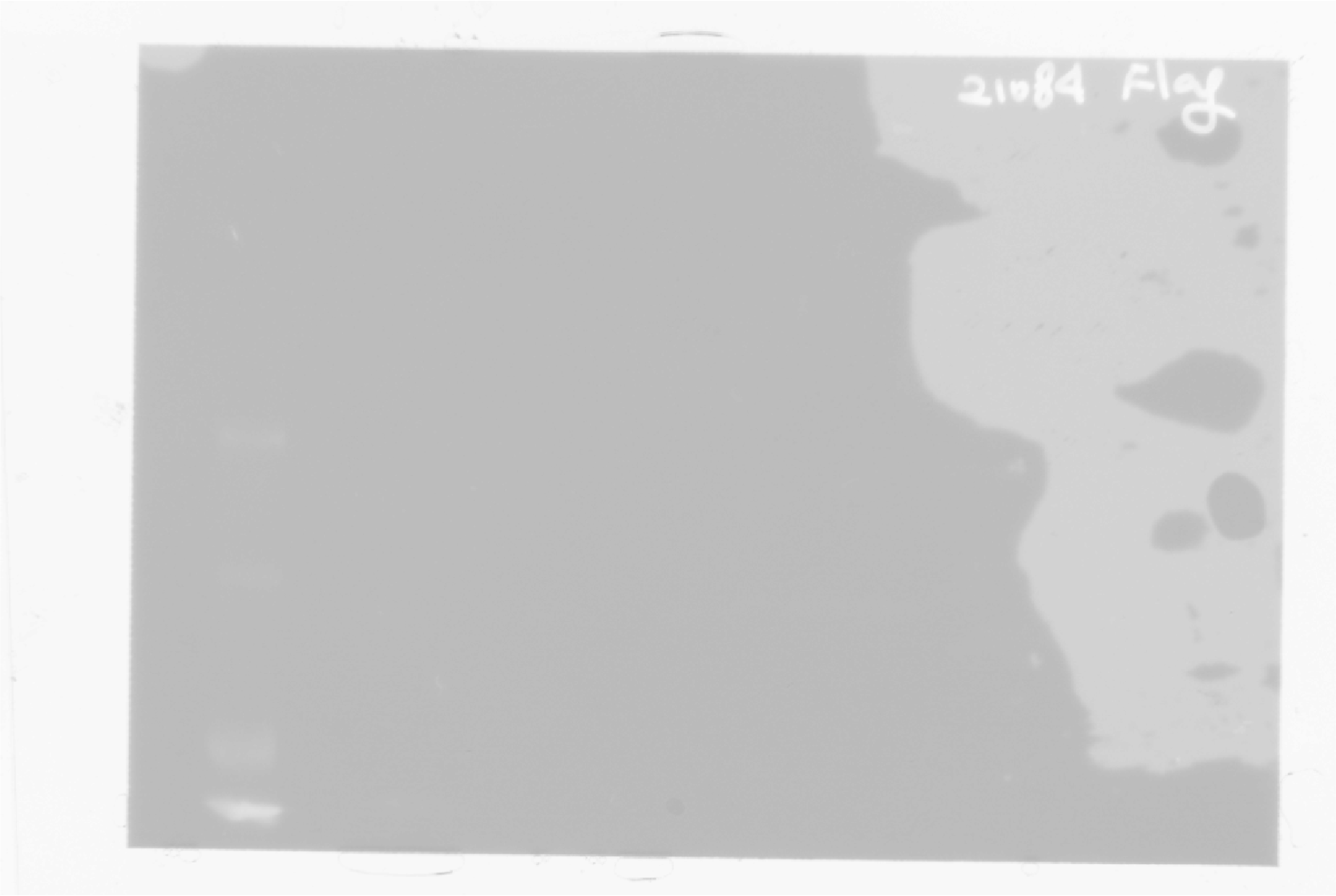

Supplement: Supplementary file 7 — Source Data for Figure 2 [file EMBJ-42-e111484-s011.zip › Figure 2/2C/Western Flag marker.tif]

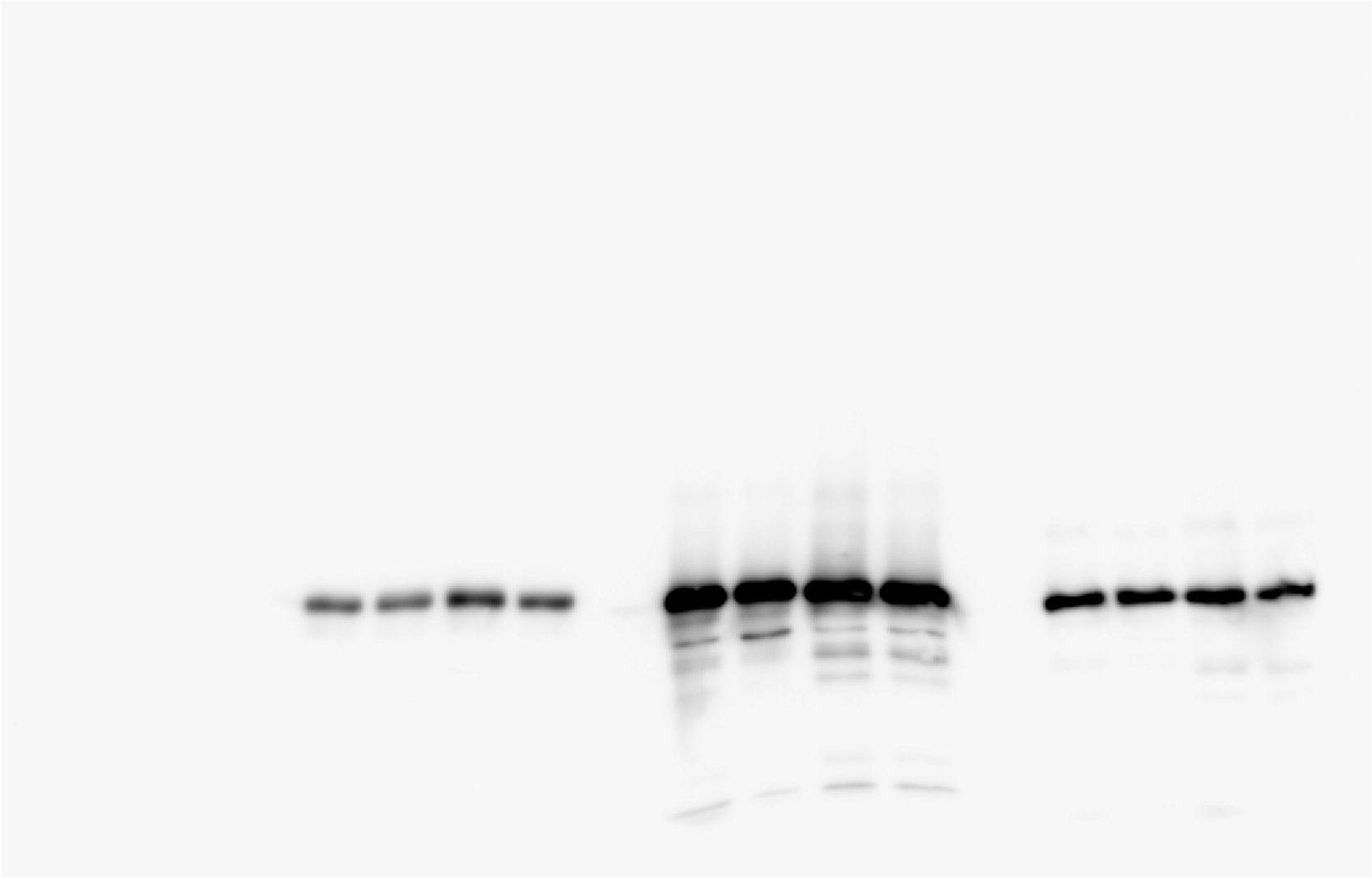

Supplement: Supplementary file 7 — Source Data for Figure 2 [file EMBJ-42-e111484-s011.zip › Figure 2/2C/Western Flag.tif]

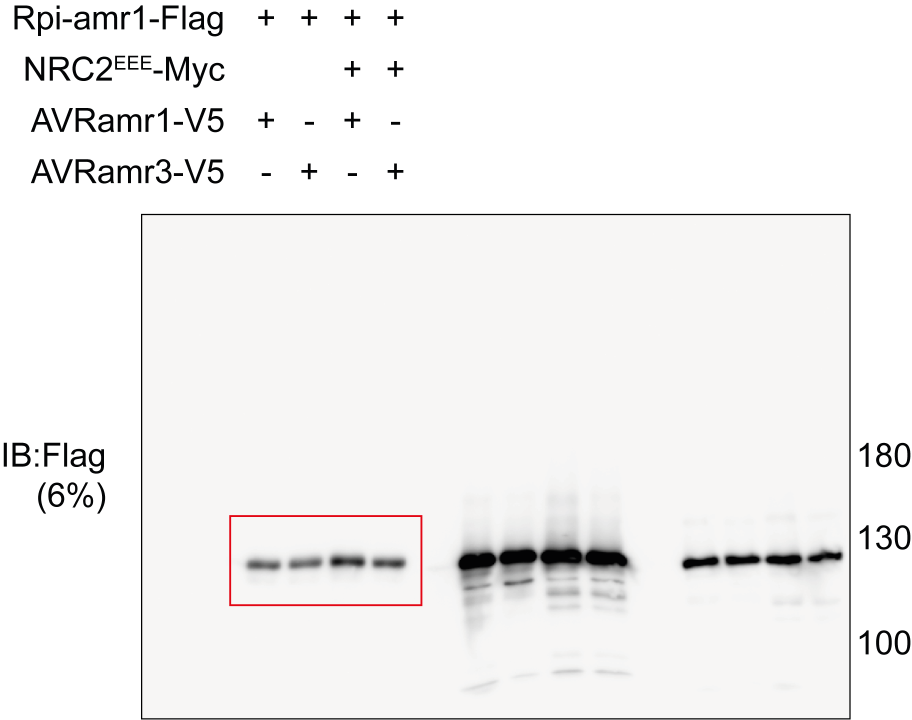

Supplement: Supplementary file 7 — Source Data for Figure 2 [file EMBJ-42-e111484-s011.zip › Figure 2/2C/Western Flag_annotations.tif]

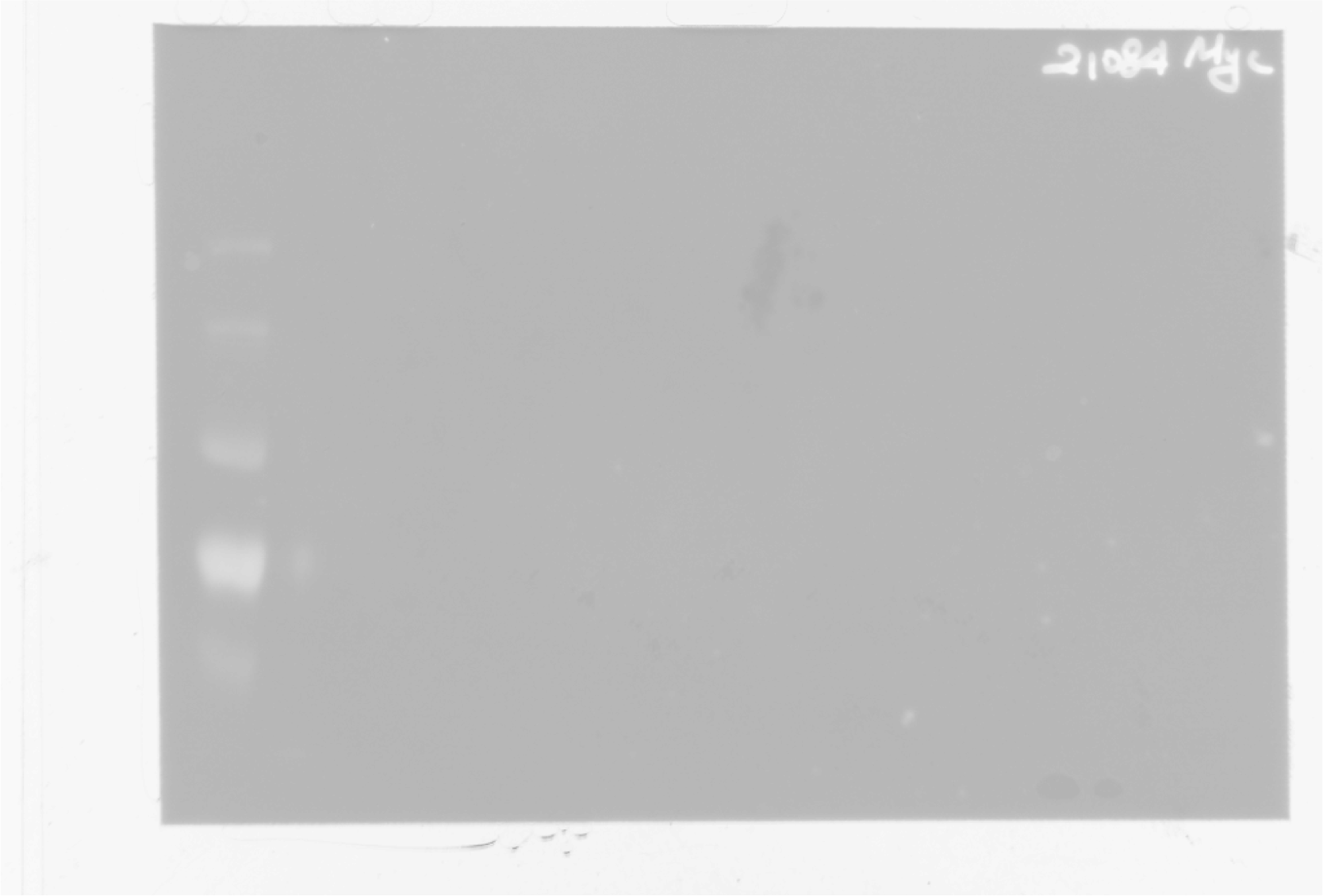

Supplement: Supplementary file 7 — Source Data for Figure 2 [file EMBJ-42-e111484-s011.zip › Figure 2/2C/Western Myc Marker.tif]

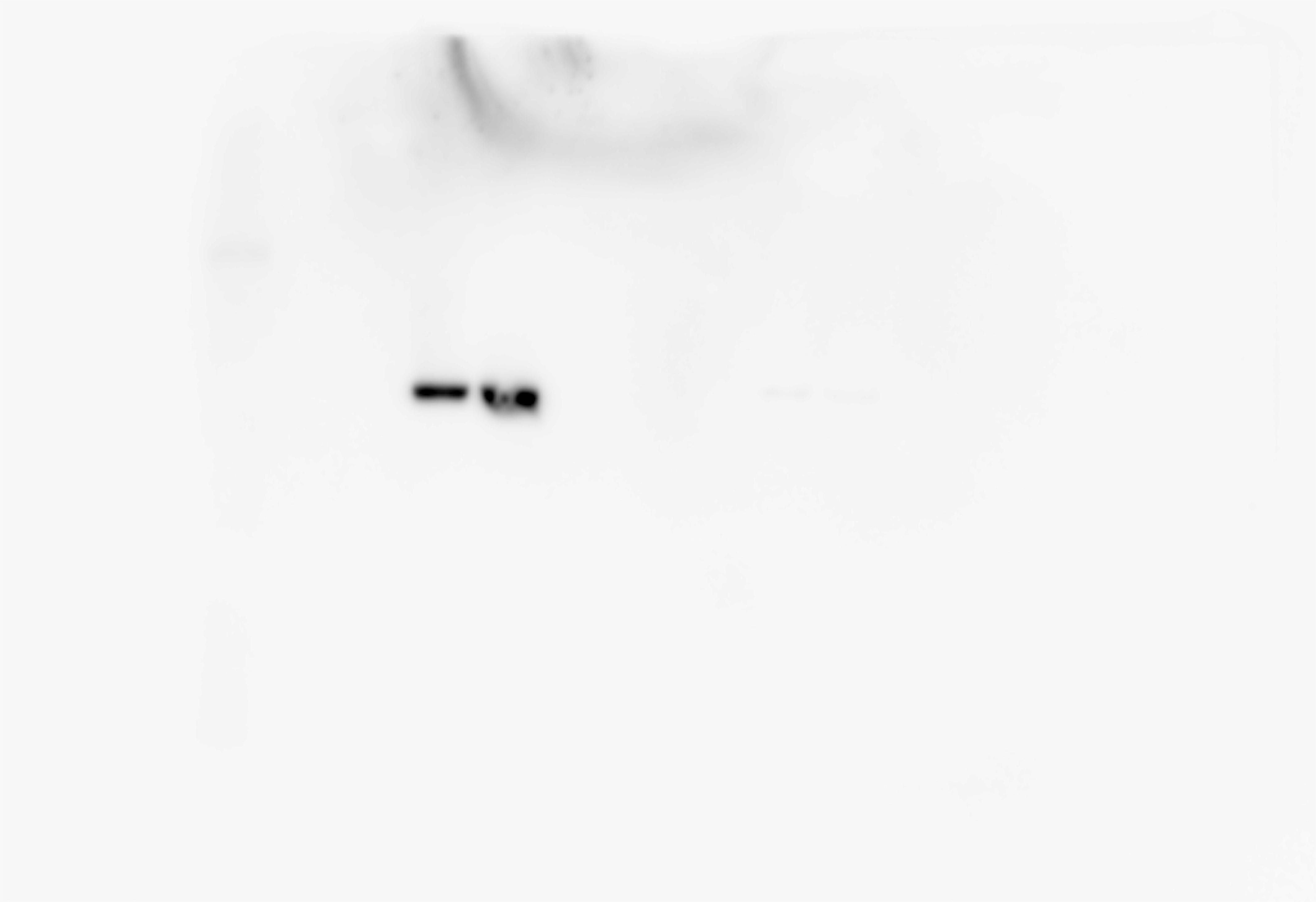

Supplement: Supplementary file 7 — Source Data for Figure 2 [file EMBJ-42-e111484-s011.zip › Figure 2/2C/Western Myc.tif]

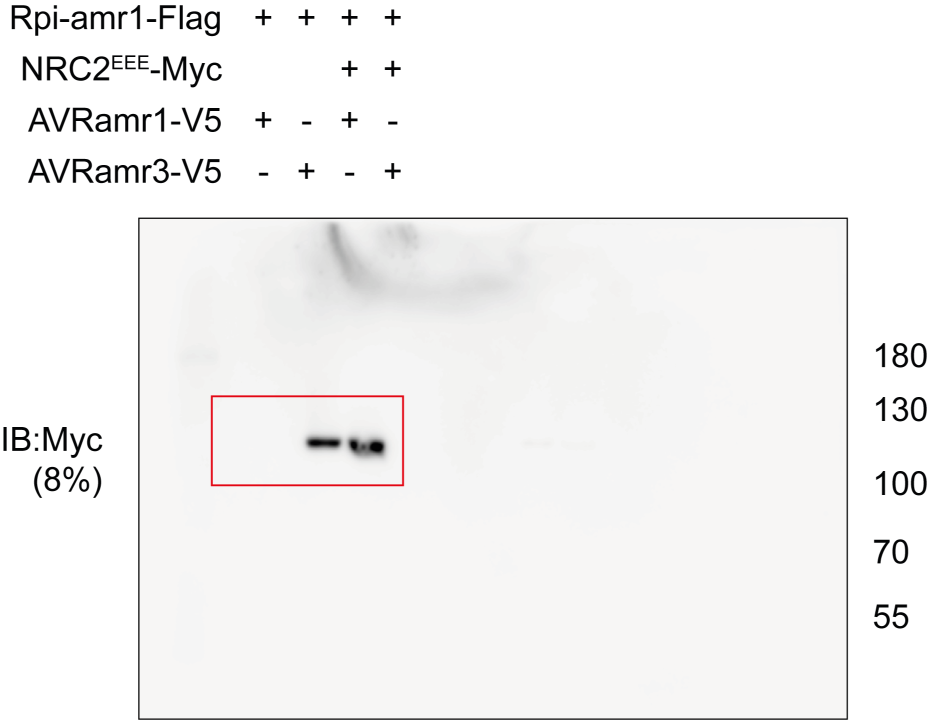

Supplement: Supplementary file 7 — Source Data for Figure 2 [file EMBJ-42-e111484-s011.zip › Figure 2/2C/Western Myc_annotations.tif]

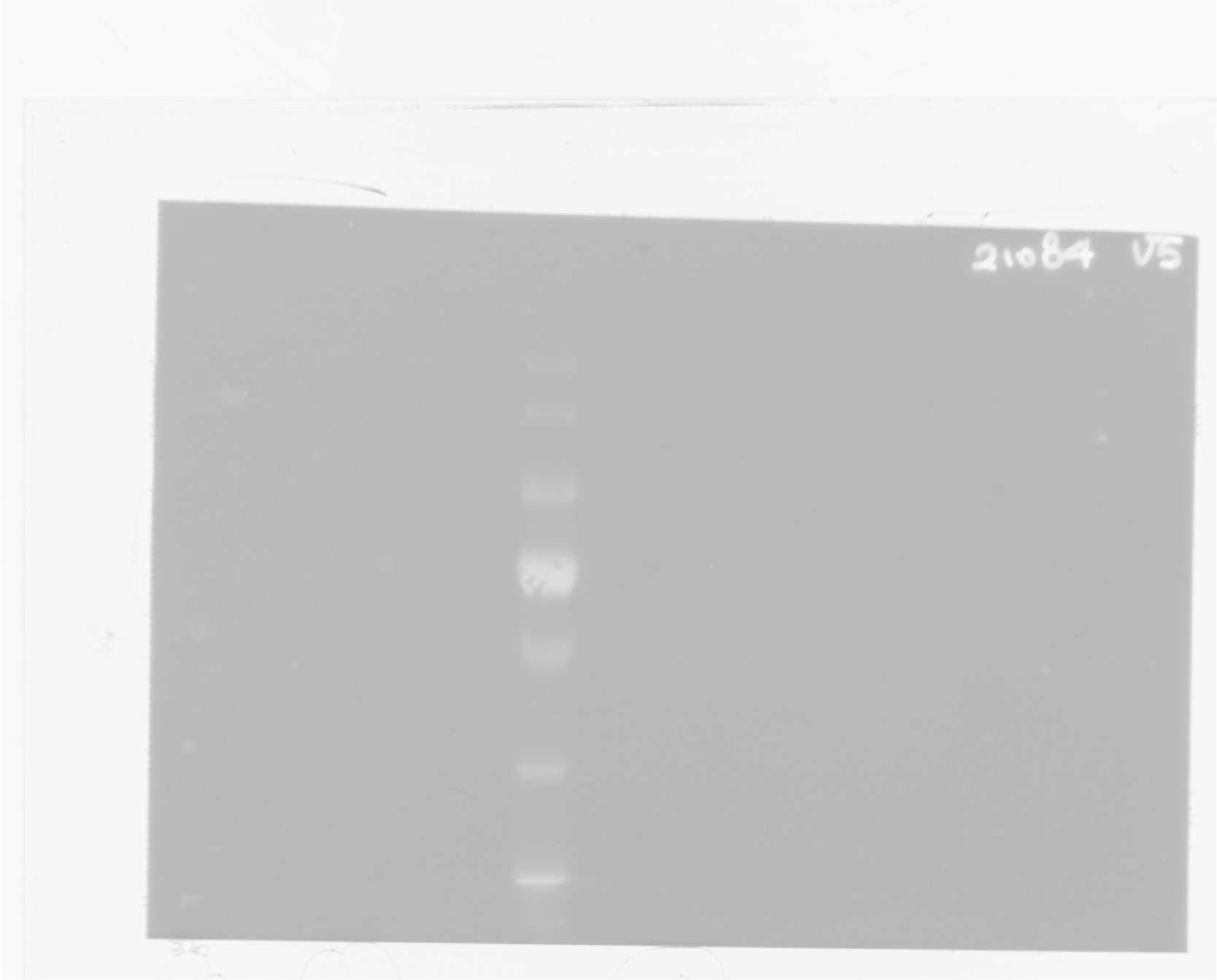

Supplement: Supplementary file 7 — Source Data for Figure 2 [file EMBJ-42-e111484-s011.zip › Figure 2/2C/Western V5 Marker.tif]

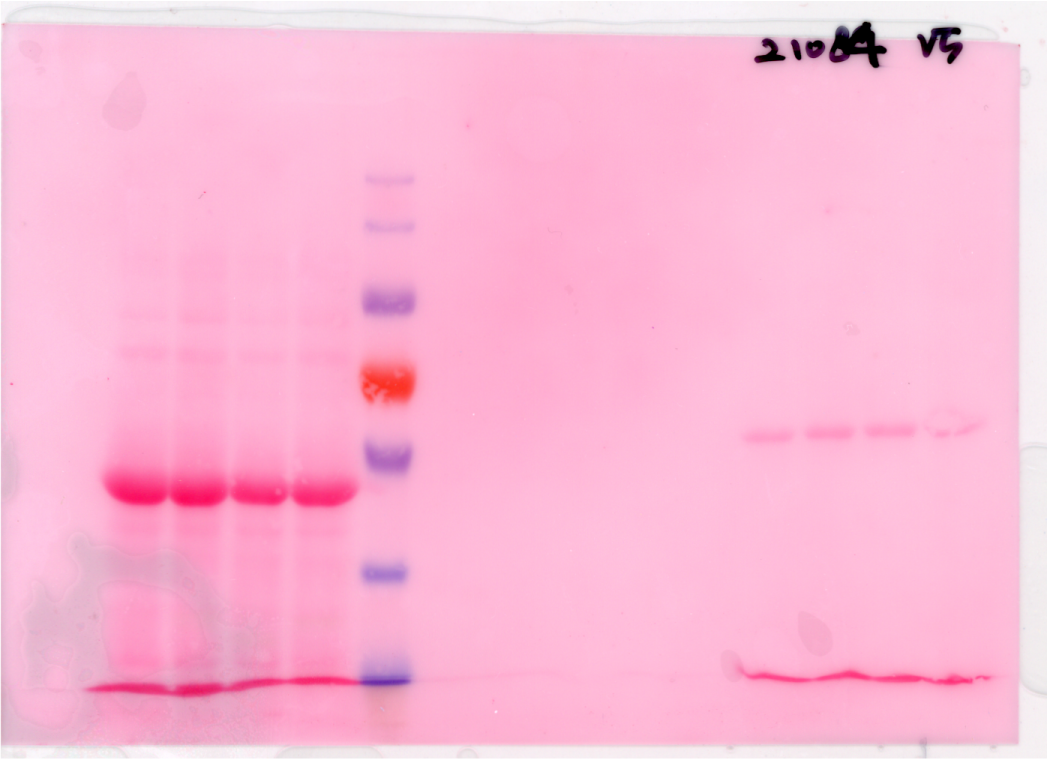

Supplement: Supplementary file 7 — Source Data for Figure 2 [file EMBJ-42-e111484-s011.zip › Figure 2/2C/Western V5 Ponceau.tif]

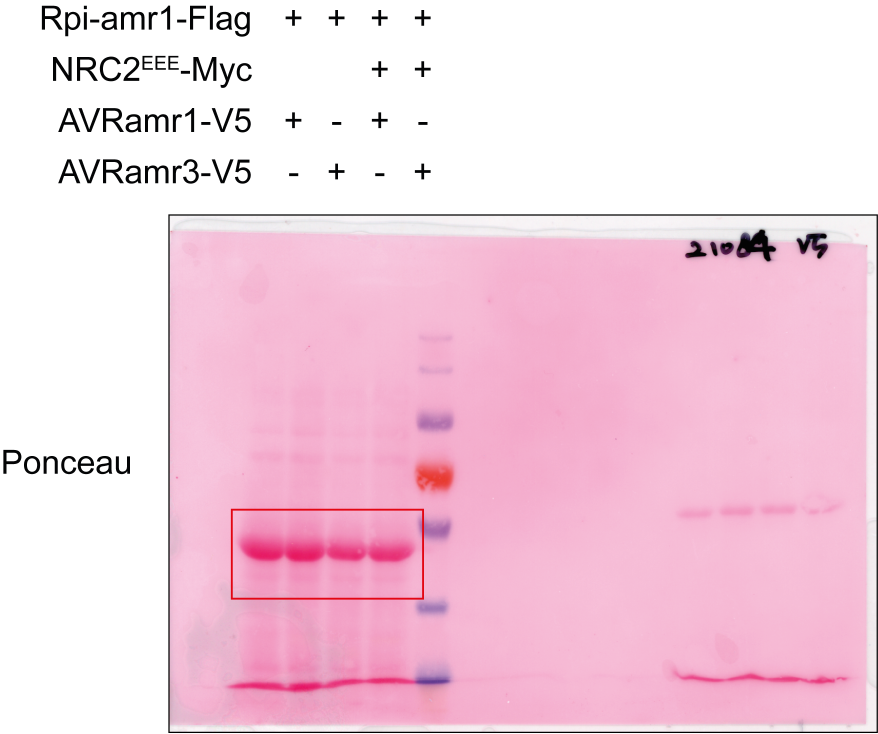

Supplement: Supplementary file 7 — Source Data for Figure 2 [file EMBJ-42-e111484-s011.zip › Figure 2/2C/Western V5 Ponceau_annotations.tif]

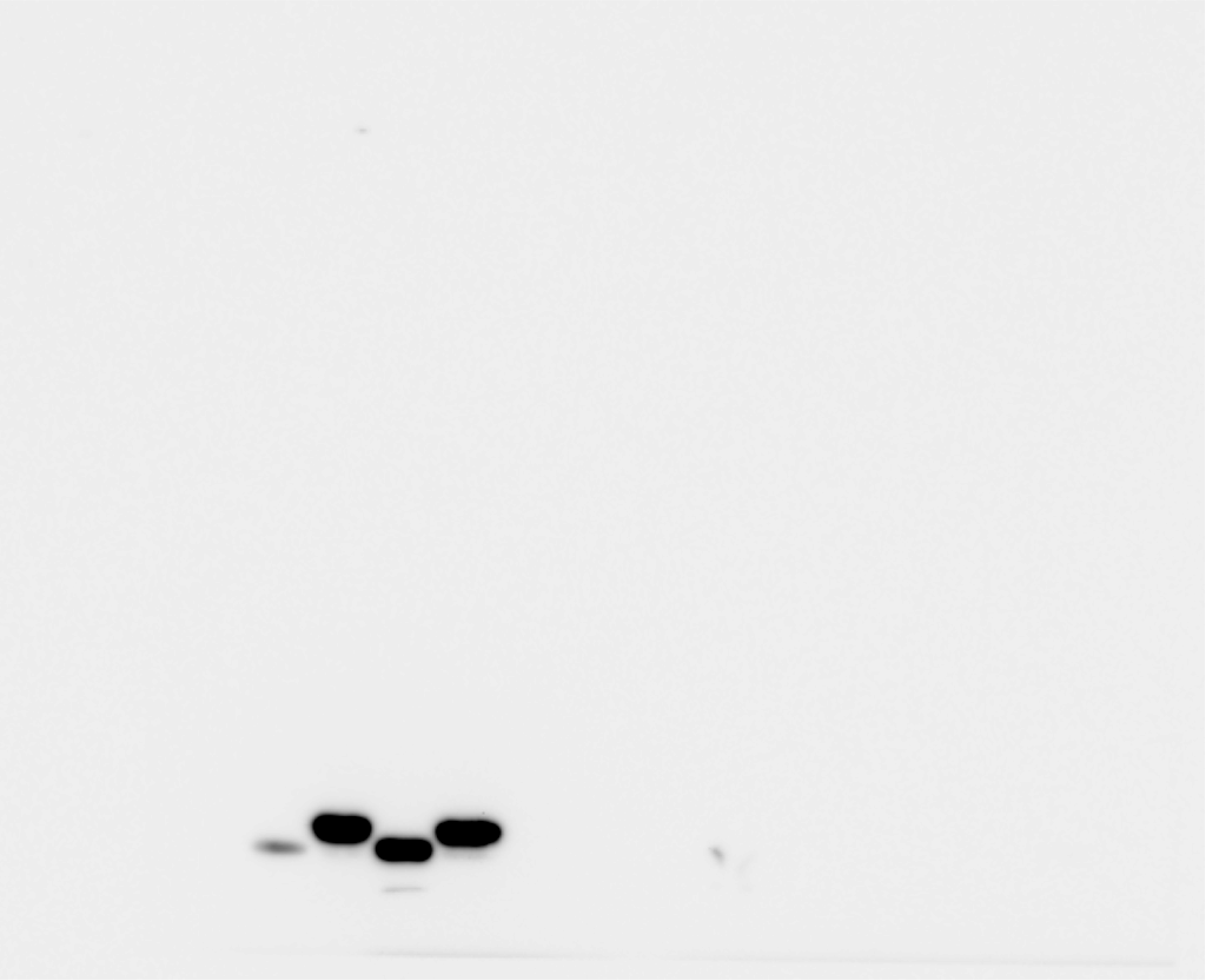

Supplement: Supplementary file 7 — Source Data for Figure 2 [file EMBJ-42-e111484-s011.zip › Figure 2/2C/Western V5.tif]

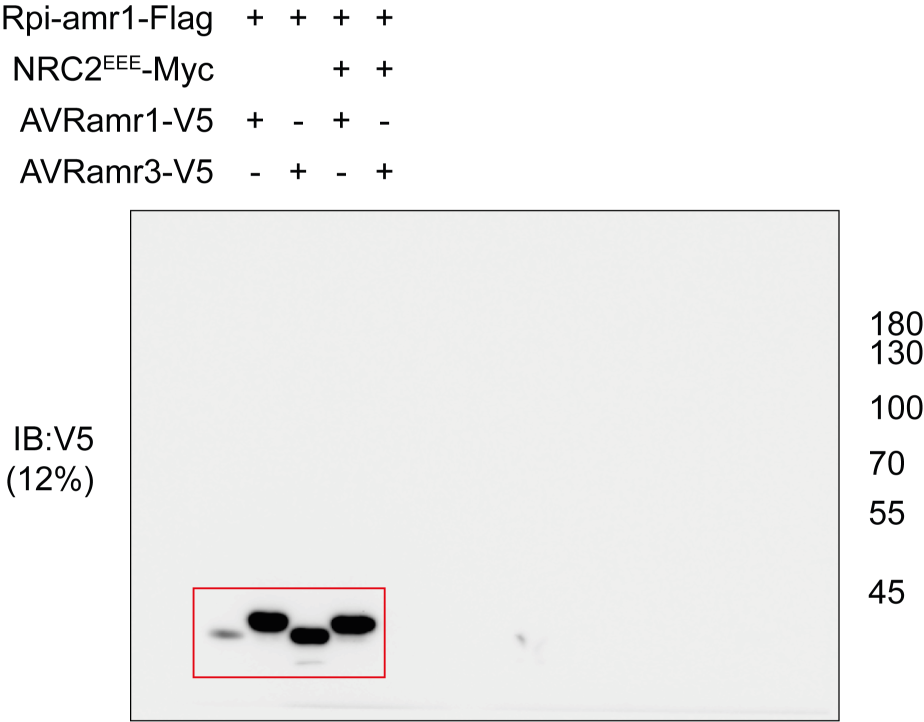

Supplement: Supplementary file 7 — Source Data for Figure 2 [file EMBJ-42-e111484-s011.zip › Figure 2/2C/Western V5_annotations.tif]

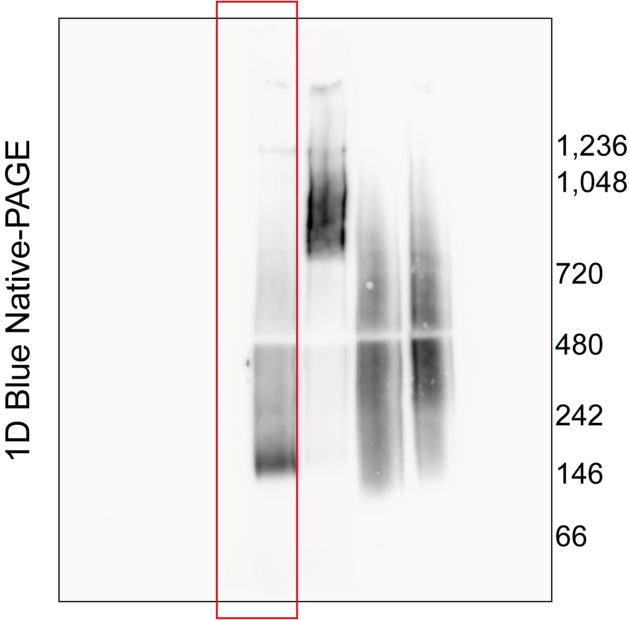

Supplement: Supplementary file 8 — Source Data for Figure 3 [file EMBJ-42-e111484-s006.zip › Figure 3/3B/1D BNP Western Myc_annotations.tif]

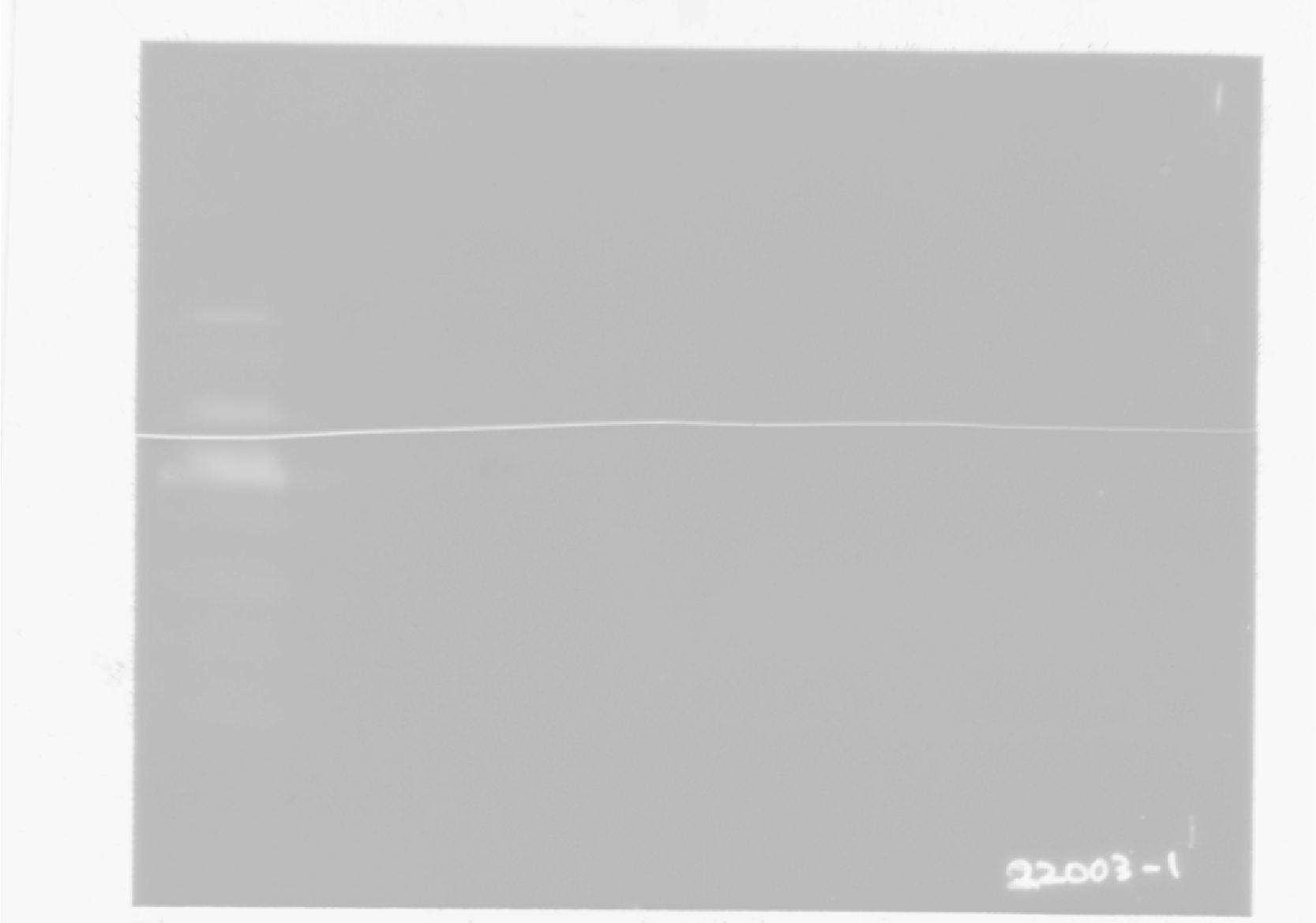

Supplement: Supplementary file 8 — Source Data for Figure 3 [file EMBJ-42-e111484-s006.zip › Figure 3/3B/2D BNP-SDS Western Flag Marker.tif]

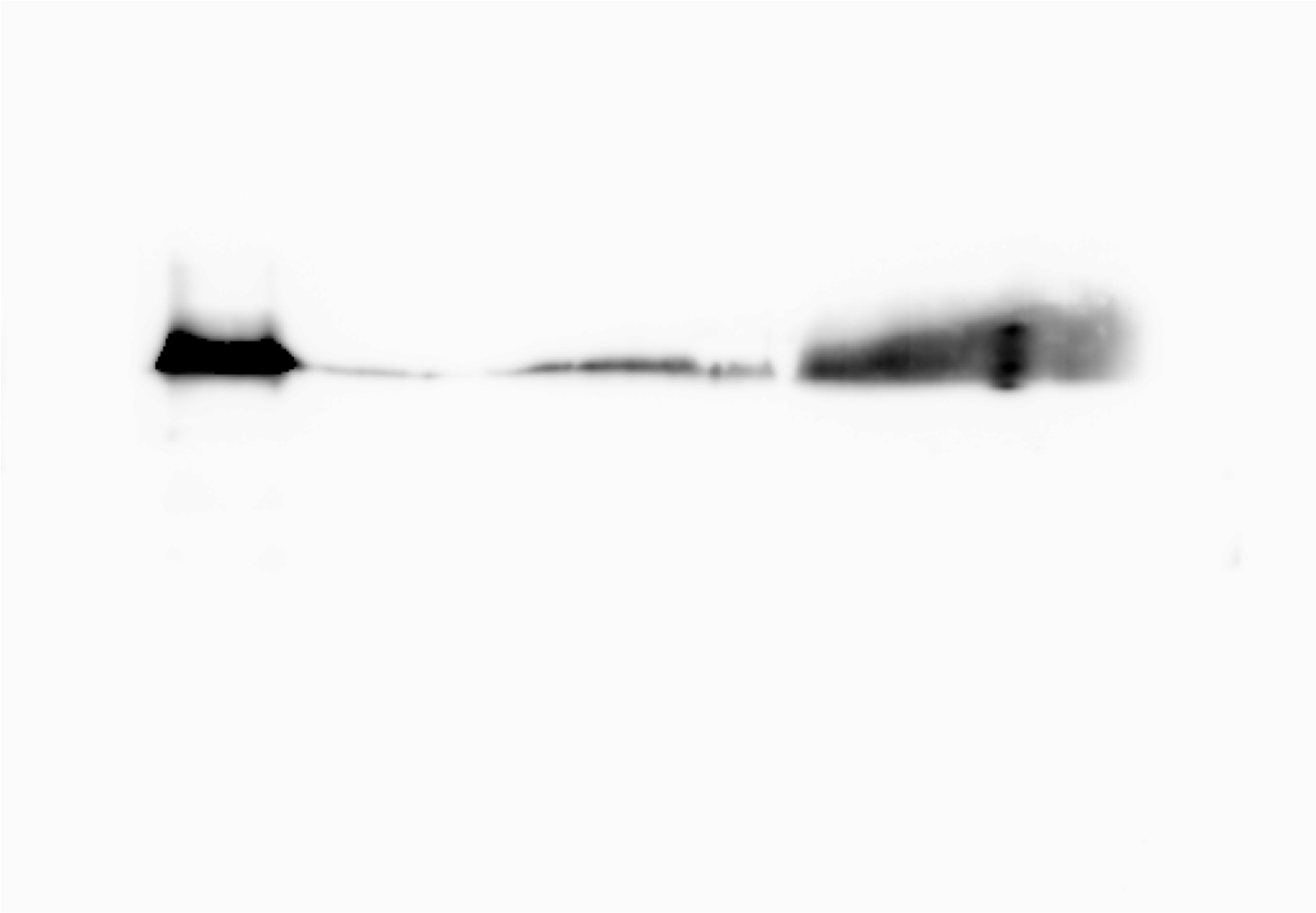

Supplement: Supplementary file 8 — Source Data for Figure 3 [file EMBJ-42-e111484-s006.zip › Figure 3/3B/2D BNP-SDS Western Flag.tif]

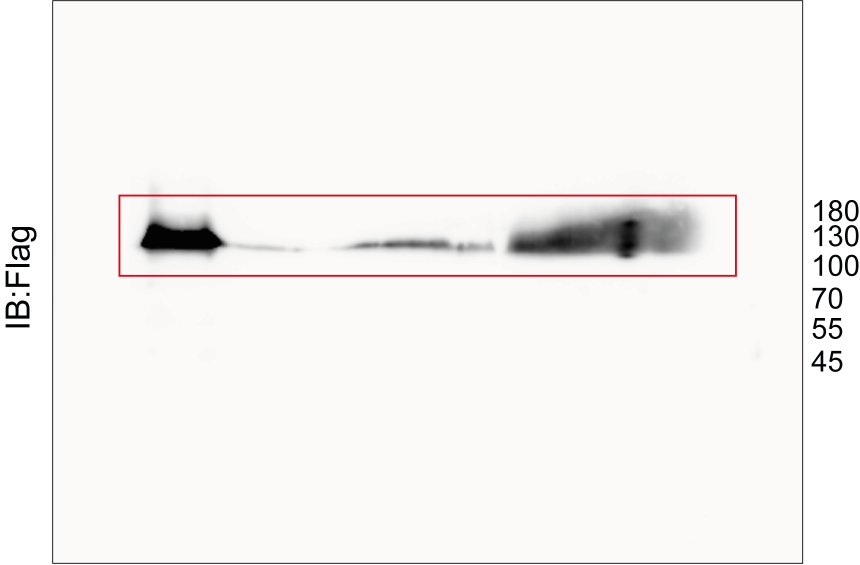

Supplement: Supplementary file 8 — Source Data for Figure 3 [file EMBJ-42-e111484-s006.zip › Figure 3/3B/2D BNP-SDS Western Flag_annotations.tif]

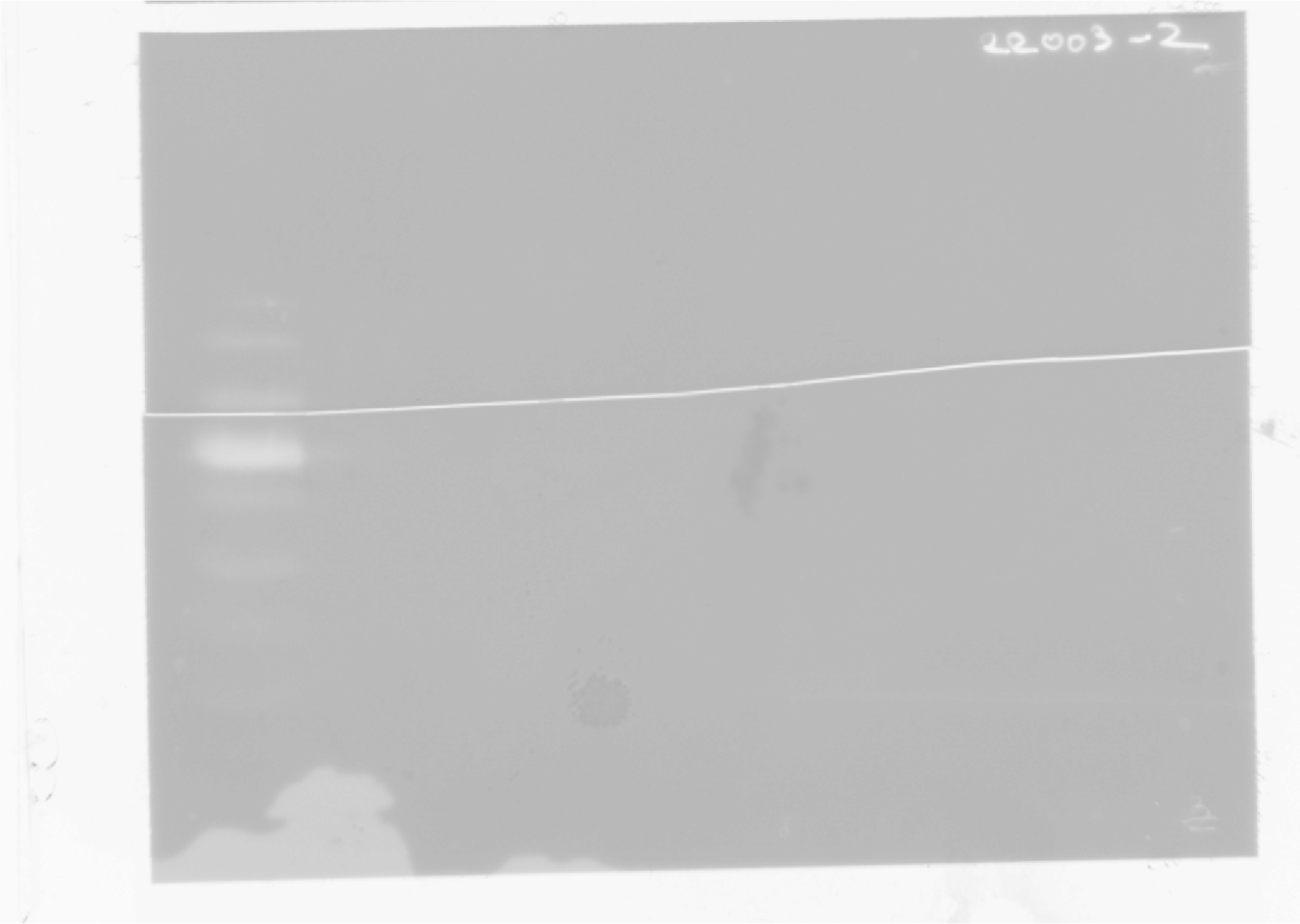

Supplement: Supplementary file 8 — Source Data for Figure 3 [file EMBJ-42-e111484-s006.zip › Figure 3/3B/2D BNP-SDS Western Myc Marker.tif]

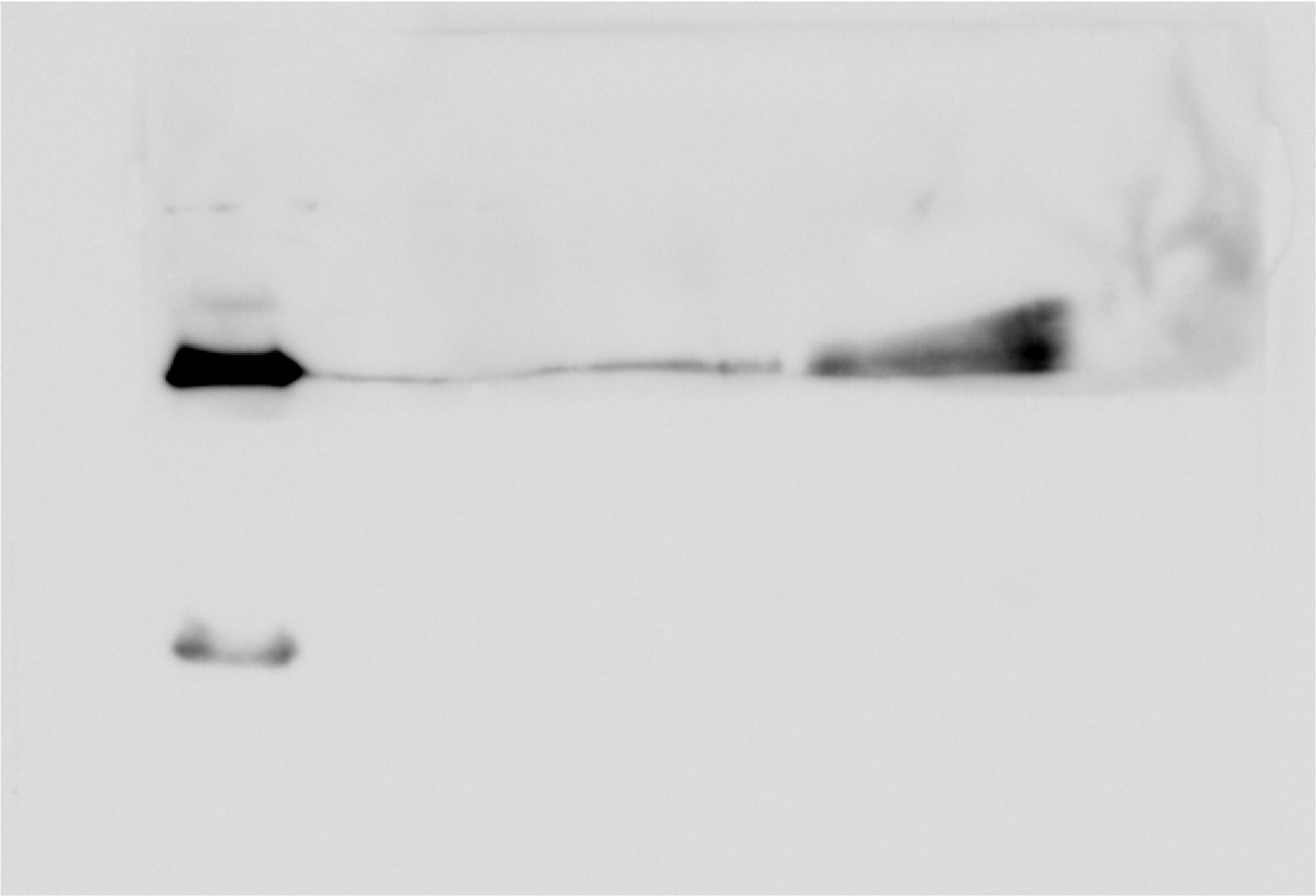

Supplement: Supplementary file 8 — Source Data for Figure 3 [file EMBJ-42-e111484-s006.zip › Figure 3/3B/2D BNP-SDS Western Myc.tif]

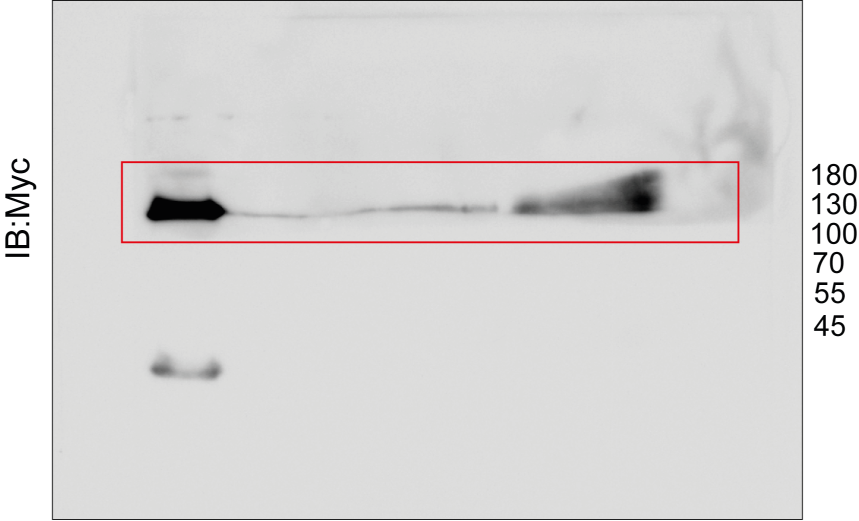

Supplement: Supplementary file 8 — Source Data for Figure 3 [file EMBJ-42-e111484-s006.zip › Figure 3/3B/2D BNP-SDS Western Myc_annotations.tif]

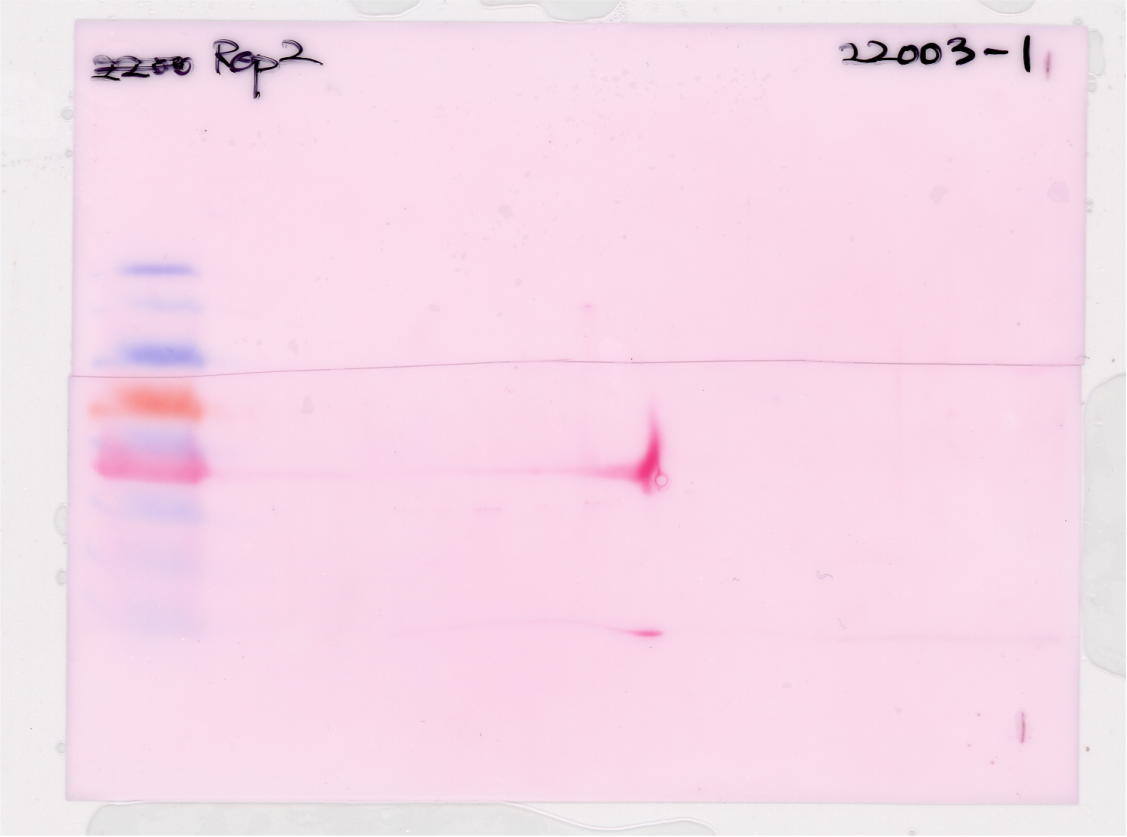

Supplement: Supplementary file 8 — Source Data for Figure 3 [file EMBJ-42-e111484-s006.zip › Figure 3/3B/2D BNP-SDS Western Ponceau.tif]

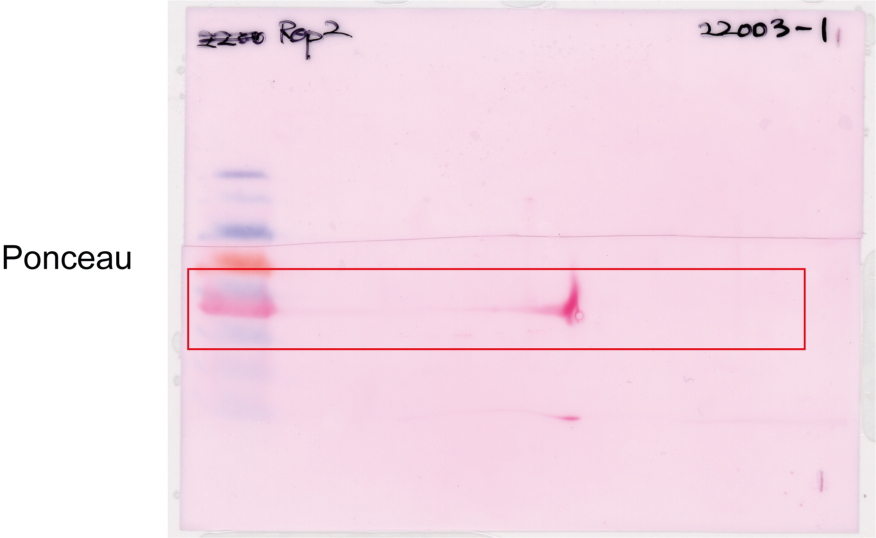

Supplement: Supplementary file 8 — Source Data for Figure 3 [file EMBJ-42-e111484-s006.zip › Figure 3/3B/2D BNP-SDS Western Ponceau_annotations.tif]

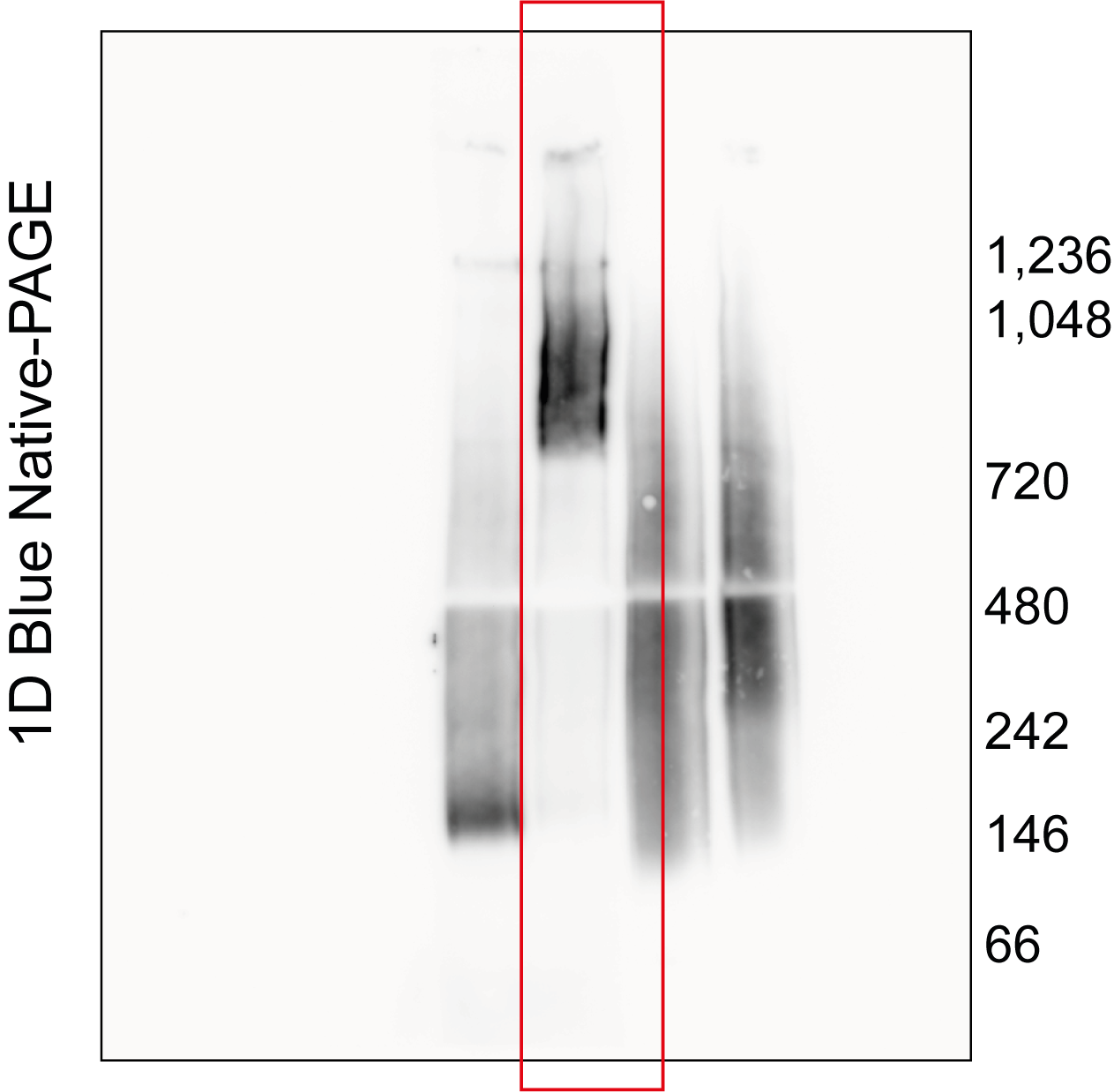

Supplement: Supplementary file 8 — Source Data for Figure 3 [file EMBJ-42-e111484-s006.zip › Figure 3/3C/1D BNP Western Myc_annotations.tif]

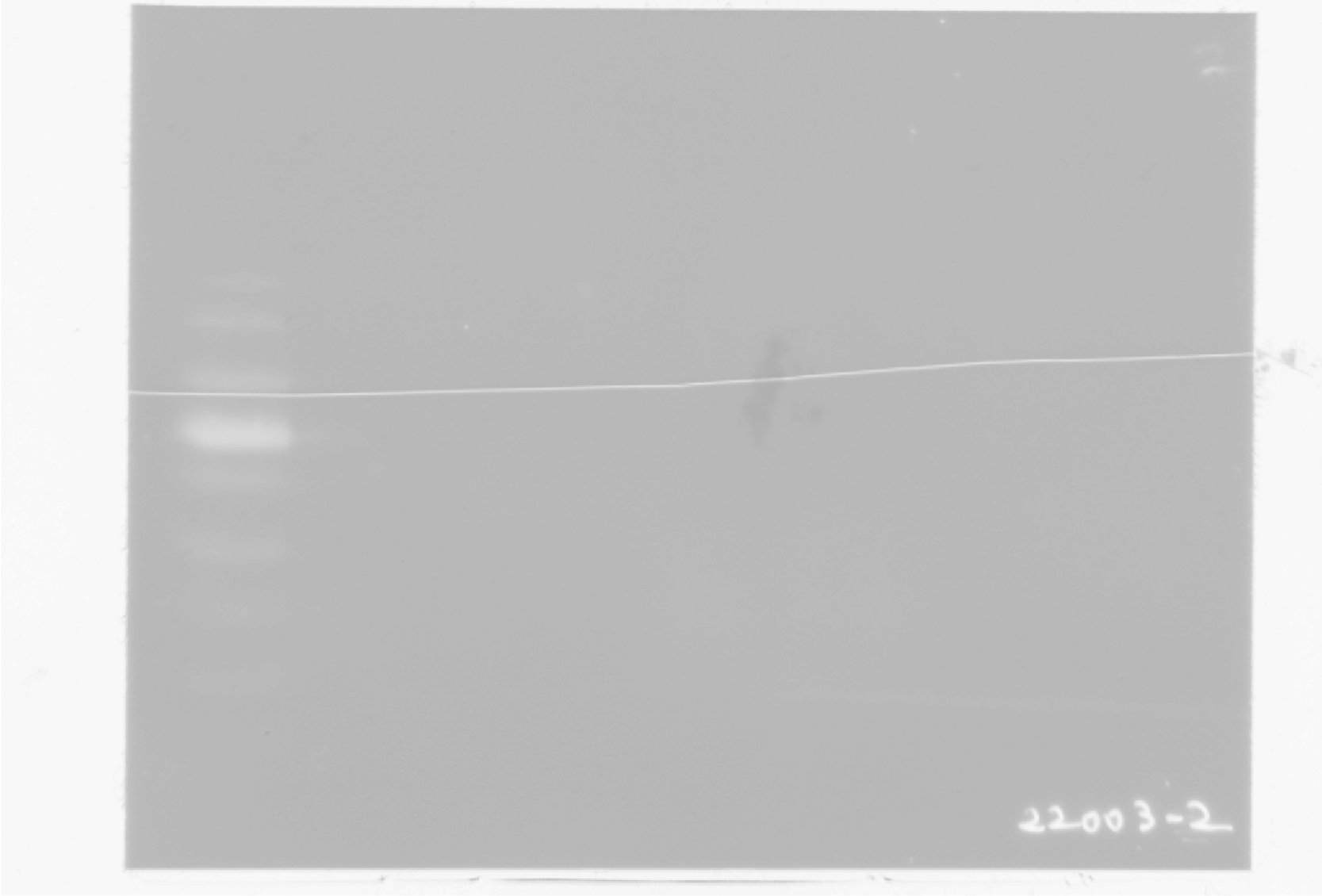

Supplement: Supplementary file 8 — Source Data for Figure 3 [file EMBJ-42-e111484-s006.zip › Figure 3/3C/2D BNP-SDS Western Flag Marker.tif]

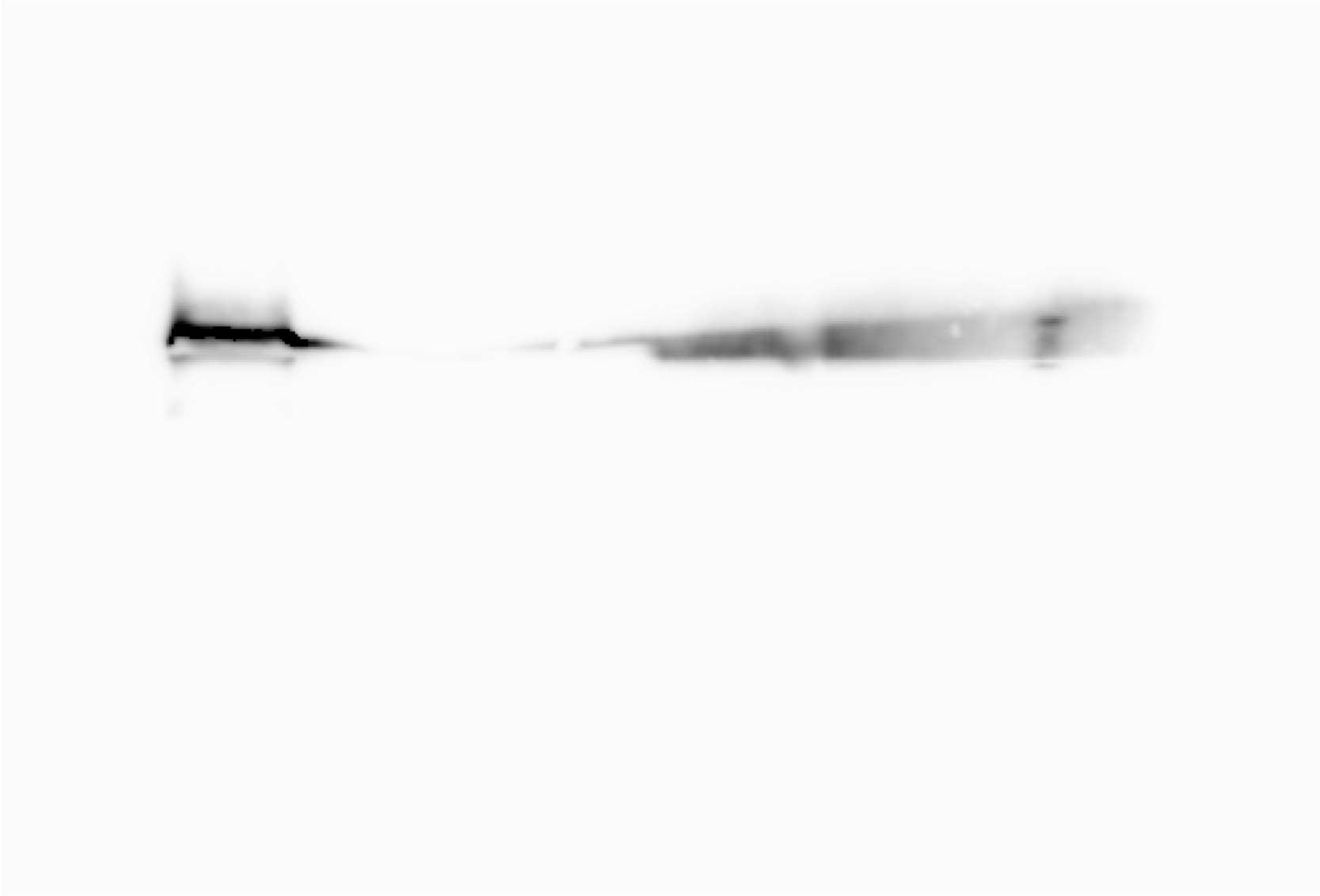

Supplement: Supplementary file 8 — Source Data for Figure 3 [file EMBJ-42-e111484-s006.zip › Figure 3/3C/2D BNP-SDS Western Flag.tif]

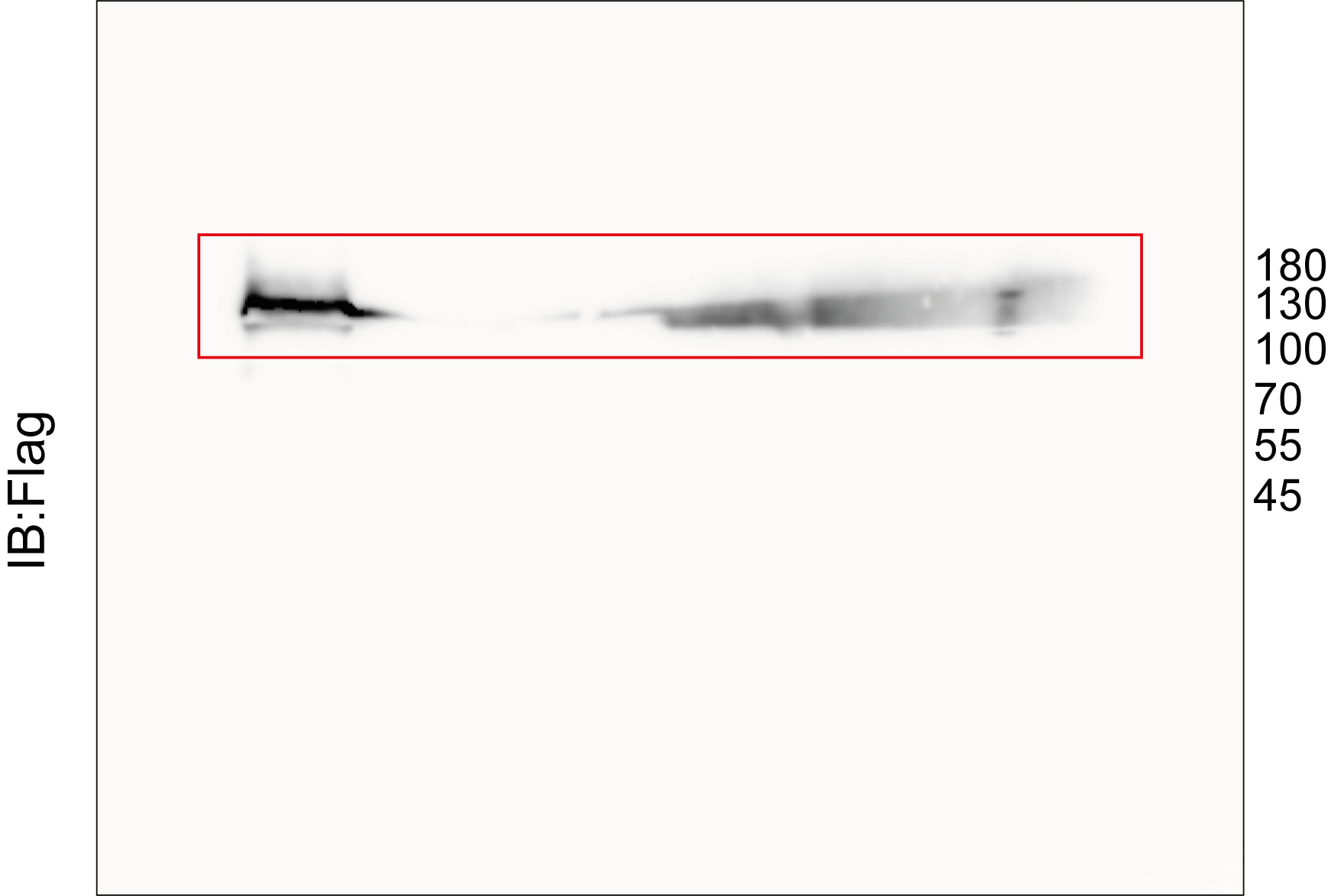

Supplement: Supplementary file 8 — Source Data for Figure 3 [file EMBJ-42-e111484-s006.zip › Figure 3/3C/2D BNP-SDS Western Flag_annotations.tif]

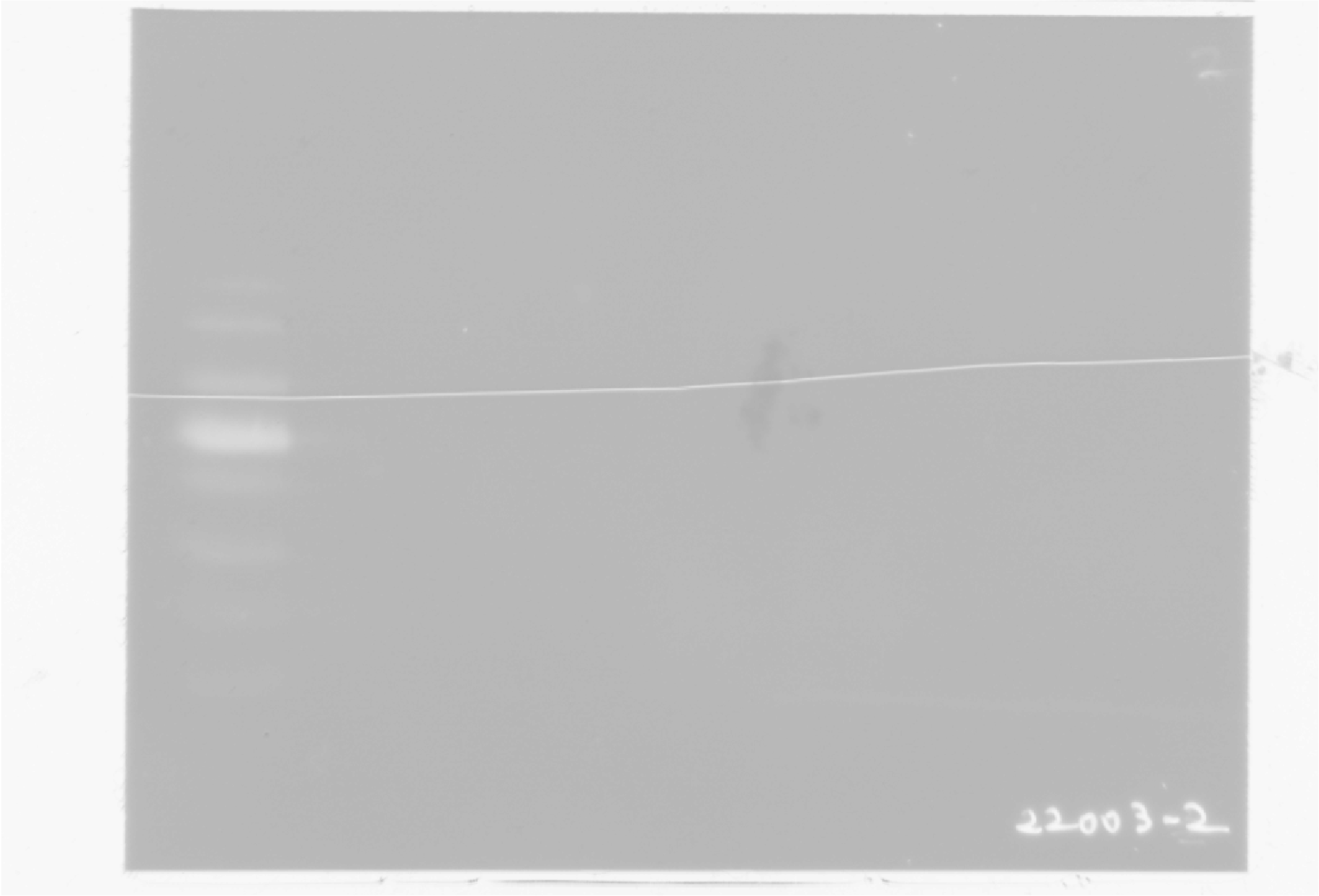

Supplement: Supplementary file 8 — Source Data for Figure 3 [file EMBJ-42-e111484-s006.zip › Figure 3/3C/2D BNP-SDS Western Myc Marker.tif]

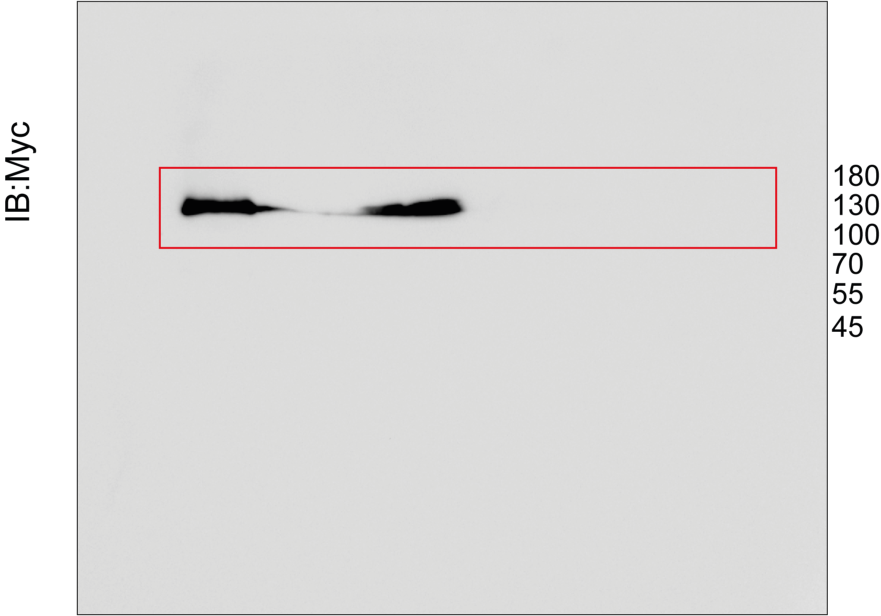

Supplement: Supplementary file 8 — Source Data for Figure 3 [file EMBJ-42-e111484-s006.zip › Figure 3/3C/2D BNP-SDS Western Myc_annotations.tif]

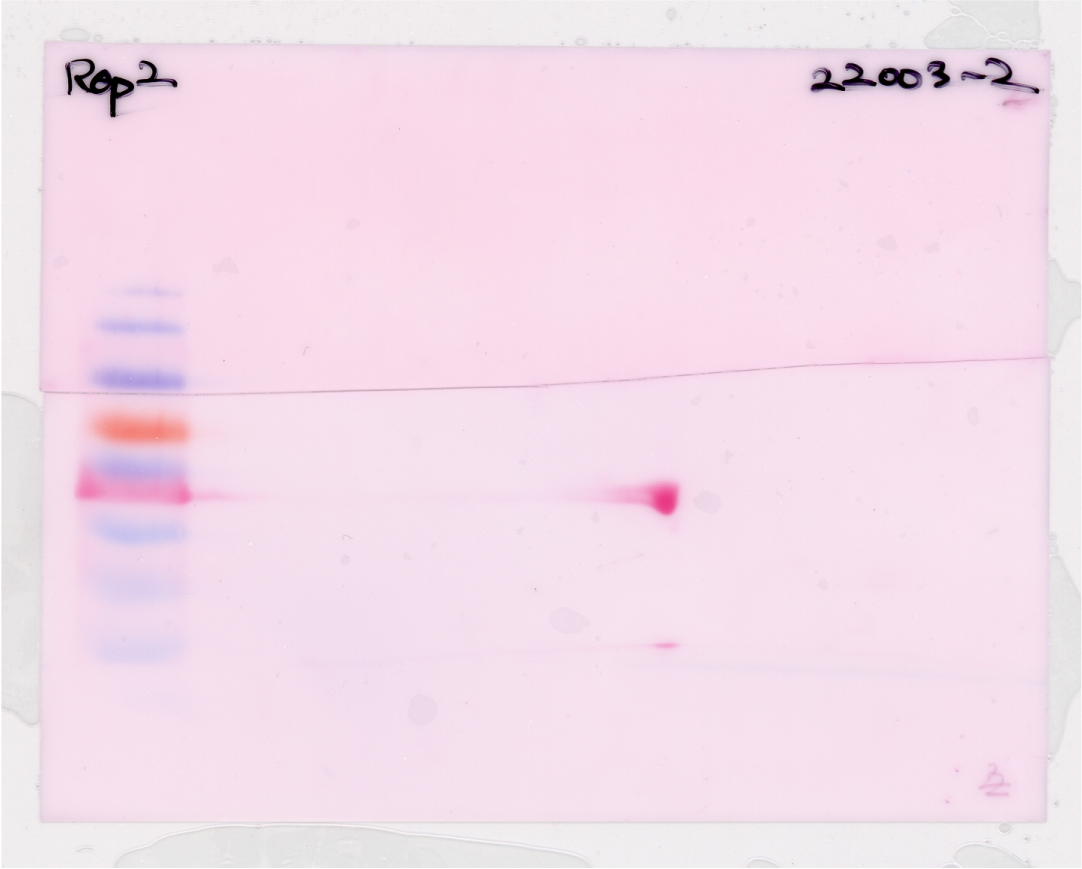

Supplement: Supplementary file 8 — Source Data for Figure 3 [file EMBJ-42-e111484-s006.zip › Figure 3/3C/2D BNP-SDS Western Ponceau.tif]

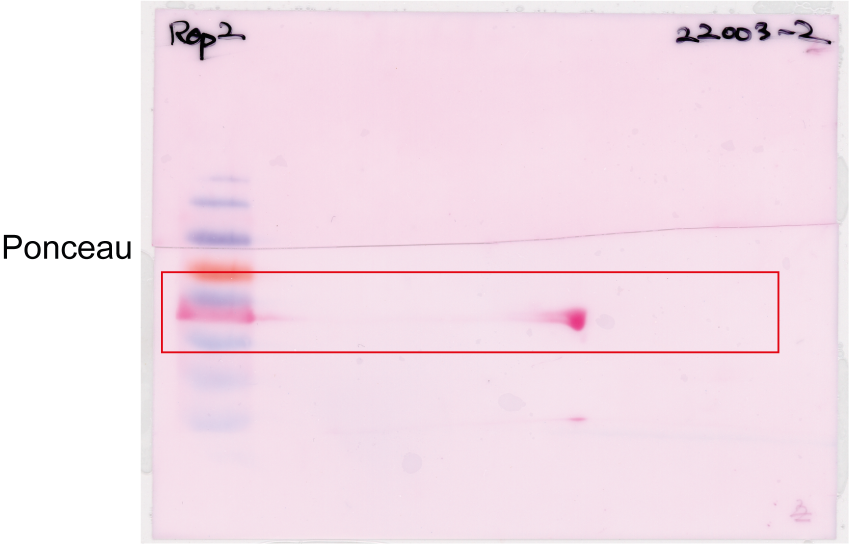

Supplement: Supplementary file 8 — Source Data for Figure 3 [file EMBJ-42-e111484-s006.zip › Figure 3/3C/2D BNP-SDS Western Ponceau_annotations.tif]

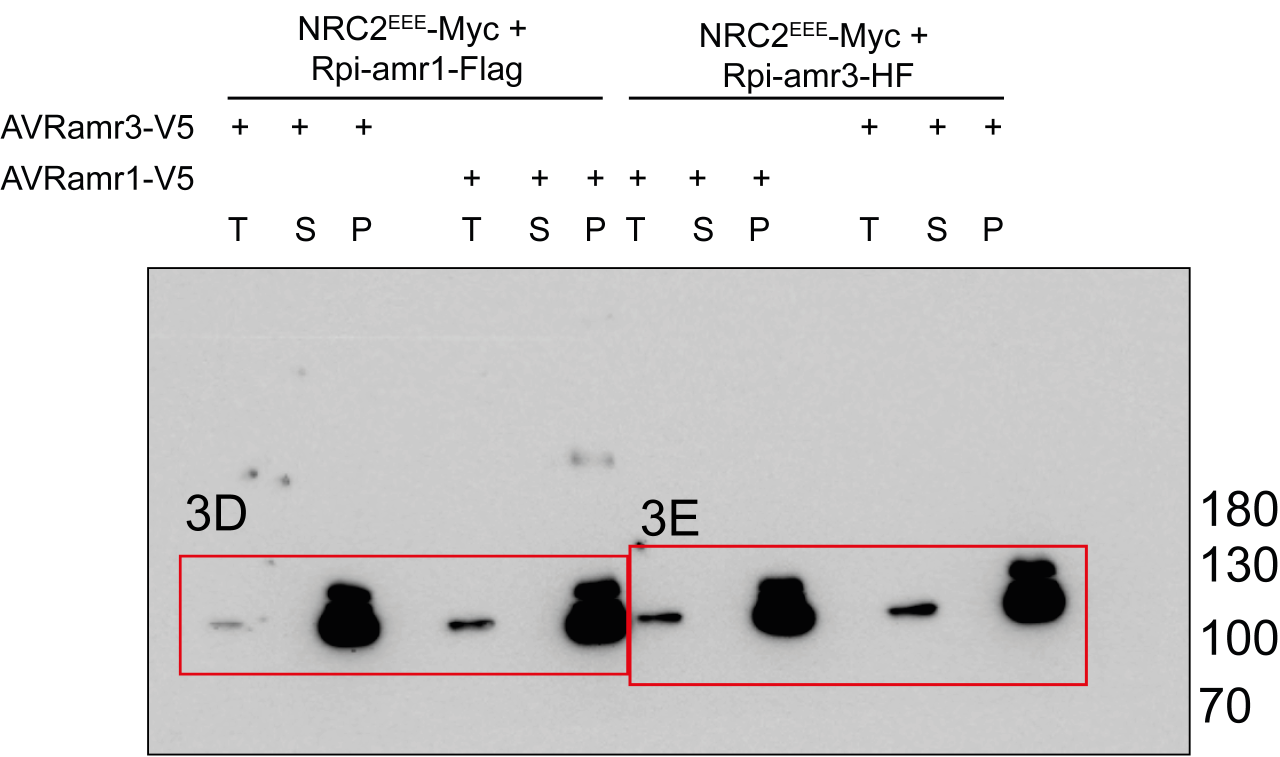

Supplement: Supplementary file 8 — Source Data for Figure 3 [file EMBJ-42-e111484-s006.zip › Figure 3/3D,E/3D, E Western ATPase_annotations.tif]

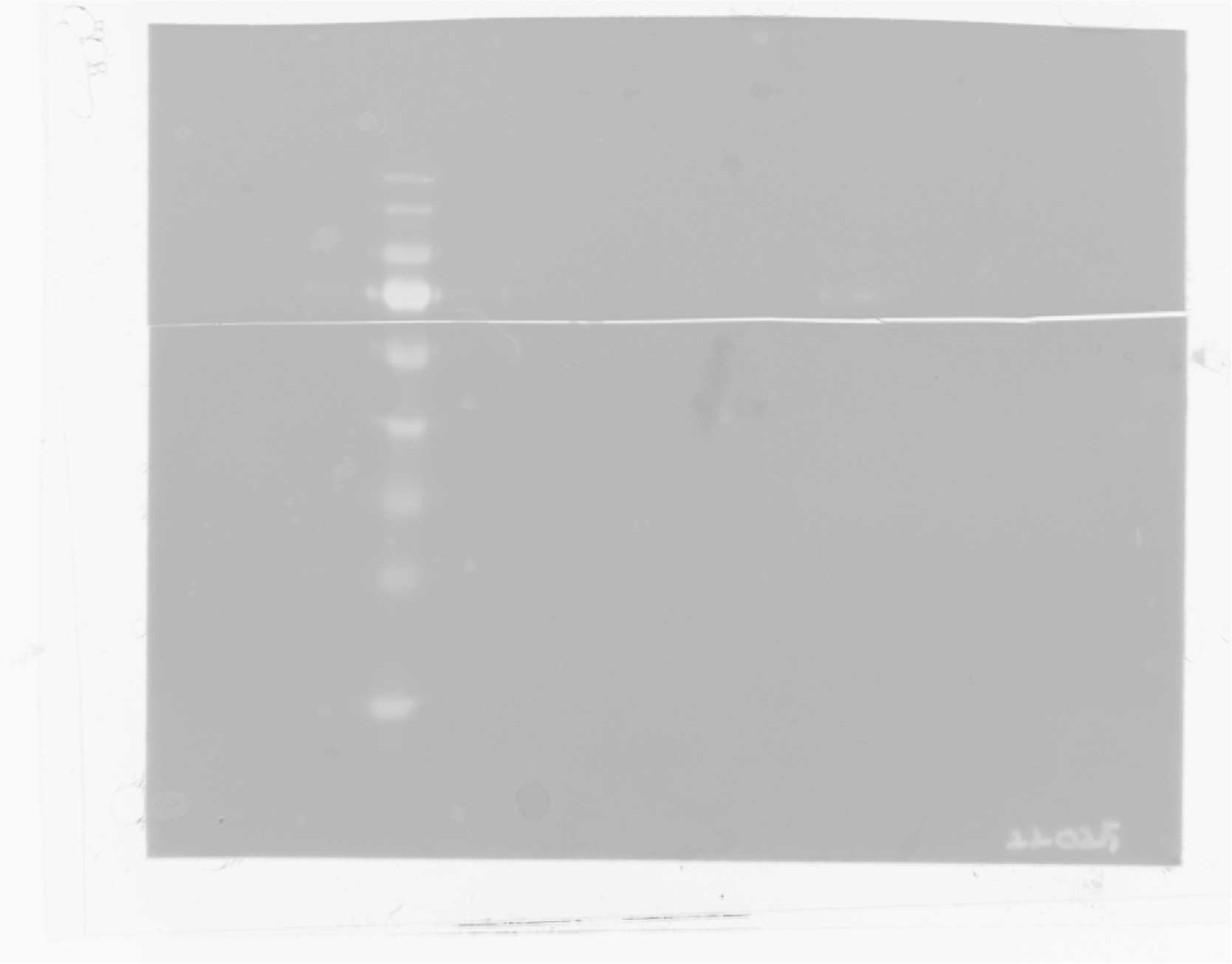

Supplement: Supplementary file 8 — Source Data for Figure 3 [file EMBJ-42-e111484-s006.zip › Figure 3/3D,E/3D, E Western Flag Marker.tif]

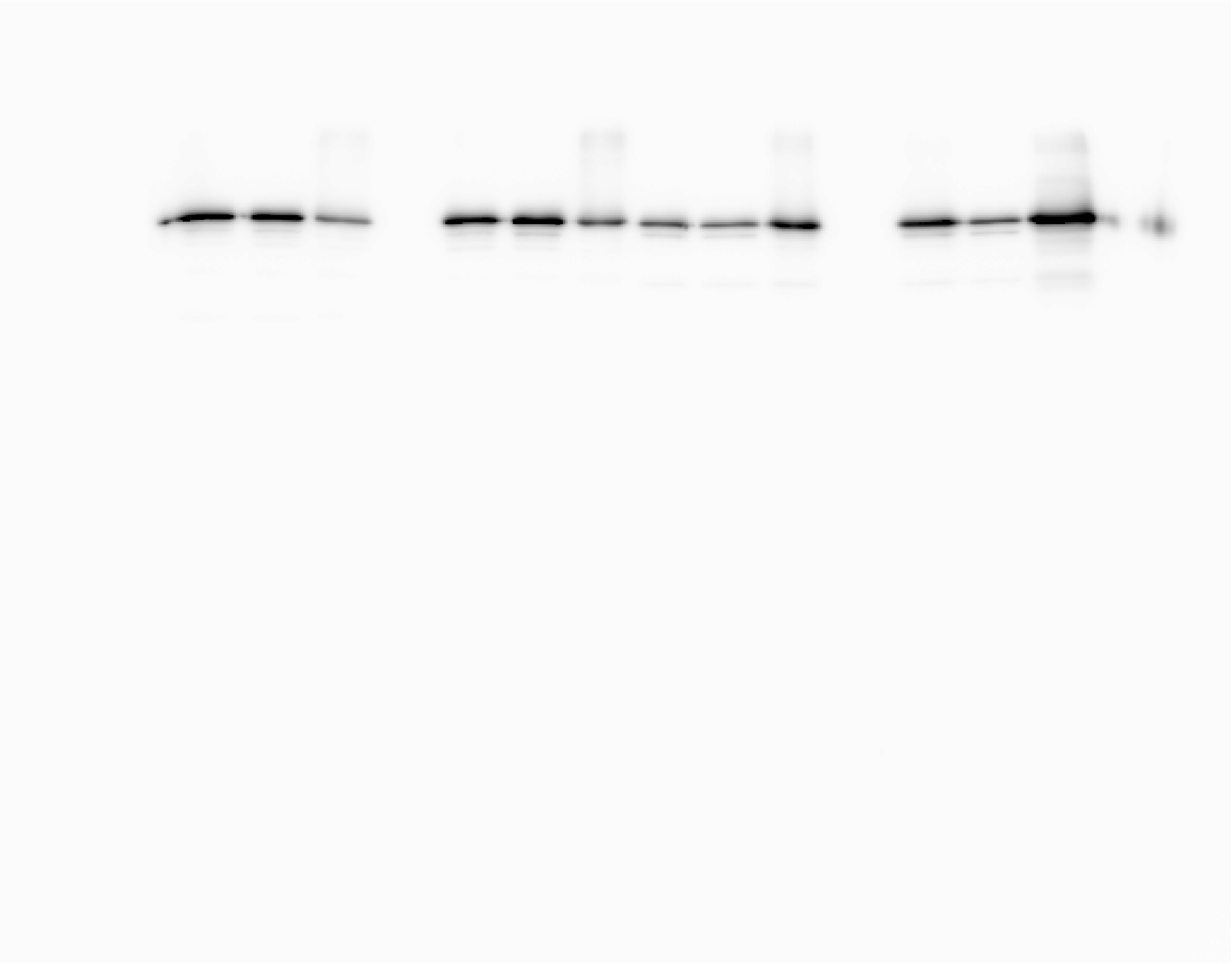

Supplement: Supplementary file 8 — Source Data for Figure 3 [file EMBJ-42-e111484-s006.zip › Figure 3/3D,E/3D, E Western Flag.tif]

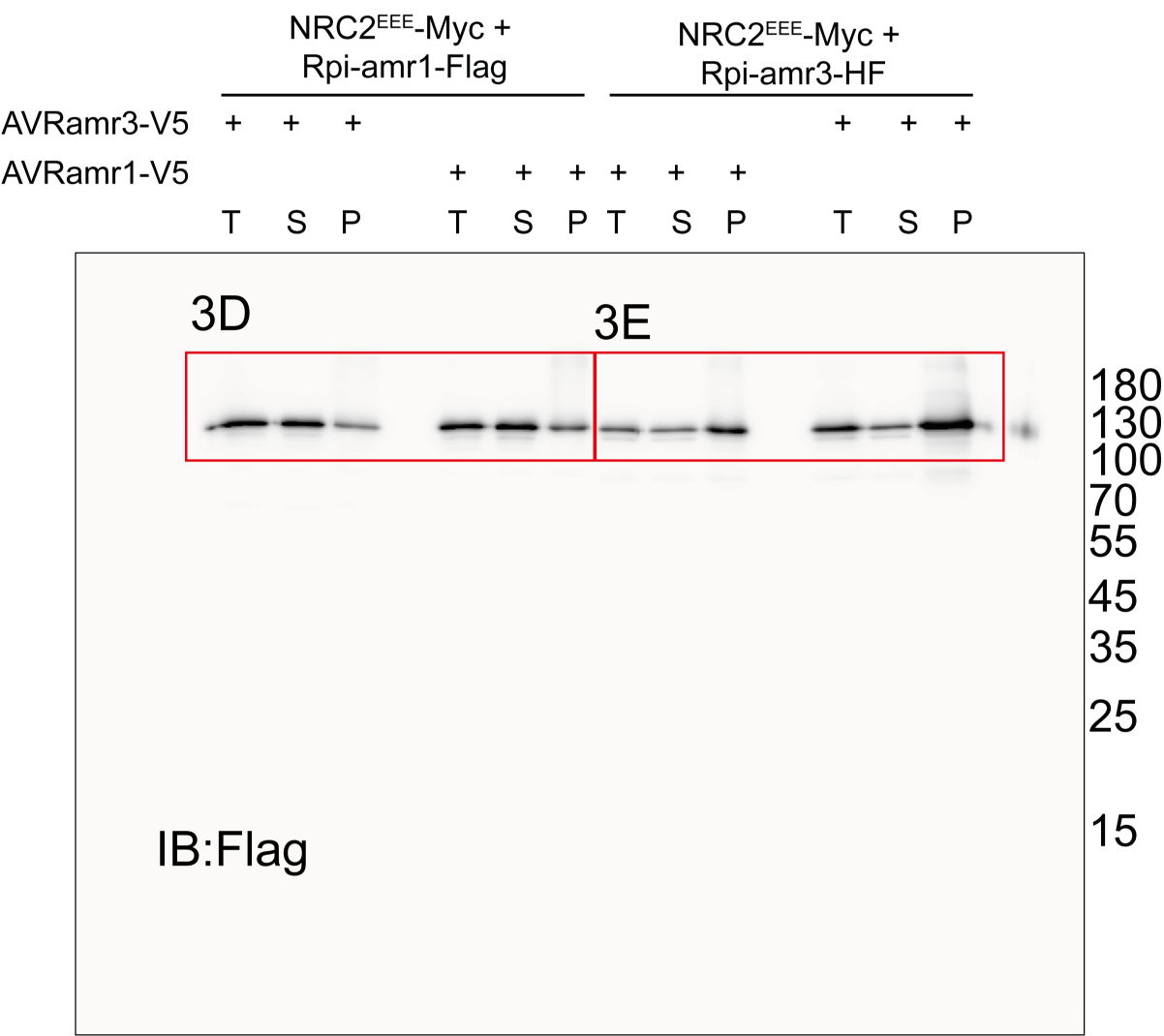

Supplement: Supplementary file 8 — Source Data for Figure 3 [file EMBJ-42-e111484-s006.zip › Figure 3/3D,E/3D, E Western Flag_annotations.tif]

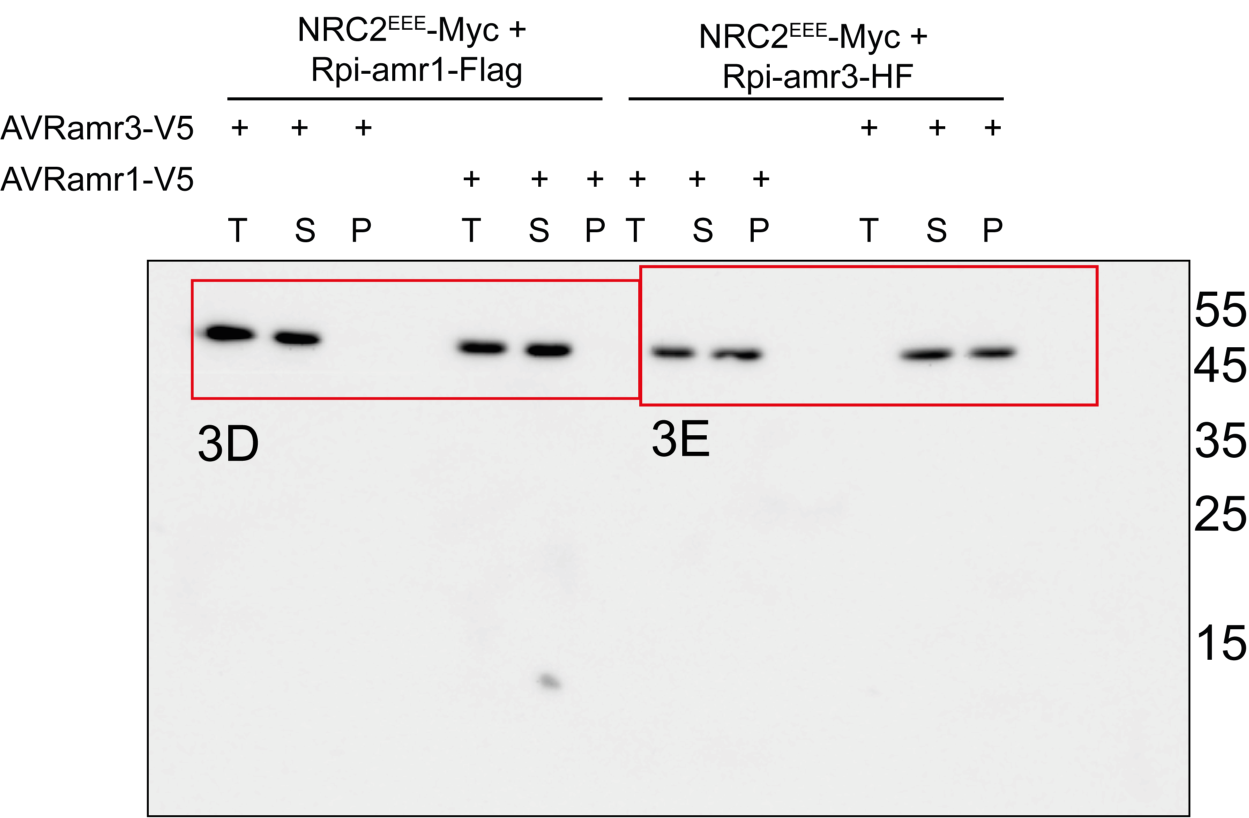

Supplement: Supplementary file 8 — Source Data for Figure 3 [file EMBJ-42-e111484-s006.zip › Figure 3/3D,E/3D, E Western MPK6_annotations.tif]

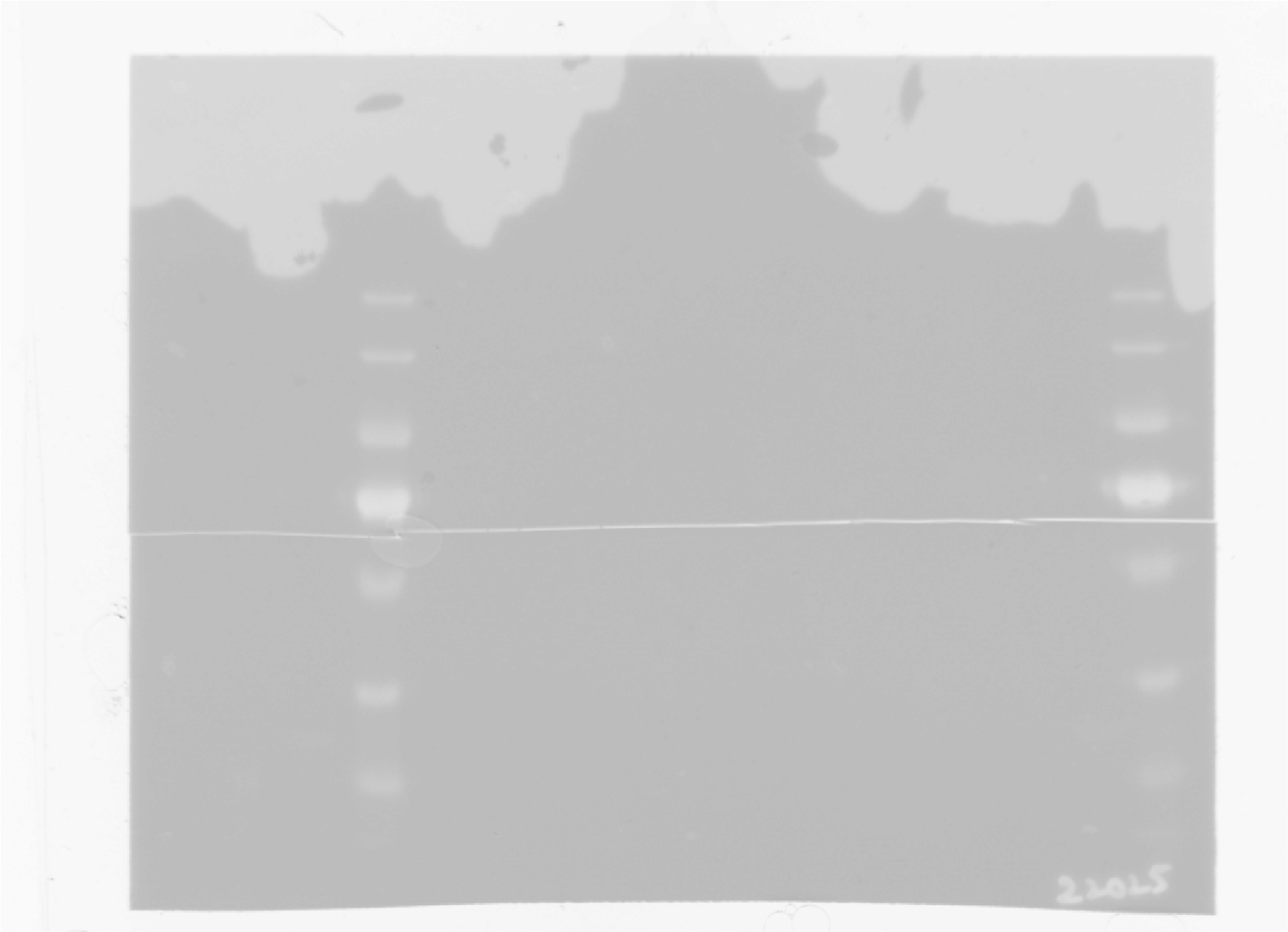

Supplement: Supplementary file 8 — Source Data for Figure 3 [file EMBJ-42-e111484-s006.zip › Figure 3/3D,E/3D, E Western Myc,V5 Marker.tif]

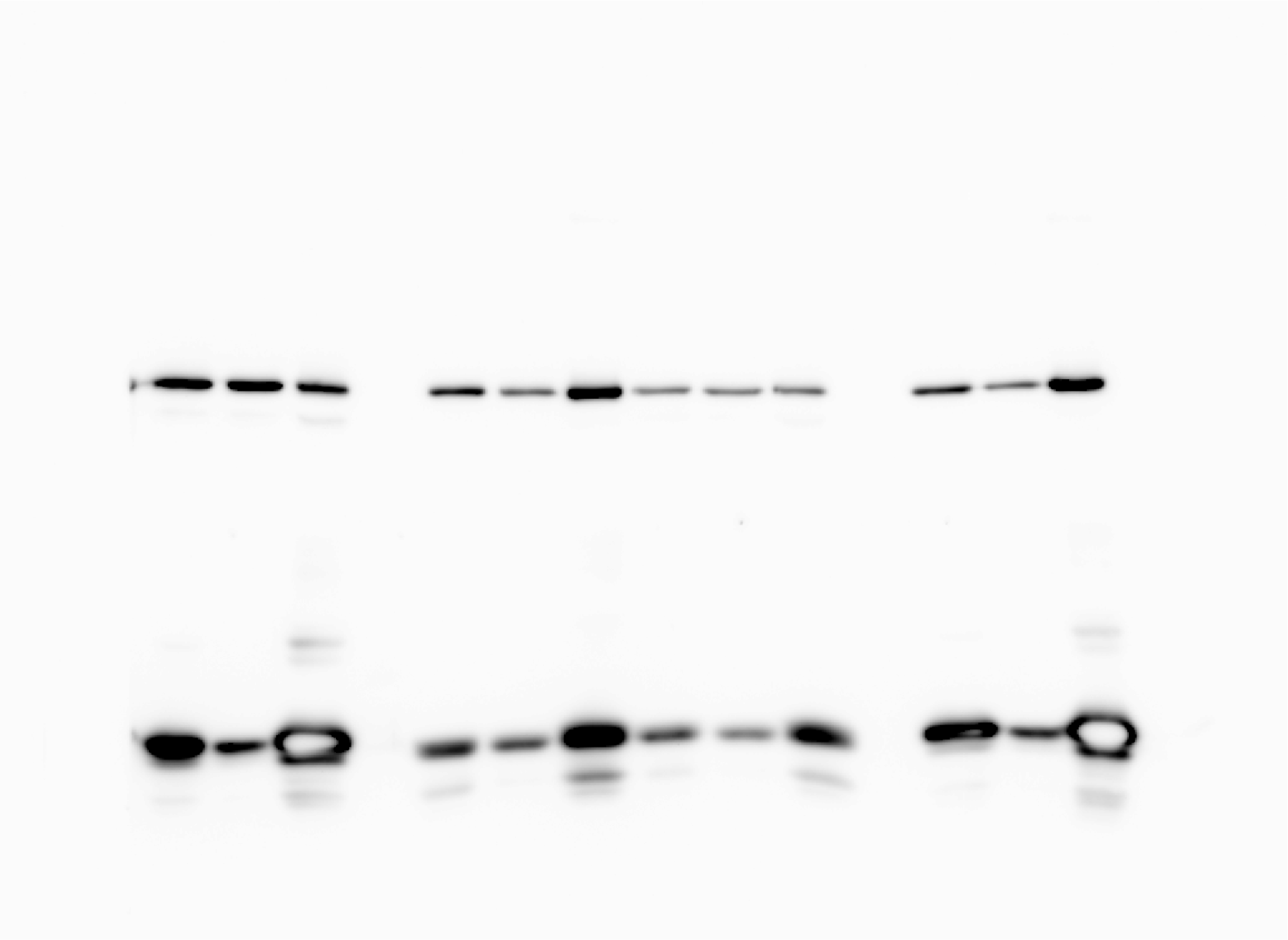

Supplement: Supplementary file 8 — Source Data for Figure 3 [file EMBJ-42-e111484-s006.zip › Figure 3/3D,E/3D, E Western Myc.tif]

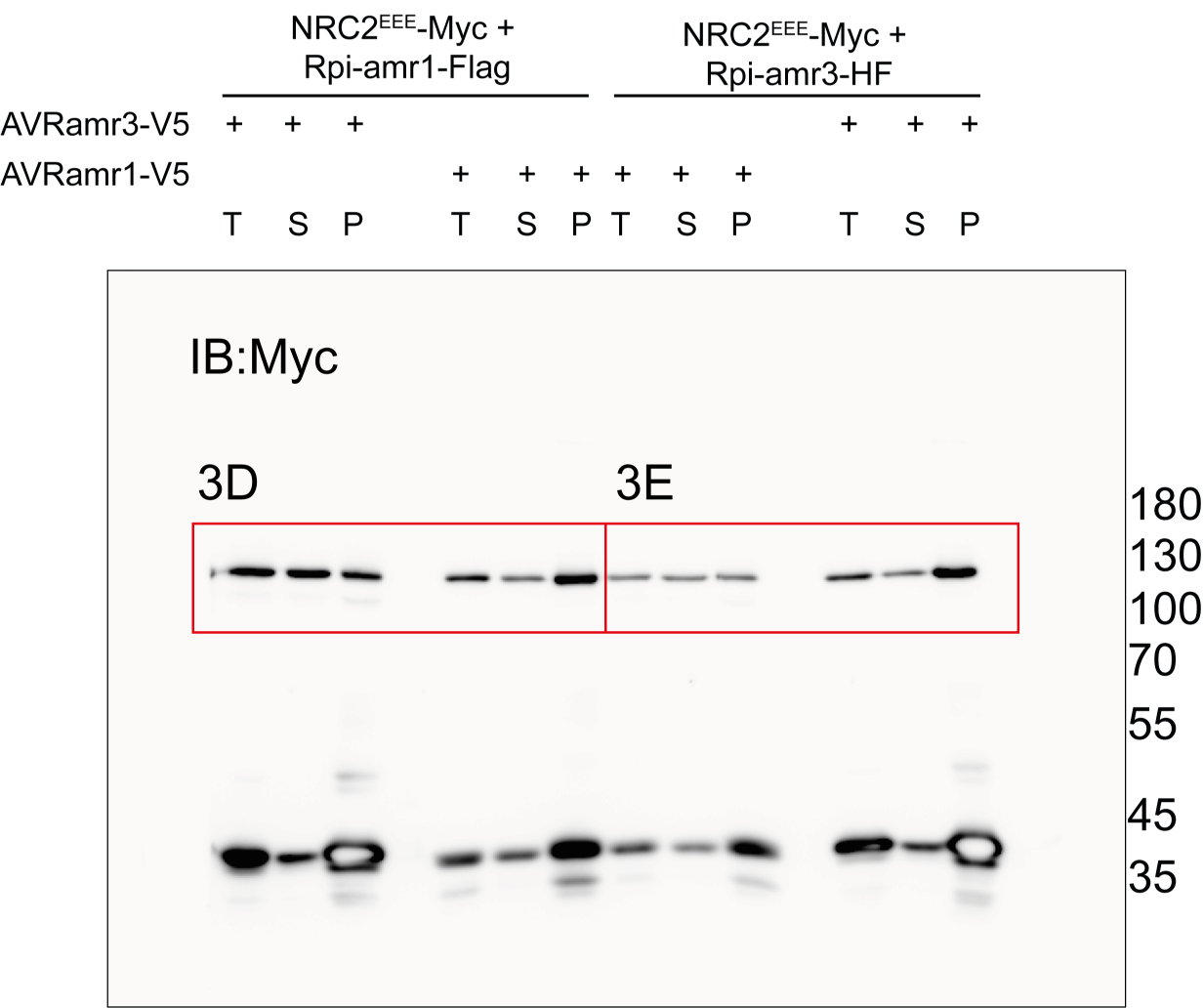

Supplement: Supplementary file 8 — Source Data for Figure 3 [file EMBJ-42-e111484-s006.zip › Figure 3/3D,E/3D, E Western Myc_annotations.tif]

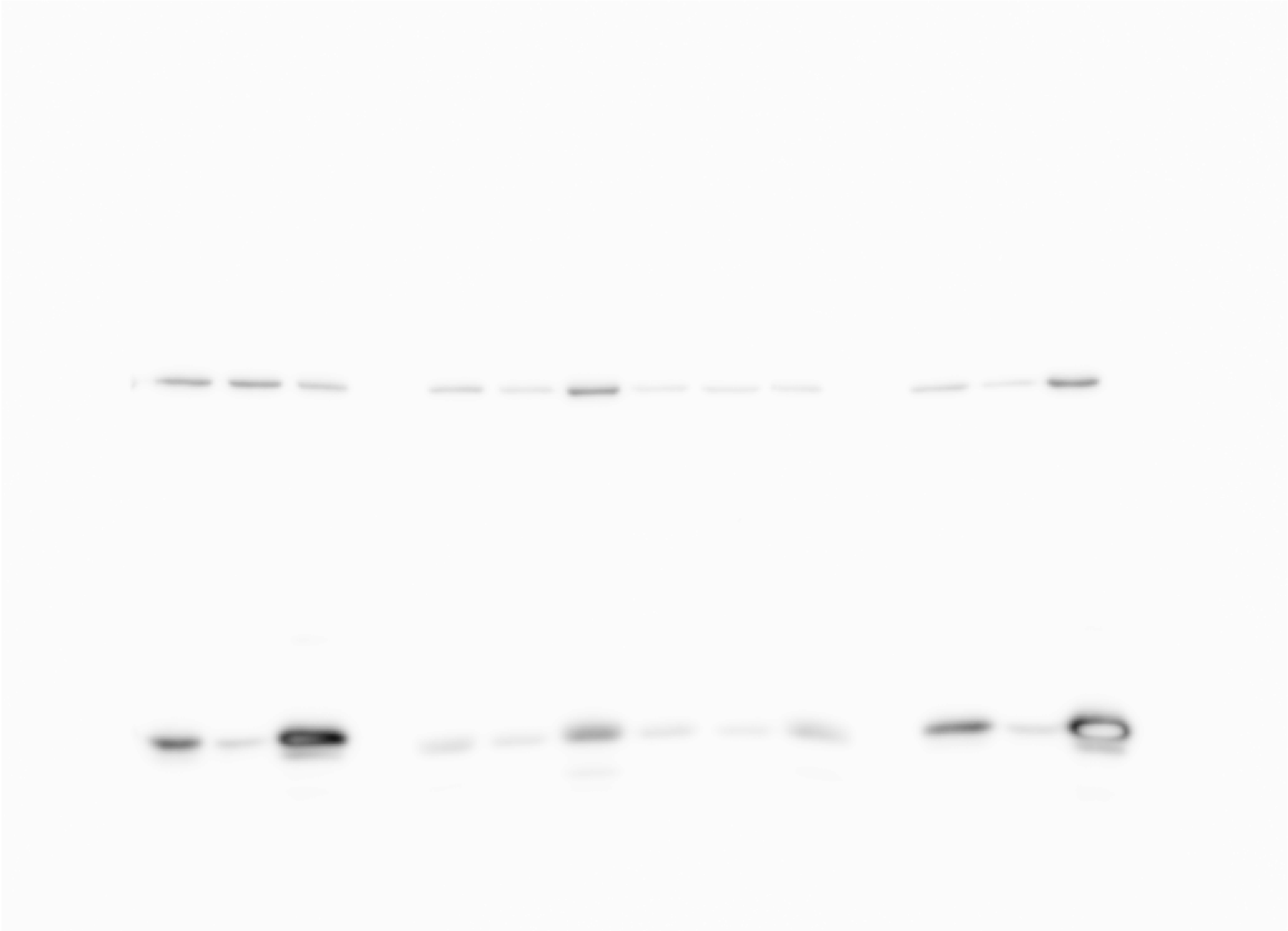

Supplement: Supplementary file 8 — Source Data for Figure 3 [file EMBJ-42-e111484-s006.zip › Figure 3/3D,E/3D, E Western V5.tif]

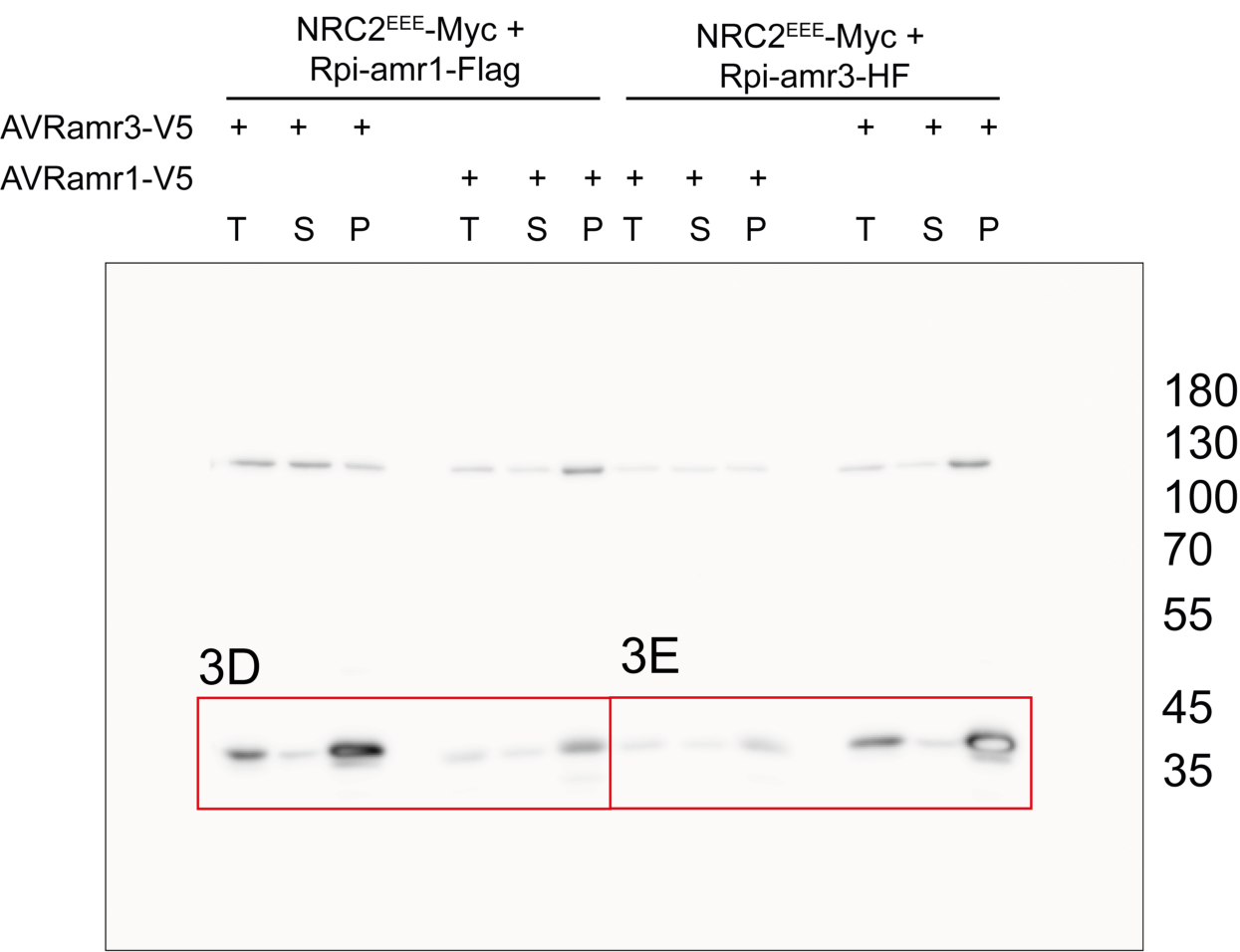

Supplement: Supplementary file 8 — Source Data for Figure 3 [file EMBJ-42-e111484-s006.zip › Figure 3/3D,E/3D, E Western V5_annotations.tif]

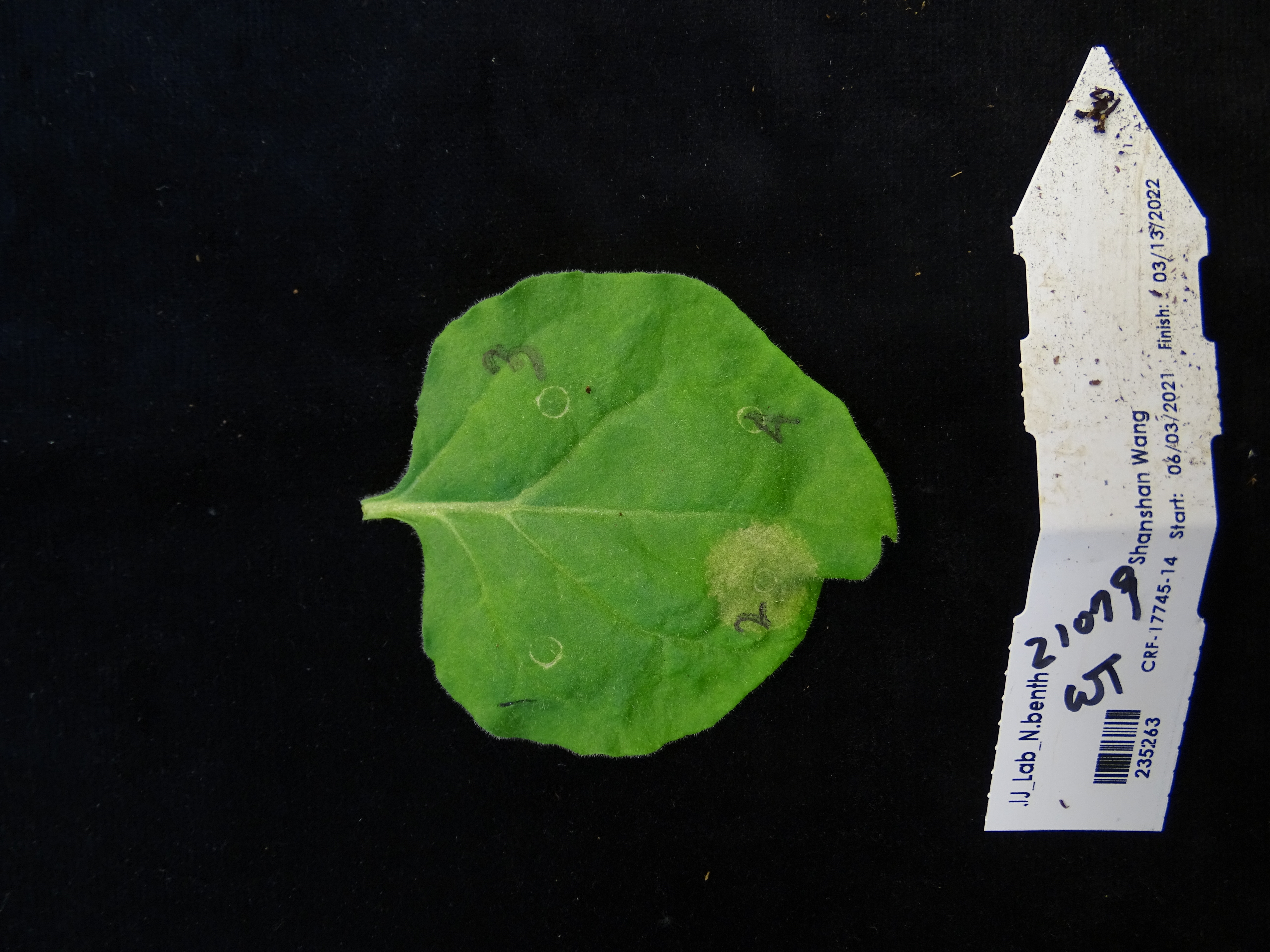

Supplement: Supplementary file 9 — Source Data for Figure 4 [file EMBJ-42-e111484-s003.zip › Figure 4/4A/DSC09795.JPG]

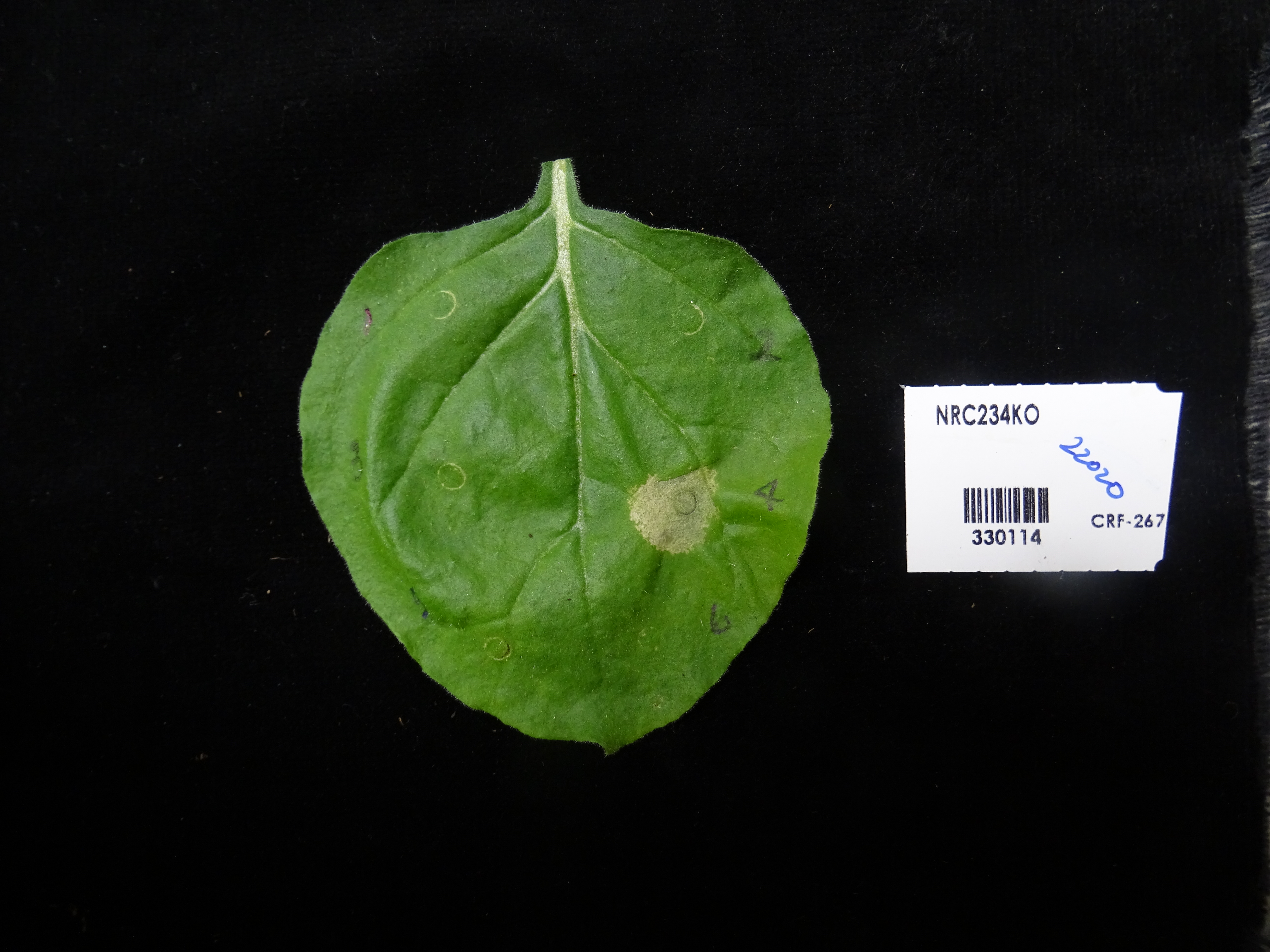

Supplement: Supplementary file 9 — Source Data for Figure 4 [file EMBJ-42-e111484-s003.zip › Figure 4/4B/DSC07697.JPG]

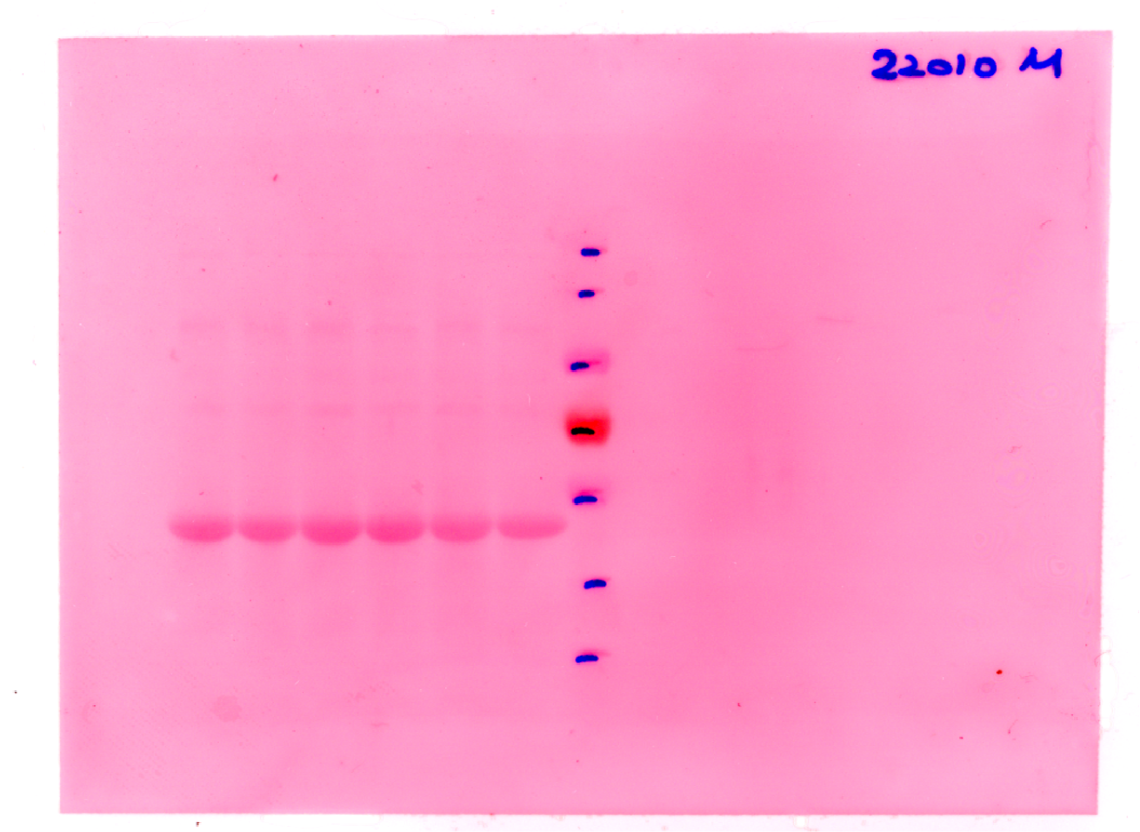

Supplement: Supplementary file 9 — Source Data for Figure 4 [file EMBJ-42-e111484-s003.zip › Figure 4/4C/BNP Western Myc Ponceau.tif]

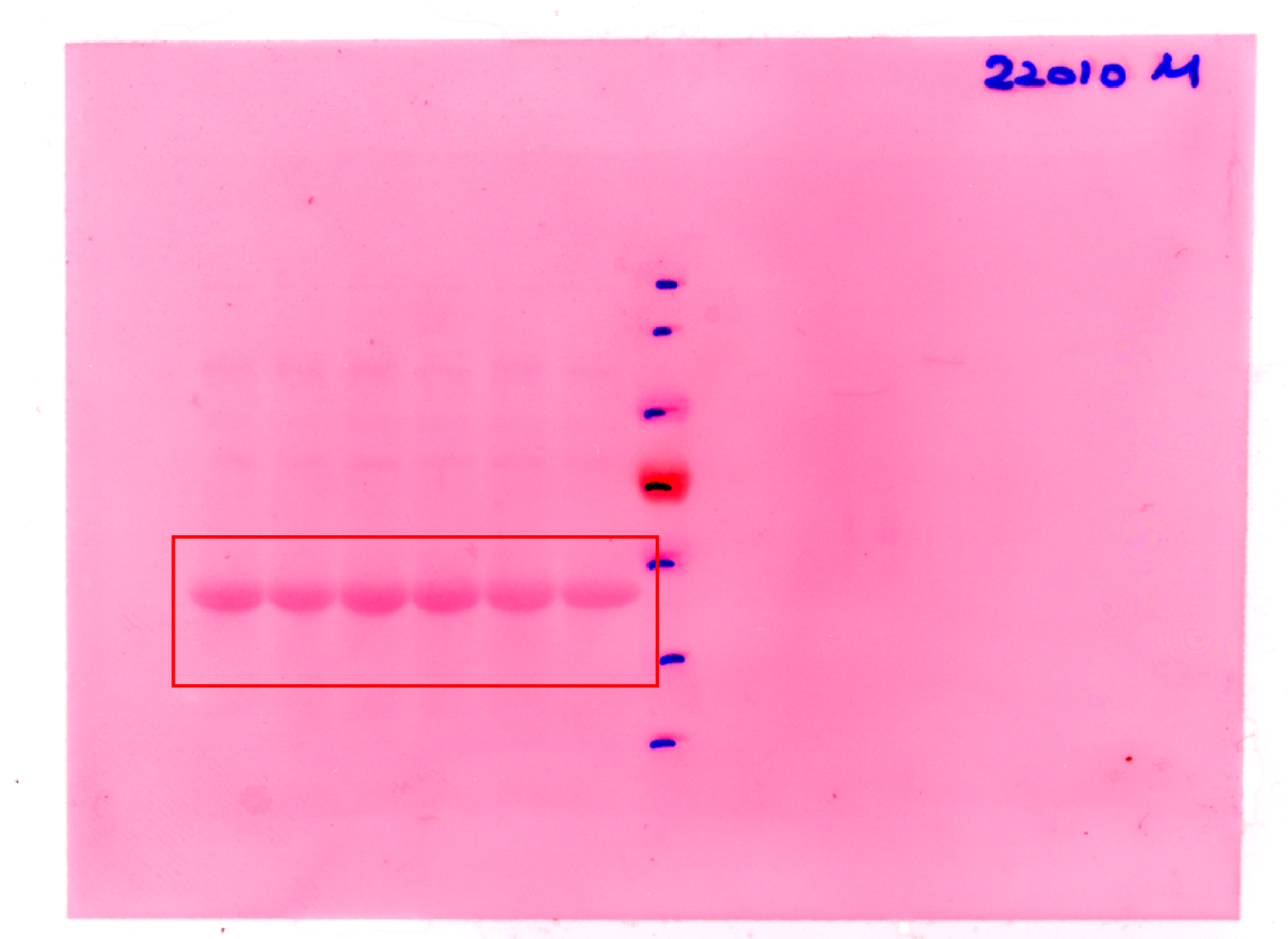

Supplement: Supplementary file 9 — Source Data for Figure 4 [file EMBJ-42-e111484-s003.zip › Figure 4/4C/BNP Western Myc Ponceau_annotations.tif]

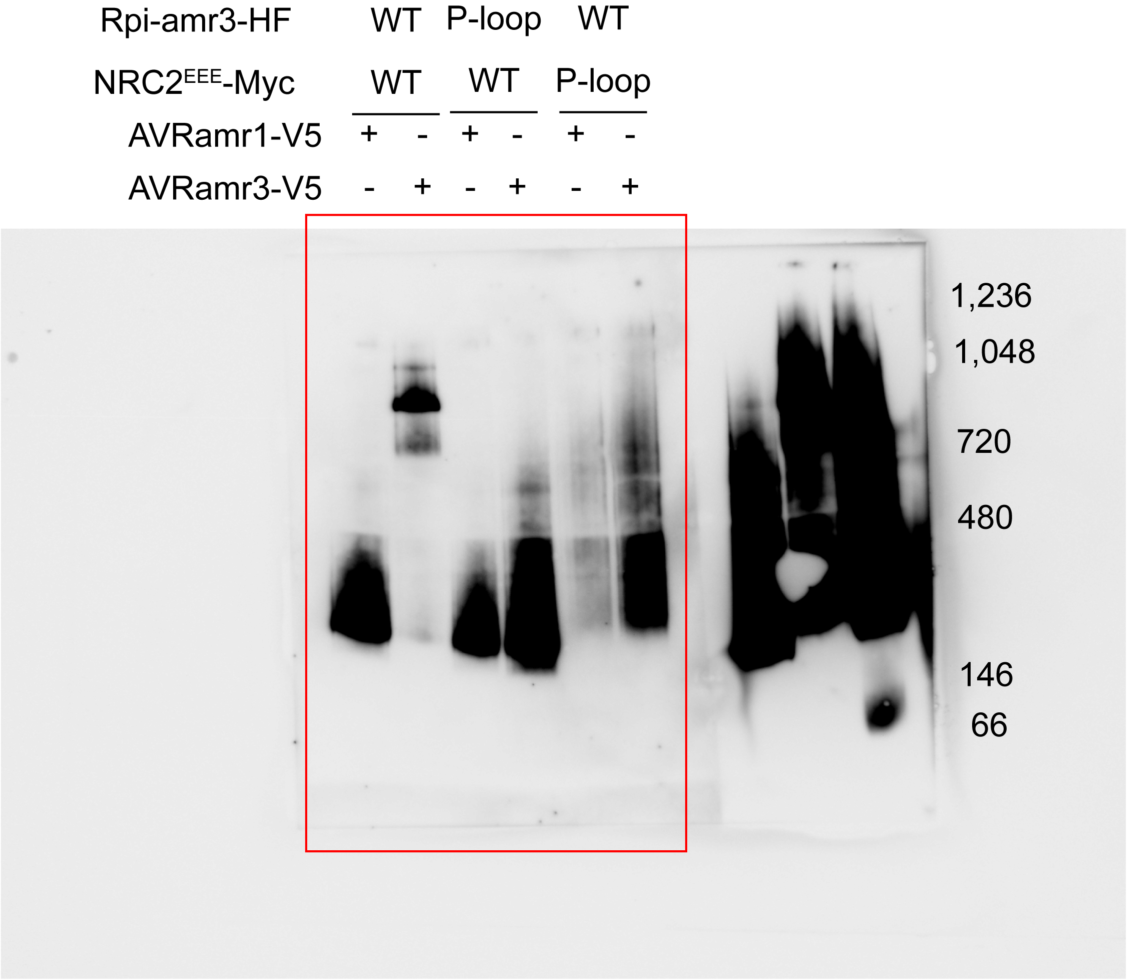

Supplement: Supplementary file 9 — Source Data for Figure 4 [file EMBJ-42-e111484-s003.zip › Figure 4/4C/BNP Western Myc_annotations.tif]

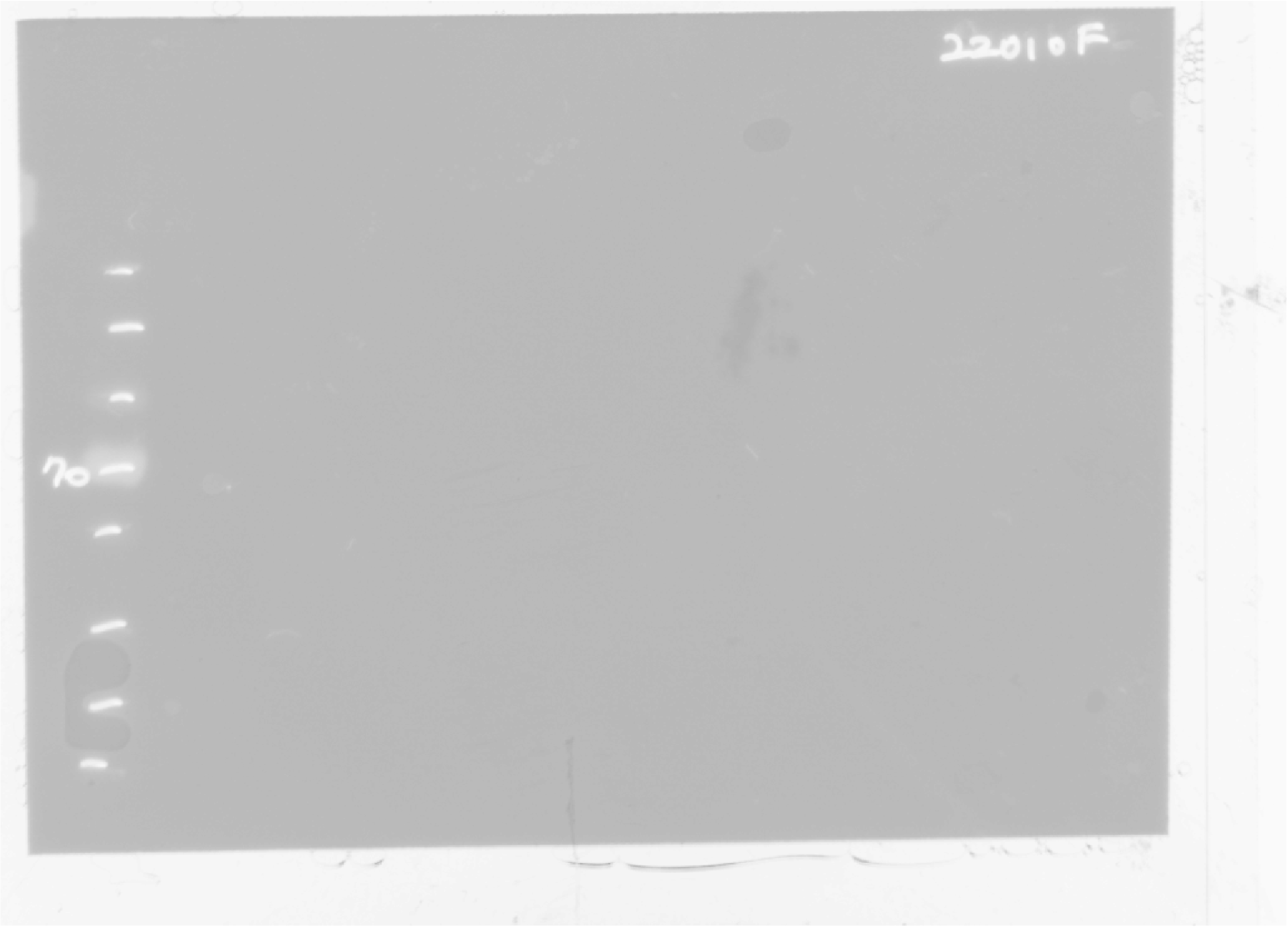

Supplement: Supplementary file 9 — Source Data for Figure 4 [file EMBJ-42-e111484-s003.zip › Figure 4/4C/SDS Western Flag Marker.tif]

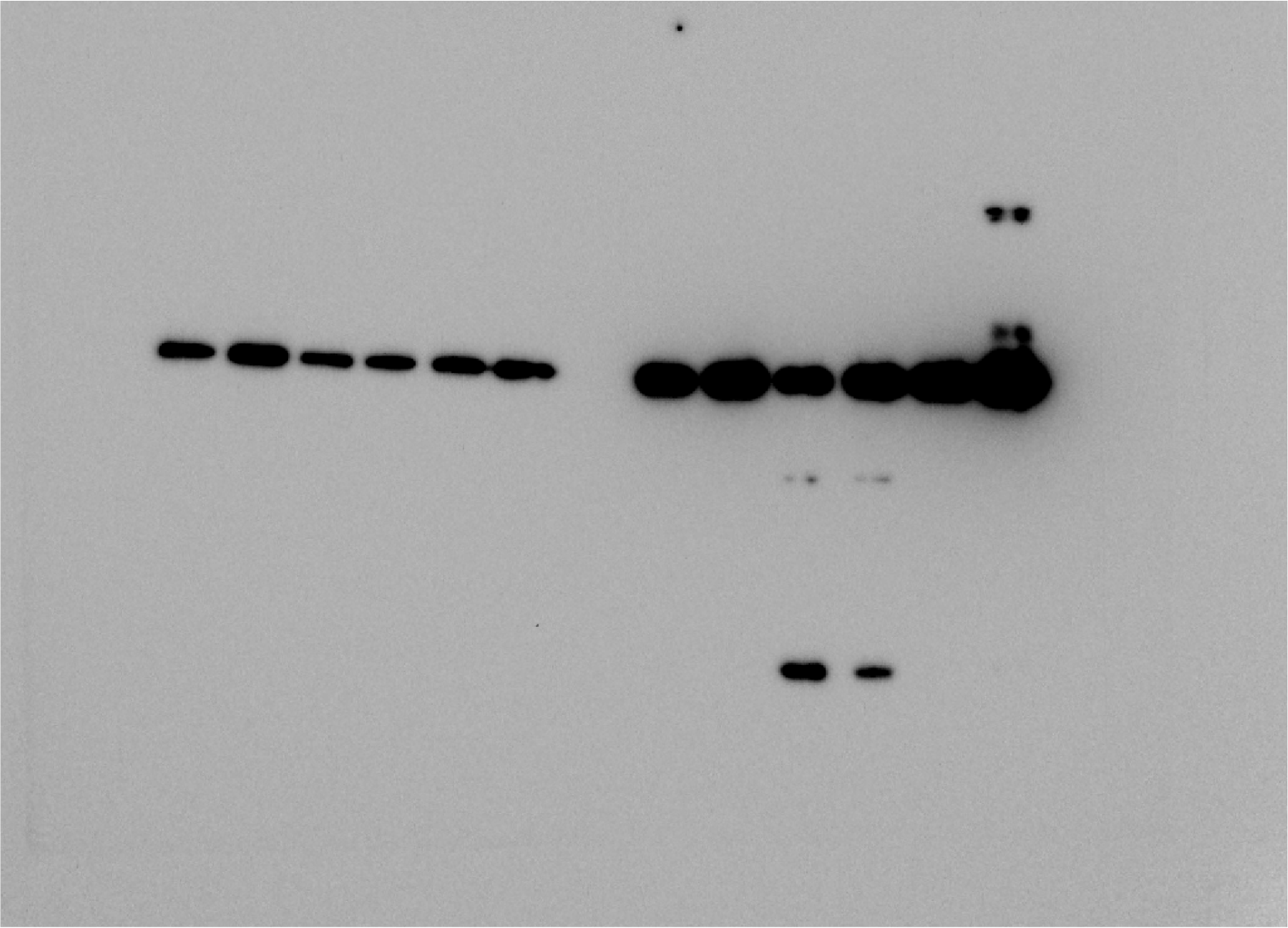

Supplement: Supplementary file 9 — Source Data for Figure 4 [file EMBJ-42-e111484-s003.zip › Figure 4/4C/SDS Western Flag.tif]

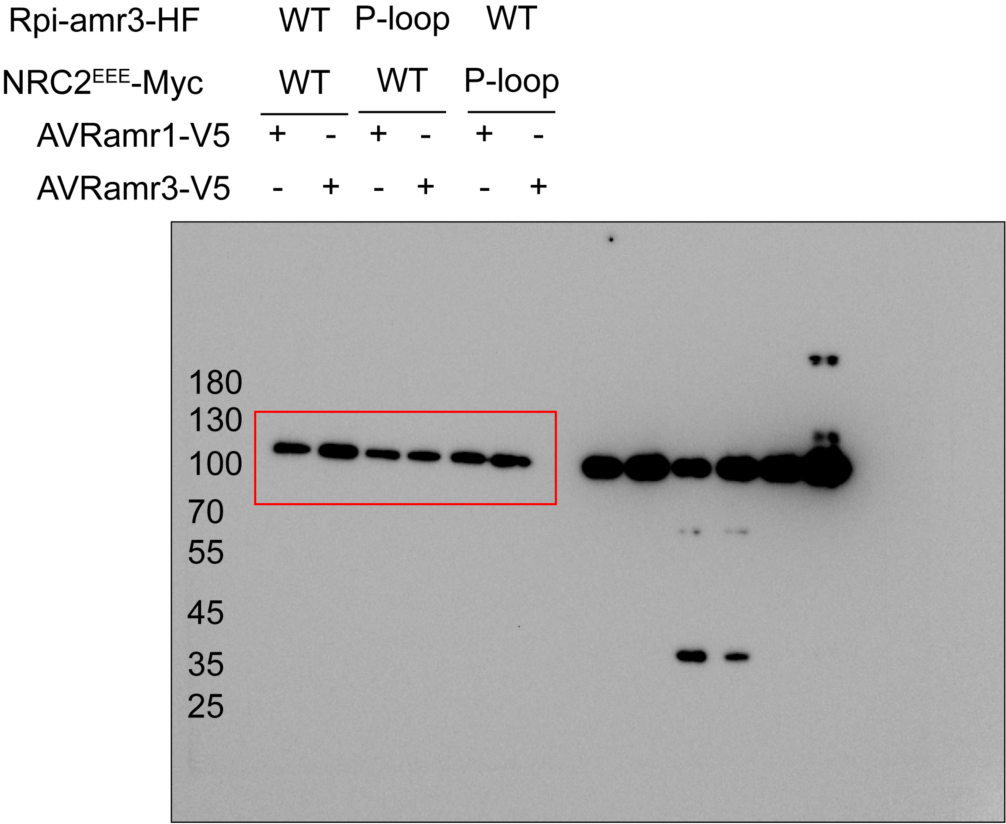

Supplement: Supplementary file 9 — Source Data for Figure 4 [file EMBJ-42-e111484-s003.zip › Figure 4/4C/SDS Western Flag_annotations.tif]

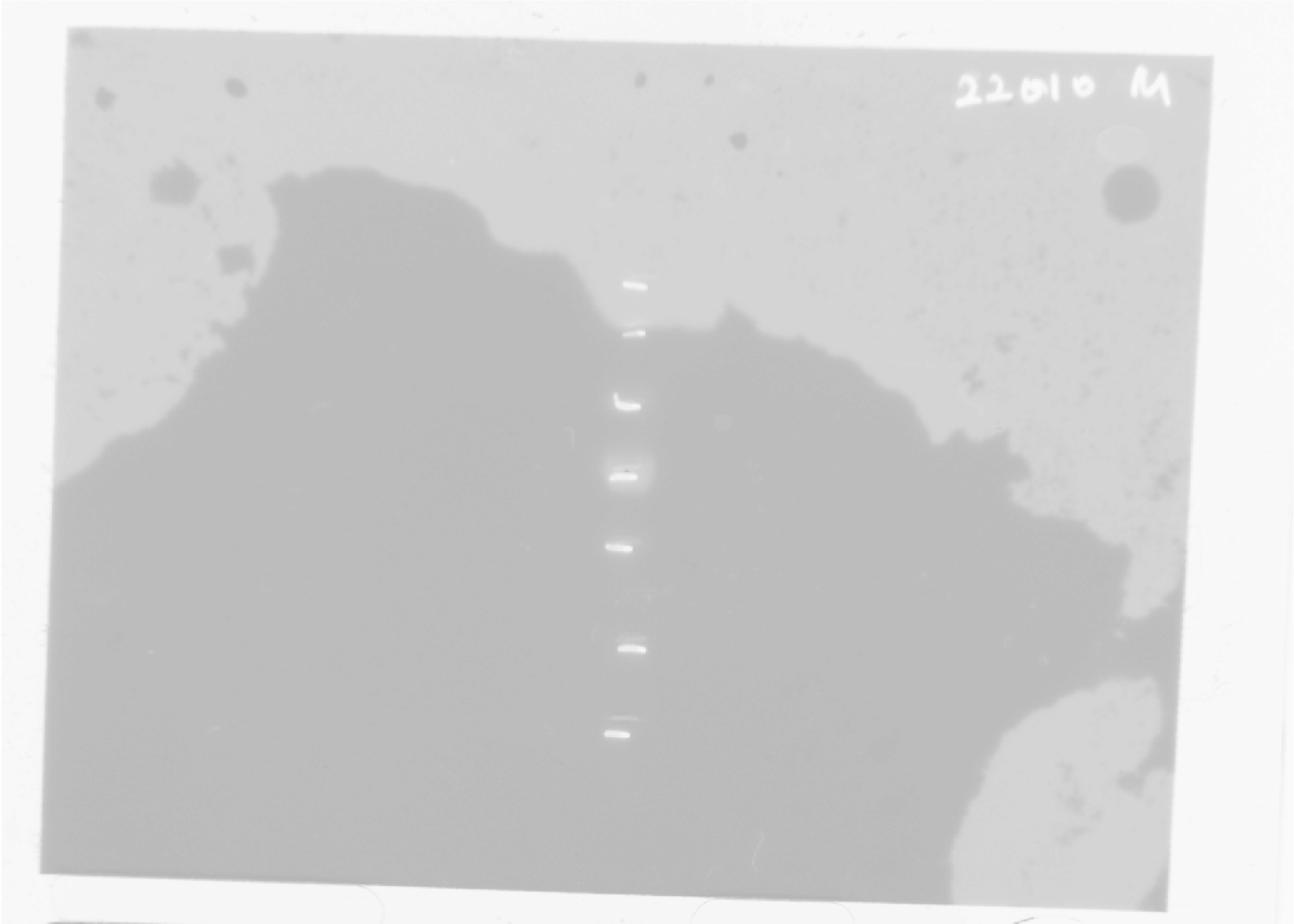

Supplement: Supplementary file 9 — Source Data for Figure 4 [file EMBJ-42-e111484-s003.zip › Figure 4/4C/SDS Western Myc Marker.tif]

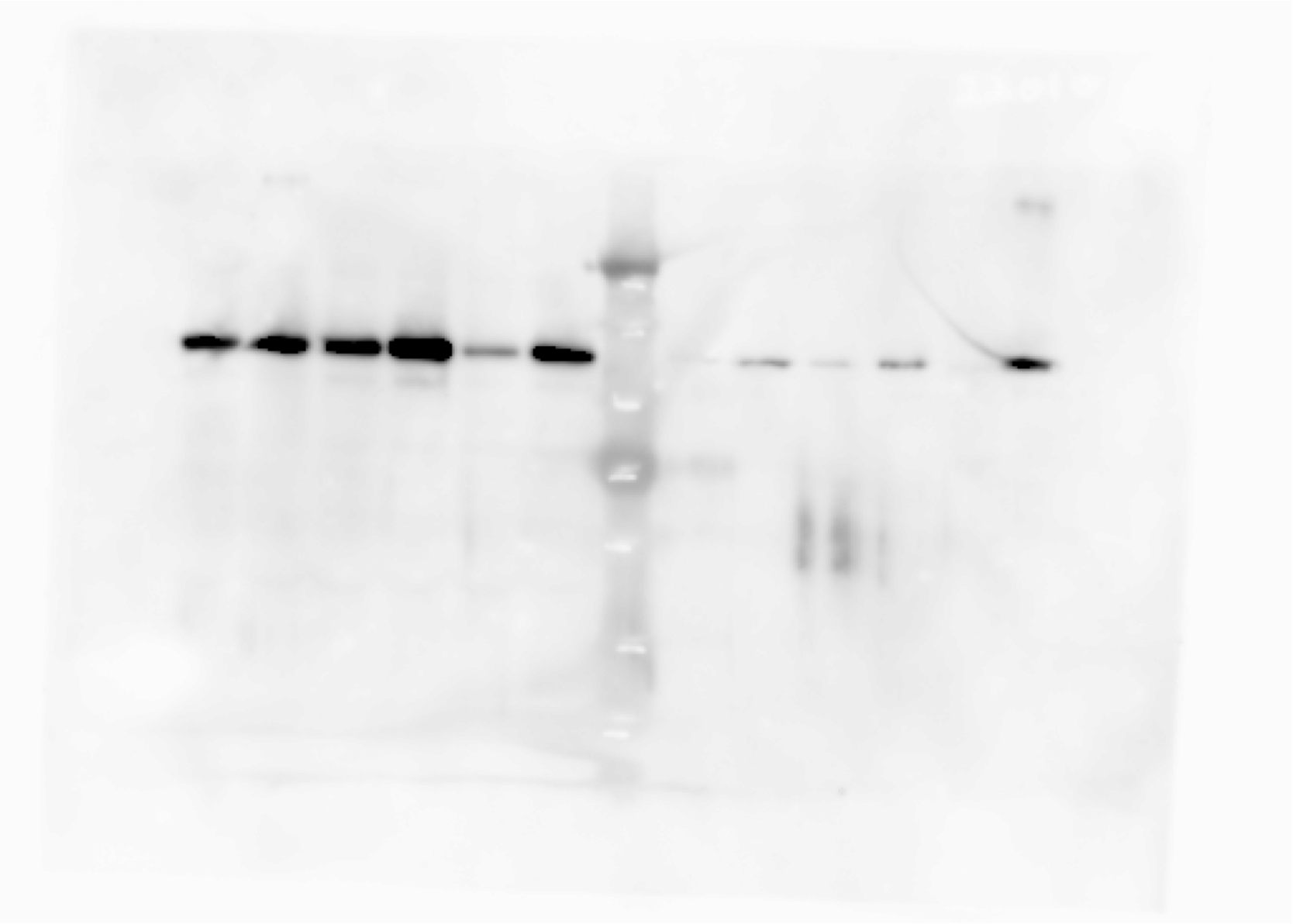

Supplement: Supplementary file 9 — Source Data for Figure 4 [file EMBJ-42-e111484-s003.zip › Figure 4/4C/SDS Western Myc.tif]

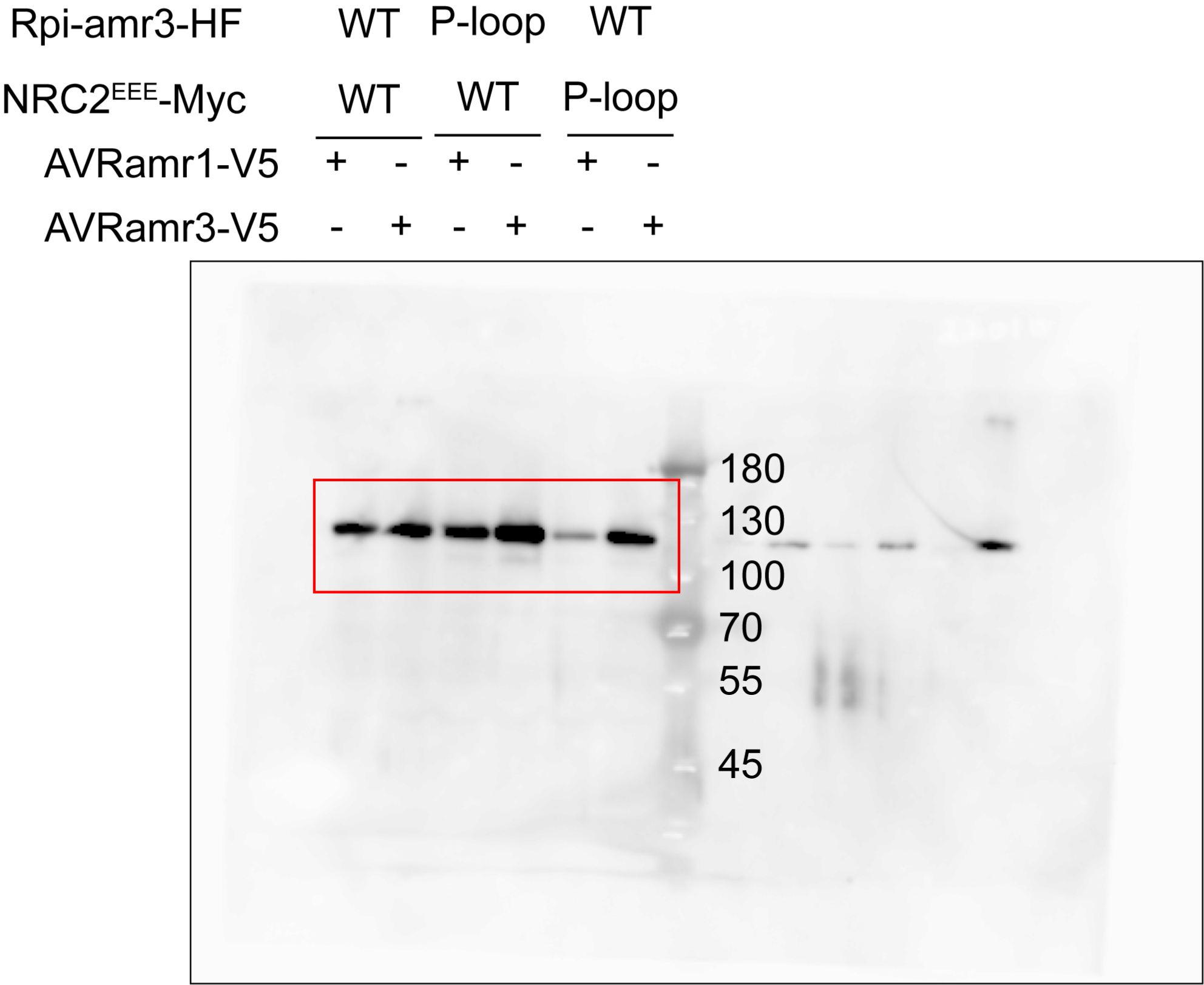

Supplement: Supplementary file 9 — Source Data for Figure 4 [file EMBJ-42-e111484-s003.zip › Figure 4/4C/SDS Western Myc_annotations.tif]

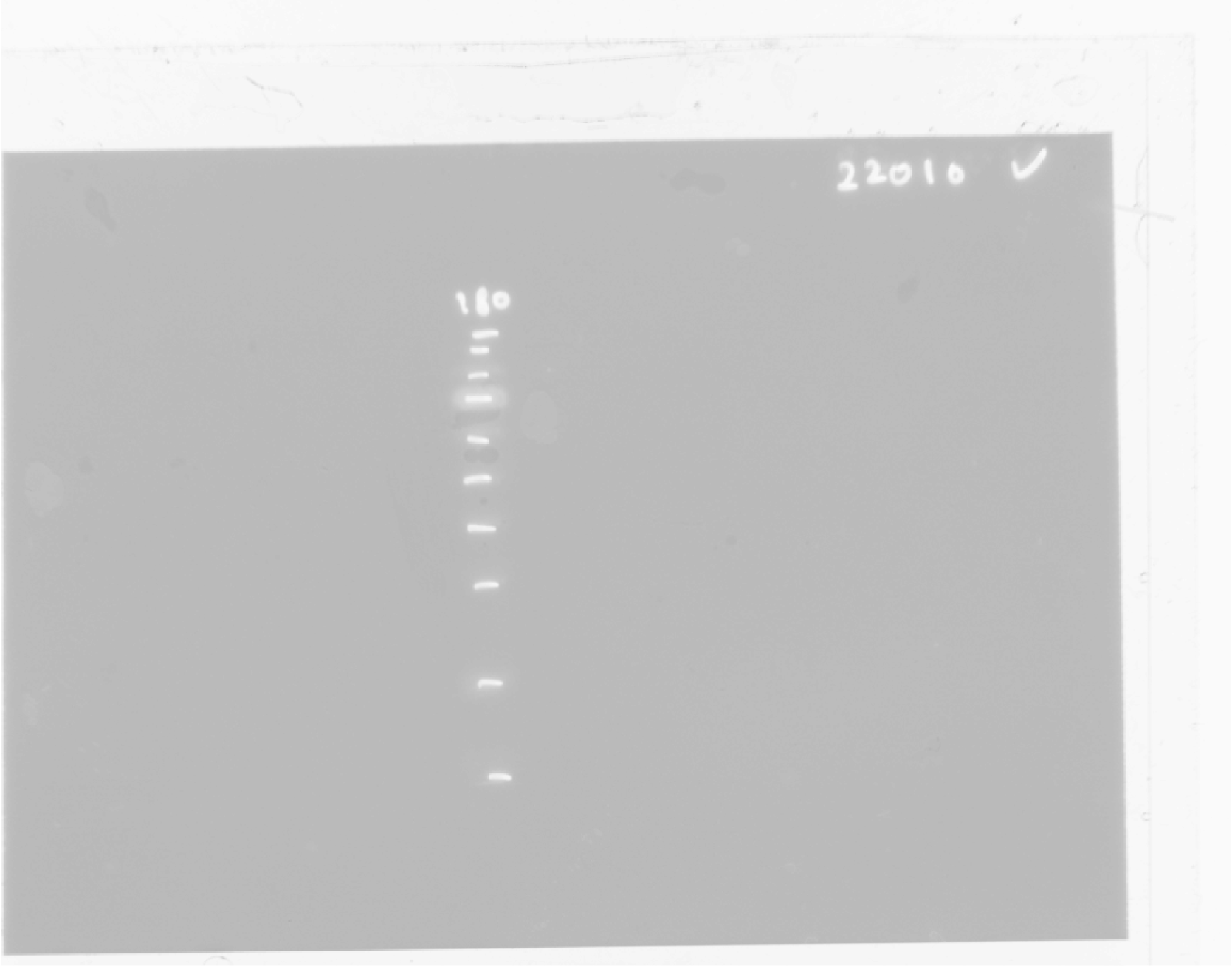

Supplement: Supplementary file 9 — Source Data for Figure 4 [file EMBJ-42-e111484-s003.zip › Figure 4/4C/SDS Western V5 Marker.tif]

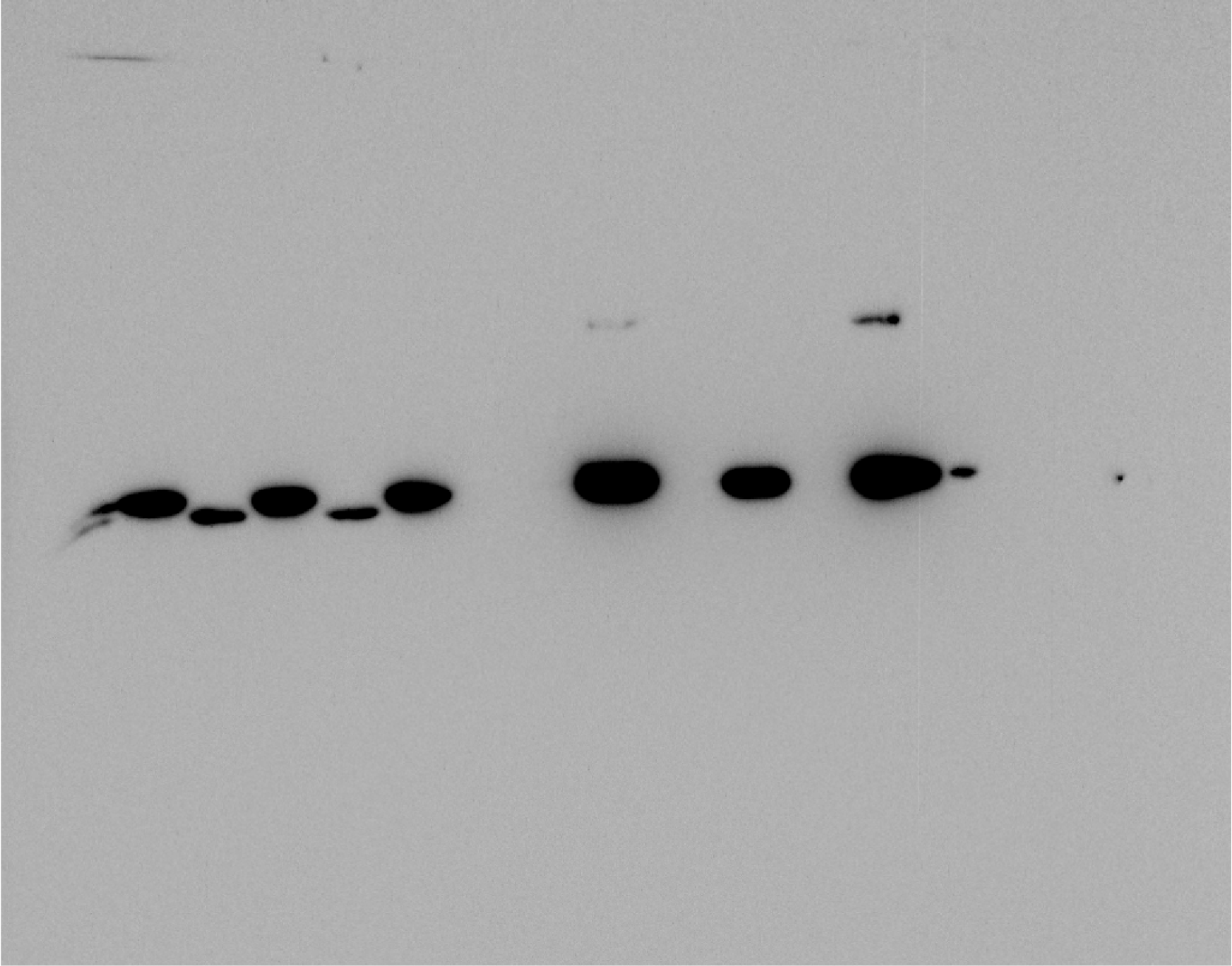

Supplement: Supplementary file 9 — Source Data for Figure 4 [file EMBJ-42-e111484-s003.zip › Figure 4/4C/SDS Western V5.tif]

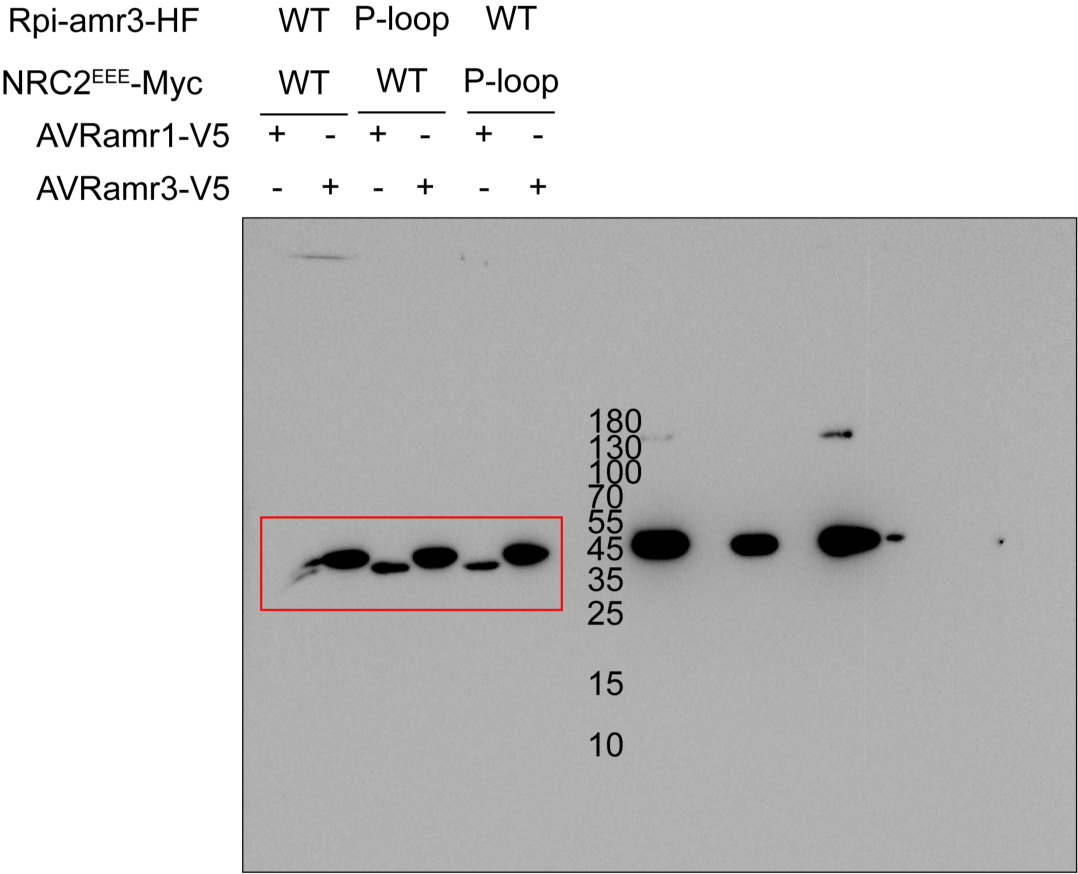

Supplement: Supplementary file 9 — Source Data for Figure 4 [file EMBJ-42-e111484-s003.zip › Figure 4/4C/SDS Western V5_annotations.tif]

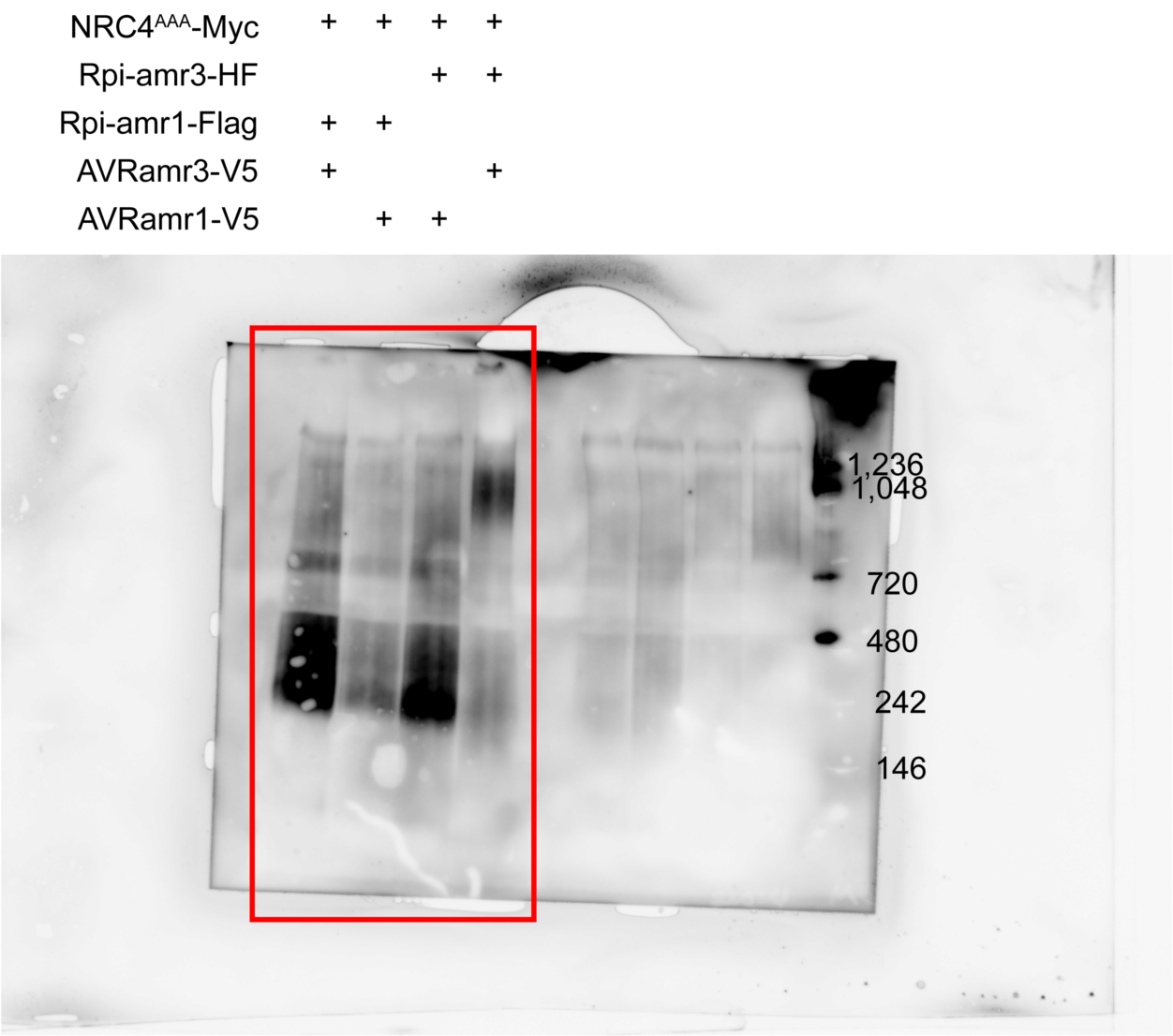

Supplement: Supplementary file 10 — Source Data for Figure 5 [file EMBJ-42-e111484-s009.zip › Figure 5/5B/BNP Western Myc_annotations.tif]

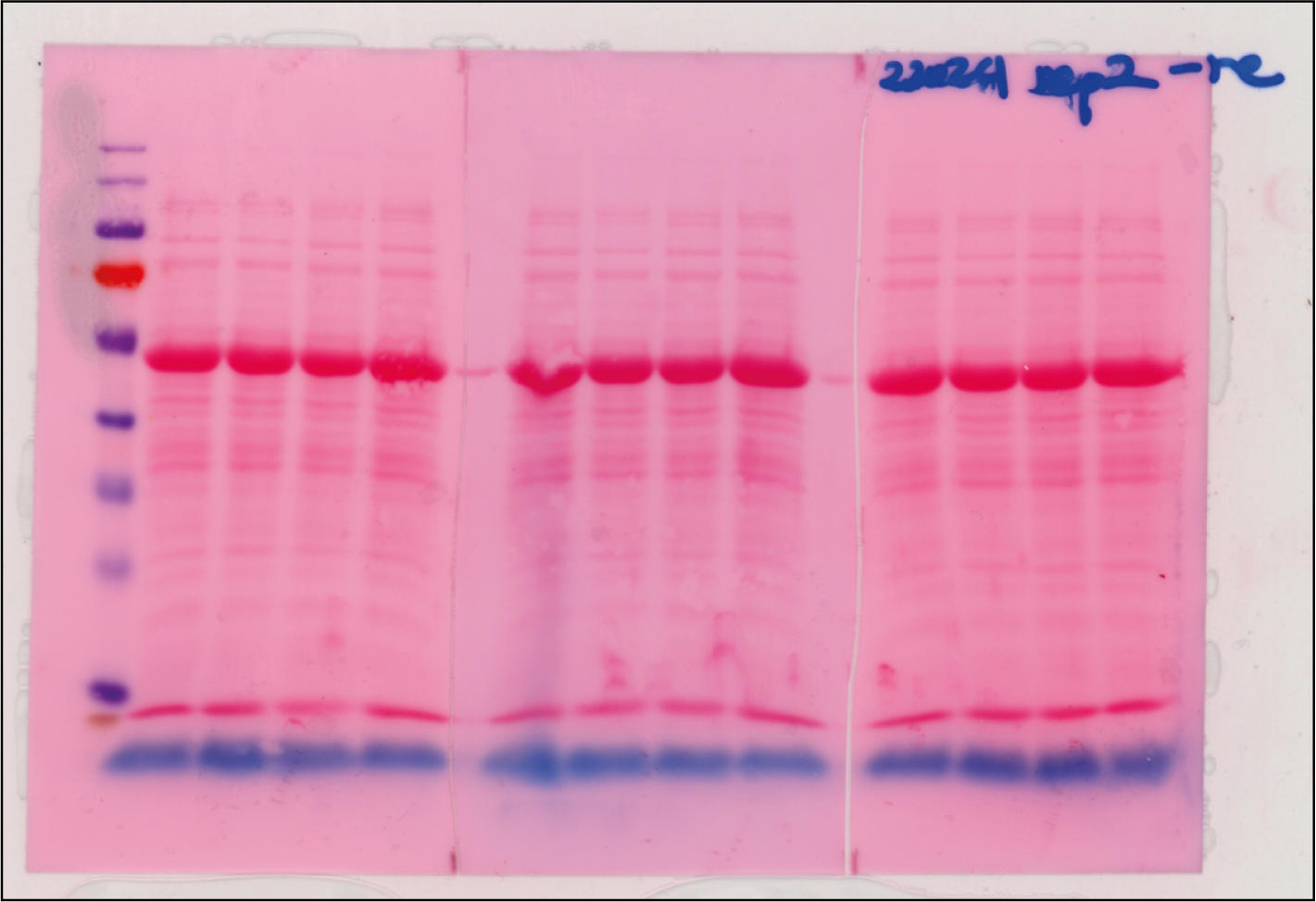

Supplement: Supplementary file 10 — Source Data for Figure 5 [file EMBJ-42-e111484-s009.zip › Figure 5/5B/SDS Ponceau.tif]

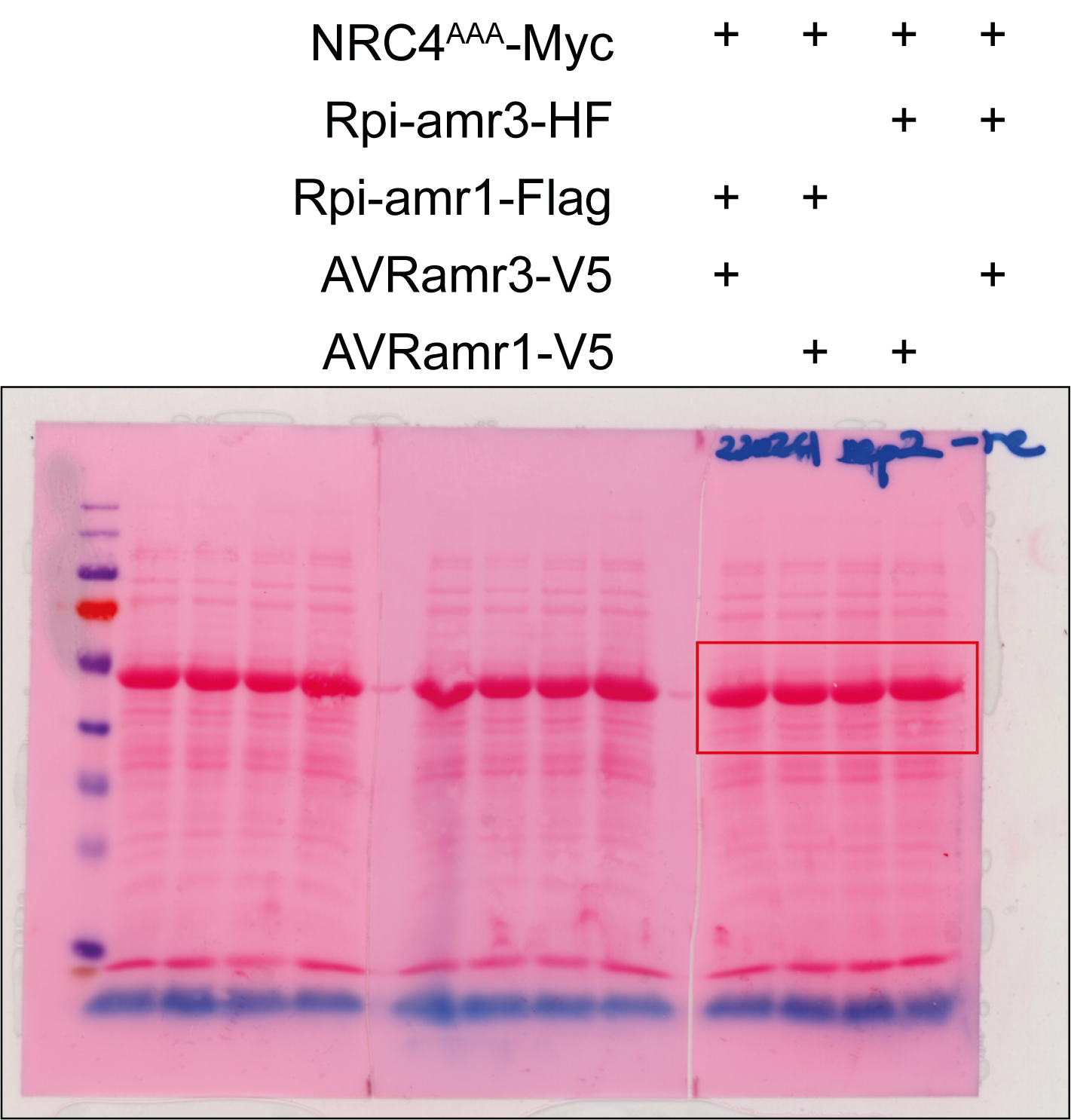

Supplement: Supplementary file 10 — Source Data for Figure 5 [file EMBJ-42-e111484-s009.zip › Figure 5/5B/SDS Ponceau_annotations.tif]

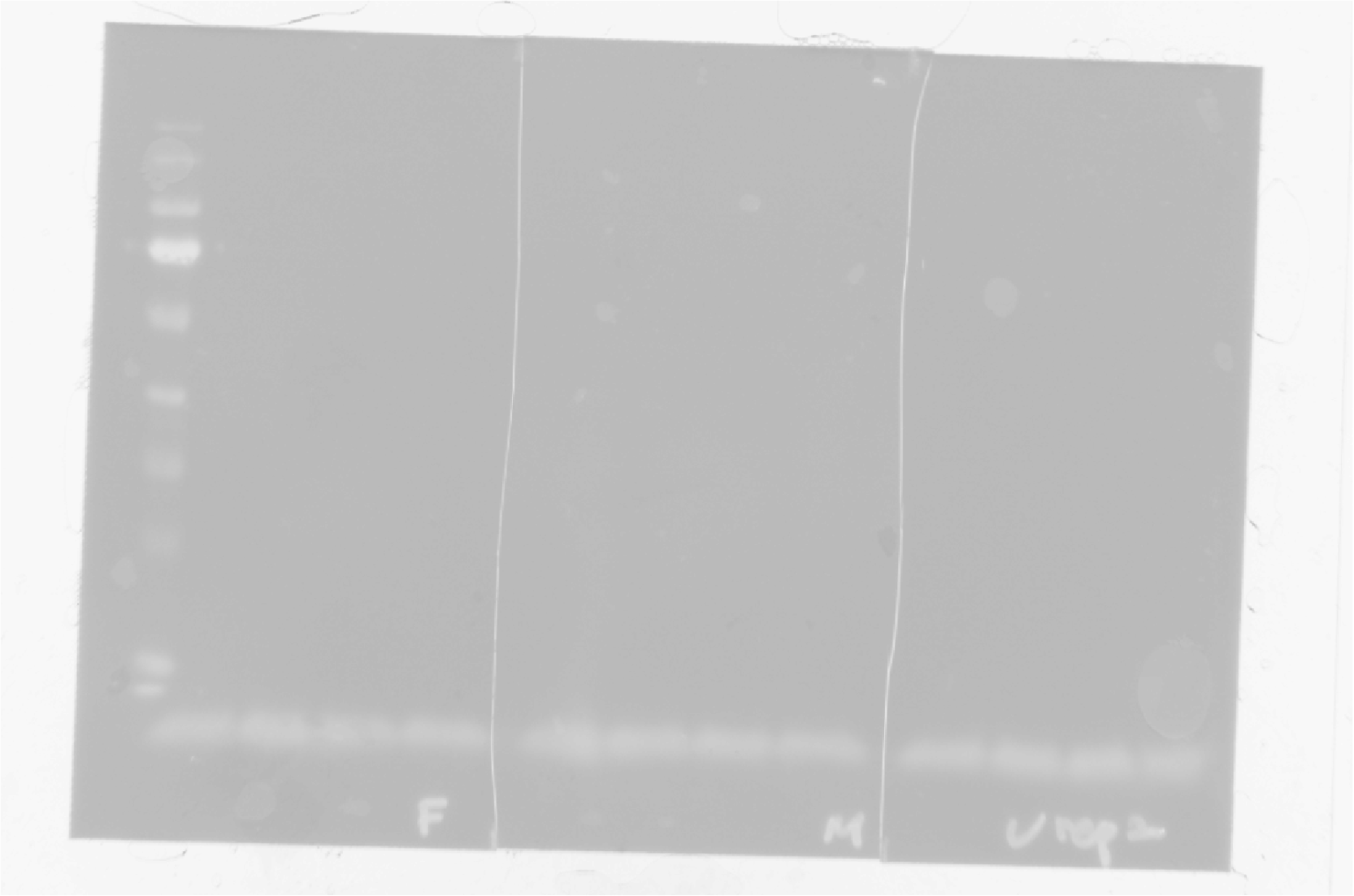

Supplement: Supplementary file 10 — Source Data for Figure 5 [file EMBJ-42-e111484-s009.zip › Figure 5/5B/SDS Western Flag Marker.tif]

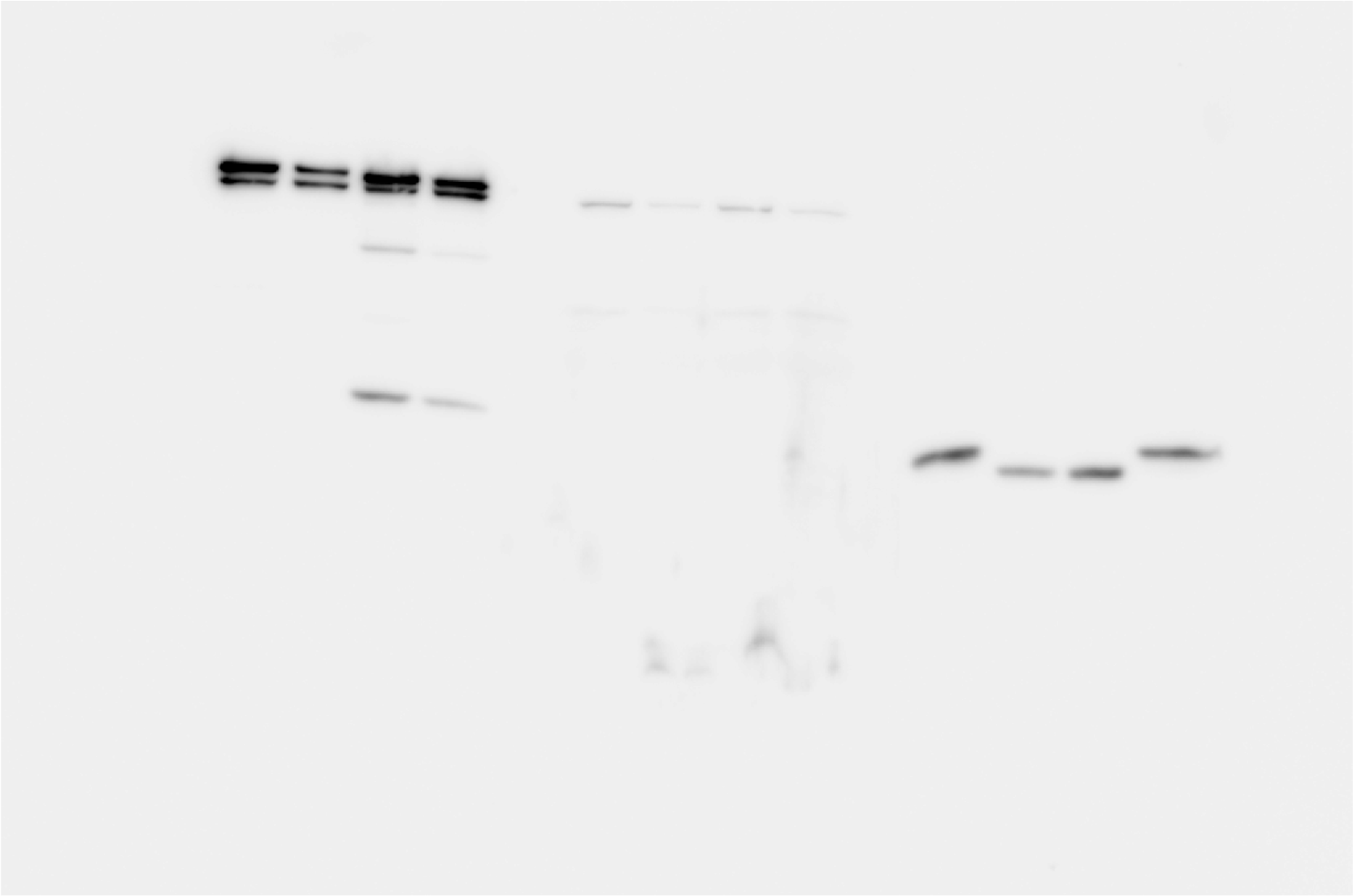

Supplement: Supplementary file 10 — Source Data for Figure 5 [file EMBJ-42-e111484-s009.zip › Figure 5/5B/SDS Western Flag.tif]

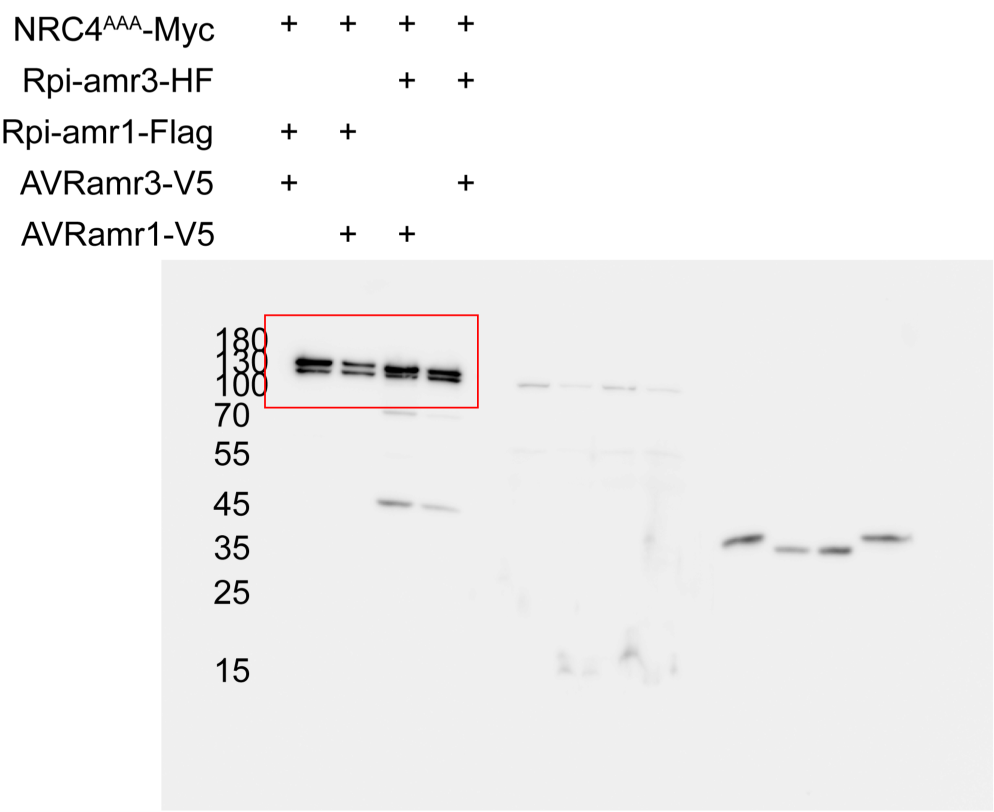

Supplement: Supplementary file 10 — Source Data for Figure 5 [file EMBJ-42-e111484-s009.zip › Figure 5/5B/SDS Western Flag_annotations.tif]

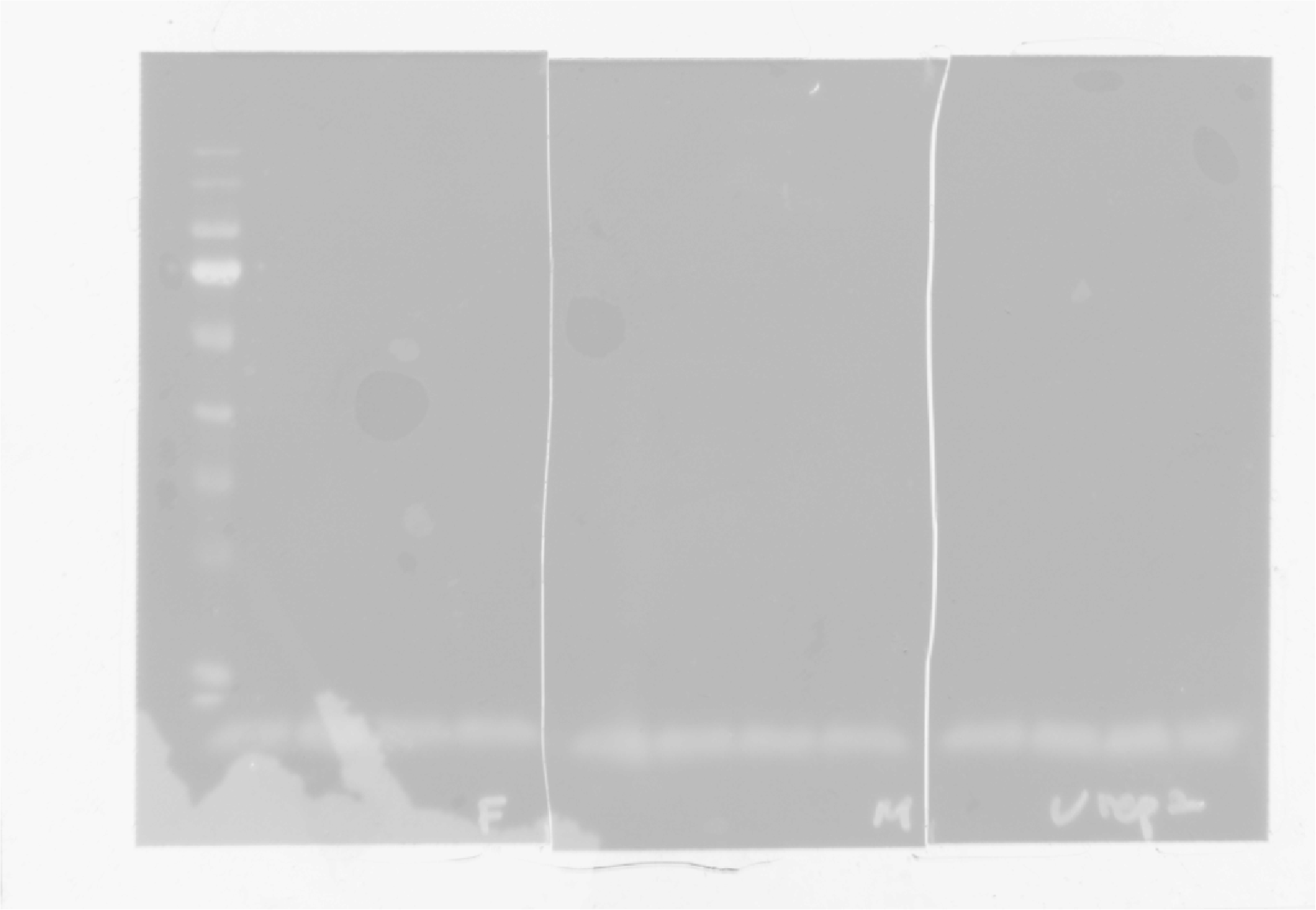

Supplement: Supplementary file 10 — Source Data for Figure 5 [file EMBJ-42-e111484-s009.zip › Figure 5/5B/SDS Western Myc, V5 Marker.tif]

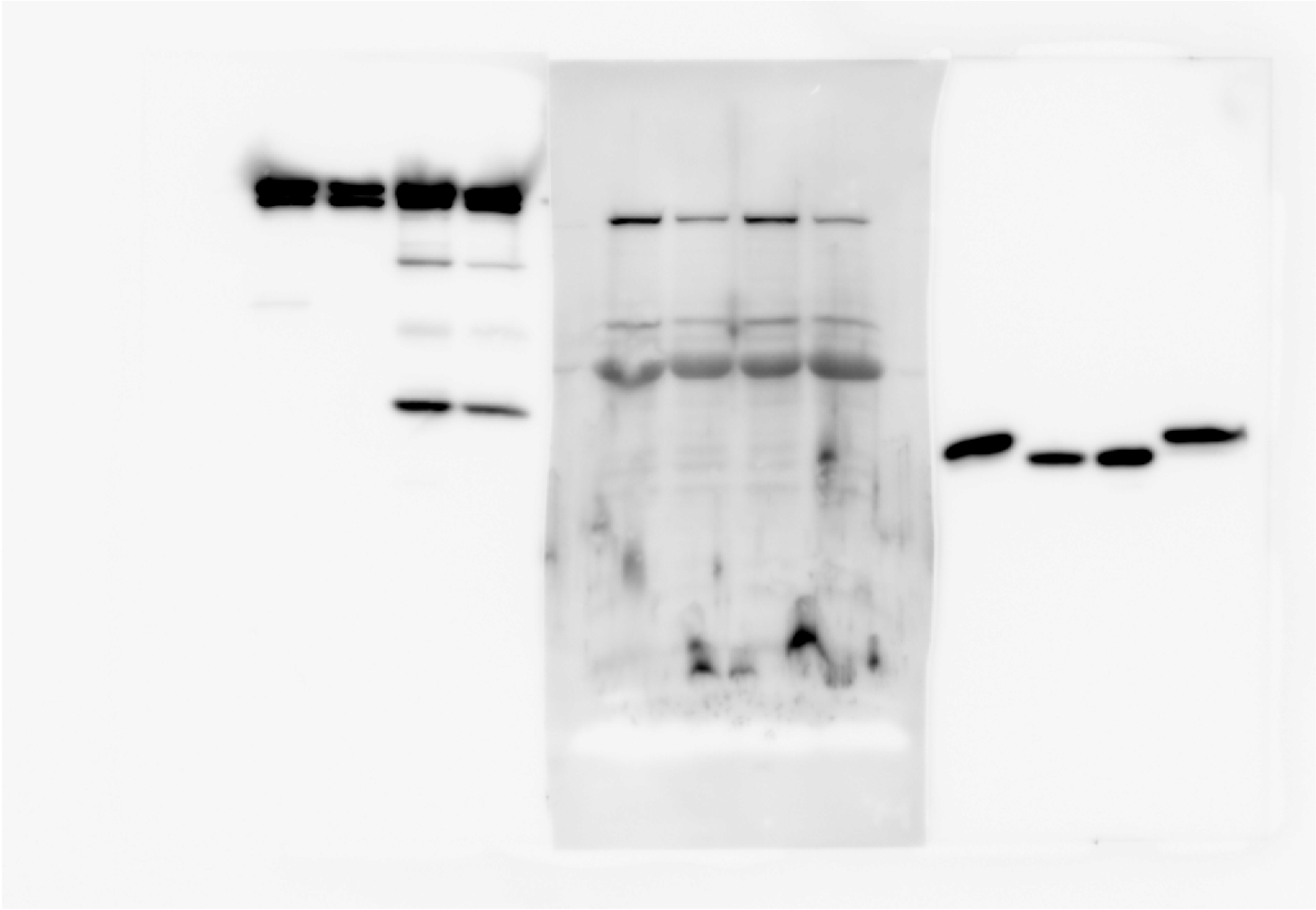

Supplement: Supplementary file 10 — Source Data for Figure 5 [file EMBJ-42-e111484-s009.zip › Figure 5/5B/SDS Western Myc, V5.tif]

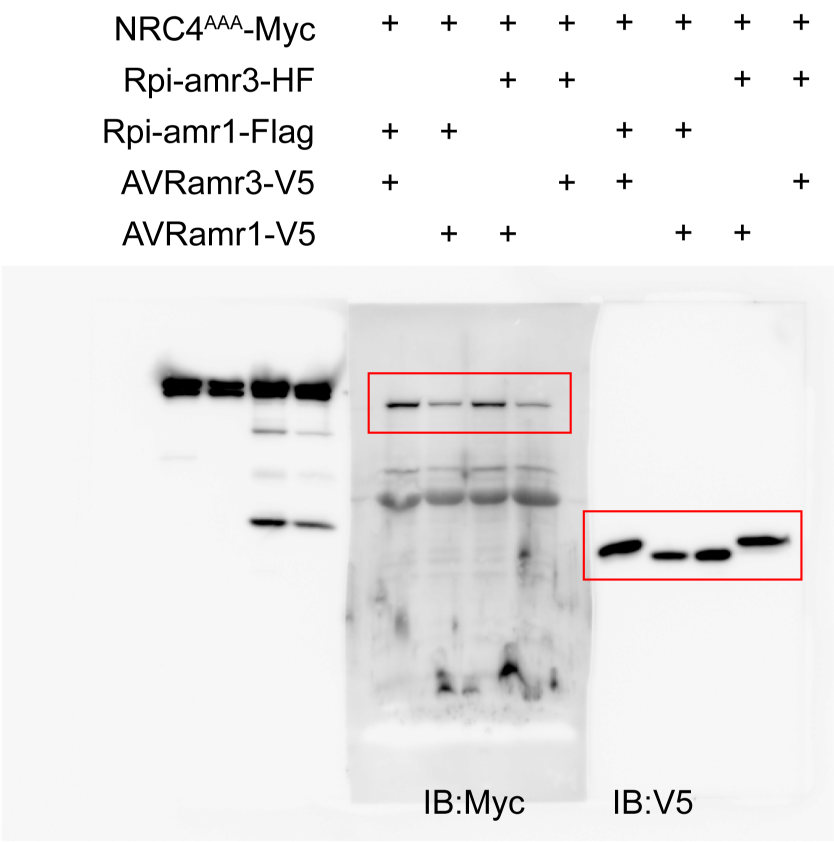

Supplement: Supplementary file 10 — Source Data for Figure 5 [file EMBJ-42-e111484-s009.zip › Figure 5/5B/SDS Western Myc, V5_annotations.tif]

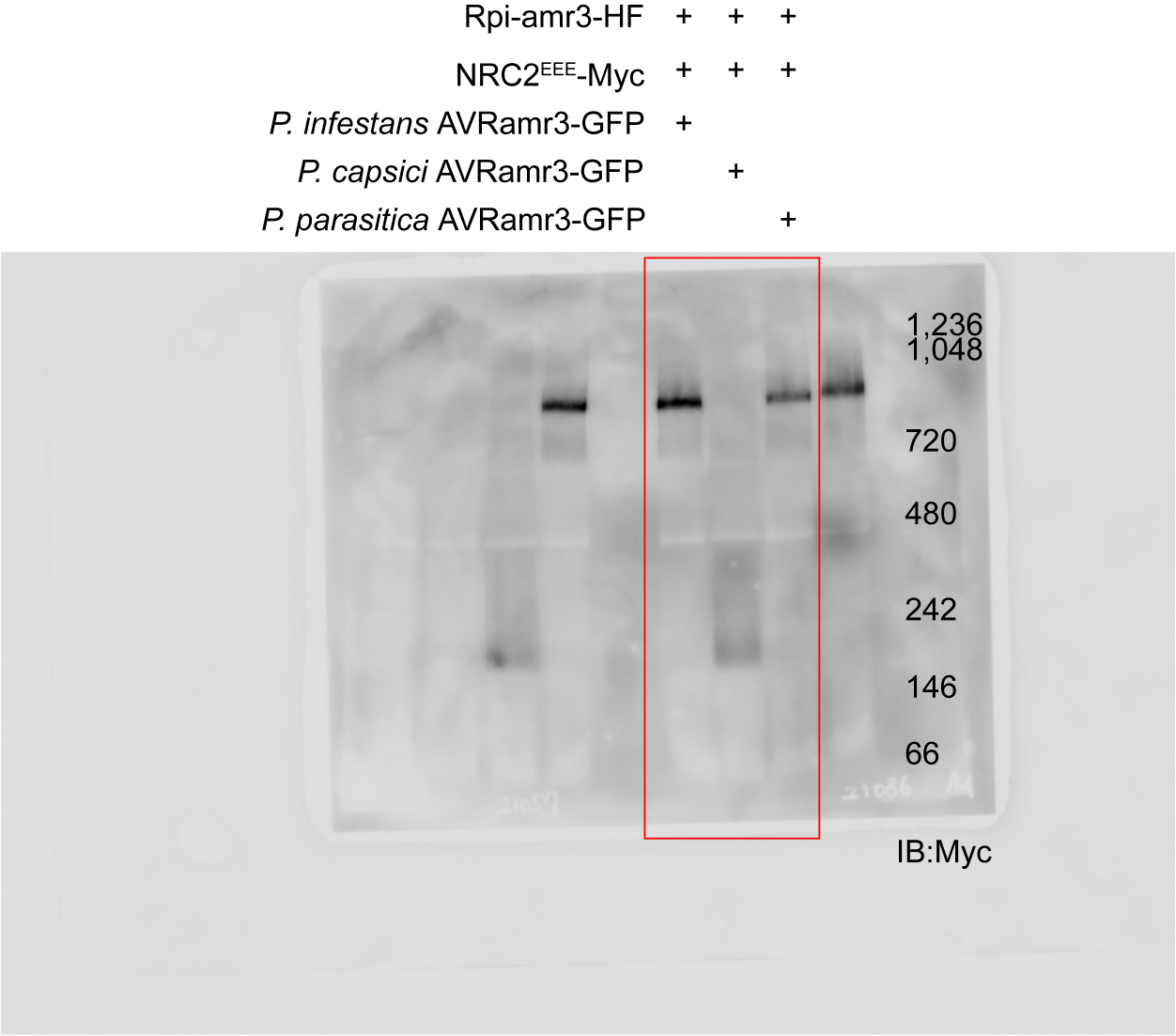

Supplement: Supplementary file 11 — Source Data for Figure 6 [file EMBJ-42-e111484-s004.zip › Figure 6/6B/BNP Western Myc_annotations.tif]

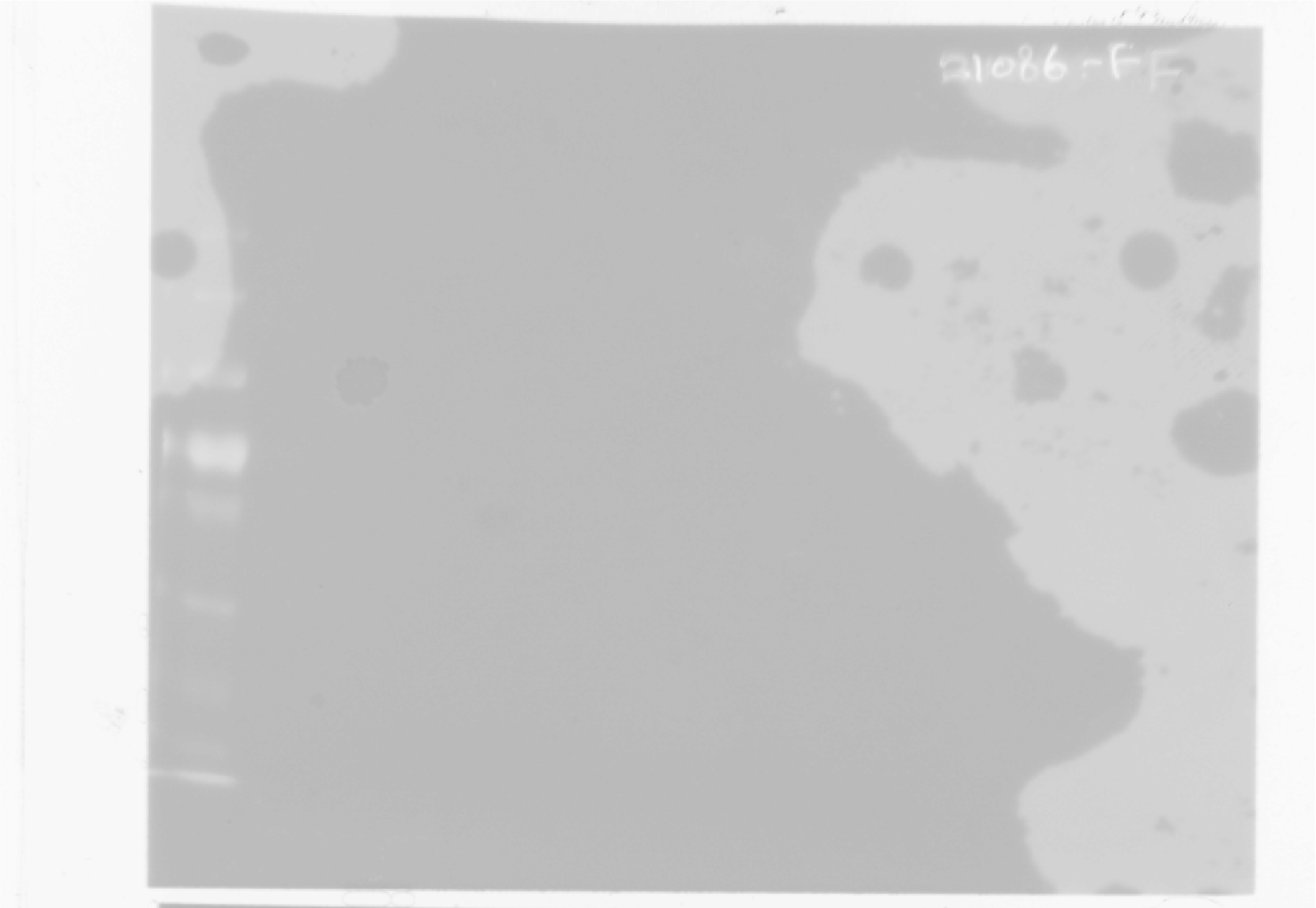

Supplement: Supplementary file 11 — Source Data for Figure 6 [file EMBJ-42-e111484-s004.zip › Figure 6/6B/SDS Western Flag Marker.tif]

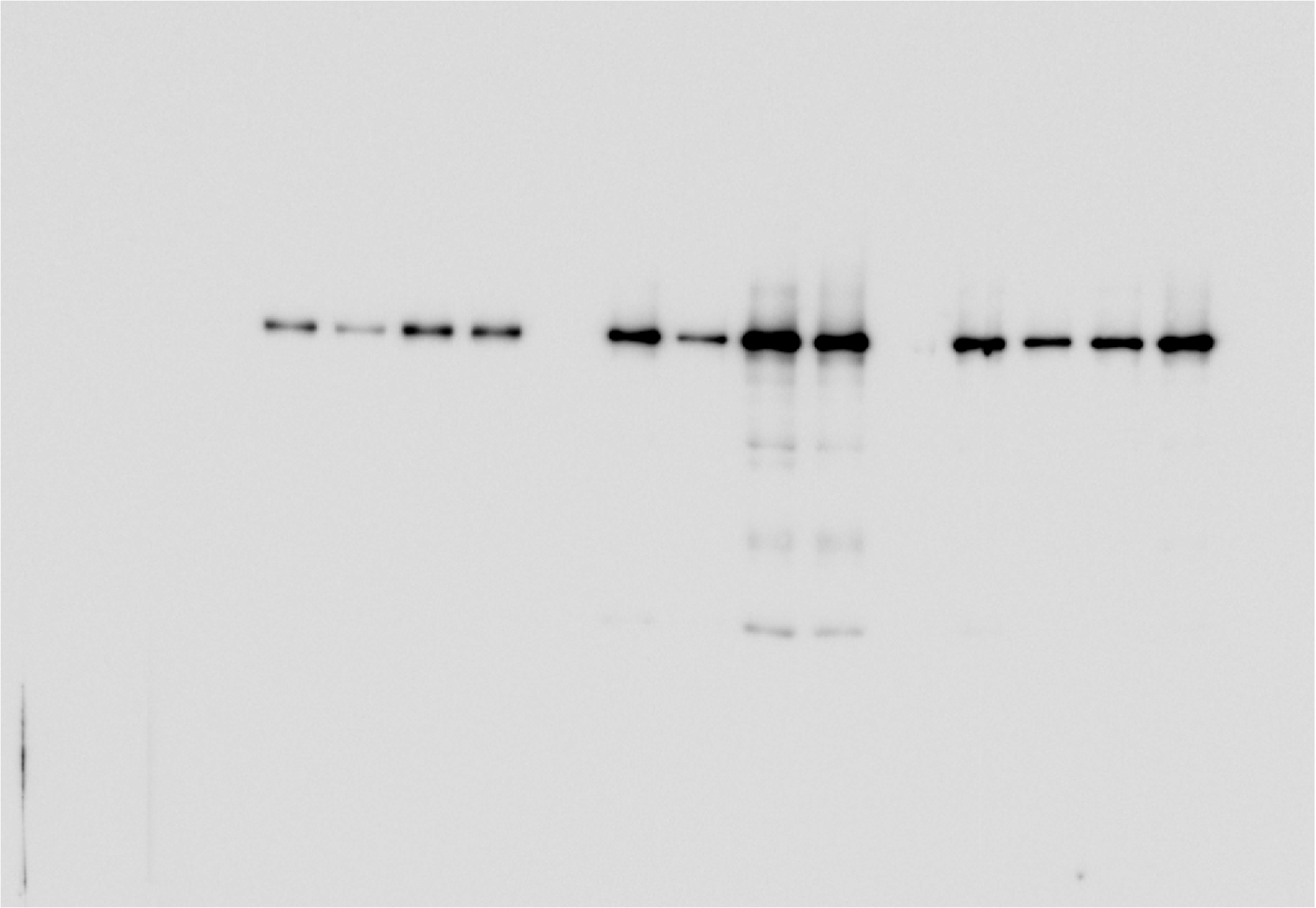

Supplement: Supplementary file 11 — Source Data for Figure 6 [file EMBJ-42-e111484-s004.zip › Figure 6/6B/SDS Western Flag.tif]

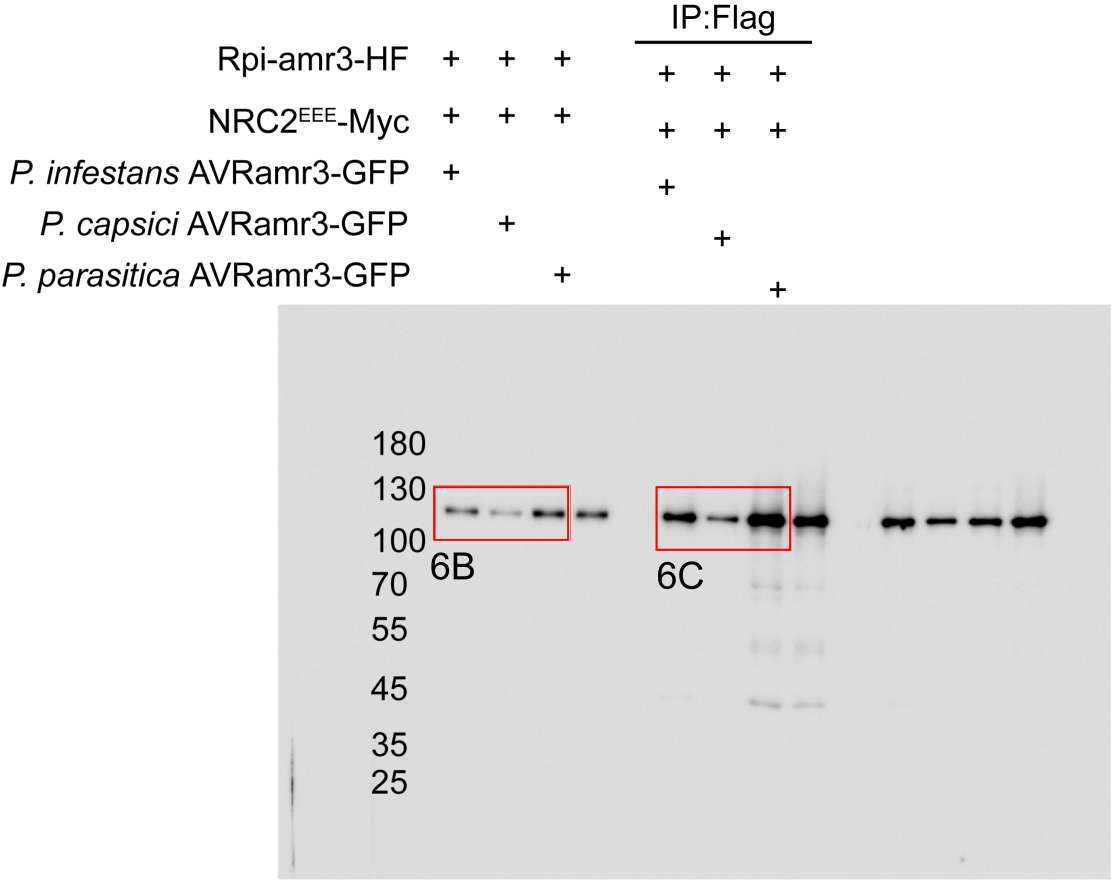

Supplement: Supplementary file 11 — Source Data for Figure 6 [file EMBJ-42-e111484-s004.zip › Figure 6/6B/SDS Western Flag_annotations.tif]

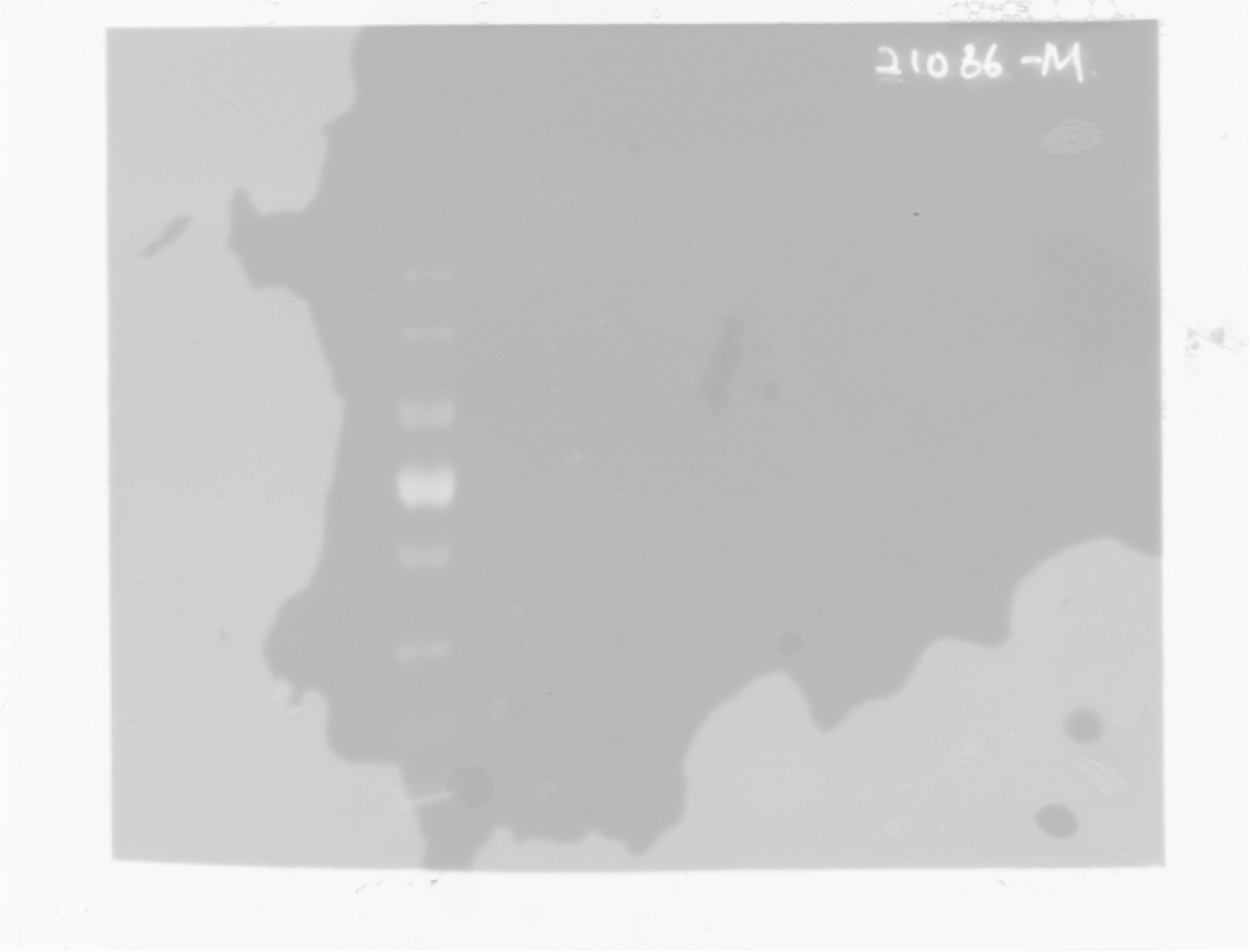

Supplement: Supplementary file 11 — Source Data for Figure 6 [file EMBJ-42-e111484-s004.zip › Figure 6/6B/SDS Western Myc Marker.tif]

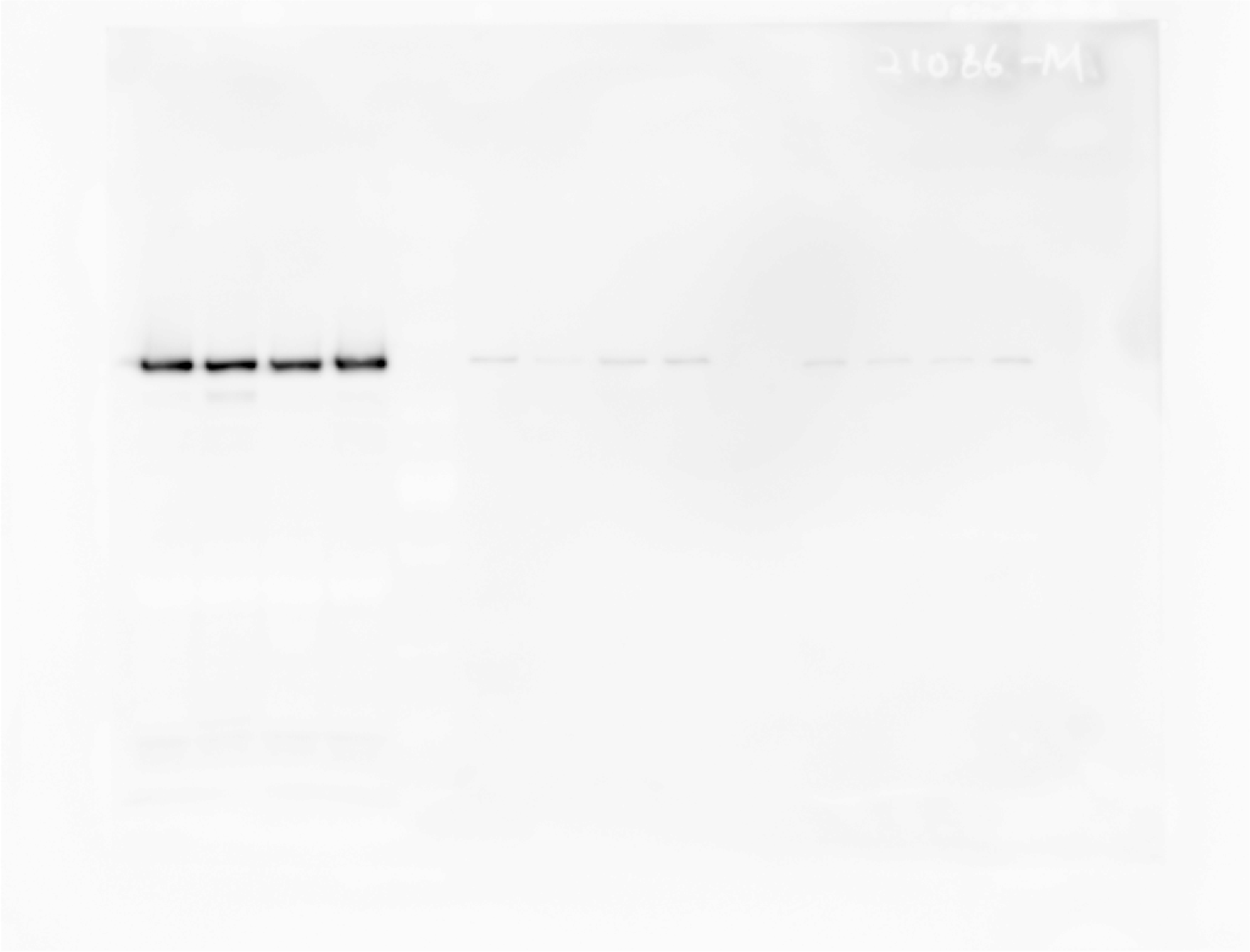

Supplement: Supplementary file 11 — Source Data for Figure 6 [file EMBJ-42-e111484-s004.zip › Figure 6/6B/SDS Western Myc.tif]

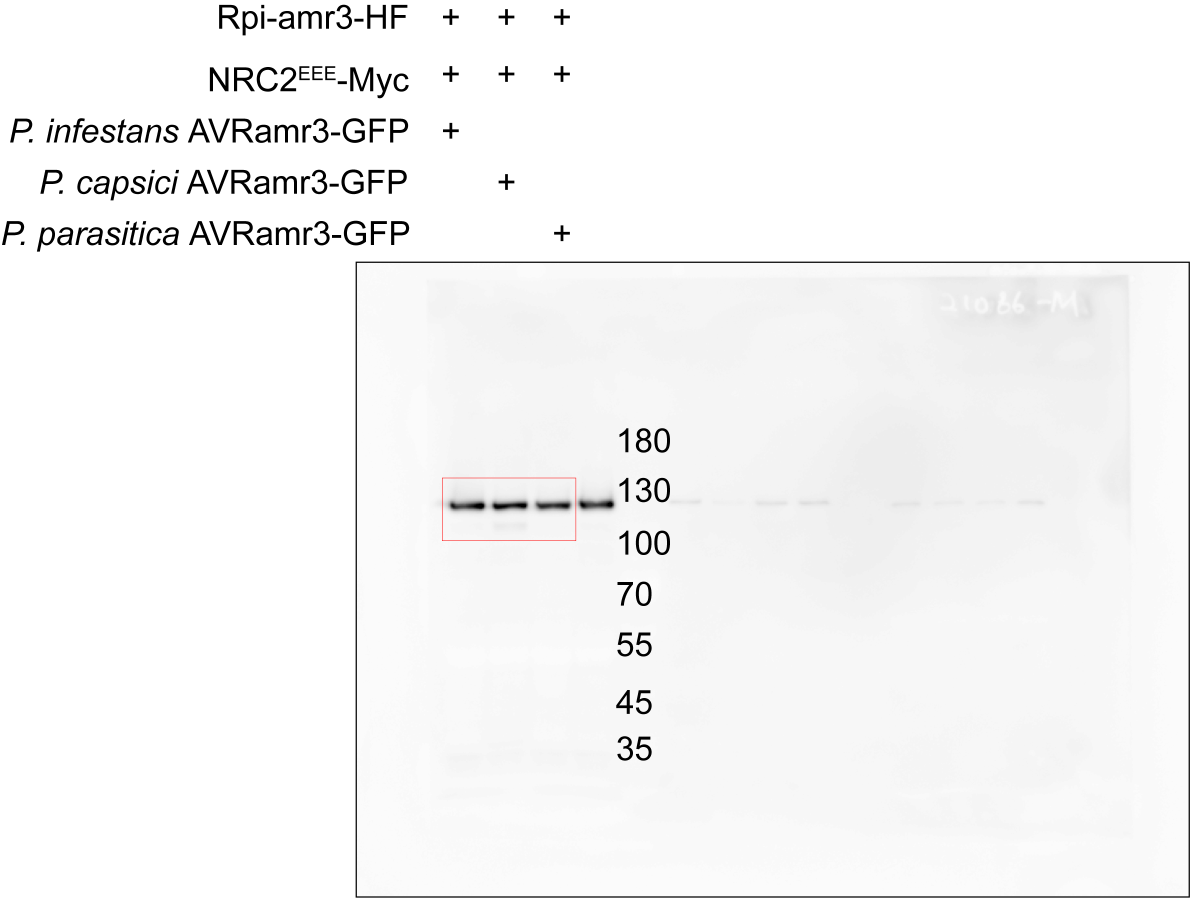

Supplement: Supplementary file 11 — Source Data for Figure 6 [file EMBJ-42-e111484-s004.zip › Figure 6/6B/SDS Western Myc_annotations.tif]

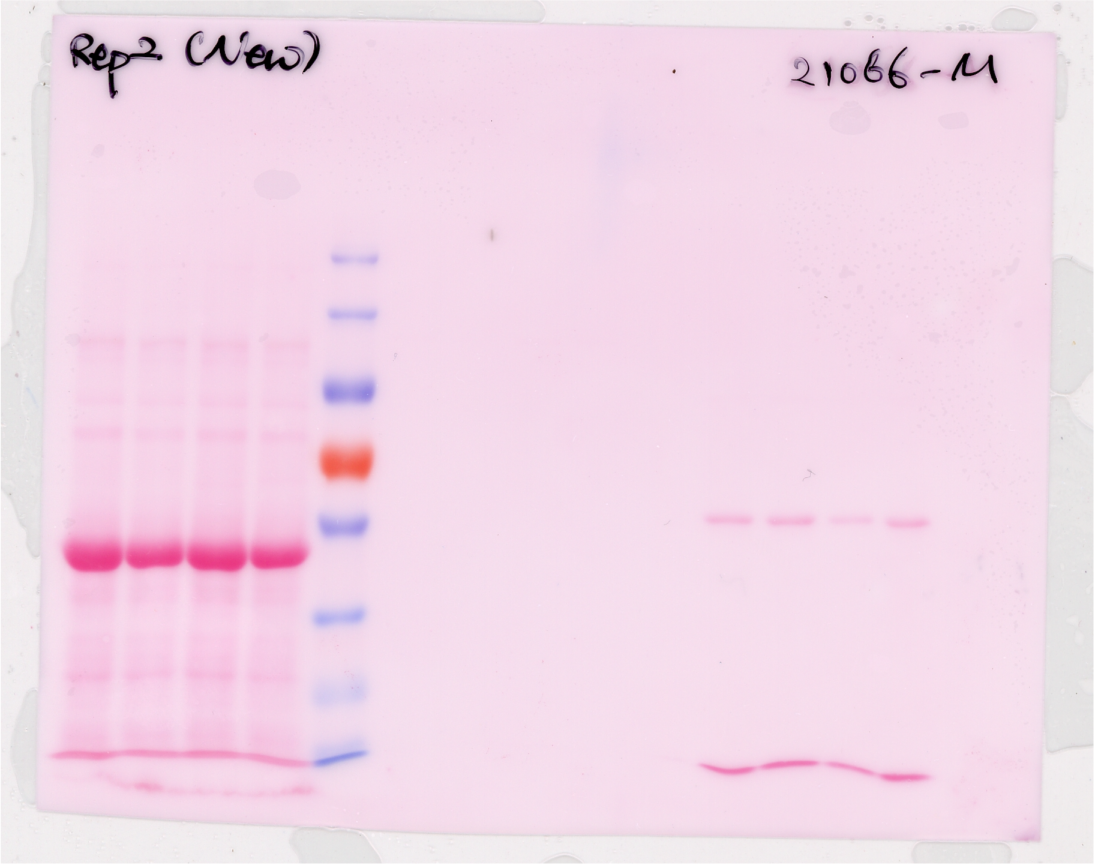

Supplement: Supplementary file 11 — Source Data for Figure 6 [file EMBJ-42-e111484-s004.zip › Figure 6/6B/SDS Western Ponceau.tif]

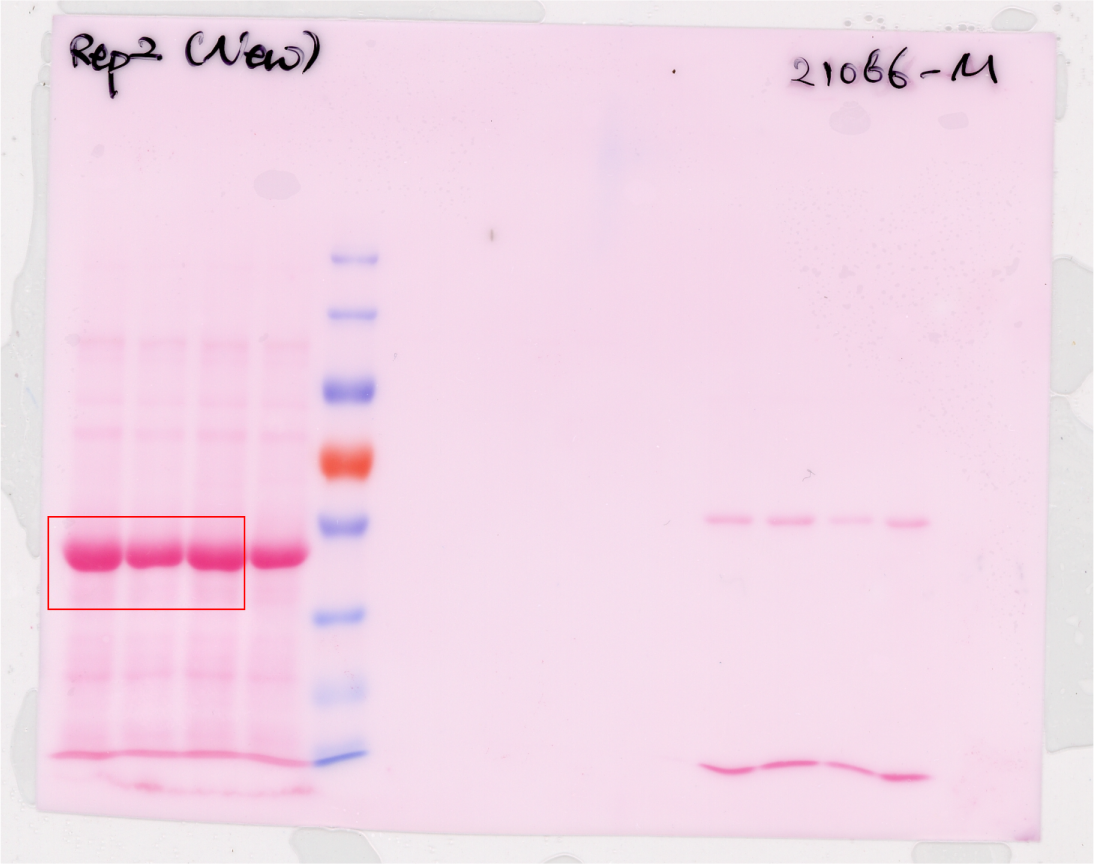

Supplement: Supplementary file 11 — Source Data for Figure 6 [file EMBJ-42-e111484-s004.zip › Figure 6/6B/SDS Western Ponceau_annotations.tif]

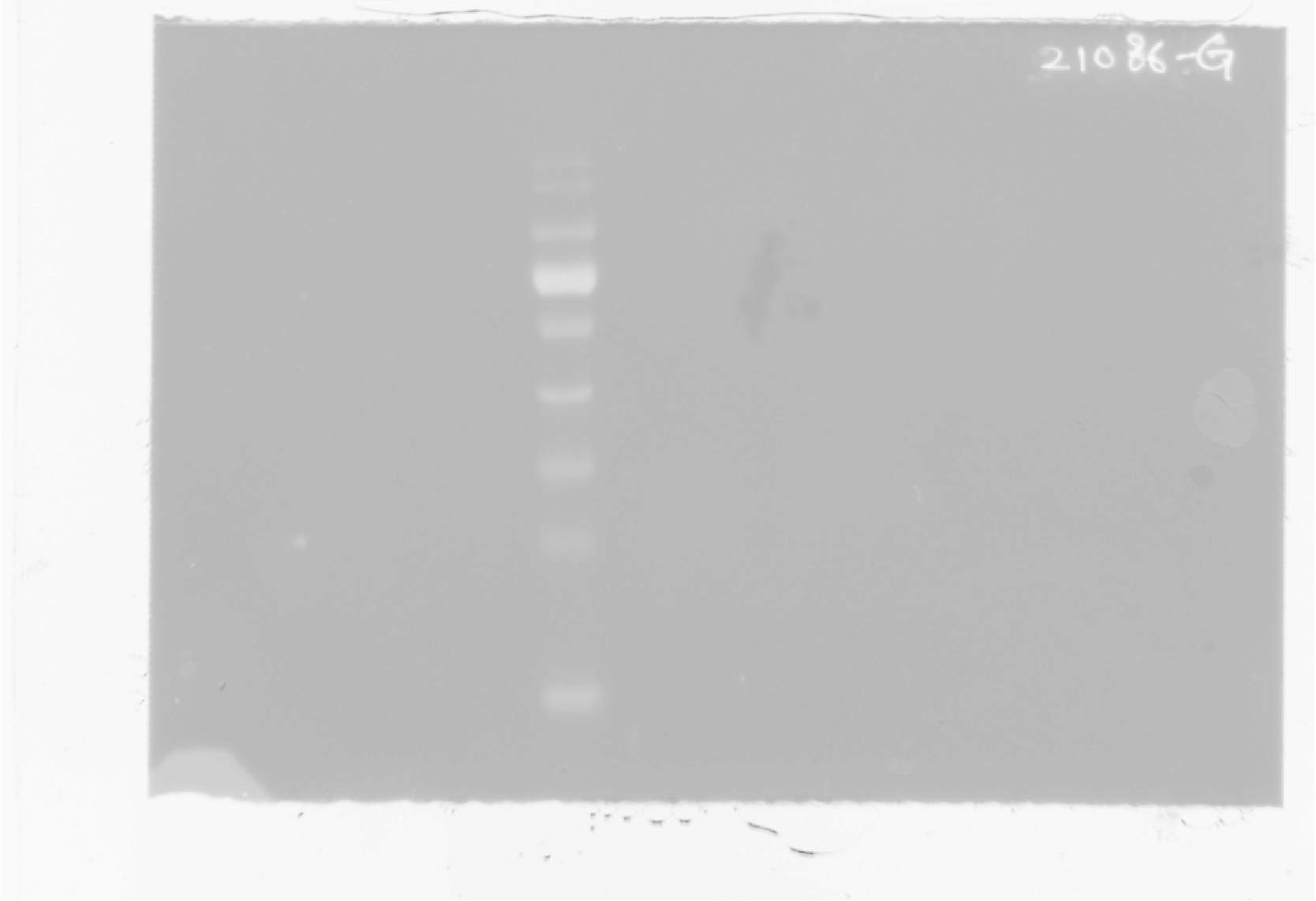

Supplement: Supplementary file 11 — Source Data for Figure 6 [file EMBJ-42-e111484-s004.zip › Figure 6/6B/SDS Western V5 Marker.tif]

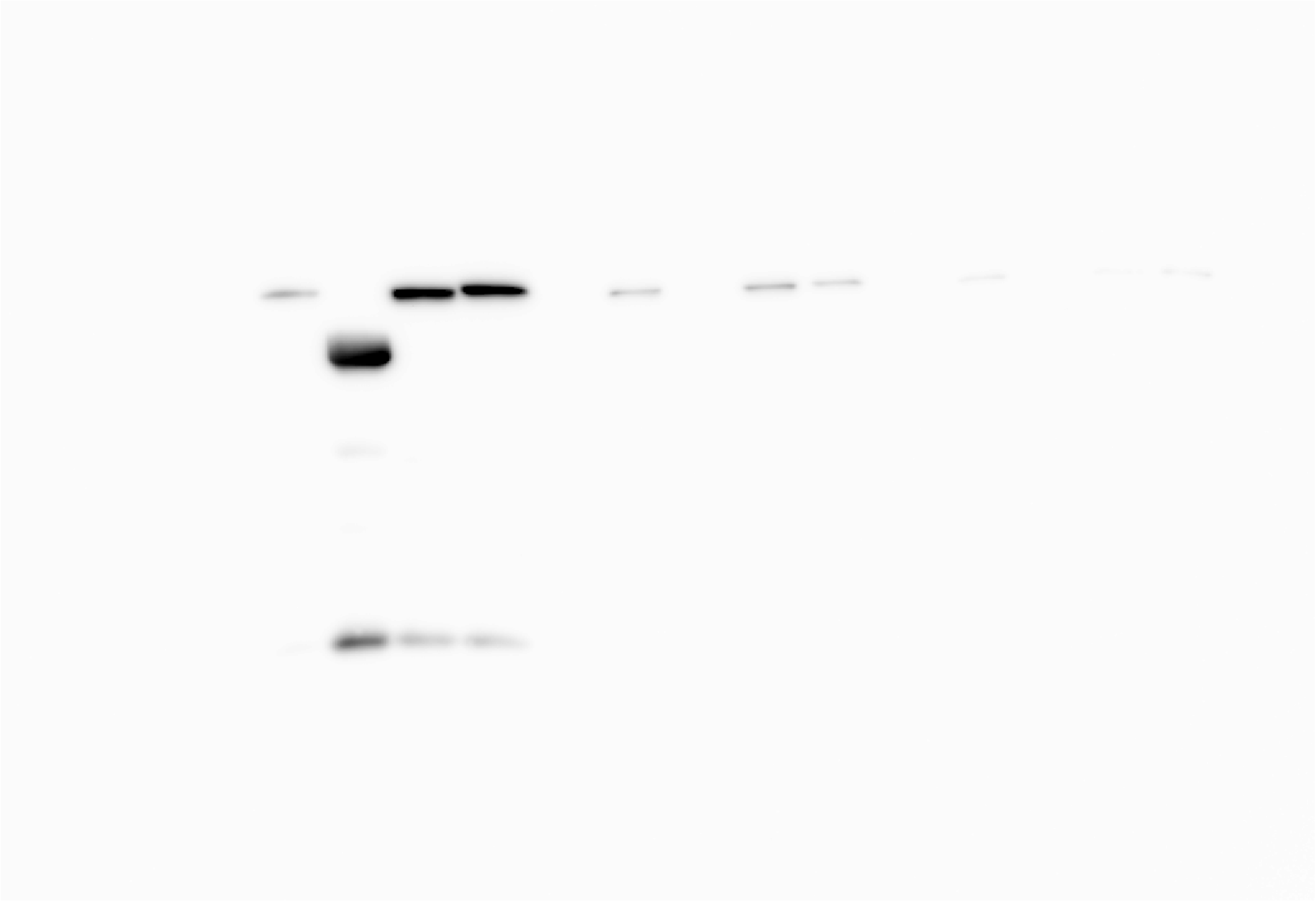

Supplement: Supplementary file 11 — Source Data for Figure 6 [file EMBJ-42-e111484-s004.zip › Figure 6/6B/SDS Western V5.tif]

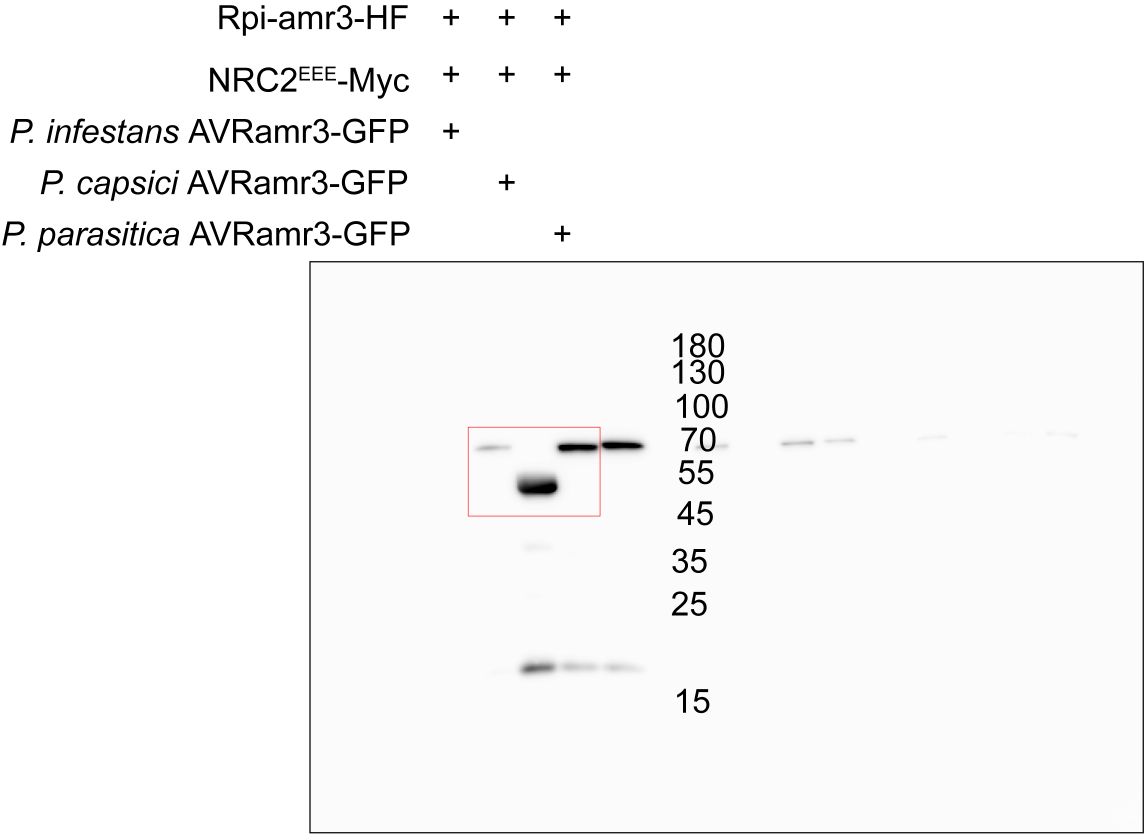

Supplement: Supplementary file 11 — Source Data for Figure 6 [file EMBJ-42-e111484-s004.zip › Figure 6/6B/SDS Western V5_annotations.tif]

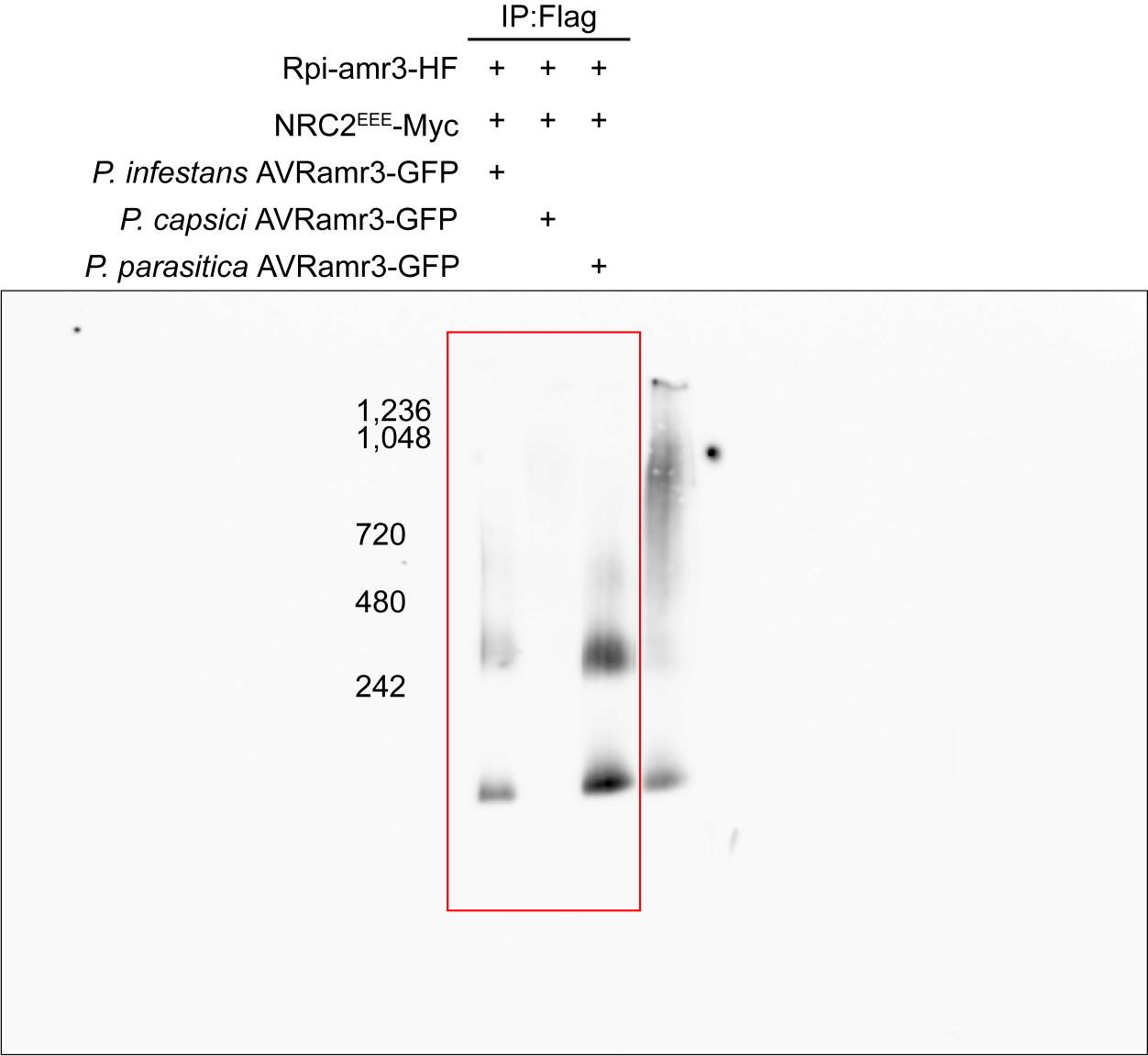

Supplement: Supplementary file 11 — Source Data for Figure 6 [file EMBJ-42-e111484-s004.zip › Figure 6/6C/BNP Western GFP_annotations.tif]

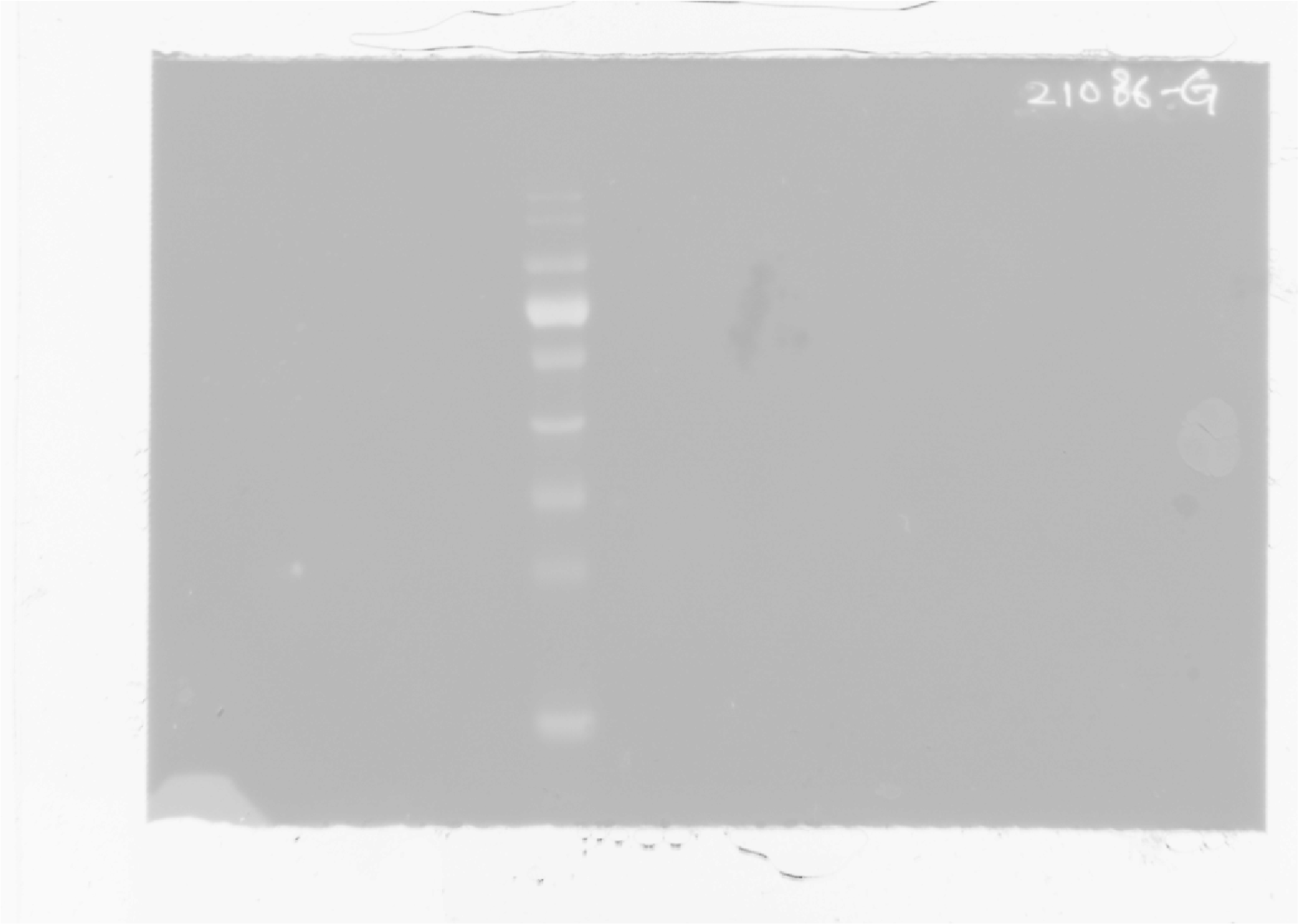

Supplement: Supplementary file 11 — Source Data for Figure 6 [file EMBJ-42-e111484-s004.zip › Figure 6/6C/SDS Western GFP Marker.tif]

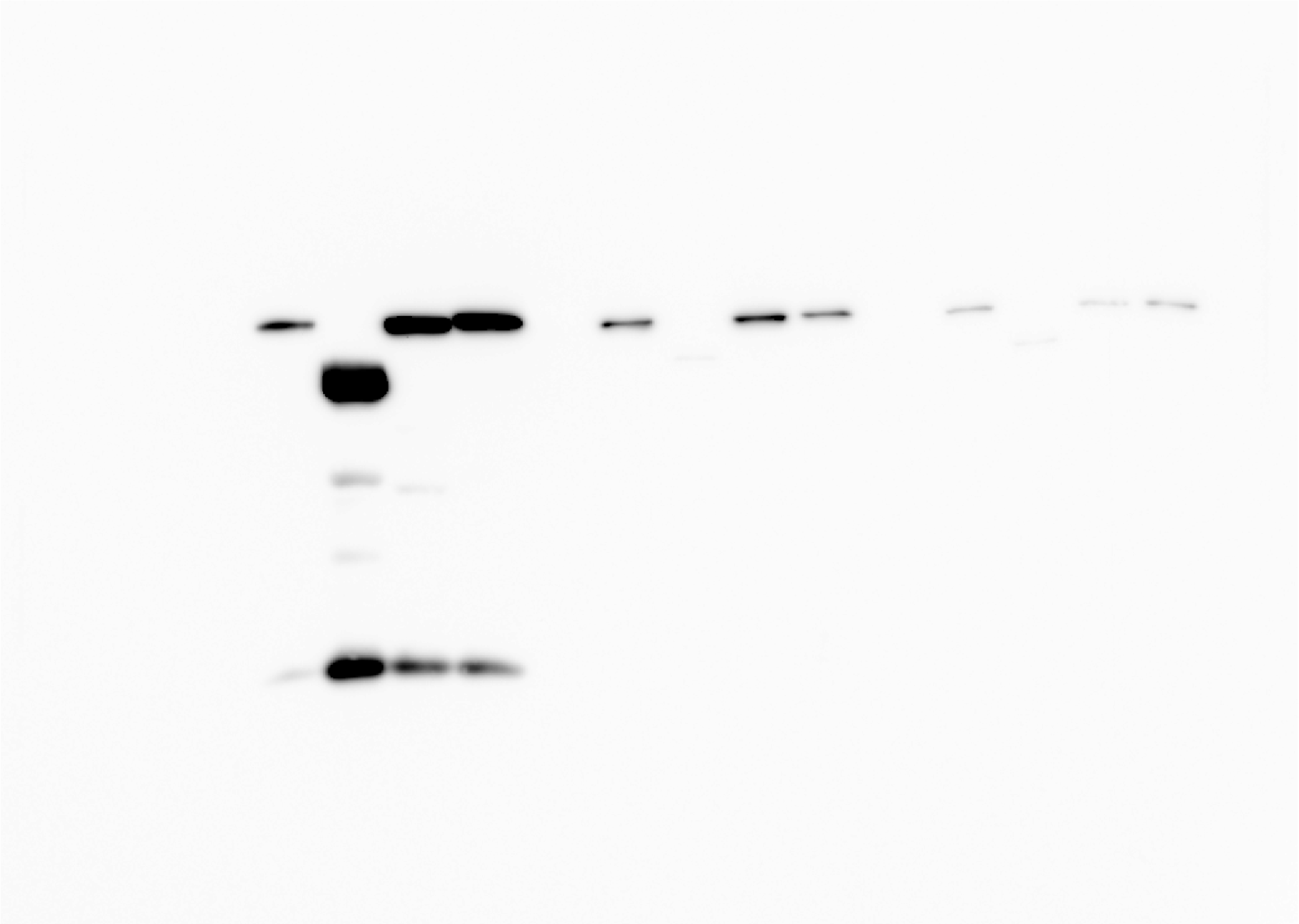

Supplement: Supplementary file 11 — Source Data for Figure 6 [file EMBJ-42-e111484-s004.zip › Figure 6/6C/SDS Western GFP.tif]

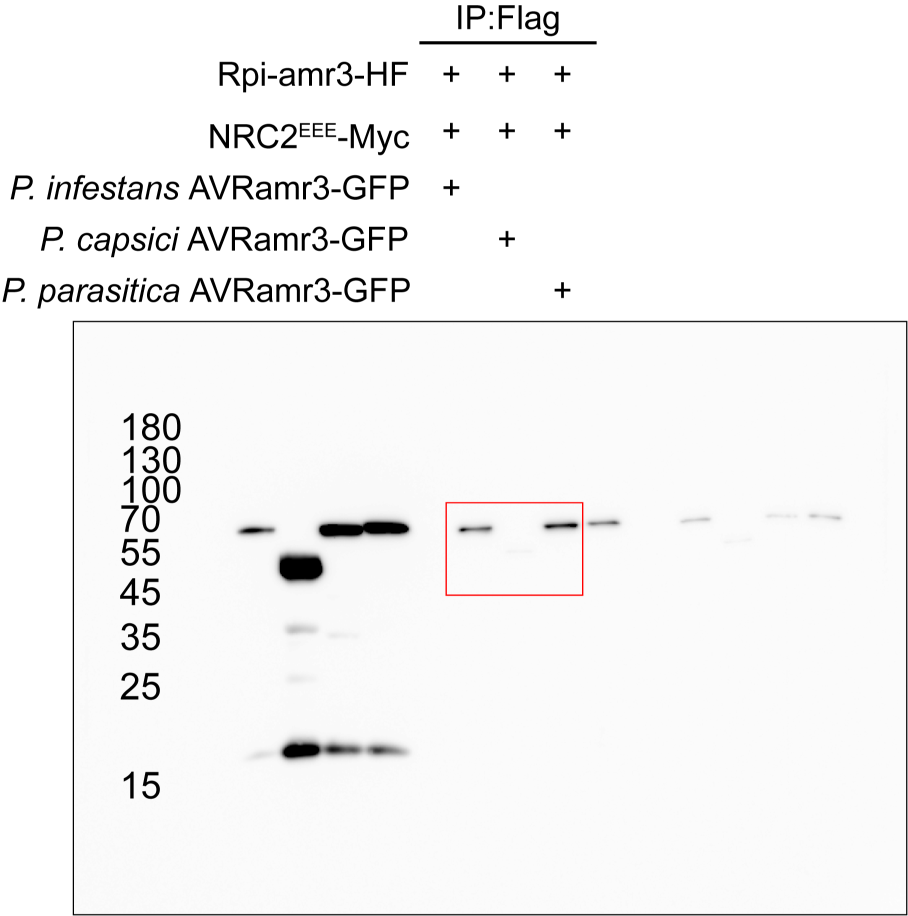

Supplement: Supplementary file 11 — Source Data for Figure 6 [file EMBJ-42-e111484-s004.zip › Figure 6/6C/SDS Western GFP_annotations.tif]
