# Supplementary material for: Effector‐dependent activation and oligomerization of plant NRC class helper NLRs by sensor NLR immune receptors Rpi‐amr3 and Rpi‐amr1
Source: EMBO J. 2023 Jan 2;42(5):e111484. doi: 10.15252/embj.2022111484 (PMC9975942; doi:10.15252/embj.2022111484)
Supplement: Supplementary file 12 [file EMBJ-42-e111484-s001.pdf]

THE EMBO JOURNAL

Effector-dependent activation and oligomerization of plant NRC class helper NLRs by sensor NLR immune receptors Rpi-amr3 and Rpi-amr1

Hee-Kyung Ahn, Xiao Lin, Andrea Carolina Olave-Achury, Lida Derevnina, Mauricio Contreras, Jiorgos Kourelis, Chih-Hang Wu, Sophien Kamoun, and Jonathan D.G. Jones
DOI: 10.15252/embj.2022111484

Corresponding author(s): Jonathan D.G. Jones (jonathan.jones@tsl.ac.uk)

Review Timeline:

Submission Date:	25th Apr 22
Editorial Decision:	21st Jun 22
Revision Received:	30th Sep 22
Editorial Decision:	2nd Nov 22
Revision Received:	29th Nov 22
Editorial Decision:	30th Nov 22
Revision Received:	30th Nov 22
Accepted:	2nd Dec 22

Editor: William Teale

Transaction Report:

(Note: With the exception of the correction of typographical or spelling errors that could be a source of ambiguity, letters and reports are not edited. Depending on transfer agreements, referee reports obtained elsewhere may or may not be included in this compilation. Referee reports are anonymous unless the Referee chooses to sign their reports.)

Dear Dr. Jones,

Thank you for submitting your manuscript for consideration at the EMBO Journal. It has now been seen by three referees whose comments are shown below.

Before deciding whether to moving forward towards publication of your manuscript, I would very much like to discuss the referee reports with you over Zoom. This will help me judge whether the referees are asking for an achievable set of revisions. I am away at a conference next week, but will be available from the following Monday. Alternatively, I can offer a meeting on Thursday morning (23rd June). I understand this gives you a very limited amount of time to digest the reports; however, I am mindful that you might want to accelerate the editorial process after the longer time taken by a back-to-back submission of this kind.

I have sent a duplicate of this email to Dr Kamoun (but including the referee reports for his manuscript).

Yours sincerely,

William Teale

William Teale, PhD
Editor
The EMBO Journal
w.teale@embojournal.org

Referee #1:

General comment.
This study describes the mechanism of helper NLR activation by two different sensor NLRs. A detailed understanding of this mechanism is required to ultimately engineer disease resistance. Authors show that sensor NLRs Rpi-amr1 and 3 can recognize a specific effector and, in turn, activate the helper NLR NRC2, in a p-loop dependent manner. Authors further show that NRC2 activation leads to the formation of a high molecular weight oligomer similar to ZAR1, SR33, NRG1.1 and ADR1. This suggests that NRC2 function similarly, ie by forming a resistosome which may act as a cation channel. Interestingly, sensor and helper NLRs do not form a stable complex and the authors show without a doubt that sensor NLR do not form a resistosome, either alone or with helper NRC2. Overall, this study is designed, executed and written with care and brings high quality, valuable information. However, one part of the study, where the authors examine self-association of the sensor NLR, must be clarified before it can be published.

Major comments:
The part on Rpi-amr self-association needs to be clarified. Figure 1, panels B and D and figure 3B are not consistent with each other. Co-IP in 1D shows a clear self-association of RPI-amr1 and 3. Blue native page in 1D shows that HA-tagged Rpi-amr1 and 3 do migrate at levels consistent with dimers. However, the flag-tagged version of Rpi-amr1 do not show the same pattern of migration in 1B. We cannot conclude whether this effect results from the tag or is a genuine property of this NLR. This raises the question; to what extent tagged RPi-amr1 and 3 proteins are functional and represent the native protein function? Functionality of tagged Rpi-amr1 and 3 proteins should be tested in order to conclude.
For Rpi-amr3, both figure 1B and 3B show this NLR forms high molecular weight complexes and figure 1D shows it self-associate. Despite this data, it is concluded line 160 that these NLRs do not self-associate? Later, it is stated that they may (168) and then, in the discussion, it is stated that they do (line 290). Please clarify this matter.
Figure 6: Cation channel activity and defense is indicated in the schematics even though this aspect of NRC2 function is not investigated in the paper. At least, a question mark should be added.

Minor comments:
Please refrain from using too many acronyms. Controlled environment room (CER) is used only once for instance.
Line 397 solid L medium. LB?
References should be checked. I noticed a few missing details. Contreras et al misses a DOI number for instance.

Additional suggestions:
I have one suggestion to improve the manuscript although it should not delay the publication. Authors refer to the potential channel activity of NRC2 but it is not known whether NRC2 activity is even correlated to elevated cytosolic calcium levels (or did I miss this paper?). Observing cytosolic calcium levels during infection is not very complex and would indicate if NRC2 regulates (directly or indirectly) calcium levels. Authors emphasize that sensor NLR function through a specific helper NLR which explains

the restricted taxonomic functionality of NLRs. However, in other organisms, NLR resistosomes can function through a different mechanism (as scaffolds for instance in mammals). I feel that it would be important to test if resistosome formation and calcium influx are conserved mechanisms in plant immunity.

Referee #2:

The manuscript by Hee-Kyung Ahn et al., describes the effector-dependent activation and oligomerization of NRC2 helper NLR by two Solanaceae sensor NLRs, Rpi-amr3 and Rpi-amr1. Rpi-amr1 and Rpi-amr3 form a protein complex with AVRamr1 and AVRamr3 effectors, respectively. Both Rpi-amr1 and Rpi-amr3 induced the in vivo oligomerization of the helper NLR variant NRC2EEE upon recognition of cognate effectors AVRamr1 and AVRamr3. A take home message from this work is that Rpi-Amr1 and Rpi-Amr3 function as sensor NLRs and trigger oligomerization of the NRC2 helper NLR without themselves being part of the activated resistosome.

General comments and major points

This work advances our understanding of sensor-helper NLR pairs to some extent, but the key question of how sensor NLRs convey their signal to specific helper NLRs (in this case NRC2) remains unanswered/speculative. In addition, the stoichiometry of the postulated 'NRC2 resistosome' remains undefined and whether oligomeric NRC2 exhibits channel activity is not tested. Another limitation of the present work is the exclusive use of BN-PAGE to characterize NLR complexes, which is known to suffer from inherent limitations (e.g., estimation of complex size) and the lack of independent assays for complex characterization, such as size exclusion chromatography (SEC). Overall the manuscript is well-written and the conclusions are carefully formulated.

1. Detection of the 'NRC2 resistosome' (>700 kDa) relies exclusively on the transient gene OE of a single tested inactive variant, designated NRC2EEE, in N. benthamiana leaves (Fig. 2 to 5). Whether WT NRC2 forms the same or a similar complex remains unclear. The unexpected absence of Rpi-amr1 and Rpi-amr3 in the NRC2 complex may be due to the exclusive use of the non-functional NRC2EEE variant, which puts at least a question mark on the main conclusion of the present work. I understand the technical challenges, but detection of a WT NRC2 oligomer with or without associated sensor NLR after Rpi-amr1 or Rpi-amr3 activation would improve the significance of this study by an order of magnitude.

2. There is often functional redundancy between NRC helpers with respect to immune responses triggered by a particular sensor NLR (Wu et al., 2017). Rpi-amr1 and Rpi-amr3 exhibit NRC2/3-dependent and NRC2/3/4-dependent cell-death activity, respectively. Oligomerization of the non-functional NRC2EEE variant is shown by both AVRamr3/Rpi-amr3 and AVRamr1/Rpi-amr1. However, unlike AVRamr3/Rpi-amr3, AVRamr1/Rpi-amr1 displays cell death activity that is not dependent on NRC4 (Lin et al., 2021; Witek et al., 2021). It is critically important to examine in parallel NRC2EEE and NRC4EEE oligomerization after activation of Rpi-amr1 or Rpi-amr3, as this at least eliminates the possibility that the formation of the NRC2EEE complex is a feature restricted to NRC2.

3. Compelling evidence is provided for Rpi-amr3 induced NRC2EEE complex formation, which is dependent on a functional ATP-binding motif in the sensor NLR (Fig. 4). Did the authors also test the effects of a NRC2 P-loop mutation on Rpi-amr3-mediated cell death activity and NRC2 oligomerization?

4. Figure 1 The authors show that although Rpi-amr1 and Rpi-amr3 can self-associate in vivo, the majority of Rpi-amr1 and Rpi-amr3 exist as monomers. In BN-PAGE, the co-immunoprecipitated Rpi-amr1-HA or Rpi-amr3-HA migrates at a size >450 kDa, whereas Rpi-amr1-Flag and Rpi-amr3-Flag migrate at a size of 270 kDa. What is the author's explanation for this? Clearly, independent assessment of the size of these complexes by routine SEC will contribute significantly to better interpret the BN-PAGE data.

5. Figure 2 Did the authors perform co-immunoprecipitation assays to examine if Rpi-amr1 and/or Rpi-amr3 interacts with NRC2 in the pre-activated state? This is an important experiment.

6. In Fig. 2A, the bands of Rpi-arm3-HF are diffuse and the major bands cannot be distinguished, making it difficult to compare their size with or without effector protein. The authors should provide better quality data to support the conclusion that NRC2EEE-myc does not alter complex formation of Rpi-amr3 and AVRamr3.

7. Figure 2 C,D. The oligomerized NRC2EEE induced by AVRamr3/Rpi-amr3 and AVRamr1/Rpi-amr1 migrates as two bands. What is the author's explanation for this?

8. Figure 3 In the inactivated state, NRC2EEE and Rpi-amr3 migrate to ~240 kDa. Upon activation, NRC2EEE-Myc migrated to ~900 kDa, while migration of Rpi-amr3 shifted tos ~480 kDa. The authors did not comment on the observed shift in the size of Rpi-amr3 upon activation. This is in contrast to the BN-PAGE assays (Fig. 1) where no change in the migration pattern of Rpi-amr1/3 was observed pre- and post activation.

9. Did the authors observe membrane enrichment of the NRC2EEE variant upon Rpi-amr1 and Rpi-amr3 sensor NLR activation?

10. Oligomerization of NRC2 is required for membrane association and HR. To further confirm this conclusion, the authors should test whether the auto-active variant NRC2H480R (Derevnina et al., 2021) is constitutively oligomerized by introducing the N-terminal EEE mutations in NRC2H480R to avoid cell death.

11. Fig. 6. Without biochemical evidence for NRC2 ion channel activity, the model presented does not appear justified. Furthermore, no evidence for NRC2 pentamer stoichiometry is presented. No data are presented to show that WT NRC2 can form a homopentamer with channel activity, and for this reason it is safer to delete this figure from the manuscript.

Minor points

Figure 4 legend: Figure 4 D not E.

Referee #3:

Transient Agrobacterium infiltration in N. benthamiana was used as a model system to study protein complexes of effector-dependent sensor NLR activation. In particular the immune activation mechanism of Rpi-amr3 or Rpi-amr1 via NRC2 were analysed upon incubation with effector proteins (AVRamr*) from Phytophtera species.
Blue-native PAGE was used to show that AVRamr1 together with Rpi-amr1, AVRamr3 together with Rpi-amr3 form complexes. Rpi-amr1 and Rpi-amr3 seem to form self-associations of Flag and HA variants. What I do not understand is, why the complexes from Fig 1B with Flag-antibody do not show a double band? One in the region 272kDa and one in 450 kDa? It seems that Fig 1 B,C,D show independent experiments with the same setting. I am not so sure if the mass ladder was assigned correctly in Fig 1D. Maybe the authors could have another evaluation of the blots and consider if the prominent high molecular band always represent a complex from Rpi* forms together with or without AVRamr*. In other words, Fig1BC does not fit with Fig1D. In Fig 1B, almost the same size was shown between left two bands. Please explain if the small difference could be the delta size between with and without AVRamr. Please calculate from the single molecular masses, what could be the stoichiometry.
Interesting that cotransfection of NRC2-Myc with Rpi-amr3 and AVR-amr3 lead to degradation (EV3B). In that gel, the shift between with and without AVRamr3 is a clearer than in Fig. 1. Did you run the gel a bit longer?
NRC2 forms a high molecular complex around 900 kDa upon expression of tagged Rpi-amr3 and AVR-amr3. The mass ladder of Fig 2D was again not clear and needs reinspection. Maybe use semilog plots to show the molecular size in native gels. What could be the stoichiometry based on your observation. Please discuss.
Fig. 4 shows that the P-loop of Rpi-amr3 is not needed to bind the effector AVR-amr3, as the heterooligomer is still formed in a mutation (Fig 4B/C). Important, the oligomerization was affected by the mutation. Interesting, NRC2 resistosomes were even formed upon incubation of effectors AVRamr3 from different Phytophtera species but not all, indicating that the binding of AVR-amr* and Rpi-amr* is the important interaction to induce NRC2 oligomerization.
I am not a specialist in the field of effector-dependent sensor NLR activation in plants. I found the topic very interesting, as it was a pleasure for me to hear something about the world how plants recognise their invaders and how they react upon infection. As I am using a lot of native gels in my research, I had a closer look to the BN-PAGE and the interpretation. The performance and the resolution are in a very good quality and the figures are clear. I am impressed, about the BN-PAGE from such a small amount of samples (10 disks of leaves)!
All together the quality of the work is very good and the manuscript was clearly written.
Minor points:
Line 113: explain abbreviation RXLR, when mentioned first time
Line 114: explain WY domains
Fig.2 "only the immunoblotting step was performed separately", Do you mean sequentially on the same membrane, or did you cut them and decorated independently?
What represents GUS-V5??? Please explain, I could not find in the text, what it is.

Dear Dr. Jones,

Thank you for submitting your manuscript for consideration at the EMBO Journal. It has now been seen by three referees whose comments are shown below.

Before deciding whether to moving forward towards publication of your manuscript, I would very much like to discuss the referee reports with you over Zoom. This will help me judge whether the referees are asking for an achievable set of revisions. I am away at a conference next week, but will be available from the following Monday. Alternatively, I can offer a meeting on Thursday morning (23rd June). I understand this gives you a very limited amount of time to digest the reports; however, I am mindful that you might want to accelerate the editorial process after the longer time taken by a back-to-back submission of this kind.

I have sent a duplicate of this email to Dr Kamoun (but including the referee reports for his manuscript).

Yours sincerely,

William Teale

William Teale, PhD
Editor
The EMBO Journal
w.teale@embojournal.org

Referee #1:

General comment.
This study describes the mechanism of helper NLR activation by two different sensor NLRs. A detailed understanding of this mechanism is required to ultimately engineer disease resistance. Authors show that sensor NLRs Rpi-amr1 and 3 can recognize a specific effector and, in turn, activate the helper NLR NRC2, in a p-loop dependent manner. Authors further show that NRC2 activation leads to the formation of a high molecular weight oligomer similar to ZAR1, SR33, NRG1.1 and ADR1. This suggests that NRC2 function similarly, ie by forming a resistosome which may act as a cation channel. Interestingly, sensor and helper NLRs do not form a stable complex and the authors show without a doubt that sensor NLR do not form a resistosome, either alone or with helper NRC2. Overall, this study is designed, executed and written with care and brings high quality, valuable information. However, one part of the study, where the authors examine self-association of the sensor NLR, must be clarified before it can be published.

Major comments:
The part on Rpi-amr self-association needs to be clarified. Figure 1, panels B and D and figure 3B are not consistent with each other. Co-IP in 1D shows a clear self-association of RPI-amr1 and 3. Blue native page in 1D shows that HA-tagged Rpi-amr1 and 3 do migrate at levels consistent with dimers. However, the flag-tagged version of Rpi-amr1 do not show the same pattern of migration in 1B. We cannot conclude whether this effect results from the tag or is a genuine property of this NLR. This raises the question; to what extent tagged RPi-amr1 and 3 proteins are functional and represent the native protein function? Functionality of tagged Rpi-amr1 and 3 proteins should be tested in order to

conclude.
For Rpi-amr3, both figure 1B and 3B show this NLR forms high molecular weight complexes and figure 1D shows it self-associate. Despite this data, it is concluded line 160 that these NLRs do not self-associate? Later, it is stated that they may (168) and then, in the discussion, it is stated that they do (line 290). Please clarify this matter.
Figure 6: Cation channel activity and defense is indicated in the schematics even though this aspect of NRC2 function is not investigated in the paper. At least, a question mark should be added.

We thank the reviewer for the helpful comments. The proportion of Rpi-amr1 and Rpi-amr3 that self-associates is substantially less than the monomeric forms of Rpi-amr1 and Rpi-amr3. Importantly, this slight self-association does not change in the presence of recognized effector, suggesting it is irrelevant to signalling upon effector detection. This is why we consider that these self-associating forms are not visible in the BNP gel detecting for Flag-epitope tagged Rpi-amr1 and Rpi-amr3 and only visible for the co-immunoprecipitated Rpi-amr1 or Rpi-amr3-HA proteins. We show data comparing the total amount of Rpi-amr1 and Rpi-amr3 with the immunoprecipitated amount, and show Flag blots with higher exposure, clearly showing the very small proportion of self-association, and make this point clearer in our revision.
We appreciate the reviewer's comments regarding the text, and we edited our manuscript to clarify the self-association of sensor NLRs, as well as the low-abundance of these self-associating forms compared to primarily monomeric sensor NLRs.
Regarding cation channel activity, we tried testing for changes in cytosolic calcium level with calcium sensor as suggested by the reviewer. However, the onset of HR was too fast, and it was difficult to capture the right time-point for calcium level measurement in our current transient expression system. We aim to design chemical-inducible constructs for AVRamr3 or AVRamr1 with NRC2 and NRC2^{EEE} mutant construct and generate transgenic lines in the *nrc234 KO* and attempt again for calcium level measurement. As our experiments do not provide evidence for NRC2 as calcium channel, we have removed the phrase "cation channel activity" in Figure 6.

Minor comments:
Please refrain from using too many acronyms. Controlled environment room (CER) is used only once for instance.
Line 397 solid L medium. LB?
References should be checked. I noticed a few missing details. Contreras et al misses a DOI number for instance.

We will reduce the use of acronyms. Solid L medium contains glucose together with tryptone, yeast extract, and sodium chloride of the LB medium. L medium was originally named "L broth" (Luria and Burrous, PMID: 13475269). References will be double checked, and changes including DOI number for Contreras *et al* will be made.

Additional suggestions:
I have one suggestion to improve the manuscript although it should not delay the publication. Authors refer to the potential channel activity of NRC2 but it is not known whether NRC2 activity is even correlated to elevated cytosolic calcium levels (or did I miss this paper?). Observing cytosolic calcium levels during infection is not very complex and would indicate if NRC2 regulates (directly or indirectly) calcium levels. Authors emphasize that sensor NLR function through a specific helper NLR which explains the restricted taxonomic functionality of NLRs. However, in other organisms, NLR resistosomes can function through a different mechanism (as scaffolds for instance in mammals). I feel that it would be important to test if resistosome formation and calcium influx are conserved mechanisms in plant immunity.

We tried testing for changes in cytosolic calcium level with calcium sensor as suggested by the reviewer. However, the onset of HR was too fast, and was difficult to capture the right time-point for calcium level measurement. We aim to design chemical-inducible constructs for AVRamr3 or AVRamr1 with NRC2^{EEE} mutant construct and generate transgenic lines in the *nrc234 KO* and attempt again for calcium level measurement.

Referee #2:

The manuscript by Hee-Kyung Ahn et al., describes the effector-dependent activation and oligomerization of NRC2 helper NLR by two Solanaceae sensor NLRs, Rpi-amr3 and Rpi-amr1. Rpi-amr1 and Rpi-amr3 form a protein complex with AVRamr1 and AVRamr3 effectors, respectively. Both Rpi-amr1 and Rpi-amr3 induced the in vivo oligomerization of the helper NLR variant NRC2EEE upon recognition of cognate effectors AVRamr1 and AVRamr3. A take home message from this work is that Rpi-Amr1 and Rpi-Amr3 function as sensor NLRs and trigger oligomerization of the NRC2 helper NLR without themselves being part of the activated resistosome.

General comments and major points

This work advances our understanding of sensor-helper NLR pairs to some extent, but the key question of how sensor NLRs convey their signal to specific helper NLRs (in this case NRC2) remains unanswered/speculative. In addition, the stoichiometry of the postulated 'NRC2 resistosome' remains undefined and whether oligomeric NRC2 exhibits channel activity is not tested. Another limitation of the present work is the exclusive use of BN-PAGE to characterize NLR complexes, which is known to suffer from inherent limitations (e.g., estimation of complex size) and the lack of independent assays for complex characterization, such as size exclusion chromatography (SEC). Overall the manuscript is well-written and the conclusions are carefully formulated.

We thank the reviewer for the comments. We would like to emphasize that the major finding of this paper is the oligomerization of NRC-type helper NLRs upon effector recognition by sensor NLR. Previously Jacob *et al*. (PMID: 34140391) showed oligomerization of helper NLR NRG1, but autoactive alleles of NRG1 were used. Our study investigates effector-dependent helper NLR oligomerization. We would like to further investigate how the signal from sensor NLRs is relayed to helper NLRs, in a detailed, spatiotemporal manner in the future. Our further investigation will include the role of NRC2 oligomer as a possible cation channel. Many studies regarding CC-NLRs have already alluded to the calcium channel activity required for activation, however, we agree with the reviewer that this manuscript does not include evidence regarding calcium channel activity of NRC2. Therefore, we will remove the phrase “cation channel activity” in figure 6. The semi-log plot measurement suggested by another reviewer indicated the NRC2 oligomer is 5 times larger than the inactive NRC2, and with these data, we would like to keep our schematic model as is.

1. Detection of the 'NRC2 resistosome' (>700 kDa) relies exclusively on the transient gene OE of a single tested inactive variant, designated NRC2EEE, in N. benthamiana leaves (Fig. 2 to 5). Whether WT NRC2 forms the same or a similar complex remains unclear. The unexpected absence of Rpi-amr1 and Rpi-amr3 in the NRC2 complex may be due to the exclusive use of the non-functional NRC2EEE variant, which puts at least a question mark on the main conclusion of the present work. I understand the technical challenges, but detection of a WT NRC2 oligomer with or without associated sensor NLR after Rpi-amr1 or Rpi-amr3 activation would improve the significance of this study by an order of magnitude.

Regarding NRC2-WT, we tested for its expression multiple times, but could not resolve its oligomerization because it is destabilized upon immune activation and cell death.

Regarding the authenticity of the behaviour of the NRC2^{EEE} protein, Förderer et al (2022) in Nature (PMID: 36163289 resolved the structure of a wheat CC-NLR Sr35. Due to cell death induction of Sr35 in insect cells, the authors in this study mutated L15 and L19 to glutamate (E), which are the corresponding residues to L13E and L17E from the NRC2^{EEE} construct. Due to this, the structural resolution of N-terminal of Sr35 was low. However, upon effector activation, the mutant Sr35 formed a pentamer, nevertheless.
Another study conducted using ZAR1 (Hu et al., PMID: 32194243) also highlights the ability of ZAR1 to form oligomer in Blue native-PAGE. For this study, authors mutated the glutamate residues E11 and E18 to alanine that leads to loss of negative charges lining the cation channel for cation transport. Mutation of these residues did not hamper ZAR1 oligomerization upon effector recognition.
All of these results are consistent with the critical mechanism of NLR activation being via 'induced proximity' (Duxbury et al. 2020, PMID: 32709746). The NB-ARC domains come together upon effector recognition, but the signal activation occurs through domains at the N-terminus, but not the NB-ARC which provides the engine to drive ATP-dependent 'induced proximity' by NB-ARC or NACHT oligomerization. Such examples are well shown not only in the two studies described above, but also in studies on animal NLRs, where the NACHT domains, like the NB-ARC domains, induce proximity of CARD domains for signal activation.
Thus, we think that not only is using the WT NRC2 for oligomerization in this study impossible due to lethality, but also unnecessary as the conformational change of the N-terminal MADA motif is the consequence of oligomerization and not the cause of it.

2. There is often functional redundancy between NRC helpers with respect to immune responses triggered by a particular sensor NLR (Wu et al., 2017). Rpi-amr1 and Rpi-amr3 exhibit NRC2/3-dependent and NRC2/3/4-dependent cell-death activity, respectively. Oligomerization of the non-functional NRC2EEE variant is shown by both AVRamr3/Rpi-amr3 and AVRamr1/Rpi-amr1. However, unlike AVRamr3/Rpi-amr3, AVRamr1/Rpi-amr1 displays cell death activity that is not dependent on NRC4 (Lin et al., 2021; Witek et al., 2021). It is critically important to examine in parallel NRC2EEE and NRC4EEE oligomerization after activation of Rpi-amr1 or Rpi-amr3, as this at least eliminates the possibility that the formation of the NRC2EEE complex is a feature restricted to NRC2.

The reviewer suggests an interesting idea. However, the focus of our paper is on how the sensor NLRs activate helper NLRs, and what are the essential components of the sensor NLRs that are required for activation. The suggested experiments are beyond the scope of this paper, and are being studied by the Kamoun group with the same results.

3. Compelling evidence is provided for Rpi-amr3 induced NRC2EEE complex formation, which is dependent on a functional ATP-binding motif in the sensor NLR (Fig. 4). Did the authors also test the effects of a NRC2 P-loop mutation on Rpi-amr3-mediated cell death activity and NRC2 oligomerization?

We have tested the NRC2 P-loop mutation with EEE mutation and this mutant does not oligomerise. We added these data to our revised manuscript in Figure 4C. We also include the HR assay data of the NRC2 P-loop mutant in Figure 4B.

4. Figure 1 The authors show that although Rpi-amr1 and Rpi-amr3 can self-associate in vivo, the majority of Rpi-amr1 and Rpi-amr3 exist as monomers. In BN-PAGE, the co-immunoprecipitated Rpi-amr1-HA or Rpi-amr3-HA migrates at a size >450 kDa, whereas Rpi-amr1-Flag and Rpi-amr3-Flag migrate at a size of 270 kDa. What is the author's explanation for this? Clearly, independent

assessment of the size of these complexes by routine SEC will contribute significantly to better interpret the BN-PAGE data.

The key point is that co-immunoprecipitated Rpi-amr1-HA and Rpi-amr3-HA are very low in abundance compared to monomer. Crucially, (i) only a small proportion of Rpi-amr1 and Rpi-amr3 self-associates (ii) this proportion does not change in the presence of effector. We think that these differences in proportion leads to the apparent absence of >450kDa band in the Flag blots. In order to present this additional information regarding the relative amounts of self-associating and monomeric Rpi-amr1 and Rpi-amr3, we add the uncropped blots of input and IP comparing the protein amounts, and also higher exposure of the Flag blot. We also moved the self-association data to Figure EV3

5. Figure 2 Did the authors perform co-immunoprecipitation assays to examine if Rpi-amr1 and/or Rpi-amr3 interacts with NRC2 in the pre-activated state? This is an important experiment.

We have tested on multiple occasions for co-immunoprecipitation of Rpi-amr1/3 with NRC2. Weak co-IP signals are sometimes detected, irrespective of the presence of Avramr1/3. We concluded that NRC2 is somewhat sticky, and so these co-IP data are inconclusive. We are following up with extensive detailed time course experiments using chemical inducible Avramr1/3, but these data are not yet available and are beyond the scope of this paper.

6. In Fig. 2A, the bands of Rpi-arm3-HF are diffuse and the major bands cannot be distinguished, making it difficult to compare their size with or without effector protein. The authors should provide better quality data to support the conclusion that NRC2EEE-myc does not alter complex formation of Rpi-amr3 and AVRamr3.

We show a better resolution image of Rpi-amr3 with AVRamr3 and these data are now in Figure 1.

7. Figure 2 C,D. The oligomerized NRC2EEE induced by AVRamr3/Rpi-amr3 and AVRamr1/Rpi-amr1 migrates as two bands. What is the author's explanation for this?

We cannot observe this particular band for inactive NRC2. The double band of NRC2 is also observed in Contreras *et al.* (https://doi.org/10.1101/2022.04.25.489342), indicating that these may be intermediate features or degraded product of the NRC2 complex.

8. Figure 3 In the inactivated state, NRC2EEE and Rpi-amr3 migrate to ~240 kDa. Upon activation, NRC2EEE-Myc migrated to ~900 kDa, while migration of Rpi-amr3 shifted tos ~480 kDa. The authors did not comment on the observed shift in the size of Rpi-amr3 upon activation. This is in contrast to the BN-PAGE assays (Fig. 1) where no change in the migration pattern of Rpi-amr1/3 was observed pre- and post activation.

We reported that the Rpi-amr3/Avramr3 heterodimer migrates at ~ 480 kD in this gel system. In Figure EV2, as well as Figure 1, we show change in migration pattern after AVRamr3 co-expression with Rpi-amr3. In figure 3, a shift in the signal intensity of Rpi-amr3 can also be observed upon activation. We will clarify in the manuscript.

9. Did the authors observe membrane enrichment of the NRC2EEE variant upon Rpi-amr1 and Rpi-amr3 sensor NLR activation?

We tested for membrane enrichment of NRC2^{EEE} variant upon Rpi-amr1 and Rpi-amr3 sensor NLR activation and added in Figure 3D, E. We observed enrichment of NRC2 EEE in the pellet fraction upon activation. We did not see changes for Rpi-amr1, and slight enrichment for Rpi-amr3. Both AVRamr1 and AVRamr3 were enriched in the pellet fractions.

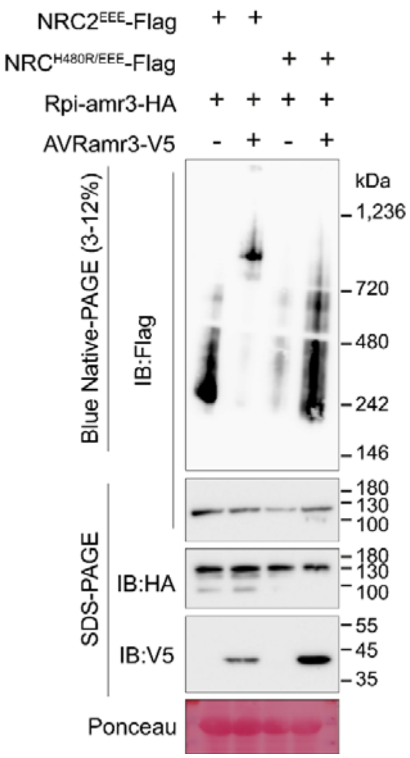

10. Oligomerization of NRC2 is required for membrane association and HR. To further confirm this conclusion, the authors should test whether the auto-active variant NRC2H480R (Derevnina et al., 2021) is constitutively oligomerized by introducing the N-terminal EEE mutations in NRC2H480R to avoid cell death.

We tested the autoactive variant of NRC2^{H480R} with EEE mutation. However, we could not observe any oligomers of the NRC2$^{H480R/EEE}$ mutation. The corresponding data is attached. We also could not prompt oligomerization of the NRC2$^{H480R/EEE}$ mutation in the presence of Rpi-amr3 and AVRamr3. This suggests that the autoactivity of NRC2$^{H480R/EEE}$ is different from the effector-dependent activation of NRC2. It's also been reported in Derevnina et al. (PMID: 34424903) that this allele is only weakly autoactive.

11. Fig. 6. Without biochemical evidence for NRC2 ion channel activity, the model presented does not appear justified. Furthermore, no evidence for NRC2 pentamer stoichiometry is presented. No data are presented to show that WT NRC2 can form a homopentamer with channel activity, and for this reason it is safer to delete this figure from the manuscript.

We thank the reviewer for the comment. We tested for cytosolic calcium influx by using the calcium sensor aequorin. However, due to the HR occurring too fast, we could not settle on the right time point to test for calcium level measurement in our transient co-expression system. We also tested transient expression of AVRamr3-inducible constructs, but this led to leakiness and cell death due to recognition. We are planning to generate transgenic lines of inducible AVRamr1 or AVRamr3 constructs with NRC2 mutant, and test for calcium measurement in the future.
Given Sr35 from wheat and Zar1 from Arabidopsis form a pentamer when activated, and that oligomerized NRC2 (120 kDa) appears to migrate in BN-PAGE at ~ 900kDa, the reviewer is right to point out that NRC2 may oligomerize to a form greater than a pentamer. However, when we analyzed the expected band size using semi-log plots, as suggested by Reviewer 3, the NRC2 monomer migrated at ~180 kDa and the oligomer at ~900kDa. The most parsimonious interpretation of this data is that there may be 5 molecules of NRC2 forming a complex upon oligomerization. Therefore, we would prefer to keep the models of NRC2 as pentamers the same but remove the phrase "cation channel activity".

Minor points

Figure 4 legend: Figure 4 D not E.

We will amend the figure legend accordingly.

Referee #3:

Transient Agrobacterium infiltration in N. benthamiana was used as a model system to study protein complexes of effector-dependent sensor NLR activation. In particular the immune activation mechanism of Rpi-amr3 or Rpi-amr1 via NRC2 were analysed upon incubation with effector proteins (AVRamr*) from Phytophtera species.

Blue-native PAGE was used to show that AVRamr1 together with Rpi-amr1, AVRamr3 together with Rpi-amr3 form complexes. Rpi-amr1 and Rpi-amr3 seem to form self-associations of Flag and HA variants. What I do not understand is, why the complexes from Fig 1B with Flag-antibody do not show a double band? One in the region 272kDa and one in 450 kDa? It seems that Fig 1 B,C,D show independent experiments with the same setting. I am not so sure if the mass ladder was assigned correctly in Fig 1D. Maybe the authors could have another evaluation of the blots and consider if the prominent high molecular band always represent a complex from Rpi* forms together with or without AVRamr*. In other words, Fig1BC does not fit with Fig1D.

We loaded the samples in duplicate onto one gel, and then split the membranes for blotting with separate antibodies. We tried to blot the images side by side, but due to the significantly weak signal of self-association Rpi-amr1 and Rpi-amr3, this could not be done. This shows that indeed self-associating Rpi-amr1 and Rpi-amr3 are higher in molecular weight. However, we want to clarify that the amounts of Rpi-amr1 and Rpi-amr3 co-immunoprecipitated by Rpi-amr1 and Rpi-amr3-Flag are much lower in abundance. Therefore, we think that the reason Rpi-amr1-Flag and Rpi-amr3-Flag are not resolved in double bands is due to this difference in accumulation of monomeric and self-associating Rpi-amr proteins. We additionally show uncropped blots of immunoprecipitations to highlight the differences in abundance of lysate and the immunoprecipitates. Also, we added the higher exposure of the Flag blots (Figure EV3C).

In Fig 1B, almost the same size was shown between left two bands. Please explain if the small difference could be the delta size between with and without AVRamr. Please calculate from the single molecular masses, what could be the stoichiometry.

AVRamr1 is approximately 35 kDa in size, and therefore addition of it will not cause a significant retardation. We have re-inspected the multiple biological/technical replicates we have tested and concluded that there was no change in the migration pattern of Rpi-amr1 before and after effector recognition. This may suggest an interesting hypothesis whereby AVRamr1 binds to Rpi-amr1 in a competitive manner with an unknown protein of similar size, that leads to non-detectable change in migration of Rpi-amr1 in terms of molecular weight. However, the main point of this paper is not the unique properties of Rpi-amr1 that distinguish it from Rpi-amr3, but rather that both provoke oligomerisation of NRC2.

Interesting that cotransfection of NRC2-Myc with Rpi-amr3 and AVR-amr3 lead to degradation (EV3B). In that gel, the shift between with and without AVRamr3 is a clearer than in Fig. 1. Did you run the gel a bit longer?

The gels were run for longer, and the immunoprecipitation conditions were different. We have adjusted the immunoprecipitation conditions and the running time of gels and provide a better resolution image for Fig 2A. These data are now in Figure 1.

NRC2 forms a high molecular complex around 900 kDa upon expression of tagged Rpi-amr3 and AVR-amr3. The mass ladder of Fig 2D was again not clear and needs reinspection. Maybe use

semilog plots to show the molecular size in native gels. What could be the stoichiometry based on your observation. Please discuss.

We thank the reviewer for helpful suggestion. We utilized semi-log plots for multiple biological replicates of NRC2 oligomers. Across multiple biological replicates, we observed that the NRC2 oligomer was approximately 5 times higher in molecular weight compared to NRC2 monomer. Furthermore, the size comparison between NRC2 oligomer formed by Rpi-amr1 and Rpi-amr3 were nearly identical. We added these data, which are statistically significant (Wilcoxon test) in Figure 2D.

Fig. 4 shows that the P-loop of Rpi-amr3 is not needed to bind the effector AVR-amr3, as the heterooligomer is still formed in a mutation (Fig 4B/C). Important, the oligomerization was affected by the mutation. Interesting, NRC2 resistosomes were even formed upon incubation of effectors AVRamr3 from different Phytophtera species but not all, indicating that the binding of AVR-amr* and Rpi-amr* is the important interaction to induce NRC2 oligomerization.

In Lin et al. (https://doi.org/10.1016/j.molp.2022.07.012) we showed that the interaction of Rpi-amr3 and the alleles of AVRamr3 is indeed correlated with recognition. Our study expands this finding further into correlation of AVRamr3 interaction with Rpi-amr3 into NRC2 oligomer formation, and cell death activation.

I am not a specialist in the field of effector-dependent sensor NLR activation in plants. I found the topic very interesting, as it was a pleasure for me to hear something about the world how plants recognise their invaders and how they react upon infection. As I am using a lot of native gels in my research, I had a closer look to the BN-PAGE and the interpretation. The performance and the resolution are in a very good quality and the figures are clear. I am impressed, about the BN-PAGE from such a small amount of samples (10 disks of leaves)!
All together the quality of the work is very good and the manuscript was clearly written.

We thank the reviewer for the comment.

Minor points:
Line 113: explain abbreviation RXLR, when mentioned first time
Line 114: explain WY domains
Fig.2 "only the immunoblotting step was performed separately", Do you mean sequentially on the same membrane, or did you cut them and decorated independently?
What represents GUS-V5??? Please explain, I could not find in the text, what it is.

We edited the manuscript with less acronyms and explain more clearly. The representation of GUS-V5 was added in the corresponding figure legends.
For Fig.2, the gel was cut into two and were blotted with different antibodies. Same samples were loaded in duplicate.

Dear Jonathan,

We have now received re-review reports from two referees. As you will see, you have addressed the majority of their concerns satisfactorily. However, as you will see, Referee 2 maintains (and I agree) that a direct comparison of Rpi-amr1-activated NRC2EEE and NRC4EEE oligomers would significantly improve the manuscript. I would like to invite you consider whether any data (either directly or through collaboration) could be made available in this direction. There are also some remaining editorial points which need to be addressed which I am listing in order to save further rounds of revision. In this regard would you please:

- Make sure that the final text file (.doc) contains no figures and no track changes
- Rename the conflict of interest statement as the "DISCLOSURE AND COMPETING INTERESTS STATEMENT"
- Please remove the AC/CrediT section from the text
- Remove one mention of BB/P021646/1 and Gatsby Charitable Foundation (GATSBY), which are both listed twice in our eJP website
- Change the names of table callouts to Appendix Table S1-S2
- Include an Appendix 1 file containg a table of contents with page numbers
- Separate and reorganize Source Data files to one file/folder per figure and zip

EMBO Press is an editorially independent publishing platform for the development of EMBO scientific publications.

Best wishes,

William

William Teale, PhD
Editor
The EMBO Journal
w.teale@embojournal.org

https://emboj.msubmit.net/cgi-bin/main.plex

Referee #1:

The authors addressed all of my concerns.

Referee #2:

In the revised manuscript by Ahn H.K. et al. the authors have addressed and resolved some of my original comments and questions. However, I am surprised that they missed the opportunity to test differential activation of NRC2EEE oligomers, but not NRC4EEE, following effector-dependent activation of Rpi-amr1, as this experiment could have been performed in a reasonable amount of time and is an important control for the formation of specific NLR helper complexes following Rpi-amr1 or Rpi-amr3 activation. Furthermore, this would have added distinctiveness to this manuscript compared to the companion paper.

In addition, I suggest that the authors add to the discussion for the readership that i) it remains unclear how effector-induced Rpi-amr1 or Rpi-amr3 sensor NLRs transmit signals to helper NLRs, as this is an important limitation of the present study, and ii) that the biochemical activity of the NRC2 helper complex remains to be defined.

Much more is needed to define the biochemical mechanisms underlying sensor-helper NLR-mediated immunity in plants.

The authors have addressed all the reviewers' comments.

2nd Revision - Editorial Decision 30th Nov 2022

Dear Jonathan,

Thank you for uploading the files with the requested changes made. Before I can finally click 'accept' though, there are a couple of small outstanding points:

please remove the table legends from the manuscript file and include them as separate tabs of the Excel files. The tables should be renamed Dataset EV1 and EV2 and called out accordingly.

Are you able to increase the resolution of your tobacco leaf images in figure EV1 panels E and F? The text is fine, but the leaves themselves are very low resolution. The colour scale from the CCD camera is illegible.

Best wishes,

William

William Teale, PhD
Editor
The EMBO Journal
w.teale@embojournal.org

Instructions for preparing your revised manuscript:

Please check that the title and abstract of the manuscript are brief, yet explicit, even to non-specialists.

When assembling figures, please refer to our figure preparation guideline in order to ensure proper formatting and readability in print as well as on screen:
https://bit.ly/EMBOPressFigurePreparationGuideline
See also figure legend guidelines: https://www.embopress.org/page/journal/14602075/authorguide#figureformat

IMPORTANT: When you send the revision we will require
- a point-by-point response to the referees' comments, with a detailed description of the changes made (as a word file).
- a word file of the manuscript text.
- individual production quality figure files (one file per figure)
- a complete author checklist, which you can download from our author guidelines (https://www.embopress.org/page/journal/14602075/authorguide).
- Expanded View files (replacing Supplementary Information)
Please see out instructions to authors
https://www.embopress.org/page/journal/14602075/authorguide#expandedview

Please remember: Digital image enhancement is acceptable practice, as long as it accurately represents the original data and conforms to community standards. If a figure has been subjected to significant electronic manipulation, this must be noted in the figure legend or in the 'Materials and Methods' section. The editors reserve the right to request original versions of figures and the original images that were used to assemble the figure.

Further information is available in our Guide For Authors: https://www.embopress.org/page/journal/14602075/authorguide

We realize that it is difficult to revise to a specific deadline. In the interest of protecting the conceptual advance provided by the work, we recommend a revision within 3 months (28th Feb 2023). Please discuss the revision progress ahead of this time with the editor if you require more time to complete the revisions. Use the link below to submit your revision:

https://emboj.msubmit.net/cgi-bin/main.plex

All editorial and formatting issues were resolved by the authors.

3rd Revision - Editorial Decision 2nd Dec 2022

Dear Jonathan,

I am pleased to inform you that your manuscript has been accepted for publication in the EMBO Journal.

Congratulations to you and your group on a really nice study!

Please note that it is EMBO Journal policy for the transcript of the editorial process (containing referee reports and your response letter) to be published as an online supplement to each paper. If you do NOT want this, you will need to inform the Editorial Office via email immediately. More information is available here: https://www.embopress.org/page/journal/14602075/authorguide#transparentprocess

Your manuscript will be processed for publication in the journal by EMBO Press. Manuscripts in the PDF and electronic editions of The EMBO Journal will be copy edited, and you will be provided with page proofs prior to publication. Please note that supplementary information is not included in the proofs.

You will be contacted by Wiley Author Services to complete licensing and payment information. The required 'Page Charges Authorization Form' is available here: https://www.embopress.org/pb-assets/embo-site/tej_apc.pdf - please download and complete the form and return to embopressproduction@wiley.com

EMBO Press participates in many Publish and Read agreements that allow authors to publish Open Access with reduced/no publication charges. Check your eligibility: https://authorservices.wiley.com/author-resources/Journal-Authors/open-access/affiliation-policies-payments/index.html

Should you be planning a Press Release on your article, please get in contact with embojournal@wiley.com as early as possible, in order to coordinate publication and release dates.

If you have any questions, please do not hesitate to call or email the Editorial Office. Thank you for your contribution to The EMBO Journal.

Yours sincerely,

William

William Teale, PhD
Editor
The EMBO Journal
w.teale@embojournal.org

** Click here to be directed to your login page: https://emboj.msubmit.net

EMBO Press Author Checklist

Corresponding Author Name: Jonathan Jones	
Journal Submitted to: EMBO Journal	
Manuscript Number: EMBOJ-2022-111484	

USEFUL LINKS FOR COMPLETING THIS FORM
The EMBO Journal - Author Guidelines
EMBO Reports - Author Guidelines
Molecular Systems Biology - Author Guidelines
EMBO Molecular Medicine - Author Guidelines

Reporting Checklist for Life Science Articles (updated January
This checklist is adapted from Materials Design Analysis Reporting (MDAR) Checklist for Authors. MDAR establishes a minimum set of requirements in transparent reporting in the life sciences (see Statement of Task: 10.31222/osf.io/9sm4x). Please follow the journal's guidelines in preparing your
Please note that a copy of this checklist will be published alongside your article.

Abridged guidelines for figures
1. Data
The data shown in figures should satisfy the following conditions:
- the data were obtained and processed according to the field's best practice and are presented to reflect the results of the experiments in an accurate and unbiased manner.
- ideally, figure panels should include only measurements that are directly comparable to each other and obtained with the same assay.
- plots include clearly labeled error bars for independent experiments and sample sizes. Unless justified, error bars should not be shown for technical
- if n<5, the individual data points from each experiment should be plotted. Any statistical test employed should be justified.
- Source Data should be included to report the data underlying figures according to the guidelines set out in the authorship guidelines on Data

2. Captions
Each figure caption should contain the following information, for each panel where they are relevant:
- a specification of the experimental system investigated (eg cell line, species name).
- the assay(s) and method(s) used to carry out the reported observations and measurements.
- an explicit mention of the biological and chemical entity(ies) that are being measured.
- an explicit mention of the biological and chemical entity(ies) that are altered/varied/perturbed in a controlled manner.
- the exact sample size (n) for each experimental group/condition, given as a number, not a range;
- a description of the sample collection allowing the reader to understand whether the samples represent technical or biological replicates (including how many animals, litters, cultures, etc.).
- a statement of how many times the experiment shown was independently replicated in the laboratory.
- definitions of statistical methods and measures:
  - common tests, such as t-test (please specify whether paired vs. unpaired), simple χ2 tests, Wilcoxon and Mann-Whitney tests, can be unambiguously identified by name only, but more complex techniques should be described in the methods section;
  - are tests one-sided or two-sided?
  - are there adjustments for multiple comparisons?
  - exact statistical test results, e.g., P values = x but not P values < x;
  - definition of 'center values' as median or average;
  - definition of error bars as s.d. or s.e.m.

Please complete ALL of the questions below.
Select "Not Applicable" only when the requested information is not relevant for your study.

Materials

Newly Created Materials	Information included in the manuscript?	In which section is the information available? (Reagents and Tools Table, Materials and Methods, Figures, Data Availability Section)
New materials and reagents need to be available; do any restrictions apply?	Yes	Availability of data and materials

Antibodies	Information included in the manuscript?	In which section is the information available? (Reagents and Tools Table, Materials and Methods, Figures, Data Availability Section)
For **antibodies** provide the following information:<br>- Commercial antibodies: RRID (if possible) or supplier name, catalogue number and or/clone number<br>- Non-commercial: RRID or citation	Yes	Methods

DNA and RNA sequences	Information included in the manuscript?	In which section is the information available? (Reagents and Tools Table, Materials and Methods, Figures, Data Availability Section)
**Short novel DNA or RNA including primers, probes**: provide the sequences.	Yes	Appendix Table S1

Cell materials	Information included in the manuscript?	In which section is the information available? (Reagents and Tools Table, Materials and Methods, Figures, Data Availability Section)
**Cell lines:** Provide species information, strain. Provide accession number in repository **OR** supplier name, catalog number, clone number, and/**OR** RRID.	Not Applicable	
**Primary cultures:** Provide species, strain, sex of origin, genetic modification status.	Not Applicable	
Report if the cell lines were recently **authenticated** (e.g., by STR profiling) and tested for mycoplasma contamination.	Not Applicable	

Experimental animals	Information included in the manuscript?	In which section is the information available? (Reagents and Tools Table, Materials and Methods, Figures, Data Availability Section)
**Laboratory animals or Model organisms:** Provide species, strain, sex, age, genetic modification status. Provide accession number in repository **OR** supplier name, catalog number, clone number, **OR** RRID.	Not Applicable	
**Animal observed in or captured from the field:** Provide species, sex, and age where possible.	Not Applicable	
Please detail **housing and husbandry conditions**.	Not Applicable	

Plants and microbes	Information included in the manuscript?	In which section is the information available? (Reagents and Tools Table, Materials and Methods, Figures, Data Availability Section)
**Plants:** provide species and strain, ecotype and cultivar where relevant, unique accession number if available, and source (including location for collected wild specimens).	Yes	Methods
**Microbes:** provide species and strain, unique accession number if available, and source.	Yes	Methods

Human research participants	Information included in the manuscript?	In which section is the information available? (Reagents and Tools Table, Materials and Methods, Figures, Data Availability Section)
If collected and within the bounds of privacy constraints report on age, sex and gender or ethnicity for all study participants.	Not Applicable	

Core facilities	Information included in the manuscript?	In which section is the information available? (Reagents and Tools Table, Materials and Methods, Figures, Data Availability Section)
If your work benefited from core facilities, was their service mentioned in the acknowledgments section?	Yes	Acknowledgements

Design

Study protocol	Information included in the manuscript?	In which section is the information available? (Reagents and Tools Table, Materials and Methods, Figures, Data Availability Section)
If study protocol has been **pre-registered, provide DOI in the manuscript**. For clinical trials, provide the trial registration number **OR** cite DOI.	Not Applicable	
Report the **clinical trial registration number** (at ClinicalTrials.gov or equivalent), where applicable.	Not Applicable	

Laboratory protocol	Information included in the manuscript?	In which section is the information available? (Reagents and Tools Table, Materials and Methods, Figures, Data Availability Section)
Provide DOI OR other citation details if **external detailed step-by-step protocols** are available.	Not Applicable	

Experimental study design and statistics	Information included in the manuscript?	In which section is the information available? (Reagents and Tools Table, Materials and Methods, Figures, Data Availability Section)
Include a statement about **sample size** estimate even if no statistical methods were used.	Yes	Methods
Were any steps taken to minimize the effects of subjective bias when allocating animals/samples to treatment (e.g. **randomization procedure**)? If yes, have they been described?	Not Applicable	
Include a statement about **blinding** even if no blinding was done.	Yes	Methods
Describe **inclusion/exclusion criteria** if samples or animals were excluded from the analysis. Were the criteria pre-established?<br><br>If sample or data points were omitted from analysis, report if this was due to attrition or intentional exclusion and provide justification.	Yes	Methods
For every figure, are **statistical tests** justified as appropriate? Do the data meet the assumptions of the tests (e.g., normal distribution)? Describe any methods used to assess it. Is there an estimate of variation within each group of data? Is the variance similar between the groups that are being statistically compared?	Yes	Figure legend, methods

Sample definition and in-laboratory replication	Information included in the manuscript?	In which section is the information available? (Reagents and Tools Table, Materials and Methods, Figures, Data Availability Section)
In the figure legends: state number of times the experiment was **replicated** in laboratory.	Yes	
In the figure legends: define whether data describe **technical or biological replicates**.	Yes	

Ethics

Ethics	Information included in the manuscript?	In which section is the information available? (Reagents and Tools Table, Materials and Methods, Figures, Data Availability Section)
Studies involving **human participants**: State details of **authority granting ethics approval** (IRB or equivalent committee(s), provide reference number for approval.	Not Applicable	
Studies involving **human participants**: Include a statement confirming that **informed consent** was obtained from all subjects and that the experiments conformed to the principles set out in the WMA Declaration of Helsinki and the Department of Health and Human Services Belmont Report.	Not Applicable	
Studies involving **human participants:** For publication of **patient photos**, include a statement confirming that consent to publish was obtained.	Not Applicable	
Studies involving experimental **animals**: State details of **authority granting ethics approva**l (IRB or equivalent committee(s), provide reference number for approval. Include a statement of compliance with ethical regulations.	Not Applicable	
Studies involving **specimen and field samples:** State if relevant **permits** obtained, provide details of authority approving study; if none were required, explain why.	Not Applicable	

Dual Use Research of Concern (DURC)	Information included in the manuscript?	In which section is the information available? (Reagents and Tools Table, Materials and Methods, Figures, Data Availability Section)
Could your study fall under dual use research restrictions? Please check biosecurity documents and list of **select agents and toxins** (CDC): https://www.selectagents.gov/sat/list.htm	Not Applicable	
If you used a select agent, is the security level of the lab appropriate and reported in the manuscript?	Not Applicable	
If a study is subject to dual use research of concern regulations, is the name of the **authority granting approval and reference number** for the regulatory approval provided in the manuscript?	Not Applicable	

Reporting

The MDAR framework recommends adoption of discipline-specific guidelines, established and endorsed through community initiatives. Journals have their own policy about requiring specific guidelines and recommendations to complement MDAR.

Adherence to community standards	Information included in the manuscript?	In which section is the information available? (Reagents and Tools Table, Materials and Methods, Figures, Data Availability Section)
State if relevant guidelines or checklists (e.g., **ICMJE, MIBBI, ARRIVE, PRISMA**) have been followed or provided.	Not Applicable	
For **tumor marker prognostic studies,** we recommend that you follow the **REMARK** reporting guidelines (see link list at top right). See author guidelines, under 'Reporting Guidelines'. Please confirm you have followed these guidelines.	Not Applicable	
For **phase II and III randomized controlled trials**, please refer to the **CONSORT** flow diagram (see link list at top right) and submit the CONSORT checklist (see link list at top right) with your submission. See author guidelines, under 'Reporting Guidelines'. Please confirm you have submitted this list.	Not Applicable	

Data Availability

Data availability	Information included in the manuscript?	In which section is the information available? (Reagents and Tools Table, Materials and Methods, Figures, Data Availability Section)
Have **primary datasets** been deposited according to the journal's guidelines (see 'Data Deposition' section) and the respective accession numbers provided in the Data Availability Section?	Not Applicable	
Were **human clinical and genomic datasets** deposited in a public access-controlled repository in accordance to ethical obligations to the patients and to the applicable consent agreement?	Not Applicable	
Are **computational models** that are central and integral to a study available without restrictions in a machine-readable form? Were the relevant accession numbers or links provided?	Not Applicable	
If publicly available data were reused, provide the respective **data citations in the reference list**.	Not Applicable	
